# Sea star populations diverge by positive selection at a sperm-egg compatibility locus

**DOI:** 10.1002/ece3.487

**Published:** 2013-02-06

**Authors:** Jennifer M Sunday, Michael W Hart

**Affiliations:** Department of Biological Sciences, Simon Fraser UniversityBurnaby, British Columbia, Canada

**Keywords:** Bindin, gamete compatibility loci, positive selection, sea star, sexual conflict, sexual selection

## Abstract

Fertilization proteins of marine broadcast spawning species often show signals of positive selection. Among geographically isolated populations, positive selection within populations can lead to differences between them, and may result in reproductive isolation upon secondary contact. Here, we test for positive selection in the reproductive compatibility locus, bindin, in two populations of a sea star on either side of a phylogeographic break. We find evidence for positive selection at codon sites in both populations, which are under neutral or purifying selection in the reciprocal population. The signal of positive selection is stronger and more robust in the population where effective population size is larger and bindin diversity is greater. In addition, we find high variation in coding sequence length caused by large indels at two repetitive domains within the gene, with greater length diversity in the larger population. These findings provide evidence of population-divergent positive selection in a fertilization compatibility locus, and suggest that sexual selection can lead to reproductive divergence between conspecific marine populations.

## Introduction

One of the most remarkable patterns to emerge from molecular genetic investigations of natural populations is the signature of diversifying selection in proteins involved in fertilization (Swanson and Vacquier 2002; Turner and Hoekstra 2008). Several nonmutually exclusive mechanisms have been proposed to explain these patterns, including sexually antagonistic coevolution (Vacquier et al. 1997; Gavrilets 2000; Galindo et al. 2003; Haygood 2004), arms races with pathogens (Vacquier et al. 1997), and reproductive character displacement among hybridizing species (Metz et al. 1998; Swanson and Vacquier 2002; Palumbi 2009). Under each of these mechanisms, the selective agents that lead to diversifying selection may act in a population-specific manner. If so, geographically isolated populations may undergo differential, or divergent, selection in the very proteins that confer reproductive compatibility (Gavrilets 2000). Such diversifying selection may thus be an important mechanism for reproductive isolation (Vacquier et al. 1997).

In broadcast spawning marine invertebrates, mate choice is primarily mediated by gamete surface proteins, providing a relatively simple system for understanding the evolution of reproductive isolation. The identification of these proteins in the genome of broadcast spawning species has lead to considerable evidence suggesting rapid evolution of these proteins in response to selection in multiple species. Signals of positive selection have been detected among sea urchins (Metz and Palumbi 1996; Biermann 1998; McCartney and Lessios 2004), abalone (Lee and Vacquier 1992; Galindo et al. 2003), turban snails (Hellberg and Vacquier 1999; Hellberg et al. 2012), mussels (Riginos and McDonald 2003; Springer and Crespi 2007), and oysters (Moy et al. 2008). Among sea urchin species, variation in pairwise reproductive compatibility was better explained by pairwise divergence in the sperm protein, bindin, than by neutral genetic markers, indicating that evolution in this protein has been particularly important in driving reproductive isolation between species pairs (Zigler et al. 2005).

The relatively simple mating systems of broadcast spawners are also good systems in which to analyze the origins of reproductive isolation at its earliest stage, among geographically separated populations of conspecifics. However, in comparison with the abundant evidence for among-species differences driven by selection, there is much less evidence implicating positive selection as the cause of population differences in reproductive compatibility loci. Differential presence or absence of alleles from clades found to be under positive selection was found among populations of *Echinometra oblonga* (Geyer and Palumbi 2003) and *Mytilus galloprovincialis* (Riginos et al. 2006; Springer and Crespi 2007), suggesting positive selection has occurred in some, but not all, populations. More traditional approaches using *F*_ST_ statistics has shown significant population differentiation of reproductive compatibility loci in *E. oblonga* (Geyer and Palumbi 2003), *M. galloprovincialis* (Riginos et al. 2006), and *Heliocidaris bajulus* (Hart et al. 2012), but not in *Strongylocentrotus franciscanus* (Debenham et al. 2000), *Echinometra lucunter* (Geyer and Lessios 2009), or *Heliocidaris erythrogramma* (Binks et al. 2012). Other evidence for population-level divergence in reproductive compatibility can be found from experimental work. Findings of higher fertilization rates between mates from the same local population compared to mates from different, geographically separated populations, in sea urchins (Biermann and Marks 2000), polychaetes (Styan et al. 2008), and oysters (Zhang et al. 2010), suggest that isolation can evolve between populations in allopatry.

**Figure 1:**
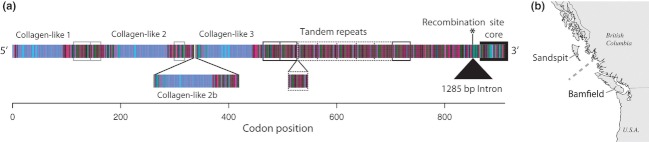
(a) Schematic diagram of bindin gene organization and coding sequence structure. (a) Main diagram (above) shows the 914-amino acid haplotype with the modal number of collagen-like copies and tandem repeats. Insets below show the extra collagen-like copy with 3' flanking region, and the extra tandem repeat, each found only once in our sample. Rectangles with solid black and grey borders show tandem repeat copies that are present in all alleles, dashed rectangles show tandem repeats that vary in copy number among alleles. Asterisk shows location of the single recombination site at codon site 855, black triangle denotes the 1285 bp intron at codon site 856, and solid black rectangle denotes the 135 bp invariant core. Colors in sequence are the default colors used in Se-al (v.2.0) (cyan = H, R, K; magenta = G, P, T, A, S; black = E, Q, D, N; green = L, V, M, I; blue = Y). (b) Map of study region and location of two main population samples. Dashed gray line represents location of phylogeographic break identified by Keever et al. (2009).

Here, we investigate population-level variation in a gamete compatibility locus in a broadcast spawning sea star, and test for divergent positive selection across populations. Our approach differs from previous work in that we directly compare lineages of alleles found within each population, and test for site-specific rates of positive selection that differ between populations. We studied the bat star, *Patiria miniata*, which is abundant and ecologically important in shallow marine habitats of the Pacific Ocean from southeastern Alaska to southern California (Rumrill 1989). We focused on two populations on either side (north and south) of an established phylogeographic break in this species, characterized previously using three classes of selectively neutral marker loci (Keever et al. 2009) and located in the region of Queen Charlotte Sound, British Columbia (at ca. 51°N; Fig. 1). The timing of this population split was dated to ca. 200,000 years b.p. using coalescent analysis, with low subsequent rates of gene flow between the populations (McGovern et al. 2010), and low potential for contemporary dispersal of planktonic larvae across Queen Charlotte Sound (J. M. Sunday et al., unpubl. ms.). We analyzed population variation in the gene encoding the sperm protein, bindin (Patiño et al. 2009). Bindin is expressed in the acrosomal vesicle of the sperm head, and interacts with a glycoprotein receptor on the egg surface (Kamei and Glabe 2003). This interaction is strongly species-specific among several sea urchins (reviewed in Vacquier et al. 1995), and bindin evolves under positive selection in many sea urchin taxa (reviewed in Swanson and Vacquier 2002; Palumbi 2009; Lessios 2011). Sea star bindin includes a short core region similar to the invariant core domain of bindin in sea urchins, but most of the sea star bindin coding sequence is structurally distinctive from sea urchins and consists of several types of long repetitive motifs (Fig. 1; see also Patiño et al. 2009).

We report a remarkable level of intraspecific variation in this repetitive structure, which we describe and analyze in detail. We find a significant difference in bindin sequences between the two populations, including many single nucleotide polymorphisms that encode amino acid substitutions, as well as a large number of insertion–deletion differences in coding sequence length (and protein size). Most importantly, we show that nucleotide differences are most likely a response to selection that favors amino acid substitutions (positive selection) at different sites among lineages of alleles in the northern and southern populations. We identify those sites, and develop a series of hypotheses pertaining to the mechanism of selection and population divergence.

## Methods

### Bindin sampling and alignment

We analyzed bindin variation among 44 individual sea stars collected from populations North and South of the phylogenetic break. We sampled 20 northern individuals from Sandspit, Haida Gwaii (53° 14′ 28″N, 131° 50′ 00″W), and 20 southern individuals from Bamfield, Vancouver Island (48° 49′ 43″N 125° 08′ 05″W). Our quantitative analyses focus on the 40 individuals from those two populations, but for a qualitative comparison within each region we also sampled two northern individuals from Dunbar Inlet, Alaska (55° 04′ 09″N 132° 50′ 47″W), and two southern individuals from Fort Bragg, California (39° 24′ 32″N 123° 48′ 22″W). Genomic DNA was extracted from a single tubefoot of each individual using a 2× cetyltrimethyl ammonium bromide (CTAB) extraction (described in Keever et al. 2009). Most of the coding sequence for the mature bindin protein was amplified using the AEE and VLS primers developed by Patiño et al. (2009) yielding a polymerase chain reaction (PCR) product 2739–4008 bp in length. It includes the coding sequence for the amino end of the mature bindin protein, consisting mainly of multiple copies of several distinctive repetitive amino acid motifs, as well as a nonrepetitive amino acid sequence, an intron, and part of the invariant core domain at the carboxy end of the predicted protein sequence (see Fig.1).

PCR products were purified on agarose gels, extracted using the Qiagen (Toronto, Ontario, Canada) gel-extraction kit, and cloned using the Invitrogen (Burlington, Ontario, Canada) Topo-TA cloning kit. Both ends of six to 10 clones for each individual were sequenced using universal plasmid primers (ca. 700 bp from both ends of each cloned PCR product). From the consensus of those sequences, one or two alleles were identified per individual. Any unique nucleotide difference between an individual clone and the consensus sequence for that allele was scored as a sequencing error (estimated as 0.00128 per nucleotide). Because the sea star bindin coding sequence is considerably longer than sea urchin bindin, it was not possible to obtain the full-length sequence of an allele from the paired-end sequences of a single clone (as is often done in sea urchin bindin studies, e.g., Geyer and Lessios 2009). To complete the genotypes for each individual sea star, one clone for each allele was then fully sequenced using the following three custom internal primers: KLN-f: 5′-CCAGTGGAAGGGAAGCTAAACT-3′; QPA-F: 5′-GGAATCGGAGTCACAACCAGCGG-3′; and GML-R 5′-CGAAGCATACCGAAACAGC-3′.

The full-length sequences for all alleles were aligned using default settings of the protein alignment algorithm in MAFFT v.6 (Katoh et al. 2002), followed by minimal adjustments by eye in Se-Al v.2.1 (see Appendix S1). That alignment of 88 sequences included 161 singleton polymorphisms in the part of the gene sequenced using internal primers from one clone per allele. Among these, all polymorphisms that were unique to one of 88 alleles could represent real, but rare polymorphisms or sequencing errors. Because the frequency of these singleton polymorphisms (about 0.0013 per site) from the internal sequence data for single clones per allele was similar to the sequencing error rate (0.00128 per nucleotide) estimated from sequences for the ends of multiple clones per allele, we assumed that they were errors and recoded them with the consensus nucleotide for that site.

### Gene genealogy

We used the genetic algorithm for recombination detection (GARD) (Kosakovsky Pond et al. 2006) to screen the bindin alignment for evidence of recombination. We found a single recombination site 4 bp upstream of the beginning of the intron (Fig. 1), and divided our alignment of alleles into two partitions upstream and downstream of this site. Because the second partition downstream of this site is comprised mainly of the intron and the highly conserved core domain (with few variable codons), our phylogeny and analysis of positive selection was conducted using only the first partition, which includes the large majority of the coding region of the gene and almost all the amino acid polymorphisms we observed.

We constructed a genealogy for bindin using a Bayesian phylogenetic analysis with Markov chain Monte Carlo (MCMC) sampling, using Mr. Bayes v. 3.1.2. We rooted the gene tree using a single bindin allele from *P. pectinifera*, the sister species of *P. miniata*, as the outgroup. We ran the MCMC search for 10,000,000 steps, sampling every 1000 steps after a burn-in of 40,000. Independence from starting conditions and convergence was checked using Tracer v.1.5. We used a model of evolution with two substitution types and four rate categories of gamma-distributed rates across sites, based on previous identification of the HKY85 model of evolution using the Model Selection procedure implemented on the Datamonkey webserver (Delport et al. 2010). Model priors were uniform.

### Repetitive domain analysis

We expected to find two repetitive domains within the coding sequence of bindin based on previous work (Patiño et al. 2009). The first is a collagen-like repeat domain, which is made up of 12–16 KGKK(G/R)R motifs (Fig. 1). Together with their flanking region, these domains were themselves repeated twice in the sequence described in Patiño et al. (2009). Downstream of these collagen-like domains is a series of tandem repeats (Fig. 1). We defined repetitive domains from the longest allele in our alignment using Radar (http://www.ebi.ac.uk/Radar), which identified the collagen-like repeat domain described above, and a tandem repeat domain of 30–35 amino acids (corresponding to the B+C repeat types of Patiño et al. 2009). To investigate phylogenetic relationships among repeat units, and to identify patterns of gene conversion among them, we aligned each repeat unit within individual alleles, and combined this alignment across all alleles. We then constructed a neighbor-joining tree from the alignment of collagen-like copies (without their flanking regions) and another from the alignment of the tandem repeats. In the alignment of tandem repeats, we included all nine copies in the major tandem repeat region downstream of the third collagen-like domain, plus the additional copies identified in the flanking regions of the first and second collagen-like domains (Figs. 1 and 4).

### Tests of population structure

We used Arlequin 3.0 to estimate bindin differentiation (Φ_ST_) between the Sandspit and Bamfield populations under a Kimura two-parameter substitution model, and used the randomization test of the hypothesis that Φ_ST_ = 0. In addition, we compared bindin differentiation to differentiation estimated in two nuclear intron loci previously sampled from different individuals in the same two populations (Keever et al. 2009). These were: an intron of the alpha subunit of ATP synthetase (ATPS, Genbank accession: FJ850593–FJ850958, Bamfield, *n* (sequences) = 52; Sandspit, *n* = 58), and an intron of the glucose-6-phosphate isomerase gene (GPI, Genbank accession: FJ850243–FJ850592; Bamfield, *n* = 50; Sandspit, *n* = 54) (Keever et al. 2009). We selected these loci among other noncoding loci also sampled from these populations (microsatellite and mitochondrial markers) because they had high levels of polymorphism comparable to that observed in bindin (Keever et al. 2009). To allow comparisons between fixation indices calculated from different loci, we used a standardized fixation index (Φ'_ST_) obtained by dividing observed Φ_ST_ by the maximum possible Φ_ST_ for each locus (Hedrick 2005; Meirmans 2006). We used the standardization methods described in Bird et al. (2011) based on (i) the maximum observed genetic distance between any two haplotypes in the alignment, and (ii) the fragment length of each locus. These analyses treated gaps in the alignment as a fifth character state.

### Tests of positive selection

We tested for Darwinian positive selection among lineages found in each population using the branch-sites model in PAML v.4.4 (Yang 2007). This model fits relative rates of nonsynonymous to synonymous nucleotide changes (ω = dN/dS) for three different rate classes (ω_0_, ω_1_, ω_2_) representing sites experiencing purifying, neutral, and positive selection, and estimates the proportion of codons assigned to each class. The test compares two sets of branches or allelic lineages in the gene tree, which are assumed to share the same estimated rate of codon evolution at sites experiencing purifying selection (ω_0_ < 1.0) or neutral accumulation of substitutions (ω_1_ = 1.0), but differ at the third class of codons. Branches or lineages of alleles in the “foreground” set are assumed to experience positive selection (ω_2_ > 1.0), while all other branches or lineages in the gene tree (the “background” set) are assumed to experience purifying selection (with the same ω_0_ rate estimated for the first class of codons) or neutral accumulation of substitutions (ω_1_ = 1.0). The hypothesis of positive selection at some sites in the foreground (that is, ω_2_ significantly greater than 1.0), and significant divergence between foreground and background populations, can be tested by comparing the maximum-likelihood scores for the selection model (with ω_2_ estimated from the data) to the likelihood score for a null model (with ω_2_ fixed to 1.0). We used the likelihood ratio test of significance (as twice the difference in log-likelihood scores for the selection and null models) with the χ^2^ approximation and one degree of freedom.

We ran this analysis twice, using Bamfield and Sandspit lineages as foreground branches, respectively. We defined the foreground set of branches as all terminal branches of the gene tree leading to alleles found in either Bamfield or Sandspit plus internal branches subtending any clades of alleles that consisted only of alleles sampled from that population. From each model fit, we used the Bayesian estimator B (Yang et al. 2005) to identify those codons with a high posterior probability that ω > 1 in the foreground population.

Because the PAML branch-sites model and similar models of codon evolution were originally developed for quantifying patterns of selection among species, the underlying gene tree is assumed to be known (i.e., the gene tree is not inferred as part of the analysis of selection), and the inference of strong or weak selection may be highly sensitive to errors in the underlying gene tree. Phylogenetic analysis of alleles from multiple species that differ from each other by large numbers of substitutions will typically reveal a single most likely genealogy with relatively long internal branches and strongly supported clades (e.g., from bootstrapping), such that gene tree uncertainty may be low. In contrast, phylogenetic analysis of many alleles from one species, in which some pairs of alleles differ from each other by one or a few substitutions, will typically result in a posterior distribution of many slightly different genealogies in which many internal branches are short and bootstrapping percentages or other measures of clade support and topological confidence will be weak. Under a codon model of positive selection, these genealogies may lead to different inferences about the overall strength of selection, or about differences in selection among lineages or among codons. It may therefore be important to account for this kind of uncertainty in intraspecific analyses using codon models of selection like the branch-sites model. To explore the sensitivity of our results to uncertainty in the bindin gene tree topology, we repeated these pairs of PAML analyses using all the 10 most likely trees from the Bayesian posterior distribution of gene trees generated by our MrBayes analysis.

The branch-sites model overall is conservative to false positives (sites inferred to be under positive selection in the foreground), because the selection model is tested against a null model that allows the same sites to be under purifying selection in the background branches, but under neutral selection in the foreground branches (Zhang et al. 2005). For all analyses, we used pairwise deletion of missing nucleotide sites so that we included potential signals of positive selection (and differences among populations) at sites that were present in some alleles, but absent in others (a difference we inferred to represent mainly deletions of parts of the repetitive structure of the gene; see Results). This approach was crucial to our analysis because of the extensive length variation in both populations (see Results). This meant that some other codon-based tests of positive selection that use complete deletion rather than pairwise deletion of gap sites were able to characterize patterns of selection for only a small proportion of the alignment that lacked gaps (e.g., the Branch-site REL method of Kosakovsky Pond et al. 2011). As a result, these methods were relatively ineffective in detecting sites or lineages under selection. Similarly, a lack of fixed differences between the populations precluded use of some other methods that use gene trees to test hypotheses of positive selection (e.g., McDonald and Kreitman 1991).

In order to characterize the potential effects of selection on codon insertion–deletion variation, we also compared the number of indels in the coding region of bindin to the number of indels in the noncoding intron. We hypothesized that under a model of neutral selection, the number of indels should be proportionally equal to the length of the fragment in the coding and noncoding regions, respectively. From the full-length sequence alignment of 80 alleles, we counted the number of unique contiguous series of gap sites found in the coding sequence (e.g., a single codon insertion, or a 10-codon deletion, each were scored as one unique indel) and in the intron (same scoring as for coding sequence). We used a χ^2^ test (with Yates' correction for small sample sizes) of the expectation of equal frequency of indel occurrence per unit length of coding or noncoding sequence (in nucleotides).

## Results

### Polymorphism

The alignment of bindin genomic sequences including the intron was 4134 bp, with 196 variable sites. The coding region without the intron (Fig. 1) was 2853 bp, with 70 variable sites and 46 amino acid polymorphisms**.** Most of this variation occurred in the first partition upstream of the recombination site (at codon site 890, Fig. 1), where there were 69 variable sites and 45 amino acid polymorphisms. We found high haplotype diversity in this first partition, including 68 unique alleles among 88 sequenced gene copies.

**Figure 2:**
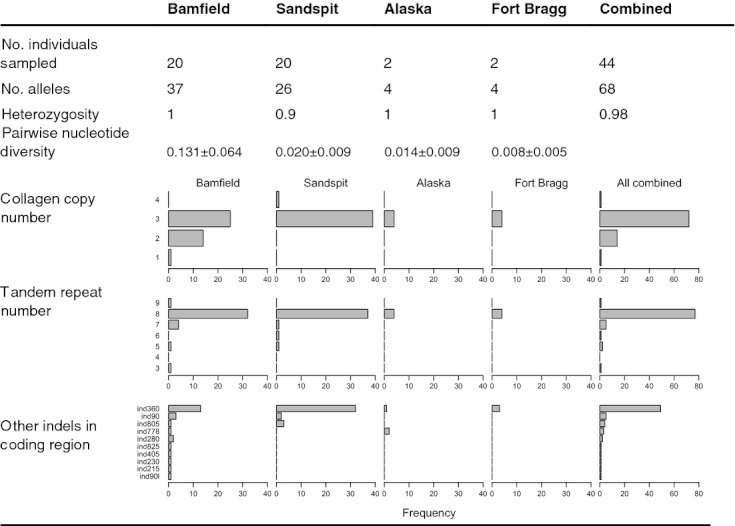
Summary of bindin diversity across sampled populations.

**Figure 3:**
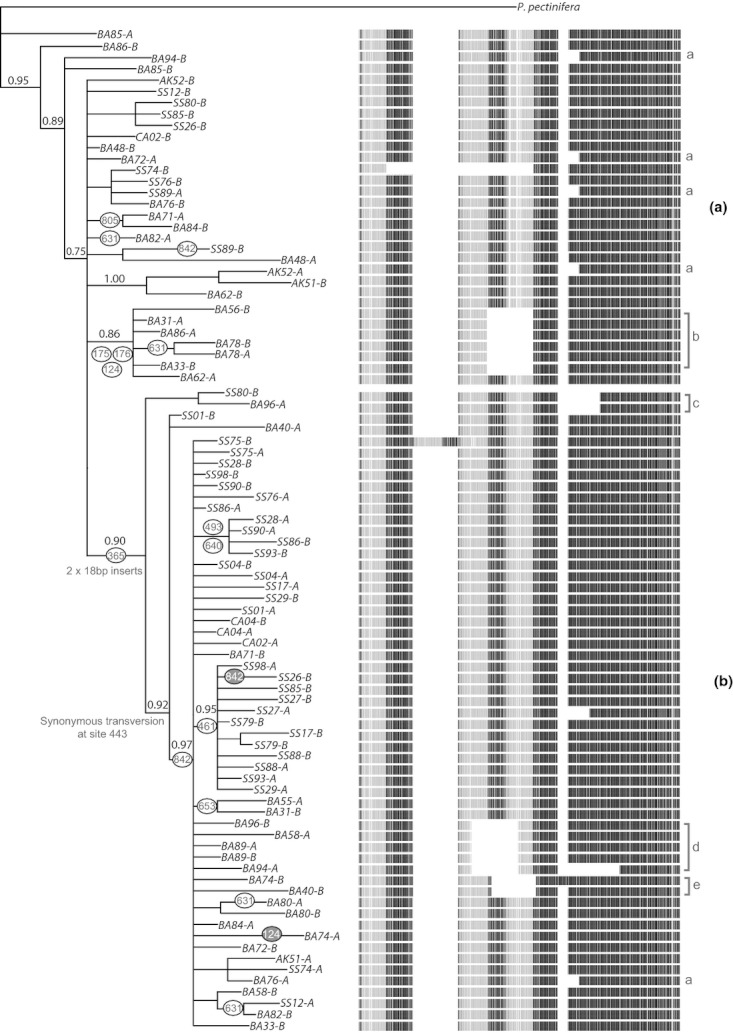
Consensus Bayesian gene tree and gene alignment for the first recombinant region of the bindin gene in *Patiria miniata*. The gene tree is annotated with the 11 codon sites under positive selection. Circled numbers refer to codon sites described in Table 1. Gray circles represent reversals. Two other differences are also annotated on the tree – the addition of two 18 bp inserts, which distinguishes the paraphyletic groups 1 and 2, and a single, but common synonymous transversion, which most members of haplotype group b share. Uncircled numbers above major clades denote Bayesian posterior probability of partition. Gaps in the gene alignment shows variation in repeat units, gray tone in sequences represents amino acid identity (light gray = H, R, K, G, P, T, A, S; dark gray = E, Q, D, N, L, V, M, I, Y), and letters a–d to the right indicate repeat-number variants that occurred multiple times (and are emphasized in the main text).

We found striking variation in the length of the gene, owing to variation in the number of repeat units in the repetitive domains. The collagen-like repeat units varied in number from 1 to 4 copies with a mode of 3 (Figs. 2 and 3). There was also polymorphism in the length of these collagen-like repeats, owing to insertions or deletions of the constituent KGKK(G/R)R repeat motifs. The tandem repeat motifs of 32–36 amino acids also varied in copy number from 3 to 9 copies, with a mode of 8 (Figs. 2 and 3). The alignment suggested that the 13 alleles with two collagen-like copies were not identical by decent. Instead, alleles with two collagen-like copies were consistently and unambiguously aligned to longer alleles (with three or four collagen-like copies) in one of three ways (variants b, d, and e in Fig. 2). This pattern strongly suggests that shorter alleles with fewer collagen-like domains were derived in parallel by several different types of deletion mutations.

Genetic diversity, heterozygosity, and repeat copy number polymorphism were greater in Bamfield compared with Sandspit (Fig. 2). Pairwise nucleotide diversity was sixfold greater in Bamfield compared with Sandspit, from which the same number of individuals were sampled (Fig. 2). Almost all of the copy number variation in the collagen-like domains occurred in the Bamfield sample (1–3 copies); in the Sandspit sample all alleles had three collagen-like domains except for a single Sandspit allele with four collagen-like copies (Fig. 2). Similarly, the Bamfield population had five length variants among the tandem repeats, while the Sandspit population had only three, two of which occurred in both populations (Fig. 2). There were 11 other indels which occurred either as variation in the number of KGKK(G/R)R repeat motifs within individual collagen-like repeats (*n* = 8), or which occurred in nonrepetitive regions (*n* = 3). Overall, most of the length variation occurred in the Bamfield population (Fig. 2). There were no fixed indel differences or nucleotide polymorphisms between the populations at nucleotide or indel sites.

### Bindin genealogy

The consensus bindin gene tree did not show an obvious strong pattern of lineage sorting between the two populations (reciprocal monophyly of large clades), although some small clades were unique to one or the other population (Fig. 3). The gene tree included a large clade of alleles characterized by two 6-codon insertions in the third collagen copy, as well as an Arg→Gly substitution at codon 365. Most alleles in this group also had a Pro→Glu transversion at codon 842 (Fig. 3). Alleles from both of these lineages were sampled in northern and southern populations (Fig. 3).

Although gaps in the alignment were not included as a character state in the phylogenetic reconstruction, some alleles with the same copy number variants were grouped together within clades, indicating that they also shared nucleotide polymorphisms (e.g., repeat variants b and c, Fig. 3). Other repeat variants did not form clades, but tended to be closely related to each other (variants d and e).

### Relationships among repeat paralogs

**Figure 4:**
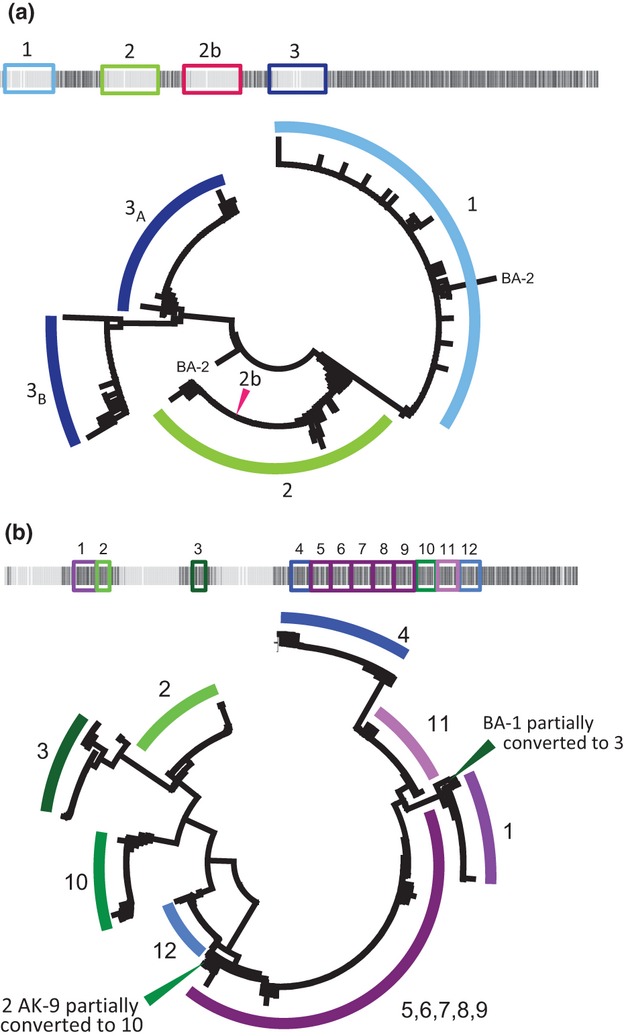
Neighbor-joining gene trees from alignments of (a) collagen-like copies and (b) tandem repeats across 44 individuals. In both, clades of repeat units match their location in the gene by number and color. In the collagen-like copy alignment (a), the location of repeat unit 2b is noted with a pink arrowhead, and the two copies of repeat unit 2 from Bamfield that show conversion with other copies (BA-2) are highlighted. In (b) the three tandem repeats that show partial or full gene conversion are highlighted (two from Alaska: AK-9; one from Bamfield: BA-1).

In the alignment of collagen-like domain copies, the neighbor-joining tree showed that copies 1, 2, and 3 formed strongly supported clades subtended by long internal branches, with no strong evidence of gene conversion among them (Fig. 4). The fourth collagen-like copy, which was only observed in one individual, was identical to copy 2 (labeled “2b” in Fig. 4). Further analysis showed that the flanking region downstream of copy 2b was also identical to that of copy 2 (Figs. 1 and 3). Collagen-like copy 3 had two forms, which differed by the two 6-codon insertions and the nucleotide transversion described above, defining a large clade of the gene tree, and these were more similar to one another than they were to copies 1 and 2. There were two unique collagen-like copies found in single individuals that showed evidence of partial gene conversion: copy 2 in an allele from Bamfield had a 15-bp region that was strongly similar and readily aligned to copy 3; and copy 2 of another Bamfield allele was almost identical to copy 1 (both labeled “BA-2” in Fig. 4a).

The neighbor-joining tree of tandem repeats indicated a complex history of duplications among these repeats (Fig. 4). Copies 5, 6, 7, 8, and 9 were strongly similar or identical to each other, consistent with relatively recent duplication. A single polymorphism was common although not fixed in copy 9. There were three apparent instances of gene conversion in the tandem repeats among individual alleles. Two of these instances involved sequence similarities that were out of phase with the repeat motif as defined by Radar, so that the affected alleles had strong sequence similarities involving parts of two adjacent repeat copies. In both these alleles, a region including parts of tandem repeats 9 and 10 was found to be identical to the analogous parts of repeats 10 and 11. Both these cases occurred within alleles sampled from Alaska. In the third instance, tandem repeat 1 in a Bamfield allele (downstream of the first collagen-like repeat) was found to be identical to repeat 3 (downstream of the third collagen-like repeat).

### Bindin population structure

Population differentiation (Φ_ST_ = 0.220) between Bamfield and Sandspit was strong and highly significant (*P* < 0.001). Standardized genetic distance in bindin (Φ'_ST_) was 0.462 (standardized by maximum genetic distance) or 0.454 (standardized by fragment length). By contrast, Φ'_ST_ for the ATPS and GPI loci were 0.0545 and 0.0940 (standardized by maximum genetic distance) or 0.0310 and 0.0697 (standardized by fragment length). Thus, standardized genetic distance was nearly an order of magnitude larger across these two populations in bindin compared with the noncoding intron sequences.

### Tests of positive selection

The branch-site model of positive selection in PAML was somewhat sensitive to which tree was used (among the top 10 most probable trees from the Bayesian posterior distribution). We therefore report all the results in the online Table S1 and summarize the general findings below.

**Figure 5:**
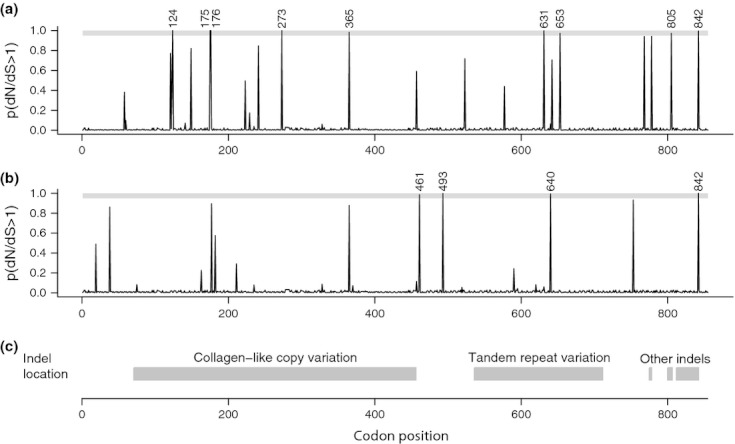
Site-by-site probability of positive selection, and location of indels, in the first exon of bindin in *Patiria miniata*. Peaks show for each codon the Bayes-empirical-Bayes estimate of the posterior probability of belonging to the positively selected class of sites (with ω > 1) and high rates of amino acid substitution along the foreground branches from the maximum-likelihood analysis. (a) Results with the Bamfield population in the foreground, (b) results with the Sandspit population in the foreground, both using the most visited tree from the posterior distribution of most likely gene trees generated by the MrBayes analysis (for results from other gene trees, see Fig. S1). Height of the curve shows the probability dN/dS >1 for each site along the length (*x*-axis) of the first recombinant region. Gray bands show the 95% probability range. Numbered peaks indicate sites with >95% probability dN/dS >1. (c) Distribution of indels.

With Bamfield alleles and clades in the foreground, significant positive selection was detected for all trees, as the likelihood ratio tests rejected the null model in all cases (Table S1). The Bayes empirical Bayes estimator detected nine sites under positive selection with a high ω > 1 (Fig. 5). We found the same sites under positive selection in this population for all 10 gene trees tested (Fig. S1).

With Sandspit alleles and clades in the foreground, significant positive selection was only detected in tests using two of 10 gene trees (Table S1). For the other eight trees, the positive selection model (with an estimated value of ω_2_ > 1) was not a significantly better fit to the data and the gene tree in comparison to the neutral model (with the value of ω_2_ fixed to 1.0 rather than estimated). For the two trees which lead to a significant signal of positive selection, the Bayes empirical Bayes estimator detected four sites under positive selection with a high ω > 1 (Fig. 5). These same four sites were also detected in nonsignificant selection models by the Bayes empirical Bayes estimator (when a subset of models were run sufficiently long for this estimation to be made; Fig. S1), but with lower posterior probabilities of falling into the positively selected class of sites. This indicates that these four sites may have notably high dN/dS when other gene trees are used, but the selection model with an extra estimated parameter was not more likely than the neutral model with a fixed value of ω = 1 at those sites.

Notably, the sites that were strongly inferred to be under positive selection in Bamfield differed from the sites that were weakly inferred to be under selection in Sandspit (Table 1, Fig. 5). For only one site (codon 842) did we find relatively strong evidence of positive selection in both populations. Of the other eight sites under positive selection in Bamfield, six were under purifying selection in Sandspit; similarly, three of the four sites putatively identified as experiencing positive selection in Sandspit were under purifying selection in Bamfield (Table 1). Also of note is the location of these sites in the gene structure. Of the 11 total sites identified as positively selected in one or both populations, five were associated with the third collagen-like domain, three occurred in the tandem repeat region, and three occurred downstream of the last tandem repeat copy in the nonrepetitive coding sequence (Table 1; Fig. 5).

**Table 1:** Details of codon sites under positive selection

Population where dN > dS	Codon position	Amino acid	Hydrophobicity	Charge	Occ. in Sandspit	Occ. in Bamfield	*P* (dN > dS) in Sandspit	*P* (dN > dS) in Bamfield	Location in gene
Bamfield	124	E	+	–	40	31	0.006	1	Flanking region of first collagen
		G	+		0	7			
		K	+	+	0	1			
Bamfield	175	A	–		40	32	0.019	1	Flanking region of first collagen
		V	+		0	7			
Bamfield	176	K	+	–	40	32	0.006	0.997	Flanking region of first collagen
		E	+	–	0	7			
Bamfield	365	R	+	+	8	12	0.693	0.995	A to B transition in third collagen
		G	+		32	13			
Bamfield	631	R	+	+	28	33	0.076	0.998	Fifth tandem repeat
		K	+	+	1	5			
Bamfield	653	I	–		40	37	0.024	0.972	10th tandem repeat
		L	–		0	2			
Bamfield	805	I	–		37	37	0.025	0.973	Nonrepeat
		F	–		0	2			
Bamfield and Sandspit	842	P	–		10	21	0.999	0.999	Nonrepeat
		Q	+		30	18			
Sandspit	461	A	–		28	40	0.988	0.001	After third collagen
		T	+		12	0			
Sandspit	493	E	+	–	36	40	0.999	0.001	Fourth tandem repeat
		D	+	–	4	0			
Sandspit	640	K	+	+	36	40	0.994	0.056	10th tandem repeat
		M	–		4	0			

Occ., occurence among the 40 samples from each focal population.

### Number of insertion–deletion differences in coding versus noncoding region

We found a total of 22 different types of indels (Fig. 3) in our alignment of coding sequences (2850 bp in length) and only two in the noncoding intron (1285 bp in length). This approximately fivefold higher density of indels among coding sequences (0.0077 per bp) compared with introns (0.00155 per bp) was significant (χ^2^_df = 1_ = 4.76, *P* < 0.05).

### Covariation among sites under selection and among repeat variants

Some of the polymorphisms at sites under positive selection (codons 124, 175, 176) co-occurred with each other in the same Bamfield alleles. These were relatively short alleles with only two collagen-like copies (Fig. 2). Similarly, polymorphisms at codons 493 and 640 co-occurred within the Sandspit population, and polymorphisms at site 365 covaried with the pair of six-codon insertions (Fig. 2).

There was no covariation between collagen copy variation and tandem repeat variation among alleles (Fig. 3). Among the 15 alleles that varied from the modal number of collagen copies (3), and the 10 that varied from the modal number of tandem repeat copies (8), there were only two alleles that varied at both. This was not significantly different from the expectation if repeats in the two domains vary independently (χ^2^_df = 1_ = 0.1382, *P* > 0.5**).**

### Co-occurrence of sites under selection and indels

All the sites found to be under positive selection in Bamfield, and two of the four sites that are probably under positive selection in Sandspit, occurred in regions of indel variation (Fig. 5). Most of these were found among the tandem repeat domains, and two codon sites were found in parts of the alignment where there were smaller indels (Fig. 5). One of these indels included codon 805, under positive selection in Bamfield. This indel was common at Sandspit (*n* = 5 alleles), but rare at Bamfield (*n* = 1). The other indel present in both populations included codon 842, a site under positive selection in both populations. In addition to these, one small indel found only in Sandspit and Alaskan alleles included codon 778, which might be under positive selection in Bamfield (with a marginally nonsignificant posterior probability, *P* = 0.94) (Fig. 5).

## Discussion

We find a different pattern of selection in the bindin locus in two geographically separated populations of a single species. Our results specifically indicate that there are sites under positive selection (dN > dS) in one population that are under purifying or neutral selection in the other population (dN ≤ dS), and the sites found to be under positive selection differed between the two populations. Although the signal of positive selection in the Sandspit population was sensitive to the gene tree used, our results clearly indicate sites under positive selection in Bamfield that are not under selection in Sandspit. These findings strongly suggest that selection favors different bindin alleles in the two populations. To the extent that the patterns differ between the two populations (fewer sites under selection in Sandspit, weaker overall evidence for positive selection among those northern alleles and lineages), the populations may be experiencing different processes as well (weaker selection, or a different mode of selection, acting on northern bindin alleles).

This strong population divergence is remarkable given the very high level of within-population bindin diversity in *P. miniata*, including both single nucleotide polymorphisms and indel variation. This overall polymorphism is notably high compared with studies of other broadcast spawning species with comparable sample sizes (Table 2). The nucleotide variation is partitioned significantly between Sandspit and Bamfield, and the standardized genetic distance is much greater than that observed for neutral markers, suggesting that the divergence in bindin has been greater than that expected by genetic drift alone. We consider how these findings compare with theoretical expectations and to previous observations of polymorphism at reproductive loci, and contribute to our understanding of speciation in the marine environment.

**Table 2:** Number of unique alleles and indel variants in gamete recognition genes of broadcast spawning marine invertebrates

Species	Study	Number of unique alleles (sample size)	Number of repeat motif copy variants	Positive selection?
*Patiria miniata*	This study	68 (88)	18	Yes
*Crassostrea gigas*	Moy et al. (2008)	Unknown	4	Yes
*Strongylocentrotus purpuratus*	Levitan and Stapper (2009)	40 (135)	2	–
*S. franciscanus*	Debenham et al. (2000)	14 (134)	0	No
	Levitan and Ferrell (2006)	15 (254)	0	No
*Echinometra mathaei*	Palumbi (1999)	15 (85)	3	Yes
*E. oblonga*	Geyer and Palumbi (2003)	40 (82)	2	Yes
*Echinometra sp. C*	Geyer and Palumbi (2003)	37 (84)	0	Yes
*E. lucunter*	Geyer and Lessios (2009)	96 (246)	6	Yes
*Echinometra viridis*	McCartney and Lessios (2004)	28 (31)	2	Weak
*Echinometra vanbrunti*	McCartney and Lessios (2004)	17 (17)	2	No

This work builds on previous studies of population differentiation in reproductive proteins driven by positive selection. In the M7 lysin locus of the mussel *M. galloprovincialis*, one clade (G_D_) of alleles which showed significant positive selection was found at different frequencies across populations, suggesting that the selective processes were localized (Riginos et al. 2006; Springer and Crespi 2007). Similarly, in the sea urchin *E. oblonga*, one clade (clade 3) of bindin alleles occurred exclusively in island populations with a sympatric congener. In this case, positive selection was detected only when clade 3 was included with other clades in an analysis, indicating that positive selection caused differentiation of clade 3 from other *E. oblonga* bindin alleles (Geyer and Palumbi 2003). In a third case study, no population differentiation was detected among bindin alleles of *S. franciscanus* sampled along the Pacific coast of North America between Alaska and southern California (Debenham et al. 2000). Taken together, while many studies have detected positive selection in fertilization proteins within populations and across species (reviewed by Swanson and Vacquier 2002), surprisingly few studies appear to have tested for divergence between groups of alleles that characterize geographically separated populations. Our observations of population differentiation driven by positive selection suggest that future phylogeographic analyses of gamete recognition genes could reveal population-level patterns associated with reproductive divergence.

One striking feature in our bindin alignment was the high number of indels in repeat regions of the gene. Indel variation has previously been observed in gamete recognition loci of broadcast spawners, both across (Metz and Palumbi 1996; Biermann 1998; Zigler et al. 2005) and within species (Table 2). Indeed, complex repetitive protein domain structure and insertion–deletion of repeats appears to be a hallmark of species-level diversity of gamete recognition loci under positive selection (Palumbi 1999; McCartney and Lessios 2004; Levitan and Stapper 2009), and may allow relatively rapid diversification through unequal crossing over (Minor et al. 1991; Vacquier et al. 1995; Metz and Palumbi 1996). Positive selection on indel variation has been identified in sperm proteins of primates (Podlaha and Zhang 2003) and rodents (Podlaha et al. 2005), and indels were also found to be associated with regions of positive selection in seminal fluid proteins of *Drosophila* (Schully and Hellberg 2006). The occurrence in our study of indel variation within regions of the gene that include codons under positive selection further suggests that indel mutations may be selected for their effects on protein function similar to selection acting on other forms of functional variation caused by positively selected amino acid substitutions.

If the number of collagen-like domains or the number of tandem repeat copies are functionally significant and under selection, that selection may differ between Bamfield and Sandspit. The most common number of collagen-like copies is three, and in the Bamfield population, alleles with only two copies appear to have evolved convergently in three different ways. This finding was supported by the differential alignment of the 2-copy variants, as well as the finding of one 2-copy variant within a distinct clade in the gene tree (clade b of Fig. 2). This is in stark contrast to the Sandspit population, in which only one allele was sampled with other than three collagen-like copies. Notably, this rare variant was found in the population with the weaker evidence for positive selection on individual codons, and had more (four) rather than fewer collagen-like domains. Tandem repeat number was also somewhat divergent between populations, with some private alleles found in both populations. The distribution of tandem repeat copy number on the consensus gene tree suggests that tandem repeat indels also occurred multiple times in parallel. Because the gene trees were inferred using pairwise deletion of missing characters, they are informed by nucleotide polymorphisms only. It therefore remains possible that the evolution of tandem repeat copy number was more conserved through time than seems apparent from the consensus tree. However, without an *a priori* model of molecular evolution that weighs indel variation relative to nucleotide polymorphism, this issue is not easily resolvable.

Although we looked for gene conversion across repeats, it does not appear to be a major process driving divergence among these *P. miniata* populations. If it were, we might expect repeats within alleles to be more similar to each other than to repeats in the same part of the coding sequence alignment in other alleles. Instead, almost all tandem repeat and collagen-like domain copies formed clades that implied homology of those domain copies to each other. However, our focus on the Bamfield and Sandspit populations does not rule out the possibility of finding more evidence of gene conversion in other populations. Of the two Alaskan individuals sampled, each had one allele with a fully converted repeat copy. Concerted evolution has been identified across species in the vitelline envelope receptor for lysin in molluscs (Swanson and Vacquier 1998), and may yet be an important mechanism of divergence in *P. miniata* bindin at the population level.

### Selective mechanisms

Several potential mechanisms have been proposed to explain observations of positive selection in fertilization proteins, some of which also predict high allelic diversity and population-level variation. First, a sexual conflict may exist in broadcast spawners under conditions of high sperm competition, in which males are selected to maximize encounters and compatibility of sperm with eggs, and females are selected to moderate encounters between eggs and sperm, possibly through increased choosiness for specific male genotypes, in order to reduce female risk of polyspermy (fertilization of individual eggs by multiple sperm). Theory suggests that such a conflict over the rate of sperm-egg encounters can lead to an arms race between the sexes, in which successive adaptations in the female reproductive system to reduce polyspermy rates are counteracted by specific male adaptations. Such an arms race operating independently in geographically isolated populations is expected to lead to population-specific male and female adaptations, population divergence of reproductive traits, and (potentially) to reproductive isolation between members of such populations (Gavrilets 2000; Gavrilets and Waxman 2002). Such a process is of obvious interest to evolutionary ecologists as a source of selection leading to the formation of new species.

Our findings of positive selection at different codon sites across populations are consistent with the evolutionary chase outcome of sexual conflict (Gavrilets and Waxman 2002). Divergent female reproductive traits may have evolved in northern and southern *P. miniata* populations, leading to differential selection on male bindin genotypes. However, because allele diversity was also high in both populations, and neither selective sweeps nor gene conversion were apparent, these findings are also consistent with the polymorphism-maintenance outcome of sexual conflict (Haygood 2004). Hence, these alleles may be maintained by negative frequency dependence, with different novel genotypes selected in either population (Haygood 2004; Tomaiuolo and Levitan 2010). While we have no direct evidence for a sexual conflict via polyspermy in this species, *P. miniata* is known to live in at high adult spatial densities in Bamfield (2.6–3.5 individuals/m^2^, Rumrill 1989) and California (1.9 individuals/m^2^, Schroeter et al. 1983), similar to the range of high densities that are associated with sperm competition and polyspermy in the urchin *S. franciscanus* (Levitan and Ferrell 2006). The stronger signal of positive selection in the Bamfield sample (compared with Sandspit) is also consistent with greater expected response to selection in the population with the larger effective population size (Keever et al. 2009; McGovern et al. 2010).

Other mechanisms of positive selection are worthy of consideration, namely reproductive character displacement, sexual selection (without conflict per se), and immunological defense (Vacquier et al. 1997). Reproductive character displacement as a result of reinforcement selection against hybrids is not a likely mechanism in *P. miniata* as there are no closely related species in its range. The most closely related and sympatric species that are abundant and likely to have similar spawning seasons are from different taxonomic families (the asteropseid *Dermasterias imbricata*; several solasterid species in the genera *Crossaster*, *Solaster*; Mah and Foltz 2011; Mah and Blake 2012). Because hybridization is unlikely across families (e.g., Nakachi et al. 2006), and introgression of haplotypes from other species has not been observed in extensive phylogeographic surveys of mtDNA (Keever et al. 2009), *P. miniata* seem unlikely to experience selection against the formation of low-fitness hybrids with other species. Sexual selection without conflict over polyspermy rates could result in directional selection driven by sperm competition and female choice (e.g., for more compatible males), and indeed divergence between populations may represent different molecular pathways toward the same optimal male phenotype. However, under this type of sexual selection, a single optimal male genotype is expected to be selected toward fixation. Without the trade-offs associated with an accompanying sexual conflict, it is difficult to explain the high within-population bindin variability on the basis of sexual selection among males alone. Finally, immunological defense is a potential driver of evolution in egg surface receptors, and pathogens may drive both diversification and positive selection in similar conflict scenarios (Hughes and Nei 1988), via selection on sperm ligands to match changing female receptors (Vacquier et al. 1997). Hence, immunological defense cannot be ruled out as a potential alternative explanation for the patterns observed. Whichever mechanism(s) may be involved, population-level divergence is an important outcome with potential consequences for incipient speciation.

### Potential function of bindin coding sequence variation

Although the functional significance of bindin amino acid and indel variation remains to be tested, it is difficult to imagine that the polymorphism in *P. miniata* bindin is neutral to fertilization success. Variation in reproductive success is correlated with quantitatively lower levels of sequence variation at gamete recognition loci of other species (Palumbi 1999; Levitan and Ferrell 2006; Levitan and Stapper 2009). For example, complete gamete incompatibility between congeneric sea urchins can be associated with as few as 8–10 bindin amino acid substitutions (and perhaps correlated changes in the egg bindin receptor) (Zigler et al. 2005). Importantly, experimental studies of species-specific molecular responses highlight repeat elements in bindin as having a possible function in species recognition (Vacquier et al. 1995).

## Conclusion

We find high intraspecific polymorphism and evidence for divergent positive selection between two populations at the gene encoding the male-expressed gamete reproductive protein bindin. We also find evidence that indel variation is subject to diversifying selection across these populations. These findings suggest that selection favors different bindin alleles in the two populations, most likely through female choice via egg cell surface molecules. This divergence may represent early stages of incipient speciation based on processes intrinsic to *P. miniata* populations, rather than in response to extrinsic selective pressures associated with, for example, environmental or biotic differences between the two habitats occupied by those populations. Study of the functional role of bindin variation is required to understand the mechanisms maintaining polymorphism within populations and leading to divergence between populations. The potential for nonecological processes to drive reproductive isolation among populations has profound implications for our understanding of the speciation process, particularly in the ocean where strong physical barriers are to gene flow are sparse, and the origins of reproductive isolation at its earliest stages are not obvious.

## Data Archive

Population sets of sequences have been archived in GenBank (accession numbers: KC503670-KC503757).

## Supporting Information

**Figure S1.** Site-by-site Bayes-empirical-Bayes probability of positive selection in the first exon of bindin in *Patiria miniata* for all gene trees investigated.

**Table S1.** Branch-sites model results from PAML analysis.

**Appendix S1.** Full sequence alignment of bindin gene, with intron. Intron begins at basepair 3137, and ends at basepair 4420; recombination site is located between basepairs 3132 and 3133.
