# Supplementary material for: Sea star populations diverge by positive selection at a sperm-egg compatibility locus
Source: Ecol Evol. 2013 Feb 6;3(3):640–54. doi: 10.1002/ece3.487 (PMC3605852; doi:10.1002/ece3.487)
Supplement: Supplementary file 1 [file ece30003-0640-SD1.pdf]

Table S1. Branch-sites model results from PAML analysis. Bold log-likelihood differences indicate significant values in a log-likelihood ratio test. Grey and missing values are from model comparisons where selected models did not resolve a Dn/Ds parameter > 1 within the first 10 rounds. Grey values were obtained by running these unresolved models for the maximum of 200 rounds.

	log likelihood of null model: foreground omega fixed at 1	log likelihood of selection model: foreground omega estimated	log likelihood difference (sel. model– null model)	estimated foreground omega	proportion sites at foreground omega w/ background at 0	proportion sites at foreground omega w/ background at 1
Bamfield in foreground						
tree 1	-4506.5	-4539	32.6	15.6	0.01566	0.00165
tree 2	-4549.9	-4581.2	31.3	13.6	0.01543	0.00155
tree 3	-4539.7	-4570.2	30.5	14.2	0.01415	0.00147
tree 4	-4526.1	-4557.2	31.1	13.4	0.01562	0.00160
tree 5	-4537.3	-4577.5	40.2	16.1	0.01440	0.00150
tree 6	-4515.0	-4546.4	31.3	14.0	0.01526	0.00160
tree7	-4523.7	-4555.6	31.8	14.1	0.01643	0.00165
tree8	-4514.9	-4554.0	39.1	14.6	0.01338	0.00374
tree9	-4550.5	-4589.3	38.8	15.8	0.01446	0.00150
tree10	-4529.0	-4560.7	31.7	13.9	0.01554	0.00158
Sandspit in foreground						
tree 1	-4521.6	-4539	17.4	21.7	0.00853	0.00099
tree 2	-4543.4	-4542.0	-1.4	0	0	0
tree 3	-4539.2	-4539.0	-0.2	0	0	0
tree 4	-4531.1	-4530.7	-0.4	0	0	0
tree 5	-4558.5	-4576.2	17.7	19.4	0.00924	0.00104
tree 6						
tree7						
tree8						
tree9						

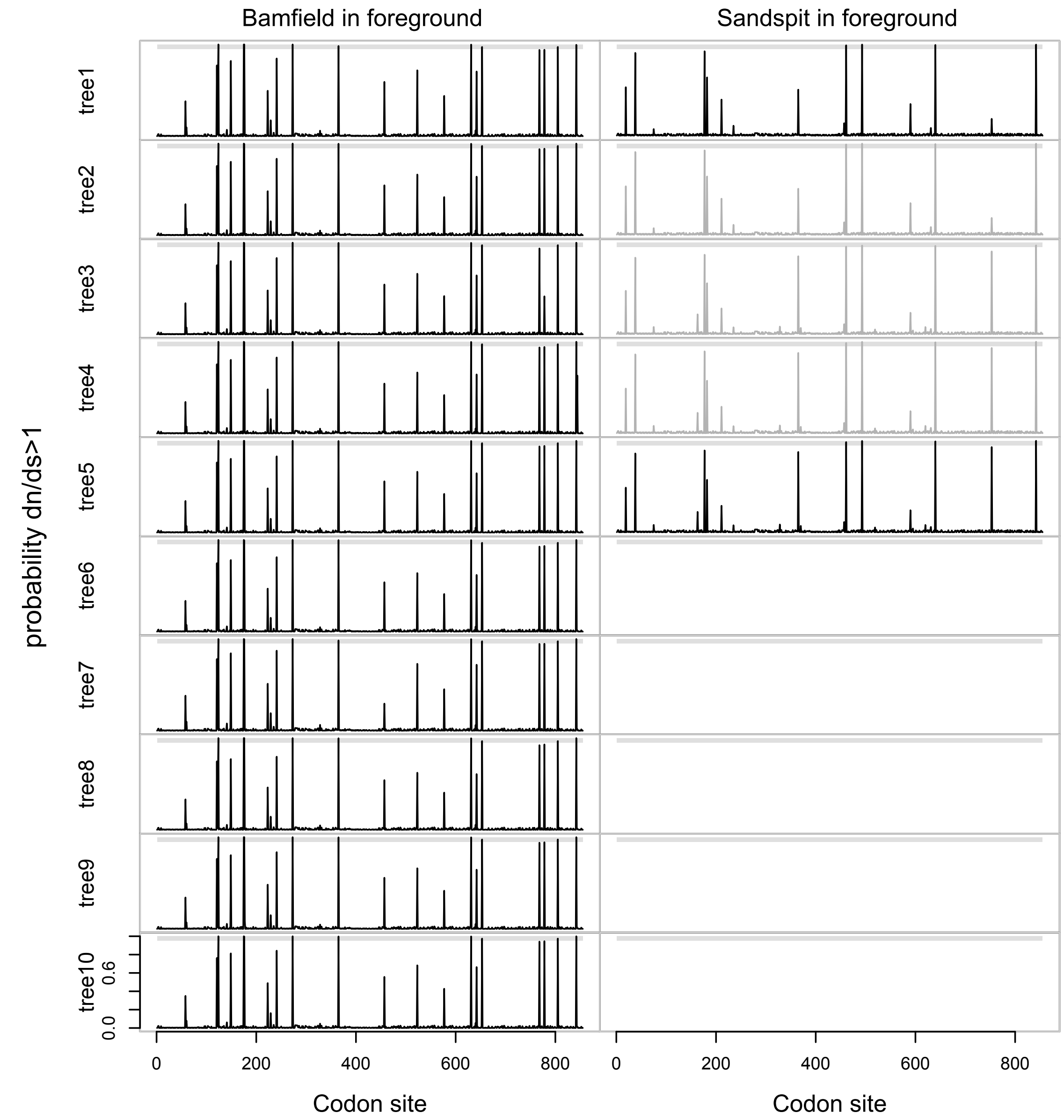

Figure S1. Site-by-site Bayes-empirical-Bayes probability of positive selection in the first exon of bindin in *P. miniata* for all gene trees investigated. Height of line shows the probability that dN/dS is greater than 1 for each site along the length (x-axis) of the first exon. Grey horizontal bands show the 95% probability range. Light grey and missing plots are where selected models did not resolve a Dn/Ds parameter > 1 within the first 10

rounds rounds; light grey plots show results of 3 of these models after 200 rounds and selection model was not better than the neutral null model.
