# Supplementary material for: Sea star populations diverge by positive selection at a sperm-egg compatibility locus
Source: Ecol Evol. 2013 Feb 6;3(3):640–54. doi: 10.1002/ece3.487 (PMC3605852; doi:10.1002/ece3.487)
Supplement: Supplementary file 3 [file ece30003-0640-SD2.docx]

>BA33-B

GCACGACGTGCCGCAGAGGAAAAACCTCAAAAGGGAAAGAAGGGACGAAAAGGAAAGAAACGACGAAAGGGAAAGAAGGGACGAAAAGGAAAGAAGGGACGAAAAGGAAAGAAACGACGAAAGGGAAAGAAAAGACGAAAGGGAAAGAAAGGACGAAAGGGAAAGAAAGGACGAAAGGGAAAGAAAGGACGAAAGGGAAAGAAAGGACGAAAGGGAAAGAAAGGACGAAAGGGAAAGAAAGGACGAAAGGGAAAGAAAGGA------------------AAACCAATCCAACCTGAAGAAACACCAGCCATTCCGACGGAGATAAAAGCTGCAGAAATAGAAAAGGAACCAAAAACAGAAGTGGTTGTGGAACCAGTTATTCCGGAAGCAGATATTGCGGAGACCGAAATACAACAAATTAAAGCAGAAGTAGAACCAGTTGAAGCAAAGCCAGAAATAGTTATGGAACCAGATGTGGAACCAGTTATTCCAGAAACGGAACTTACAGAGACCGGAAAAGAAGCGAAAGTAGAACAAGTTAAACCAGTGGAAGGGAAGCTAAACTTA------------------------------------------------------------------------------------------------------------------------------------------------------------------------------------------------------------------------------------------------------------------------------------------------------------------------------------------------------------------------------------------------------------------------------------------------------------------------------GGAAAAGGAAAGGGCAAAGGAAAGAAGGTTAGAAAGGGAAAGAAAGGACGAAAGGGAAAGAAAGGACGAAAGGGAAAGAAAGGACGGAAAGGAAAGAAGGGGCGAAAAGGAAAGAAAAGAAGAAAAGGAAAAAAAGGACGAAAGGGAAAGAAAGGACGAAAGGGAAAGAAAGGACGAAAGGGCAAGAAAGGACGAAAGGGAAAGAAAGGACGAAAGGGAAAGAAAGGACGAAAGGGAAAGAAAGGACGAAGGGGAAAGAAAGGA------------------CGAAAGGGCAAGAAAGGAAATCCAATCAAAACTGAAGAAACACCAGCCATTCTGACGGAGATAAAAGCTGCAGAATTAGAAAAGGAACCAAAAACAGAAATAGTTATGAAACCAGTTATTCCCGAAACGGAACTAACAGAGACCGGAAAAGAAGCAGAAGTAGAACAAGTTAAACCAGTGGAAAGGAAGCTAAAAATAGGAAAAGGAAAGGGCAAAGGAAAGAAGGTTAGAAAGGGAAAGAAAGGACGAAAGGGAAAGAAAGGACGAAAGGGAAAGAAGGGACGGAAAGGAAAGAAGGGGCGAAAAGGAAAGAAAAGAAGAAAAGGAAAGAAAAGACGAAAAGGAAAGAAACGACGAAAGGGAAAGAAAGGACGAAAGGGAAAAAAAGGACGAAAGGGAAAGAAAGGACGAAAGGGAAAAAAAGGACGAAAGGGAAAAAAAGGACGAAAGGGAAAGAAAGGACGAAAGGGAAAGAAAGGCAAACCAATCCAAACTGAAGAAACACCGGCCATTCCGACGGAGATAAAAGCTGCAGAAATAGAAAAGGAACCAAAAACAGAAGTGGTTGTGGAACCACTTATTCAGGAAAAAGATGTTGCGGAGACCGAAATACAACCCATCGAAGCAGAAGTAGAACCAGTTGAACCAAAGACAGAAATAGTTATGGAACCAGTTATTCCGGAAGCAGATATTGCGGAGACCGAAATACAACCCATCAAAGCAGAAGAAGAACCAGTT------------------------------------------------------------------------------------------------------------GAACCAAAGCCAGAAATAGTTATGGAACCAGATGAGGAACCAGTTATTCCGGAAGCAGATATTGCGGAGACCGAAATACAACCCATCAAAGCAGAAGAAGAACCAGTTGAACCAAAGCCAGAAATAGTTATGGAACCAGATGAGGAACCAGTTATTCCGGAAGCAGATATTGCGGAGACCGAAATACAACCCATCAAAGCAGAAGAAGAACCAGTTGAACCAAAGCCAGAAATAGTTATGGAACCAGATGAGGAACCAGTTATTCCGGAAGCAGATATTGCGGAGACCGAAATACAACCCATCAGAGCAGAAGAAGAACCAGTTGAACCAAAGCCAGAAATAGTTATGGAACCAGATGAGGAACCAGTCATTCCAGAAACAGAACTAACAGAGACAGACAAACAACCAATCGAAGCAGAAGTAGAACCAGTTGAACCAAAAACAGAAGTGGTTGTGGAACCAGTTATTCCGGAAGCAGATATTGCGGAGACCGAAATACAACCCATCGAAGCAGAAGTAGAACCAGTTGAACCAAAGCCAGAAATAGTTATGGAACCAGATGTGGAACCAGTAATTCCAGAAACAGATCTTACAGAGACCGAAAAACAACCAATCGAAGCAGAAGTAGAACCAGTTGAACCGAAGACAGAGATAGTCGAACCAGAAACAGAGCCAGTCGAAGAAGCCGAGGAAGAAGCGGTTGAAGCACCCGTTATGGAACCAGTTATACCTGAGATAGAACCAAAGACAGAACCGGAAGTTGAAGCCGAGGAAAAAGAAGTCGAAACAAACGTTGAACCAGAACTACTGGAGACAGGAAAACCGATTGAAATTGGCCAAGCAACTGAAATCGAGGAGCCACCAGAAAAACAACCGGTGGAATCGGAGTCACAACCAGCGGAAGTTGAGATGGAACAAATTGAAACAAAGGAAGAACAAACTGAACCAAAGGAAGAACTAGCAGGAATTGATGAGAAATGTAAGTTTTAGTAACAATTATTTATTTTATGATTCCTTTTGAGCTGTTTCGGTATGCTTCGATGCAAGTCAAATTTGTGGATGTTTTAAATCCATCATTCAATTATGTTTGCAATGTAGTCATGAAGTTTGTTCGTTTGTTCAACACAGATATGGGACATATACGCATGAGCAGACCTTTTTTGTGCACAGATCTTCGTTCAGGTTAATCGTGCATGGCAACAAAAATTGGTTCATTCCGTTTTATGTCGTGCAGTCTTATTTTGCTTGAATTAAGCATTGCTGTAAAGTTCAAGTTAAAGGTACAGTCCATCATTTGTAATTTGTGCATTTTGTTCGTTTGAAGAAACAAACATGCTCAAAATCTAAAGAAGGCTGCTGAACAACTAACCT---------------------------------------------------------------------------------------------------------------------------------------------------------------------------------------------------------------------------------------------------------------------------------------GATGAAGTTTCAGCTCAGCTTCAGCTGTATAAATAATTTCGAGAAAAACAAAATTCTAGGATCTCCATTTCAAATCAAAAGTTGCTGAATCTTGTTTTGAATTGGGCCTGAATTCAGCGTCCGAGGCACGTTCTCATCACTGAGCCGATTGCAGCTACGCGATGTCGCGCACGAATTAAAGCAATCGAGGGCGCGTGTGCGTTGTGTCAACGCTTGCGCGCGCTTCGCCAAATAAAAAAAAAAGAACTAAACGGAAATTTCTTTAGACGCATGTCTTTAGACGCACCGTTGGAAAGGCCTACTTTCGTTGCTTTCTTCAAAATGACAGCGTGCCAGAAAGAAAAATCTCTTATCTTTCAACGAAGTATGTTTTTTAACAATAGGCCTAATTACGTCAGTTACTTTTTGGATAGTTGCAAATAATGATGGACTGTAGCTTTAAGAAATTTGTGGAACTCAAGTTGTGCTTGGCAAGTTTTACTTTAGAATTCGAAAATGCATCGACACCAAAAAAAAGTGTAATTTTGGTCCTATAGTTGAAGTGTCAAACTAGAACAAATTTAATGATAAATTAAATAAATTTAAATAATTTATTCCTACCTTGCTTTGACAGTGATGAAGGAGCTGCGTGACCTTTTGGAATCTACGAAGATTGACCTTCCTGTTGACATCAATGATCCATACGACCTAGGTCTTCTTCTCAGACATTTACGTCACCATTCAAATCTTCTTGCTCGTATTGGAGACCCCGATGTCAAAAAGGAAGTCCTCAGCGCCATGAATGAAAAC

>BA82-B

GCACGACGTGCCGCAGAGGAAAAACCTCAAAAGGGAAAGAAGGGACGAAAAGGAAAGAAACGACGAAAGGGAAAGAAGGGACGAAAAGGAAAGAAGGGACGAAAAGGAAAGAAACGACGAAAGGGAAAGAAAAGACGAAAGGGAAAGAAAGGACGAAAGGGAAAGAAAGGACGAAAGGGAAAGAAAGGACGAAAGGGAAAGAAAGGACGAAAGGGAAAGAAAGGACGAAAGGGAAAGAAAGGACGAAAGGGAAAGAAAGGA------------------AAACCAATCCAACCTGAAGAAACACCAGCCATTCCGACGGAGATAAAAGCTGCAGAAATAGAAAAGGAACCAAAAACAGAAGTGGTTGTGGAACCAGTTATTCCGGAAGCAGATATTGCGGAGACCGAAATACAACAAATTAAAGCAGAAGTAGAACCAGTTGAAACAAAGCCAGAAATAGTTATGGAACCAGATGTGGAACCAGTTATTCCAGAAACGGAACTTACAGAGACCGGAAAAGAAGCGAAAGTAGAACAAGTTAAACCAGTGGAAGGGAAGCTAAACTTA------------------------------------------------------------------------------------------------------------------------------------------------------------------------------------------------------------------------------------------------------------------------------------------------------------------------------------------------------------------------------------------------------------------------------------------------------------------------------GGAAAAGGAAAGGGCAAAGGAAAGAAGGTTAGAAAGGGAAAGAAAGGACGAAAGGGAAAGAAAGGACGAAAGGGAAAGAAAGGACGGAAAGGAAAGAAGGGGCGAAAAGGAAAGAAAAGAAGAAAAGGAAAAAAAGGACGAAAGGGAAAGAAAGGACGAAAGGGAAAGAAAGGACGAAAGGGCAAGAAAGGACGAAAGGGAAAGAAAGGACGAAAGGGAAAGAAAGGACGAAAGGGAAAGAAAGGACGAAGGGGAAAGAAAGGA------------------CGAAAGGGCAAGAAAGGAAATCCAATCAAAACTGAAGAAACACCAGCCATTCTGACGGAGATAAAAGCTGCAGAATTAGAAAAGGAACCAAAAACAGAAATAGTTATGAAACCAGTTATTCCCGAAACGGAACTAACAGAGACCGGAAAAGAAGCAGAAGTAGAACAAGTTAAACCAGTGGAAAGGAAGCTAAAAATAGGAAAAGGAAAGGGCAAAGGAAAGAAGGTTAGAAAGGGAAAGAAAGGACGAAAGGGAAAGAAAGGACGAAAGGGAAAGAAAGGACGGAAAGGAAAGAAGGGGCGAAAAGGAAAGAAAAGAAGAAAAGGAAAGAAAAGACGAAAAGGAAAGAAACGACGAAAGGGAAAGAAAGGACGAAAGGGAAAAAAAGGACGAAAGGGAAAGAAAGGACGAAAGGGAAAAAAAGGACGAAAGGGAAAAAAAGGACGAAAGGGAAAGAAAGGACGAAAGGGAAAGAAAGGCAAACCAATCCAAACTGAAGAAACACCAGCCATTCCGACGGAGATAAAAGCTGCAGAAATAGAAAAGGAACCAAAAACAGAAGTGGTTGTGGAACCACTTATTCAGGAAAAAGATGTTGCGGAGACCGAAATACAACCCATCGAAGCAGAAGTAGAACCAGTTGAACCAAAGACAGAAATAGTTATGGAACCAGTTATTCCGGAAGCAGATATTGCGGAGACCGAAATACAACCCATCAAAGCAGAAGAAGAACCAGTT------------------------------------------------------------------------------------------------------------GAACCAAAGCCAGAAATAGTTATGGAACCAGATGAGGAACCAGTTATTCCGGAAGCAGATATTGCGGAGACCGAAATACAACCCATCAAAGCAGAAGAAGAACCAGTTGAACCAAAGCCAGAAATAGTTATGGAACCAGATGAGGAACCAGTTATTCCGGAAGCAGATATTGCGGAGACCGAAATACAACCCATCAAAGCAGAAGAAGAACCAGTTGAACCAAAGCCAGAAATAGTTATGGAACCAGATGAGGAACCAGTTATTCCGGAAGCAGATATTGCGGAGACCGAAATACAACCCATCAAAGCAGAAGAAGAACCAGTTGAACCAAAGCCAGAAATAGTTATGGAACCAGATGAGGAACCAGTTATTCCAGAAACAGAACTAACAGAGACAGACAAACAACCAATCGAAGCAGAAGTAGAACCAGTTGAACCAAAAACAGAAGTGGTTGTGGAACCAGTTATTCCGGAAGCAGATATTGCGGAGACCGAAATACAACCCATCGAAGCAGAAGTAGAACCAGTTGAACCAAAGCCAGAAATAGTTATGGAACCAGATGTGGAACCAGTAATTCCAGAAACAGATCTTACAGAGACCGAAAAACAACCAATCGAAGCAGAAGTAGAACCAGTTGAACCGAAGACAGAGATAGTCGAACCAGAAACAGAGCCAGTCGAAGAAGCCGAGGAAGAAGCGGTTGAAGCACCCGTTATGGAACCAGTTATACCTGAGATAGAACCAAAGACAGAACCGGAAGTTGAAGCCGAGGAAAAAGAAGTCGAAACAAACGTTGAACCAGAACTACTGGAGACAGGAAAACCGATTGAAATTGGCCAAGCAACTGAAATCGAGGAGCCACCAGAAAAACAACCGGTGGAATCGGAGTCACAACCAGCGGAAGTTGAGATGGAACAAATTGAAACAAAGGAAGAACAAACTGAACCAAAGGAAGAACTAGCAGGAATTGATGAGAAATGTAAGTTTTAGTAACAATTATTTATTTTATGATTCCTTTTGAGCTGTTTCGGTATGCTTCGATGCAAGTCAAATTTGTGGATGTTTTAAATCCATCATTCAATTATGTTTGCAATGTAGTCATGAAGTTTGTTCGTTTGTTCAACACAGATATGGGACATATACGCATGAGCAGACCTTTTTTGTGCACAGATCTTCGTTCAGGTTAATCGTGCATGGCAACAAAAATTGGTTCATTCCGTTTTATGTCGTGCAGTCTTATTTTGCTTGAATTAAGCATTGCTGTAAAGTTCAAGTTAAAGGTACAGTCCATCATTTGTAATTTGTGCATTTTGTTCGTTTGAAGAAACAAACATGCTCAAAATCTAAAGAAGGCTGCTGAACAACTAACCT---------------------------------------------------------------------------------------------------------------------------------------------------------------------------------------------------------------------------------------------------------------------------------------GATGAAGTTTCAGCTCAGCTTCAGCTGTATAAATTATTTCGAGAAAAACAAAATTCTAGGATCTCCATTTCAAATCAAAAGTTGCTGAATCTTGTTTTGAATTGGGCCTGAATTCAGCGTCCGAGGCACGTTCTCATCACTGAGCCGATTGCAGCTACGCGATGTCGCGCACGAATTAAAGCAATCGAGGGCGCGTGTGCGTTGTGTCAACGCTTGCGCGCGCTTCGCCAAAT-AAAAAAAAAGAACTAAACGGAAATTTCTTTAGACGCATGTCTTTAGACGCACCGTTGGAAAGGCCTACTTTCGTTGCTTTCTTCAAAATGACAGCATGCCAGAAAGAAAAATCTCTTATCTTTCAACGAAGTATGTTTTTTAACAATAGGCCTAATTACGTCAGTTACTTTTTGGATAGTTGCAAATAATGATGGACTGTAGCTTTAAGAAATTTGTGGAACTCAAGTTGTGCTTGGCAAGTTTTACTTTAGAATTCGAAAATGCATCGACACCAAAAAAAAGTGTAATTTTGGTCCTATAGTTGAAGTGTCAAACTAGAACAAATTTAATGATAAATTAAATAAATTTAAATAATTTATTCCTACCTTGCTTTGACAGTGATGAAGGAGCTGCGTGACCTTTTGGAATCTACGAAGATTGACCTTCCTGTTGACATCAATGATCCATACGACCTAGGTCTTCTTCTCAGACATTTACGTCACCATTCAAATCTTCTTGCTCGTATTGGAGACCCCGATGTCAAAAAGGAAGTCCTCAGCGCCATGAATGAAAAC

>BA76-A

GCACGACGTGCCGCAGAGGAAAAACCTCAAAAGGGAAAGAAGGGACGAAAAGGAAAGAAACGACGAAAGGGAAAGAAGGGACGAAAAGGAAAGAAGGGACGAAAAGGAAAGAAACGACGAAAGGGAAAGAAAAGACGAAAGGGAAAGAAAGGACGAAAGGGAAAGAAAGGACGAAAGGGAAAGAAAGGACGAAAGGGAAAGAAAGGACGAAAGGGAAAGAAAGGACGAAAGGGAAAGAAAGGACGAAAGGGAAAGAAAGGACGAAAGGGAAAGAAAGGAAAACCAATCCAACCTGAAGAAACACCAGCCATTCCGACGGAGATAAAAGCTGCAGAAATAGAAAAGGAACCAAAAACAGAAGTGGTTGTGGAACCAGTTATTCCGGAAGCAGATATTGCGGAGACCGAAATACAACAAATTAAAGCAGAAGTAGAACCAGTTGAAACAAAGCCAGAAATAGTTATGGAACCAGATGTGGAACCAGTTATTCCAGAAACGGAACTTACAGAGACCGGAAAAGAAGCGAAAGTAGAACAAGTTAAACCAGTGGAAGGGAAGCTAAACTTA------------------------------------------------------------------------------------------------------------------------------------------------------------------------------------------------------------------------------------------------------------------------------------------------------------------------------------------------------------------------------------------------------------------------------------------------------------------------------GGAAAAGGAAAGGGCAAAGGAAAGAAGGTTAGAAAGGGAAAGAAAGGACGAAAGGGAAAGAAAGGACGAAAGGGAAAGAAAGGACGGAAAGGAAAGAAGGGGCGAAAAGGAAAGAAAAGAAGAAAAGGAAAAAAAGGACGAAAGGGAAAGAAAGGACGAAAGGGAAAGAAAGGACGAAAGGGCAAGAAAGGACGAAAGGGAAAGAAAGGACGAAAGGGAAAGAAAGGACGAAAGGGAAAGAAAGGACGAAGGGGAAAGAAAGGA------------------CGAAAGGGCAAGAAAGGAAATCCAATCAAAACTGAAGAAACACCAGCCATTCTGACGGAGATAAAAGCTGCAGAATTAGAAAAGGAACCAAAAACAGAAATAGTTATGAAACCAGTTATTCCCGAAACGGAACTAACAGAGACCGGAAAAGAAGCAGAAGTAGAACAAGTTAAACCAGTGGAAAGGAAGCTAAAAATAGGAAAAGGAAAGGGCAAAGGAAAGAAGGTTAGAAAGGGAAAGAAAGGACGAAAGGGAAAGAAAGGACGAAAGGGAAAGAAAGGACGGAAAGGAAAGAAGGGGCGAAAAGGAAAGAAAAGAAGAAAAGGAAAGAAAAGACGAAAAGGAAAGAAACGACGAAAGGGAAAGAAAGGACGAAAGGGAAAAAAAGGACGAAAGGGAAAGAAAGGACGAAAGGGAAAAAAAGGACGAAAGGGAAAAAAAGGACGAAAGGGAAAGAAAGGACGAAAGGGAAAGAAAGGCAAACCAATCCAAACTGAAGAAACACCAGCCATTCCGACGGAGATAAAAGCTGCAGAAATAGAAAAGGAACCAAAAACAGAAGTGGTTGTGGAACCACTTATTCAGGAAAAAGATGTTGCGGAGACCGAAATACAACCCATCGAAGCAGAAGTAGAACCAGTTGAACCAAAGACAGAAATAGTTATGGAACCAGTTATTCCGGAAGCAGATATTGCGGAGACCGAAATACAACCCATCAAAGCAGAAGAAGAACCAGTT------------------------------------------------------------------------------------------------------------------------------------------------------------------------------------------------------------------------GAACCAAAGCCAGAAATAGTTATGGAACCAGATGAGGAACCAGTTATTCCGGAAGCAGATATTGCGGAGACCGAAATACAACCCATCAAAGCAGAAGAAGAACCAGTTGAACCAAAGCCAGAAATAGTTATGGAACCAGATGAGGAACCAGTTATTCCGGAAGCAGATATTGCGGAGACCGAAATACAACCCATCAGAGCAGAAGAAGAACCAGTTGAACCAAAGCCAGAAATAGTTATGGAACCAGATGAGGAACCAGTCATTCCAGAAACAGAACTAACAGAGACAGACAAACAACCAATCGAAGCAGAAGTAGAACCAGTTGAACCAAAAACAGAAGTGGTTGTGGAACCAGTTATTCCGGAAGCAGATATTGCGGAGACCGAAATACAACCCATCGAAGCAGAAGTAGAACCAGTTGAACCAAAGCCAGAAATAGTTATGGAACCAGATGTGGAACCAGTAATTCCAGAAACAGATCTTACAGAGACCGAAAAACAACCAATCGAAGCAGAAGTAGAACCAGTTGAACCGAAGACAGAGATAGTCGAACCAGAAACAGAGCCAGTCGAAGAAGCCGAGGAAGAAGCGGTTGAAGCACCCGTTATGGAACCAGTTATACCTGAGATAGAACCAAAGACAGAACCGGAAGTTGAAGCCGAGGAAAAAGAAGTCGAAACAAACGTTGAACCAGAACTACTGGAGACAGGAAAACCGATTGAAATTGGCCAAGCAACTGAAATCGAGGAGCCACCAGAAAAACAACCGGTGGAATCGGAGTCACAACCAGCGGAAGTTGAGATGGAACAAATTGAAACAAAGGAAGAACAAACTGAACCAAAGGAAGAACTAGCAGGAATTGATGAGAAATGTAAGTTTTAGTAACAATTATTTATTTTATGATTCCTTTTGAGCTGTTTCGGTATGCTTCGATGCAAGCCAAATTTGTGGATGTTTTAAATCCATCATTCAATTATGTTTGCAATGTAGTCATGAAGTTTGTTCGTTTGTTCAACACAGATATGGGACATATACGCATGAGCAGACCTTTTTTGTGCACAGATCTTCGTTCAGGTTAATCGTGCATGGCAACAAAAATTGGTTCATTCTGTTTTATGTCGTGCAGTCTTATTTTGCTTGAATTAAGCATTGCTGTAAAGTTCAAGTTAAAGGTACAGTCCATCATTTGTAATTTGTGCATTTTGTTCGTTTGAAGAAACAAACATGCTCAAAATCTAAAGAAGGCTGCTGAACAACTAACCT---------------------------------------------------------------------------------------------------------------------------------------------------------------------------------------------------------------------------------------------------------------------------------------GATGAAGTTTCAGCTCAGCTTCAGCTGTATAAATTATTTCGAGAAAAACAAAATTCTAGGATCTCCATTTCAAATCAAAAGTTGCTGAATCTTGTTTTGAATTGGGCCTGAATTCAGCGTCCGAGGCACGTTCTCATCACTGAGCCGATTGCAGCTACGCGATGTCGCGCACGAATTAAAGCAATCGAGGGCGCGTGTGCGTTGTGTCAACGCTTGCGCGCGCTTCGCCAAATAAAAAAAAAAGAACTAAACGGAAATTTCTTTAGACGCATGTCTTTAGACGCACCGTTGGAAAGGCCTACTTTCGTTGCTTTCTTCAAAATGACAGCATGCCAGAAAGAAAAATCTCTTATCTTTCAACGAAGTATGTTTTTTAACAATAGGCCTAATTACGTCAGTTACTTTTTGGATAGTTGCAAATAATGATGGACTGTAGCTTTAAGAAATTTGTGGAACTCAAGTTGTGCTTGGCAAGTTTTACTTTAGAATTCGAAAATGCATCGACACCAAAAAAAAGTGTAATTTTGGTCCTATAGTTGAAGTGTCAAACTAGAACAAATTTAATGATAAATTAAATAAATTTAAATAATTTATTCCTACCTTGCTTTGACAGTGATGAAGGAGCTGCGTGACCTTTTGGAATCTACGAAGATTGACCTTCCTGTTGACATCAATGATCCATACGACCTAGGTCTTCTTCTCAGACATTTACGTCACCATTCAAATCTTCTTGCTCGTATTGGAGACCCCGATGTCAAAAAGGAAGTCCTCAGCGCCATGAATGAAAAC

>BA72-B

GCACGACGTGCCGCAGAGGAAAAACCTCAAAAGGGAAAGAAGGGACGAAAAGGAAAGAAACGACGAAAGGGAAAGAAGGGACGAAAAGGAAAGAAGGGACGAAAAGGAAAGAAACGACGAAAGGGAAAGAAAAGACGAAAGGGAAAGAAAGGACGAAAGGGAAAGAAAGGACGAAAGGGAAAGAAAGGACGAAAGGGAAAGAAAGGACGAAAGGGAAAGAAAGGACGAAAGGGAAAGAAAGGACGAAAGGGAAAGAAAGGACGAAAGGGAAAGAAAGGAAAACCAATCCAACCTGAAGAAACACCAGCCATTCCGACGGAGATAAAAGCTGCAGAAATAGAAAAGGAACCAAAAACAGAAGTGGTTGTGGAACCAGTTATTCCGGAAGCAGATATTGCGGAGACCGAAATACAACAAATTAAAGCAGAAGTAGAACCAGTTGAAACAAAGCCAGAAATAGTTATGGAACCAGATGTGGAACCAGTTATTCCAGAAACGGAACTTACAGAGACCGGAAAAGAAGCGAAAGTAGAACAAGTTAAACCAGTGGAAGGGAAGCTAAACTTA------------------------------------------------------------------------------------------------------------------------------------------------------------------------------------------------------------------------------------------------------------------------------------------------------------------------------------------------------------------------------------------------------------------------------------------------------------------------------GGAAAAGGAAAGGGCAAAGGAAAGAAGGTTAGAAAGGGAAAGAAAGGACGAAAGGGAAAGAAAGGACGAAAGGGAAAGAAAGGACGGAAAGGAAAGAAGGGGCGAAAAGGAAAGAAAAGAAGAAAAGGAAAAAAAGGACGAAAGGGAAAGAAAGGACGAAAGGGAAAGAAAGGACGAAAGGGCAAGAAAGGACGAAAGGGAAAGAAAGGACGAAAGGGAAAGAAAGGACGAAAGGGAAAGAAAGGACGAAGGGGAAAGAAAGGA------------------CGAAAGGGCAAGAAAGGAAATCCAATCAAAACTGAAGAAACACCAGCCATTCTGACGGAGATAAAAGCTGCAGAATTAGAAAAGGAACCAAAAACAGAAATAGTTATGAAACCAGTTATTCCCGAAACGGAACTAACAGAGACCGGAAAAGAAGCAGAAGTAGAACAAGTTAAACCAGTGGAAAGGAAGCTAAAAATAGGAAAAGGAAAGGGCAAAGGAAAGAAGGTTAGAAAGGGAAAGAAAGGACGAAAGGGAAAGAAAGGACGAAAGGGAAAGAAAGGACGGAAAGGAAAGAAGGGGCGAAAAGGAAAGAAAAGAAGAAAAGGAAAGAAAAGACGAAAAGGAAAGAAACGACGAAAGGGAAAGAAAGGACGAAAGGGAAAAAAAGGACGAAAGGGAAAGAAAGGACGAAAGGGAAAAAAAGGACGAAAGGGAAAAAAAGGACGAAAGGGAAAGAAAGGACGAAAGGGAAAGAAAGGCAAACCAATCCAAACTGAAGAAACACCAGCCATTCCGACGGAGATAAAAGCTGCAGAAATAGAAAAGGAACCAAAAACAGAAGTGGTTGTGGAACCACTTATTCAGGAAAAAGATGTTGCGGAGACCGAAATACAACCCATCGAAGCAGAAGTAGAACCAGTTGAACCAAAGACAGAAATAGTTATGGAACCAGTTATTCCGGAAGCAGATATTGCGGAGACCGAAATACAACCCATCAAAGCAGAAGAAGAACCAGTT------------------------------------------------------------------------------------------------------------GAACCAAAGCCAGAAATAGTTATGGAACCAGATGAGGAACCAGTTATTCCGGAAGCAGATATTGCGGAGACCGAAATACAACCCATCAAAGCAGAAGAAGAACCAGTTGAACCAAAGCCAGAAATAGTTATGGAACCAGATGAGGAACCAGTTATTCCGGAAGCAGATATTGCGGAGACCGAAATACAACCCATCAAAGCAGAAGAAGAACCAGTTGAACCAAAGCCAGAAATAGTTATGGAACCAGATGAGGAACCAGTTATTCCGGAAGCAGATATTGCGGAGACCGAAATACAACCCATCAGAGCAGAAGAAGAACCAGTTGAACCAAAGCCAGAAATAGTTATGGAACCAGATGAGGAACCAGTCATTCCAGAAACAGAACTAACAGAGACAGACAAACAACCAATCGAAGCAGAAGTAGAACCAGTTGAACCAAAAACAGAAGTGGTTGTGGAACCAGTTATTCCGGAAGCAGATATTGCGGAGACCGAAATACAACCCATCGAAGCAGAAGTAGAACCAGTTGAACCAAAGCCAGAAATAGTTATGGAACCAGATGTGGAACCAGTAATTCCAGAAACAGATCTTACAGAGACCGAAAAACAACCAATCGAAGCAGAAGTAGAACCAGTTGAACCGAAGACAGAGATAGTCGAACCAGAAACAGAGCCAGTCGAAGAAGCCGAGGAAGAAGCGGTTGAAGCACCCGTTATGGAACCAGTTATACCTGAGATAGAACCAAAGACAGAACCGGAAGTTGAAGCCGAGGAAAAAGAAGTCGAAACAAACGTTGAACCAGAACTACTGGAGACAGGAAAACCGATTGAAATTGGCCAAGCAACTGAAATCGAGGAGCCACCAGAAAAACAACCGGTGGAATCGGAGTCACAACCAGCGGAAGTTGAGATGGAACAAATTGAAACAAAGGAAGAACAAACTGAACCAAAGGAAGAACTAGCAGGAATTGATGAGAAATGTAAGTTTTAGTAACAATTATTTATTTTATGATTCCTTTTGAGCTGTTTCGGTATGCTTCGATGCAAGTCAAATTTGTGGATGTTTTAAATCCATCATTCAATTATGTTTGCAATGTAGTCATGAAGTTTGTTCGTTTGTTCAACACAGATATGGGACATATACGCATGAGCAGACCTTTTTTGTGCACAGATCTTCGTTCAGGTTAATCGTGCATGGCAACAAAAATTGGTTCATTCTGTTTTATGTCGTGCAGTCTTATTTTGCTTGAATTAAGCATTGCTGTAAAGTTCAAGTTAAAGGTACAGTCCATCATTTGTAATTTGTGCATTTTGTTCGTTTGAAGAAACAAACATGCTCAAAATCTAAAGAAGGCTGCTGAACAACTAACCT---------------------------------------------------------------------------------------------------------------------------------------------------------------------------------------------------------------------------------------------------------------------------------------GATGAAGTTTCAGCTCAGCTTCAGCTGTATAAATTATTTCGAGAAAAACAAAATTCTAGGATCTCCATTTCAAATCAAAAGTTGCTGAATCTTGTTTTGAATTGGGCCTGAATTCAGCGTCCGAGGCACGTTCTCATCACTGAGCCGATTGCAGCTACGCGATGTCGCGCACGAATTAAAGCAATCGAGGGCGCGTGCGCGTTGTGTCAACGCTTGCGCGCGCTTCGCCAAATAAAAAAAAAAGAACTAAACGGAAATTTCTTTAGACGCATGTCTTTAGACGCACCGTTGGAAAGGCCTACTTTCGTTGCTTTCTTCAAAATGACAGCATGCCAGAAAGAAAAATCTCTTATCTTTCAACGAAGTATGTTTTTTAACAATAGGCCTAATTACGTCAGTTACTTTTTGGATAGTTGCAAATAATGATGGACTGTAGCTTTAAGAAATTTGTGGAACTCAAGTTGTGCTTGGCAAGTTTTACTTTAGAATTCGAAAATGCATCGACACCAAAAAAAAGTGTAATTTTGGTCCTATAGTTGAAGTGTCAAACTAGAACAAATTTAATGATAAATTAAATAAATTTAAATAATTTATTCCTACCTTGCTTTGACAGTGATGAAGGAGCTGCGTGACCTTTTGGAATCTACGAAGATTGACCTTCCTGTTGACATCAATGATCCATACGACCTAGGTCTTCTTCTCAGACATTTACGTCACCATTCAAATCTTCTTGCTCGTATTGGAGACCCCGATGTCAAAAAGGAAGTCCTCAGCGCCATGAATGAAAAC

>BA74-A

GCACGACGTGCCGCAGAGGAAAAACCTCAAAAGGGAAAGAAGGGACGAAAAGGAAAGAAACGACGAAAGGGAAAGAAGGGACGAAAAGGAAAGAAGGGACGAAAAGGAAAGAAACGACGAAAGGGAAAGAAAAGACGAAAGGGAAAGAAAGGACGAAAGGGAAAGAAAGGACGAAAGGGAAAGAAAGGACGAAAGGGAAAGAAAGGACGAAAGGGAAAGAAAGGACGAAAGGGAAAGAAAGGACGAAAGGGAAAGAAAGGACGAAAGGGAAAGAAAGGAAAACCAATCCAACCTGAAGAAACACCAGCCATTCCGACGGAGATAAAAGCTGCAGAAATAGAAAAGGAACCAAAAACAGAAATAGTTATGAAACCAGTTATTCCGGAAGCAGATATTGCGGAGACCGAAATACAACAAATTAAAGCAGAAGTAGAACCAGTTGAAACAAAGCCAGAAATAGTTATGGAACCAGATGTGGAACCAGTTATTCCAGAAACGGAACTTACAGAGACCGGAAAAGAAGCGAAAGTAGAACAAGTTAAACCAGTGGAAGGGAAGCTAAACTTA------------------------------------------------------------------------------------------------------------------------------------------------------------------------------------------------------------------------------------------------------------------------------------------------------------------------------------------------------------------------------------------------------------------------------------------------------------------------------GGAAAAGGAAAGGGCAAAGGAAAGAAGGTTAGAAAGGGAAAGAAAGGACGAAAGGGAAAGAAAGGACGAAAGGG------------------------------------------------------------------------------------------------------------CAAGAAAGGACGAAAGGGAAAGAAAGGACGAAAGGGAAAGAAAGGACGAAAGGGAAAGAAAGGACGAAAGGGAAAGAAAGGA------------------CGAAAGGGCAAGAAAGGAAATCCAATCAAAACTGAAGAAACACCAGCCATTCTGACGGAGATAAAAGCTGCAGAATTAGAAAAGGAACCAAAAACAGAAATAGTTATGAAACCAGTTATTCCCGAAACGGAACTAACAGAGACCGGAAAAGAAGCAGAAGTAGAACAAGTTAAACCAGTGGAAAGGAAGCTAAAAATAGGAAAAGGAAAGGGCAAAGGAAAGAAGGTTAGAAAGGGAAAGAAAGGACGAAAGGGAAAGAAAGGACGAAAGGGAAAGAAAGGACGGAAAGGAAAGAAGGGGCGAAAAGGAAAGAAAAGAAGAAAAGGAAAGAAAAGACGAAAAGGAAAGAAACGACGAAAGGGAAAGAAAGGACGAAAGGGAAAAAAAGGACGAAAGGGAAAGAAAGGACGAAAGGGAAAAAAAGGACGAAAGGGAAAAAAAGGACGAAAGGGAAAGAAAGGACGAAAGGGAAAGAAAGGCAAACCAATCCAAACTGAAGAAACACCAGCCATTCCGACGGAGATAAAAGCTGCAGAAATAGAAAAGGAACCAAAAACAGAAGTGGTTGTGGAACCACTTATTCAGGAAAAAGATGTTGCGGAGACCGAAATACAACCCATCGAAGCAGAAGTAGAACCAGTTGAACCAAAGACAGAAATAGTTATGGAACCAGTTATTCCGGAAGCAGATATTGCGGAGACCGAAATACAACCCATCAAAGCAGAAGAAGAANCAGTT------------------------------------------------------------------------------------------------------------GAACCAAAGCCAGAAATAGTTATGGAACCAGATGAGGAACCAGTTATTCCGGAAGCAGATATTGCGGAGACCGAAATACAACCCATCAAAGCAGAAGAAGAACCAGTTGAACCAAAGCCAGAAATAGTTATGGAACCAGATGAGGAACCAGTTATTCCGGAAGCAGATATTGCGGAGACCGAAATACAACCCATCAAAGCAGAAGAAGAACCAGTTGAACCAAAGCCAGAAATAGTTATGGAACCAGATGAGGAACCAGTTATTCCGGAAGCAGATATTGCGGAGACCGAAATACAACCCATCAGAGCAGAAGAAGAACCAGTTGAACCAAAGCCAGAAATAGTTATGGAACCAGATGAGGAACCAGTCATTCCAGAAACAGAACTAACAGAGACAGACAAACAACCAATCGAAGCAGAAGTAGAACCAGTTGAACCAAAAACAGAAGTGGTTGTGGAACCAGTTATTCCGGAAGCAGATATTGCGGAGACCGAAATACAACCCATCGAAGCAGAAGTAGAACCAGTTGAACCAAAGCCAGAAATAGTTATGGAACCAGATGTGGAACCAGTAATTCCAGAAACAGATCTTACAGAGACCGAAAAACAACCAATCGAAGCAGAAGTAGAACCAGTTGAACCGAAGACAGAGATAGTCGAACCAGAAACAGAGCCAGTCGAAGAAGCCGAGGAAGAAGCGGTTGAAGCACCCGTTATGGAACCAGTTATACCTGAGATAGAACCAAAGACAGAACCGGAAGTTGAAGCCGAGGAAAAAGAAGTCGAAACAAACGTTGAACCAGAACTACTGGAGACAGGAAAACCGATTGAAATTGGCCAAGCAACTGAAATCGAGGAGCCACCAGAAAAACAACCGGTGGAATCGGAGTCACAACCAGCGGAAGTTGAGATGGAACAAATTGAAACAAAGGAAGAACAAACTGAACCAAAGGAAGAACTAGCAGGAATTGATGAGAAATGTAAGTTTTAGTAACAATTATTTATTTTATGATTCCTTTTGAGCTGTTTCGGTATGCTTCGATGCAAGTCAAATTTGTGGATGTTTTAAATCCATCATTCAATTATGTTTGCAATGTAGTCATGAAGTTTGTTCGTTTGTTCAACACAGATATGGGACATATACGCATGAGCAGACCTTTTTTGTGCACAGATCTTCGTTCAGGTTAATCGTGCATGGCAACAAAAATTGGTTCATTCCGTTTTATGTCGTGCAGTCTTATTTTGCTTGAATTAAGCATTGCTGTAAAGTTCAAGTTAAAGGTACATTCCATCATTTGTAATTTGTGCATTTTGTTCGCTTGAAGAAACAAACATGCTCAAAATCTAAAGAAGGCTGCTGAACAACTAACCT---------------------------------------------------------------------------------------------------------------------------------------------------------------------------------------------------------------------------------------------------------------------------------------GATGAAGTTTCAGCTCAGCTTCAGCTGTATAAATTATTTCGAGAAAAACAAAATTCTAGGATCTCCATTTCAAATCAAAAGTTGCTGAATCTTGTTTTGAATTGGGCCTGAATTCAGCGTCCGAGGCACGTTCTCATCACTGAGCCGATTGCAGCTACGCGATGTCGCGCACGAATTAAAGCAATCGAGGGCGCGTGTGCGTTGTGTCAACGCTTGCGCGCGCTTCGCCAAATAAAAAAAAAAGAACTAAACGGAAATTTCTTTAGACGCATGTCTTTAGACGCACCGTTGGAAAGGCCTACTTTCGTTGCTTTCTTCAAAATGACAGCATGCCAGAAAGAAAAATCTCTTATCTTTCAACGAAGTATGTTTTTTAACAATAGGCCTAATTACGTCAGTTACTTTTTGGATAGTTGCAAATAATGATGGACTGTAGCTTTAAGAAATTTGTGGAACTCAAGTTGTGCTTGGCAAGTTTTACTTTAGAATTCGAAAATGCATCGACACCAAAAAAAAGTGTAATTTTGGTCCTATAGTTGAAGTGTCAAACTAGACCAAATTTAATGATAAATTAAATAAATTTAAAT----------------------------------------------------------------------------------------------------------------------------------------------------------------------------------------------------------

>BA84-A

GCACGACGTGCCGCAGAGGAAAAACCTCAAAAGGGAAAGAAGGGACGAAAAGGAAAGAAACGACGAAAGGGAAAGAAGGGACGAAAAGGAAAGAAGGGACGAAAAGGAAAGAAACGACGAAAGGGAAAGAAAAGACGAAAGGGAAAGAAAGGACGAAAGGGAAAGAAAGGACGAAAGGGAAAGAAAGGACGAAAGGGAAAGAAAGGA------------------------------------------------------------------------AAACCAATCCAACCTGAAGAAACACCAGCCATTCCGACGGAGATAAAAGCTGCAGAAATAGAAAAGGAACCAAAAACAGAAGTGGTTGTGGAACCAGTTATTCCGGAAGCAGATATTGCGGAGACCGAAATACAACAAATTAAAGCAGAAGTAGAACCAGTTGAAACAAAGCCAGAAATAGTTATGGAACCAGATGTGGAACCAGTTATTCCAGAAACGGAACTTACAGAGACCGGAAAAGAAGCGAAAGTAGAACAAGTTAAACCAGTCGAAGGGAAGCTAAACTTA------------------------------------------------------------------------------------------------------------------------------------------------------------------------------------------------------------------------------------------------------------------------------------------------------------------------------------------------------------------------------------------------------------------------------------------------------------------------------GGAAAAGGAAAGGGCAAAGGAAAGAAGGTTAGAAAGGGAAAGAAAGGACGAAAGGGAAAGAAAGGACGAAAGGGAAAGAAAGGACGGAAAGGAAAGAAGGGGCGAAAAGGAAAGAAAAGAAGAAAAGGAAAAAAAGGACGAAAGGGAAAGAAAGGACGAAAGGGAAAGAAAGGACGAAAGGGCAAGAAAGGACGAAAGGGAAAGAAAGGACGAAAGGGAAAGAAAGGACGAAAGGGAAAGAAAGGACGAAGGGGAAAGAAAGGA------------------CGAAAGGGCAAGAAAGGAAATCCAATCAAAACTGAAGAAACACCAGCCATTCTGACGGAGATAAAAGCTGCAGAATTAGAAAAGGAACCAAAAACAGAAATAGTTATGAAACCAGTTATTCCCGAAACGGAACTAACAGAGACCGGAAAAGAAGCAGAAGTAGAACAAGTTAAACCAGTGGAAAGGAAGCTAAAAATAGGAAAAGGAAAGGGCAAAGGAAAGAAGGTTAGAAAGGGAAAGAAAGGACGAAAGGGAAAGAAAGGACGAAAGGGAAAGAAAGGACGGAAAGGAAAGAAGGGGCGAAAAGGAAAGAAAAGAAGAAAAGGAAAGAAAAGACGAAAAGGAAAGAAACGACGAAAGGGAAAGAAAGGACGAAAGGGAAAAAAAGGACGAAAGGGAAAGAAAGGACGAAAGGGAAAAAAAGGACGAAAGGGAAAAAAAGGACGAAAGGGAAAGAAAGGACGAAAGGGAAAGAAAGGCAAACCAATCCAAACTGAAGAAACACCAGCCATTCCGACGGAGATAAAAGCTGCAGAAATAGAAAAGGAACCAAAAACAGAAGTGGTTGTGGAACCACTTATTCAGGAAAAAGATGTTGCGGAGACCGAAATACAACCCATCGAAGCAGAAGTAGAACCAGTTGAACCAAAGACAGAAATAGTTATGGAACCAGTTATTCCGGAAGCAGATATTGCGGAGACCGAAATACAACCCATCAAAGCAGAAGAAGAACCAGTT------------------------------------------------------------------------------------------------------------GAACCAAAGCCAGAAATAGTTATGGAACCAGATGAGGAACCAGTTATTCCGGAAGCAGATATTGCGGAGACCGAAATACAACCCATCAAAGCAGAAGAAGAACCAGTTGAACCAAAGCCAGAAATAGTTATGGAACCAGATGAGGAACCAGTTATTCCGGAAGCAGATATTGCGGAGACNGAAATACAACCCATCAAAGCAGAAGAAGAACCAGTTGAACCAAAGCCAGAAATAGTTATGGAACCAGATGAGGAACCAGTTATTCCGGAAGCAGATATTGCGGAGACCGAAATACAACCCATCAGAGCAGAAGAAGAACCAGTTGAACCAAAGCCAGAAATAGTTATGGAACCAGATGAGGAACCAGTCATTCCAGAAACAGAACTAACAGAGACAGACAAACAACCAATCGAAGCAGAAGTAGAACCAGTTGAACCAAAAACAGAAGTGGTTGTGGAACCAGTTATTCCGGAAGCAGATATTGCGGAGACCGAAATACAACCCATCGAAGCAGAAGTAGAACCAGTTGAACCAAAGCCAGAAATAGTTATGGAACCAGATGTGGAACCAGTAATTCCAGAAACAGATCTTACAGAGACCGAAAAACAACCAATCGAAGCAGAAGTAGAACCAGTTGAACCGAAGACAGAGATAGTCGAACCAGAAACAGAGCCAGTCGAAGAAGCCGAGGAAGAAGCGGTTGAAGCACCCGTTATGGAACCAGTTATACCTGAGATAGAACCAAAGACAGAACCGGAAGTTGAAGCCGAGGAAAAAGAAGTCGAAACAAACGTTGAACCAGAACTACTGGAGACAGGAAAACCGATTGAAATTGGCCAAGCAACTGAAATCGAGGAGCCACCAGAAAAACAACCGGTGGAATCGGAGTCACAACCAGCGGAAGTTGAGATGGAACAAATTGAAACAAAGGAAGAACAAACTGAACCAAAGGAAGAACTAGCAGGAATTGATGAGAAATGTAAGTTTTAGTAACAATTATTTATTTTATGATTCCTTTTGAGTTGTTTCGGTATGCTTCGATGCAAGTCAAATTTGTGGATGTTTTAAATCCATCATTCAATTATGTTTGCAATGTAGTCATGAAGTTTGTTCGTTTGTTCAACACAGATATGGGACATATACGCATGAGTAGACCTTTTTTGTGCACAGATCTTCGTTCAGGTTAATCGTGCATGGCAACAAAAATTGGTTCATTCTGTTTTATGTCGTGCAGTCTTATTTTGCTTGAATTAAGCATTGCTGTAAAGTTCAAGTTAAAGGTACAGTCCATCATTTGTAA-TTGTGCATTTTGTTCGTTTGAAGAAACAAACATGCTCAAAATCTAAAGAAGGCTGCTGAACAACTAACCT---------------------------------------------------------------------------------------------------------------------------------------------------------------------------------------------------------------------------------------------------------------------------------------GATGAAGTTTCAGCTCAGCTTCAGCTGTATAAATTATTTCTAGAAAAACAAAATTCTAGGATCTCCATTTCAAATCAAAAGTTGCTGAATCTTGTTTTGAATTGGGCCTGAATTCAGCGTCCGAGGCACGTTCTCATCACTGAGCCGATTGCAGCTACGCGATGTCGCGCACGAATTAAAGCAATCGAGGGCGCGTGTGCGTTGTGTCAACGCTTGCGCGCGCTTCGCCAAATAAAAAAAAAAGAACTAAACGGAAATTTCTTTAGACGCATGTCTTTAGACGCACCGTTGGAAAGGCCTACTTTCGTTGCTTTCTTCAAAATGACAGCATGCCAGAAAGAAAAATCTCTTATCTTTCAACGAAGTATGTTTTTTAACAATAGGCCTAATTACGTCAGTTACTTTTTGGATAGTTGCAAATAATGATGGACTGTAGCTTTAAGAAATTTGTGGAACTCAAGTTGTGCTTGGCAAGTTTTACTTTAGAATTCGAAAATGCATCGACACCAAAAAAAAGTGTAATTTTGGTCCTATAGTTGAAGTGTCAAACTAGAACAAATTTAATGATAAATTAAATAAATTTAAATAATTTATTCCTACCTCGCTTTGACAGTGATGAAGGAGCTACGTGACCTTTTGGAATCTACGAAGATTGACCTTCCTGTTGACATCAATGATCCATACGACCTAGGTCTTCTTCTCAGACATTTACGTCACCATTCAAATCTTCTTGCTCGTATTGGAGACCCCGATGTCAAAAAGGAAGTCCTCAGCGCCATGAATGAAAAC

>BA40-A

GCACGACGTGCCGCAGAGGAAAAACCTCAAAAGGGAAAGAAGGGACGAAAAGGAAAGAAACGACGAAAGGGAAAGAAGGGACGAAAAGGAAAGAAGGGACGAAAAGGAAAGAAACGACGAAAGGGAAAGAAAAGACGAAAGGGAAAGAAAGGACGAAAGGGAAAGAAAGGACGAAAGGGAAAGAAAGGACGAAAGGGAAAGAAAGGACGAAAGGGAAAGAAAGGACGAAAGGGAAAGAAAGGACGAAAGGGAAAGAAAGGACGAAAGGGAAAGAAAGGAAAACCAATCCAACCTGAAGAAACACCAGCCATTCCGACGGAGATAAAAGCTGCAGAAATAGAAAAGGAACCAAAAACAGAAGTGGTTGTGGAACCAGTTATTCCGGAAGCAGATATTGCGGAGACCGAAATACAACAAATTAAAGCAGAAGTAGAACCAGTTGAAACAAAGCCAGAAATAGTTATGGAACCAGATGTGGAACCAGTTATTCCAGAAACGGAACTTACAGAGACCGGAAAAGAAGCGAAAGTAGAACAAGTTAAACCAGTGGAAGGGAAGCTAAACTTA------------------------------------------------------------------------------------------------------------------------------------------------------------------------------------------------------------------------------------------------------------------------------------------------------------------------------------------------------------------------------------------------------------------------------------------------------------------------------GGAAAAGGAAAGGGCAAAGGAAAGAAGGTTAGAAAGGGAAAGAAAGGACGAAAGGGAAAGAAAGGACGAAAGGGAAAGAAAGGACGGAAAGGAAAGAAGGGGCGAAAAGGAAAGAAAAGAAGAAAAGGAAAAAAAGGACGAAAGGGAAAGAAACGACGAAAGGGAAAGAAAGGACGAAAGGGCAAGAAAGGACGAAAGGGAAAGAAAGGACGAAAGGGAAAGAAAGGACGAAAGGGAAAGAAAGGACGAAGGGGAAAGAAAGGA------------------CGAAAGGGCAAGAAAGGAAATCCAATCAAAACTGAAGAAACACCAGCCATTCTGACGGAGATAAAAGCTGCAGAATTAGAAAAGGAACCAAAAACAGAAATAGTTATGAAACCAGTTATTCCCGAAACGGAACTAACAGAGACCGGAAAAGAAGCAGAAGTAGAACAAGTTAAACCAGTGGAAAGGAAGCTAAAAATAGGAAAAGGAAAGGGCAAAGGAAAGAAGGTTAGAAAGGGAAAGAAAGGACGAAAGGGAAAGAAAGGACGAAAGGGAAAGAAAGGACGGAAAGGAAAGAAGGGGCGAAAAGGAAAGAAAAGAAGAAAAGGAAAGAAAAGACGAAAAGGAAAGAAACGACGAAAGGGAAAGAAAGGACGAAAGGGAAAAAAAGGACGAAAGGGAAAGAAAGGACGAAAGGGAAAAAAAGGACGAAAGGGAAAAAAAGGACGAAAGGGAAAGAAAGGACGAAAGGGAAAGAAAGGCAAACCAATCCAAACTGAAGAAACACCAGCCATTCCGACGGAGATAAAAGCTGCAGAAATAGAAAAGGAACCAAAAACAGAAGTGGTTGTGGAACCACTTATTCAGGAAAAAGATGTTGCGGAGACCGAAATACAACCCATCGAAGCAGAAGTAGAACCAGTTGAACCAAAGACAGAAATAGTTATGGAACCAGTTATTCCGGAAGCAGATATTGCGGAGACCGAAATACAACCCATCAAAGCAGAAGAAGAACCAGTT------------------------------------------------------------------------------------------------------------GAACCAAAGCCAGAAATAGTTATGGAACCAGATGAGGAACCAGTTATCGCGGAAGCAGATATTGCGGAGACCGAAATACAACCCATCAAAGCAGAAGAAGAACCAGTTGAACCAAAGCCAGAAATAGTTATGGAACCAGATGAGGAACCAGTTATTCCGGAAGCAGATATTGCGGAGACCGAAATACAACCCATCAAAGCAGAAGAAGAACCAGTTGAACCAAAGCCAGAAATAGTTATGGAACCAGATGAGGAACCAGTTATTCCGGAAGCAGATATTGCGGAGACCGAAATACAACCCATCAGAGCAGAAGAAGAACCAGTTGAACCAAAGCCAGGAATAGTTATGGAACCAGATGAGGAACCAGTCATTCCAGAAACAGAACTAACAGAGACAGACAAACAACCAATCGAAGCAGAAGTAGAACCAGTTGAACCAAAAACAGAAGTGGTTGTGGAACCAGTTATTCCGGAAGCAGATATTGCGGAGACCGAAATACAACCCATCGAAGCAGAAGTAGAACCAGTTGAACCAAAGCCAGAAATAGTTATGGAACCAGATGTGGAACCAGTAATTCCAGAAACAGATCTTACAGAGACCGAAAAACAACCAATCGAAGCAGAAGTAGAACCAGTTGAACCGAAGACAGAGATAGTCGAACCAGAAACAGAGCCAGTCGAAGAAGCCGAGGAAGAAGCGGTTGAAGCACCCGTTATGGAACCAGTTATACCTGAGATAGAACCAGAGACAGAACCGGAAGTTGAAGCCGAGGAAAAAGAAGTCGAAACAAACGTTGAACCAGAACTACTGGAGACAGGAAAACCGATTGAAATTGGCCAAGCAACTGAAATCGAGGAGCCACCAGAAAAACAACCGGTGGAATCGGAGTCACAACCAGCGGAAGTTGAGATGGAACAAATTGAAACAAAGGAAGAACCAACTGAACCAAAGGAAGAACTAGCAGGAATTGATGAGAAATGTAAGTTTTAGTAACAATTATTTATTTTATGATTCCTTTTGAGCTGCTTCGGTATGCTTCGATGCAAGTCAAATTTGTGGATGTTTTAAATCCATCATTCAATTATGTTTGCAATGTAGTCATGAAGTTTGTTCGTTTGTTCAACACAATTATGGGACATATACGCATGAGCAGACCTTTTTTGTGCACAGATCTTCGTTCAGGTTAATCGTGCATGGCAACAAAAATTGGTTCATTCCGTTTTATGTCGTGCAGTCTTATTTTGCTTGAATTAAGCATTGCTGTAAAGTTCAAGTTAAAGGTACAGTCCATCATTTGTAATTTGTGCATTTTGTTCGTTTGAAGAAACAAACATGCTCAAAATCTAAAGAAGGCTGCTGAACAACTAACCT---------------------------------------------------------------------------------------------------------------------------------------------------------------------------------------------------------------------------------------------------------------------------------------GATGAAGTTTCAGCTCAGCTTCAGCTGTATAAATTATTTCGAGAAAAACAAAATTCTAGGATCTCCATTTCAAATAAAAAGTTGCTGAATTTTGTTTTGAATTGGGCCTGAATTCAGCGTCCGAGGCACGTTCTCATCACTGAGTCGATTGCAGCTACGCGATGTCGCGCACGAATTACAGCAATCGAGGGCGCGTGTGCGTTGTGTCAACGCTTGCGCGCGCTTCGCCAAATAAAAAAAAAAGAACTAAACGGAAATTTCTTTAGACGCATGTCTTTAGACGCACCGTTGGAAAGGCCTACTTTCGTTGCTTTCTTCAAAATGACAGCATGCCAGAAAGAAAAATCTCTTATCTTTCAACGAAGTATGTTTTTTAACAATAGGCCTAATTACGTCAGTTACTTTTTGGATAGTTGCAAATAATGATGGACTGTAGCTTTAAGAAATTTGTGGAACTAAAGTTGTGCTTGGCAAGTTTTACTTTAGAATTCGAAAATGCATCGACACCAAAAAAAAGTGTAATTTTGGTCCTATAGTTGAAGTGTCAAACTAGAACAAATTTAATGATAAATTAAATAAATTTAAATAATTTATTCCTACCTTGCTTTGACAGTGATGAAGGAGCTGCGTGACCTTTTGGAATCTACGAAGATTGACCTTCCTGTTGACATCAATGATCCATACGACCTAGGTCTTCTTCTCAGACATTTACGTCACCATTCAAATCTTCTTGCTCGTATTGGAGACCCCGATGTCAAAAAGGAAGTCCTCAGCGCCATGAATGAAAAC

>BA80-B

GCACGACGTGCCGCAGAGGAAAAACCTCAAAAGGGAAAGAAGGGACGAAAAGGAAAGAAACGACGAAAGGGAAAGAAGGGACGAAAAGGAAAGAAGGGACGAAAAGGAAAGAAACGACGAAAGGGAAAGAAAAGACGAAAGGGAAAGAAAGGACGAAAGGGAAAGAAAGGACGAAAGGGAAAGAAAGGACGAAAGGGAAAGAAAGGACGAAAGGGAAAGAAAGGACGAAAGGGAAAGAAAGGACGAAAGGGAAAGAAAGGACGAAAGGGAAAGAAAGGAAAACCAATCCAACCTGAAGAAACACCAGCCATTCCGACGGAGATAAAAGCTGCAGAAATAGAAAAGGAACCAAAAACAGAAGTGGTTGTGGAACCAGTTATTCCGGAAGCAGATATTGCGGAGACCGAAATACAACAAATTAAAGCAGAAGTAGAACCAGTTGAAACAAAGCCAGAAATAGTTATGGAACCAGATGTGGAACCAGTTATTCCAGAAACGGAACTTACAGAGACCGGAAAAGAAGCGAAAGTAGAACAAGTTAAACCAGTGGAAGGGAAGCTAAACTTA------------------------------------------------------------------------------------------------------------------------------------------------------------------------------------------------------------------------------------------------------------------------------------------------------------------------------------------------------------------------------------------------------------------------------------------------------------------------------GGAAAAGGAAAGGGCAAAGGAAAGAAGGTTAGAAAGGGAAAGAAAGGACGAAAGGGAAAGAAAGGACGAAAGGGAAAGAAAGGACGGAAAGGAAAGAAGGGGCGAAAAGGAAAGAAAAGAAGAAAAGGAAAAAAAGGACGAAAGGGAAAGAAAGGACGAAAGGGAAAGAAAGGACGAAAGGGCAAGAAAGGACGAAAGGGAAAGAAAGGACGAAAGGGAAAGAAAGGACGAAAGGGAAAGAAAGGACGAANGGGAAAGAAAGGA------------------CGAAAGGGCAAGAAAGGAAATCCAATCAAAACTGAAGAAACACCAGCCATTCTGACGGAGATAAAAGCTGCAGAATTAGAAAAGGAACCAAAAACAGAAATAGTTATGAAACCAGTTATTCCCGAAACGGAACTAACTGAGACCGGAAAAGAAGCAGAAGTAGAACAAGTTAAACCAGTGGAAAGGAAGCTAAAAATAGGAAAAGGAAAGGGCAAAGGAAAGAAGGTTAGAAAGGGAAAGAAAGGACGAAAGGGAAAGAAAGGACGAAAGGGAAAGAAAGGACGGAAAGGAAAGAAGGGGCGAAAAGGAAAGAAAAGAAGAAAAGGAAAGAAAAGACGAAAAGGAAAGAAACGACGAAAGGGAAAGAAAGGACGAAAGGGAAAAAAAGGACGAAAGGGAAAGAAAGGACGAAAGGGAAAAAAAGGACGAAAGGGAAAAAAAGGACGAAAGGGAAAGAAAGGACGAAAGGGAAAGAAAGGCAAACCAATCCAAACTGAAGAAACACCAGCCATTCCGACGGAGATAAAAGCTGCAGAAATAGAAAAGGAACCAAAAACAGAAGTGGTTGTGGAACCACTTATTCAGGAAAAAGATGTTGCGGAGACCGAAATACAACCCATCGAAGCAGAAGTAGAACCAGTTGAACCAAAGACAGAAATAGTTATGGAACCAGTTATTCCGGAAGCAGATATTGCGGAGACCGAAATACAACCCATCAAAGCAGAAGAAGAACCAGTT------------------------------------------------------------------------------------------------------------GAACCAAAGCCAGAAATAGTTATGGAACCAGATGAGGAACCAGTTATTCCGGAAGCAGATATTGCGGAGACCGAAATACAACCCATCAAAGCAGAAGAAGAACCAGTTGAACCAAAGCCAGAAATAGTTATGGAACCAGATGAGGAACCAGTTATTCCGGAAGCAGATATTGCGGAGACCGAAATACAACCCATCAAAGCAGAAGAAGAACCAGTTGAACCAAAGCCAGAAATAGTTATGGAACCAGATGAGGAACCAGTTATTCCGGAAGCAGATATTGCGGAGACCGAAATACAACCCATCAGAGCAGAAGAAGAACCAGTTGAACCAAAGCCAGAAATAGTTATGGAACCAGATGAGGAACCAGTCATTCCAGAAACAGAACTAACAGAGACGGACAAACAACCAATCGAAGCAGAAGTAGAACCAGTTGAACCAAAAACAGAAGTGGTTGTGGAACCAGTTATTCCGGAAGCAGATATTGCGGAGACCGAAATACAACCCATCGAAGCAGAAGTAGAACCAGTTGAACCAAAGCCAGAAATAGTTATGGAACCAGATGTGGAACCAGTAATTCCAGAAACAGATCTTACAGAGACCGAAAAACAACCAATCGAAGCAGAAGTAGAACCAGTTGAACCGAAGACAGAGATAGTCGAACCAGAAACAGAGCCAGTCGAAGAAGCCGAGGAAGAAGCGGTTGAAGCACCCGTTATGGAACCAGTTATACCTGAGATAGAACCAAAGACAGAACCGGAAGTTGAAGCCGAGGAAAAAGAAGTCGAAACAAACGTTGAACCAGAACTACTGGAGACAGGAAAACCGATTGAAATTGGCCAAGCAACNNNNNNNNNNNNNNNNNNNNNNNNNNNNNNNNNNNNNNNNNNNNNNNNNNNNNNNNNNNNNNNNNNNNNNNNNNNNNNNNNNNNNNNNNNNNNNACTGAACCAAAGGAAGAACTAGCAGGAATTGATGAGAAATGTAAGTTTTAGTAACAATTATTTATTTTATGATTCCTTTTGAGCTGTTTCGGTATGCTTCGATGCAAGGCAAATTTGTGGATGTTTTAAATCCATCATTCAATTATGTTTGCAATGTAGTCATGAAGTTTGTTCGTTTGTTCAACACAGATATGGGACATATACGCATGAGCAGACCTTTTTTGTGCACAGATCTTCGTTCAGGTTAATCGTGCATGGCAACAAAAATTGGTTCATTCCGTTTTATGTCGTGCAGTCTTATTTTGCTTGAATTAAGCATTGCTGTAAAGTTCAAGTTAAAGGTACAGTCCATCATTTGTAATTTGTGCATTTTGTTCGTTTGAAGAAACAAACATGCTCAAAATCTAAAGAAGGCTGCTGAACAACTAACCT---------------------------------------------------------------------------------------------------------------------------------------------------------------------------------------------------------------------------------------------------------------------------------------GATGAAGTTTCAGCTCAGCTTCAGCTGTATAAATTATTTCGAGAAAAACAAAATTCTAGGATCTCCATTTCAAATCAAAAGTTGCTGAATCTTGTTTTGAATTGGGCCTGAATTCAGCGTCCGAGGCACGTTCTCATCACTGAGCCGATTGCAGCTACGCGATGTCGCGCACGAATTAAAGCAATCGAGGGCGCGTGTGCGTTGCGTCAACGCTTGCGCGCGCTTCGCC-AATAAAAAAAAAAGAACTAAACGGAAATTTCTTTAGACGCATGTCTTTAGACGCACCGTTGGAAAGGCCTACTTTCGTTGCTTTCTTCAAAATGACAGCATGCCAGAAAGAAAAATCTCTTATCTTTCAACGAAGTATGTTTTTTAACAATAGGCCTAATTACGTCAGTTACTTTTTGGATAGTTGCAAATAATGATGGACTGTAGCTTTAAGAAATTTGTGGAACTCAAGTTGTGCTTGGCAAGTTTTACTTTAGAATTCGAAAATGCATCGACACCAAAAAAAAGTGTAATTTTGGTCCTATAGTTGAAGTGTCAAACTAGAACAAATTTAATGATAAATTAAATAAATTTAAATAATTTATTCCTACCTTGCTTTGACAGTGATGAAGGAGCTGCGTGACCTTTTGGAATCTACGAAGATTGACCTTCCTGTTGACATCAATGATCCATACGACCTAGGTCTTCTTCTCAGACATTTACGTCACCATTCAAATCTTCTTGCTCGTATTGGAGACCCCGATGTCAAAAAGGAAGTCCTCAGCGCCATGAATGAAAAC

>BA48-A

GCACGACGTGCCGCAGAGGAAAAACCTCAAAAGGGAAAGAAGGGACGAAAAGGAAAGAAACGACGAAAGGGAAAGAAGGGACGAAAAGGAAAGAAGGGACGAAAAGGAAAGAAACGACGAAAGGGAAAGAAAAGACGAAAGGGAAAGAAAGGACGAAAGGGAAAGAAAGGACGAAAGGGAAAGAAAGGACGAAAGGGAAAGAAAGGACGAAAGGGAAAGAAAGGACGAAAGGGAAAGAAAGGACGAAAGGGAAAGAAAGGA------------------AAACCAATCCAACCTGAAGAAACACCAGCCATTCCGACGGAGATAAAAGCTGCAGAAATAGAAAAGGAACCAAAAACAGAAGTGGTTGTGGAACCAGTTATTCCGGAAGCAGATATTGCGGAGACCGAAATACAACAAATTAAAGCAGAAGTAGAACCAGTTGAAACAAAGCCAGAAATAGTTATGGAACCAGATGTGGAACCAGTTATTCCAGAAACGGAACTTACAGAGACCGGAAAAGAAGCGAAAGTAGAACAAGTTAAACCAGTGGAAGGGAAGCTAAACTTA------------------------------------------------------------------------------------------------------------------------------------------------------------------------------------------------------------------------------------------------------------------------------------------------------------------------------------------------------------------------------------------------------------------------------------------------------------------------------GGAAAAGGAAAGGGCAAAGGAAAGAAGGTTAGAAAGGGAAAGAAAGGACGAAAGGGAAAGAAAGGACGAAAGGGAAAGAAAGGACGGAAAGGAAAGAAACGACGAAAAGGAAAGAAGAGAAGAAAAGGAAAGAAAGGACGAAAAGGAAAGAAACGACGAAAGGGAAAGAAAGGACGAAAGGGAAAGAAAGGACGAAAGGGAAAGAAAGGACGAAAGGGAAAGAAAGGACGAAAGGGAAAGAAAGGACGAAGGGGAAAGAAAGGA------------------CGAAAGGGCAAGAAAGGAAATCCAATCAAAACTGAAGAAACACCAGCCATTCTGACGGAGATAAAAGCTGCAGAATTAGAAAAGGAACCAAAAACAGAAATAGTTATGAAACCAGTTATTCCCGAAACGGAACTAACAGAGACCGGAAAAGAAGCAGAAGTAGAACAAGTTAAACCAGTGGAAAGGAAGCTAAAAATAGGAAAAGGAAAGGGCAAAGGAAAGAAG------------------AGGAGAAAGGGAAAGAAAGGACGAAAGGGAAAGAAAGGACGGAAAGGAAAGAAGGGGCGT------------------AAAGGAAAGAAAAGACGAAAAGGAAAGAAACGACGAAAGGGAAAGAAAGGACGAAAGGGAAAAAAAGGACGAAAGGGAAAGAAAGGACGAAAGGGAAAAAAAGGACGAAAGGGAAAAAAAGGACGAAAGGGAAAGAAAGGACGAAAGGGAAAGAAAGGAAAACCAATCCAAACTGAAGAAACACCAGCCATTCCGACGGAGATAAAAGCTGCAGAAATAGAAAAGGAACCAAAAACAGAAGTGGTTGTGGAACCACTTATTCAGGAAAAAGATGTTGCGGAGACCGAAATACAACCCATCGAAGCAGAAGTAGAACCAGTTGAACCAAAGACAGAAATAGTTATGGAACCAGTTATTCCGGAAGCAGATATTGCGGAGACCGAAATACAACCCATCAAAGCAGAAGAAGAACCAGTT------------------------------------------------------------------------------------------------------------------------------------------------------------------------------------------------------------------------GAACCAAAGCCAGAAATAGTTATGGAACCAGATGANGAACCAGTTATTCCGGAAGCAGATATTGCGGAGACCGAAATACAACCCATCAAAGCAGAAGAAGAACCAGTTGAACCAAAGCCAGAAATAGTTATGGAACCAGATGAGGAACCAGTTATTCCGGAAGCAGATATTGCGGAGACCGAAATACAACCCATCAGAGCAGAAGAAGAACCAGTTGAACCAAAGCCAGAAATAGTTATGGAACCAGATGAGGAACCAGTCATTCCAGAAACAGAACTAACAGAGACAGACAAACAACCAATCGAAGCAGAAGTAGAACCAGTTGAACCAAAAACAGAAGTGGTTGTGGAACCAGTTATTCCGGAAGCAGATATTGCGGAGACCGAAATACAACCCATCGAAGCAGAAGTAGAACCAGTTGAACCAAAGCCAGAAATAGTTATGGAACCAGATGTGGAACCAGTAATTCCAGAAACAGATCTTACAGAGACCGAAAAACAACCAATCGAAGCAGAAGTAGAACCAGTTGAACCGAAGACAGAGATAGTCGAACCAGAAACAGAGCCAGTCGAAGAAGCCGAGGAAGAAGCGGTTGAAGCACCCGTTATGGAACCAGTTATACCTGAGATAGAACCAAAGACAGAACCGGAAGTTGAAGCCGAGGAAAAAGAAGTCGAAACAAACGTTGAACCAGAACTACTGGAGACAGGAAAACCGATTGAAATTGGCCAAGCAACTGAAATCGAGGAGCCACCAGAAAAACAACCGGTGGAATCGGAGTCACAACCAGCGGAAGTTGAGATGGAACAAATTGAAACAAAGGAAGAACCAACTGAACCAAAGGAAGAACTAGCAGGAATTGATGAGAAATGTAAGTTTTAGTAACAATTATTTATTTTATGATTCCTTTTGAGCTGTTTCGGTATGCTTCGATGCAAGTCAAATTTGTGGATGTTTTAAATCCATCATTCAATTATGTTTGCAATGTAGTCATGAAGTTTGTTCGTTTGTTCAACACAGATATGGGACATATACGCATGAGCAGACCTTTTTTGTGCACAGATCTTCGTTCAGGTTAATCGTGCATGGCAACAAAAATTGGTTCATTCCGTTTTATGTCGTGCAGTCTTATTTTGCTTGAATTAAGCATTGCTGTAAAGTTCAAGTTAAAGGTACAGTCCATCATTTGTAATTTGTGCATTTTGTTCGTTTGAAGAAACAAACATGCTCAAAATCTAAAGAAGGCTGCTGAACAACTAACCT---------------------------------------------------------------------------------------------------------------------------------------------------------------------------------------------------------------------------------------------------------------------------------------GATGAAGTTTCAGCTCAGCTTCAGCTGTATAAATTATTTCGAGAAAAACAAAGTTCTAGGATCTCCATTTCAAATCAAAAGTTGCTGAATCTTGTTTTGAATTGGGCCTGAATTCAGCGTCCGAGGCACGTTCTCATCACTGAGCCGATTGCAGCTACGCGATGTCGCGCACGAATTAAAGCAATCGAGGGCGCGTGTGCGTTGTGTCAACGCTTGCGCGCGCTTCGCCAAATAAAAAAAAAAGAACTAAACGGAAATTTCTTTAGACGCATGTCTTTAGACGCACCGTTGGAAAGGCCTACTTTCGTTGCTTTCTTCAAAATGACAGCATGCCAGAAAGAAAAATCTCTTATCTTTCAACGAAGTATGTTTTTTAACAATANGCCTAATTACGTCAGTTACTTTTTGGATAGTTGCAAATAATGATGGACTGTAGCTTTAAGAAATTTGTGGAACTAAAGTTGTGCTTGGCAAGTTTTACTTTAGAATTCGAAAATGCATCGACAC--AAAAAAAGTGTAATTTTGGTCCTATAGTTGAAGTGTCAAACTAGAACAAATTTAATGATAAATTAAATAAATTTAAATAATTTATTCCTACCTTGCTTTGACAGTGATGAAGGAGCTGCGTGACCTTTTGGAATCTACGAAGATTGACCTTCCTGTTGACATCAATGATCCATACGACCTAGGTCTTCTTCTCAGACATTTACGTCACCATTCAAATCTTCTTGCTCGTATTGGAGACCCCGATGTCAAAAAGGAAGTCCTCAGCGCCATGAATGAAAAC

>BA62-A

GCACGACGTGCCGCAGAGGAAAAACCTCAAAAGGGAAAGAAGGGACGAAAAGGAAAGAAACGACGAAAGGGAAAGAAGGGACGAAAAGGAAAGAAGGGACGAAAAGGAAAGAAACGACGAAAGGGAAAGAAAAGACGAAAGGGAAAGAAAGGACGAAAGGGAAAGAAAGGACGAAAGGGAAAGAAAGGACGAAAGGGAAAGAAAGGACGAAAGGGAAAGAAAGGACGAAAGGGAAAGAAAGGACGAAAGGGAAAGAAAGGACGAAAGGGAAAGAAAGGAAAACCAATCCAACCTGAAGAAACACCAGCCATTCCGACGGAGATAAAAGCTGCAGAAATAGAAAAGGAACCAAAAACAGAAGTGGTTGTGGGACCAGTTATTCCGGAAGCAGATATTGCGGAGACCGAAATACAACAAATTAAAGCAGAAGTAGAACCAGTTGAAACAAAGCCAGAAATAGTTATGGAACCAGATGTGGAACCAGTTATTCCAGAAACGGAACTTACAGAGACCGGAAAAGAAGTGGAAGTAGAACAAGTTAAACCAGTGGAAGGGAAGCTAAACTTA------------------------------------------------------------------------------------------------------------------------------------------------------------------------------------------------------------------------------------------------------------------------------------------------------------------------------------------------------------------------------------------------------------------------------------------------------------------------------GGAAAAGGAAAGGGCAAAGGAAAGAAGGTTAGAAAGGGAAAGAAAGGACGAAAGGGAAAGAAAGGACGAAAGGGAAAGAAAGGACGGAAAGGAAAGAAGGGGCGAAAAGGAAAGAAAAGAAGAAAAGGAAAAAAAGGACGAAAGGGAAAGAAAGGACGAAAGGGAAAGAAAGGACGAAAGGGCAAGAAAGGACGAAAGGGAAAGAAAGGACGAAAGGGAAAGAAAGGACGAAAGGGAAAGAAAGGACGAAGGGGAAAGAAAGGACGAAGGGGAAAGAAAGGACGAAAGGGCAAGAAAGGAAATCCAATCAAAACTGAAGAAACACCAGCCATTCTGACGGAGATAAAAGCTGCAGAATTAGAAAAGGAACCAAAAACAGAAATAGTTATGAAACCAGTTATTCCCGAAACGGAACTAACAGAGACCGGAAAAGAAGCAGAAGTAGAACAAGTTAAACCAGTGGAAAGGAAGCTAAAAATAGGAAAAGGAAAGGGCAAAGGAAAGAAG------------------AGGAGAAAGGGAAAGAAAGGACGAAAGGGAAAGAAAGGACGGAAAGGAAAGAAGGGGCGT------------------AAAGGAAAGAAAAGACGAAAAGGAAAGAAACGACGAAAGGGAAAGAAAGGACGAAAGGGAAAAAAAGGACGAAAGGGAAAGAAAGGACGAAAGGGAAAAAAAGGACGAAAGGGAAAAAAAGGACGAAAGGGAAAGAAAGGACGAAAGGGAAAGAAAGGAAAACCAATCCAAACTGAAGAAACACCAGCCATTCCGACGGAGATAAAAGCTGCAGAAATAGAAAAGGAACCAAAAACAGAAGTGGTTGTGGAACCACTTATTCAGGAAAAAGATGTTGCGGAGACCGAAATACAACCCATCGAAGCAGAAGTAGAACCAGTTGAACCAAAGACAGAAATAGTTATGGAACCAGTTATTCCGGAAGCAGATATTGCGGAGACCGAAATACAACCCATCAAAGCAGAAGAAGAACCAGTT------------------------------------------------------------------------------------------------------------GAACCAAAGCCAGAAATAGTTATGGAACCAGATGAGGAACCAGTTATTCCGGAAGCAGATATTGCGGAGACCGAAATACAACCCATCAAAGCAGAAGAAGAACCAGTTGAACCAAAGCCAGAAATAGTTATGGAACCAGATGAGGAACCAGTTATTCCGGAAGCAGATATTGCGGAGACCGAAATACAACCCATCAAAGCAGAAGAAGAACCAGTTGAACCAAAGCCAGAAATAGTTATGGAACCAGATGAGGAACCAGTTATTCCGGAAGCAGATATTGCGGAGACCGAAATACAACCCATCAGAGCAGAAGAAGAACCAGTTGAACCAAAGCCAGAAATAGTTATGGAACCAGATGAGGAACCAGTCATTCCAGAAACAGAACTAACAGAGACAGACAAACAACCAATCGAAGCAGAAGTAGAACCAGTTGAACCAAAAACAGAAGTGGTTGTGGAACCAGTTATTCCGGAAGCAGATATTGCGGAGACCGAAATACAACCCATCGAAGCAGAAGTAGAACCAGTTGAACCAAAGCCAGAAATAGTTATGGAACCAGATGTGGAACCAGTAATTCCAGAAACAGATCTTACAGAGACCGAAAAACAACCAATCGAAGCAGAAGTAGAACCAGTTGAACCGAAGACAGAGATAGTCGAACCAGAAACAGAGCCAGTCGAAGAAGCCGAGGAAGAAGCGGTTGAAGCACCCGTTATGGAACCAGTTATACCTGAGATAGAACCAAAGACAGAACCGGAAGTTGAAGCCGAGGAAAAAGAAGTCGAAACAAACGTTGAACCAGAACTACTGGAGACAGGAAAACCGATTGAAATTGGCCAAGCAACTGAAATCGAGGAGCCACCAGAAAAACAACCGGTGGAATCGGAGTCACAACCAGCGGAAGTTGAGATGGAACAAATTGAAACAAAGGAAGAACCAACTGAACCAAAGGAAGAACTAGCAGGAATTGATGAGAAATGTAAGTTTTAGTAACAATTATTTATTTTATGATTCCTTTTGAGCTGTTTCGGTATGCTTCGATGCAAGTCAAATTTGTGGATGTTTTAAATCCATCATTCAATTATGTTTGCAATGTAGTCATGAAGTTTGTTCGTTTGTTCAACACAGATATGGGACATATACGCATGAGCATACCTTTTTTGTGCACAGATCTTCGTTCAGGTTAATCGTGCATGGCAACAAAAAATGGTTCATTCCGTTTTATGTCGTGCAGTCTTATTTTGCTTGAATTAAGCATTGCTGTAAAGTTCAAGTTAAAGGTACAGTCCACCATTTGTAATTTGTGCATTTTGTTCGTTTGAAGAAACAAACATGCTCAAAATCTAAAGAAGGCTGCTGAACAACTAACCT---------------------------------------------------------------------------------------------------------------------------------------------------------------------------------------------------------------------------------------------------------------------------------------GATGAAGTTTCAGCTCAGCTTCAGCTGTATAAATTATTTCGAGAAAAACAAAATTCTAGGATCTCCATTTCAAATAAAAAGTTGCTGAATCTTGTTTTGAATTGGGCCTGAATTCAGCGTCCGAGGCACGTTCTCATCACTGAGCCGATTGCAGCTACGCGATGTCGCGCACGAATTAAAGCAATCGAGGGCGCGTGTGCGTTGTGTCAACGCTTGCGCGCGCTTCGCCAAATAAAAAAAAAAGAACTAAACGGAAATTTCTTTAGACGCATGTCTTTAGACGCACCGTTGGAAAGGCCTACTTTCGTTGCTTTCTTCAAAATGACAGCATGCCAGAAAGAAAAATCTCTTATCTTTCAACGAAGTATGTTTTTTAACAATAGGCCTAATTACGTCAGTTACTTTTTGGATAGTTGCAAATAATGATGGACTGTAGCTTTAAGAAATTTGTGGAACTAAAGTTGTGCTTGGCAAGTTTTACTTTAGAATTCGAAAATGCATCGACACCAAAAAAAAGTGTAATTTTGGTCCTATAGTTGAAGTGTCAAACTAGAACAAATTTAATGATAAATTAAATAAATTTAAATAATTTATTCCTACCTTGCTTTGACAGTGATGAAGGAGCTGCGTGACCTTTTGGAATCTACGAAGATTGACCTTCCTGTTGACATCAATGATCCATACGACCTAGGTCTTCTTCTCAGACATTTACGTCACCATTCAAATCTTCTTGCTCGTATTGGAGACCCCGATGTCAAAAAGGAAGTCCTCAGCGCCATGAATGAAAAC

>BA85-B

GCACGACGTGCCGCAGAGGAAAAACCTCAAAAGGGAAAGAAGGGACGAAAAGGAAAGAAACGACGAAAGGGAAAGAAGGGACGAAAAGGAAAGAAGGGACGAAAAGGAAAGAAACGACGAAAGGGAAAGAAAAGACGAAAGGGAAAGAAAGGACGAAAGGGAAAGAAAGGACGAAAGGGAAAGAAAGGACGAAAGGGAAAGAAAGGACGAAAGGGAAAGAAAGGACGAAAGGGAAAGAAAGGACGAAAGGGAAAGAAAGGACGAAAGGGAAAGAAAGGAAAACCAATCCAACCTGAAGAAACACCAGCCATTCCGACGGAGATAAAAGCTGCAGAAATAGAAAAGGAACCAAAAACAGAAGTGGTTGTGGAACCAGTTATTCCGGAAGCAGATATTGCGGAGACCGAAATACAACAAATTAAAGCAGAAGTAGAACCAGTTGAAACAAAGCCAGAAATAGTTATGGAACCAGATGTGGAACCAGTTATTCCAGAAACGGAACTTACAGAGACCGGAAAAGAAGCGAAAGTAGAACAAGTTAAACCAGTGGAAGGGAAGCTAAACTTA------------------------------------------------------------------------------------------------------------------------------------------------------------------------------------------------------------------------------------------------------------------------------------------------------------------------------------------------------------------------------------------------------------------------------------------------------------------------------GGAAAAGGAAAGGGCAAAGGAAAGAAGGTTAGAAAGGGAAAGAAAGGACGAAAGGGAAAGAAAGGACGAAAGGGAAAGAAAGGACGGAAAGGAAAGAAGGGGCGAAAAGGAAAGAAAAGAAGAAAAGGAAAAAAAGGACGAAAGGGAAAGAAAGGACGAAAGGGAAAGAAAGGACGAAAGGGAAAGAAAGGACGAAAGGGAAAGAAAGGACGAAAGGGAAAGAAAGGACGAAAGGGAAAGAAAGGACGAAAGGGAAAGAAAGGACGAAGGGGAAAGAAAGGACGAAAGGGCAAGAAAGGAAATCCAATCAAAACTGAAGAAACACCAGCCATTCTGACGGAGATAAAAGCTGCAGAATTAGAAAAGGAACCAAAAACAGAAATAGTTATGAAACCAGTTATTCCCGAAACGGAACTAACAGAGACCGGAAAAGAAGCAGAAGTAGAACAAGTTAAACCAGTGGAAAGGAAGCTAAAAATAGGAAAAGGAAAGGGCAAAGGAAAGAAG------------------AGGAGAAAGGGAAAGAAAGGACGAAAGGGAAAGAAAGGACGGAAAGGAAAGAAGGGGCGT------------------AAAGGAAAGAAAAGACGAAAAGGAAAGAAACGACGAAAGGGAAAGAAAGGACGAAAGGGAAAAAAAGGACGAAAGGGAAAGAAAGGACGAAAGGGAAAAAAAGGACGAAAGGGAAAAAAAGGACGAAAGGGAAAGAAAGGACGAAAGGGAAAGAAAGGAAAACCAATCCAAACTGAAGAAACACCGGCCATTCCGACGGAGATAAAAGCTGCAGAAATAGAAAAGGAACCAAAAACAGAAGTGGTTGTGGAACCACTTATTCAGGAAAAAGATGTTGCGGAGACCGAAATACAACCCATCGAAGCAGAAGTAGAACCAGTTGAACCAAAGACAGAAATAGTTATGGAACCAGTTATTCCGGAAGCAGATATTGCGGAGACCGAAATACAACCCATCAAAGCAGAAGAAGAACCAGTT------------------------------------------------------------------------------------------------------------GAACCAAAGCCAGAAATAGTTATGGAACCAGATGAGGAACCAGTTATTCCGGAAGCAGATATTGCGGAGACCGAAATACAACCCATCAAAGCAGAAGAAGAACCAGTTGAACCAAAGCCAGAAATAGTTATGGAACCAGATGAGGAACCAGTTATTCCGGAAGCAGATATTGCGGAGACCGAAATACAACCCATCAAAGCAGAAGAAGAACCAGTTGAACCAAAGCCAGAAATAGTTATGGAACCAGATGAGGAACCAGTTATTCCGGAAGCAGATATTGCGGAGACCGAAATACAACCCATCAGAGCAGAAGAAGAACCAGTTGAACCAAAGCCAGAAATAGTTATGGAACCAGATGAGGAACCAGTCATTCCAGAAACAGAACTAACAGAGACAGACAAACAACCAATCGAAGCAGAAGTAGAACCAGTTGAACCAAAAACAGAAGTGGTTGTGGAACCAGTTATTCCGGAAGCAGATATTGCGGAGACCGAAATACAACCCATCGAAGCAGAAGTAGAACCAGTTGAACCAAAGCCAGAAATAGTTATGGAACCAGATGTGGAACCAGTAATTCCAGAAACAGATCTTACAGAGACCGAAAAACAACCAATCGAAGCAGAAGTAGAACCAGTTGAACCGAAGACAGAGATAGTCGAACCAGAAACAGAGCCAGTCGAAGAAGCCGAGGAAGAAGCGGTTGAAGCACCCGTTATGGAACCAGTTATACCTGAGATAGAACCAGAGACAGAACCGGAAGTTGAAGCCGAGGAAAAAGAAGTCGAAACAAACGTTGAACCAGAACTACTGGAGACAGGAAAACCGATTGAAATTGGCCAAGCAACTGAAATCGAGGAGCCACCAGAAAAACAACCGGTGGAATCGGAGTCACAACCAGCGGAAGTTGAGATGGAACAAATTGAAACAAAGGAAGAACCAACTGAACCAAAGGAAGAACTAGCAGGAATTGATGAGAAATGTAAGTTTTAGTAACAATTATTTATTTTATGATTCCTTTTGAGCTGTTTCGGTATGCTTCGATGCAAGTCAAATTTGTGGATGTTTTAAATCCATCATTCAATTATGTTTGCAATGTAGTCATGAAGTTTGTTCGTTTGTTCAACACAGATATGGGACATATACGCATGAGCAGACCTTTTTTGTGCACAGATCTTCGTTCAGGTTAATCGTGCATGGCAACAAAAATTGGTTCATTCCGTTTTATGTCGTGCAGTCTTATTTTGCTTGAATTAAGCATTGCTGTAAAGTTCAAGTTAAAGGTACAGTCCATCATTTGTAATTTGTGCATTTTGTTCGTTTGAAGAAACAAACATGCTCAAAATCTAAAGAAGGCTGCTGAACAACTAACCT---------------------------------------------------------------------------------------------------------------------------------------------------------------------------------------------------------------------------------------------------------------------------------------GATGAAGTTTCAGCTCAGCTTCAGCTGTATAAATTATTTCGAGAAAAACAAAATTCTAGGATCTCCATTTCAAATAAAAAGTTGCTGAATTTTGTTTTGAATTGGGCCTGAATTCAGCGTCCGAGGCACGTTCTCATCACTGAGCCGATTGCAGCTACGCGATGTCGCGCACGAATTACAGCAATCGAGGGCGCGTGTGCGTTGTGTCAACGCTTGCGCGCGCTTCGCCAAATAAAAAAAAAAGAACTAAACGGAAATTTCTTTAGACGCATGTCTTTAGACGCACCGTTGGAAAGGCCTACTTTCGTTGCTTTCTTCAAAATGACAGCATGCCAGAAAGAAAAATCTCTTATCTTTCAACGAAGTATGTTTTTTAACAATAGGCCTAATTACGTCAGTTACTTTTTGGATAGTTGCAAATAATGATGGACTGTAGCTTTAAGAAATTTGTGGAACTAAAGTTGTGCTTGGCAAGTTTTACTTTAGAATTCGAAAATGCATCGACACCAAAAAAAAGTGTAATTTTGGTCCTATAGTTGAAGTGTCAAACTAGAACAAATTTAATGATAAATTAAATAAATTTAAATAATTTATTCCTACCTTGCTTTGACAGTGATGAAGGAGCTGCGTGACCTTTTGGAATCTACGAAGATTGACCTTCCTGTTGACATCAATGATCCATACGACCTAGGTCTTCTTCTCAGACATTTACGTCACCATTCAAATCTTCTTGCTCGTATTGGAGACCCCGATGTCAAAAAGGAAGTCCTCAGCGCCATGAATGAAAAC

>BA48-B

GCACGACGTGCCGCAGAGGAAAAACCTCAAAAGGGAAAGAAGGGACGAAAAGGAAAGAAACGACGAAAGGGAAAGAAGGGACGAAAAGGAAAGAAGGGACGAAAAGGAAAGAAACGACGAAAGGGAAAGAAAAGACGAAAGGGAAAGAAAGGACGAAAGGGAAAGAAAGGACGAAAGGGAAAGAAAGGACGAAAGGGAAAGAAAGGACGAAAGGGAAAGAAAGGACGAAAGGGAAAGAAAGGACGAAAGGGAAAGAAAGGACGAAAGGGAAAGAAAGGAAAACCAATCCAACCTGAAGAAACACCAGCCATTCCGACGGAGATAAAAGCTGCAGAAATAGAAAAGGAACCAAAAACAGAAGTGGTTGTGGAACCAGTTATTCCGGAAGCAGATATTGCGGAGACCGAAATACAACAAATTAAAGCAGAAGTAGAACCAGTTGAAACAAAGCCAGAAATAGTTATGGAACCAGATGTGGAACCAGTTATTCCAGAAACGGAACTTACAGAGACCGGAAAAGAAGCGAAAGTAGAACAAGTTAAACCAGTGGAAGGGAAGCTAAACTTA------------------------------------------------------------------------------------------------------------------------------------------------------------------------------------------------------------------------------------------------------------------------------------------------------------------------------------------------------------------------------------------------------------------------------------------------------------------------------GGAAAAGGAAAGGGCAAAGGAAAGAAGGTTAGAAAGGGAAAGAAAGGACGAAAGGGAAAGAAAGGACGAAAGGGAAAGAAAGGACGGAAAGGAAAGAAGGGGCGAAAAGGAAAGAAAAGAAGAAAAGGAAAAAAAGGACGAAAGGGAAAGAAAGGACGAAAGGGAAAGAAAGGACGAAAGGGCAAGAAAGGACGAAAGGGAAAGAAAGGACGAAAGGGAAAGAAAGGACGAAAGGGAAAGAAAGGACGAAGGGGAAAGAAAGGA------------------CGAAAGGGCAAGAAAGGAAATCCAATCAAAACTGAAGAAACACCAGCCATTCTGACGGAGATAAAAGCTGCAGAATTAGAAAAGGAACCAAAAACAGAAATAGTTATGAAACCAGTTATTCCCGAAACGGAACTAACAGAGACCGGAAAAGAAGCAGAAGTAGAACAAGTTAAACCAGTGGAAAGGAAGCTAAAAATAGGAAAAGGAAAGGGCAAAGGAAAGAAG------------------AGGAGAAAGGGAAAGAAAGGACGAAAGGGAAAGAAAGGACGGAAAGGAAAGAAGGGGCGT------------------AAAGGAAAGAAAAGACGAAAAGGAAAGAAACGACGAAAGGGAAAGAAAGGACGAAAGGGAAAAAAAGGACGAAAGGGAAAGAAAGGACGAAAGGGAAAAAAAGGACGAAAGGGAAAAAAAGGACGAAAGGGAAAGAAAGGACGAAAGGGAAAGAAAGGAAAACCAATCCAAACTGAAGAAACACCAGCCATTCCGACGGAGATAAAAGCTGCAGAAATAGAAAAGGAACCAAAAACAGAAGTGGTTGTGGAACCACTTATTCAGGAAAAAGATGTTGCGGAGACCGAAATACAACCCATCGAAGCAGAAGTAGAACCAGTTGAACCAAAGACAGAAATAGTTATGGAACCAGTTATTCCGGAAGCAGATATTGCGGAGACCGAAATACAACCCATCAAAGCAGAAGAAGAACCAGTT------------------------------------------------------------------------------------------------------------------------------------------------------------------------------------------------------------------------GAACCAAAGCCAGAAATAGTTATGGAACCAGATGAGGAACCAGTTATTCCGGAAGCAGATATTGCGGAGACCGAAATACAACCCATCAAAGCAGAAGAAGAACCAGTTGAACCAAAGCCAGAAATAGTTATGGAACCAGATGAGGAACCAGTTATTCCGGAAGCAGATATTGCGGAGACCGAAATACAACCCATCAGAGCAGAAGAAGAACCAGTTGAACCAAAGCCAGAAATAGTTATGGAACCAGATGAGGAACCAGTCATTCCAGAAACAGAACTAACAGAGACAGACAAACAACCAATCGAAGCAGAAGTAGAACCAGTTGAACCAAAAACAGAAGTGGTTGTGGAACCAGTTATTCCGGAAGCAGATATTGCGGAGACCGAAATACAACCCATCGAAGCAGAAGTAGAACCAGTTGAACCAAAGCCAGAAATAGTTATGGAACCAGATGTGGAACCAGTAATTCCAGAAACAGATCTTACAGAGACCGAAAAACAACCAATCGAAGCAGAAGTAGAACCAGTTGAACCGAAGACAGAGATAGTCGAACCAGAAACAGAGCCAGTCGAAGAAGCCGAGGAAGAAGCGGTTGAAGCACCCGTTATGGAACCAGTTATACCTGAGATAGAACCAAAGACAGAACCGGAAGTTGAAGCCGAGGAAAAAGAAGTCGAAACAAACGTTGAACCAGAACTACTGGAGACAGGAAAACCGATTGAAATTGGCCAAGCAACTGAAATCGAGGAGCCACCAGAAAAACAACCGGTGGAATCGGAGTCACAACCAGCGGAAGTTGAGATGGAACAAATTGAAACAAAGGAAGAACCAACTGAACCAAAGGAAGAACTAGCAGGAATTGATGAGAAATGTAAGTTTTAGTAACAATTATTTATTTTATGATTCCTTTTGAGCTGTTTCGGTATGCTTCGATGCAAGTCAAATTTGTGGATG----------------------------CAATGTAGTCATGAAGTTTGTTCGTTTGTTCAACACAGATATGGGACATATACGCATGAGCAGACCTTTTTTGTGCACAGATCTTCGTTCAGGTTAATCGTGCATGGCAACAAAAATTGGTTCATTCCGTTTTATGTCGTGCAGTCTTATTTTGCTTGAATTAAGCATTGCTGTAAAGTTCAAGTTAAAGGTACAGTCCATCATTTGTAATTTGTGCATTTTGTTCGTTTGAAGAAACAAACATGCTCAAAATCTAAAGAAGGCTGCTGAACAACTAACCT---------------------------------------------------------------------------------------------------------------------------------------------------------------------------------------------------------------------------------------------------------------------------------------GATGAAGTTTCAGCTCAGCTTCAGCTGTATAAATTATTTCGAGAAAAACAAAATTCTAGGATCTCCGTTTCAAATAAAAAGTTGCTGAATCTTGTTTTGAATTGGGCCTGAATTCAGCGTCCGAGGCACGTTCTCATCACTGAGCCGATTGCAGCTACGCGATGTCGCGCACGAATTAAAGCAATCGAGGGCGCGTGTGCGTTGTGTCAACGCTTGCGCGCGCTTCGCCAAATAAAAAAAAAAGAACTAAACGGAAATTTCTTTAGACGCATGTCTTTAGACGCACCGTTGGAAAGGCCTACTTTCGTTGCTTTCTTCAAAATGACAGCATGCCAGAAAGAAAAATCTCTTATCTTTCAACGAAGTATGTTTTTTAACAATAGGCCTAATTACGTCAGTTACTTTTTGGATAGTTGCAAATAANGATGGACTGTAGCTTTAAGAAATTTGTGGAACTAAAGTTGTGCTTGGCAAGTTTTACTTTAGAATTCGAAAATGCATCGACACCAAAAAAAAGTGTAATTTTGGTCCTATAGTTGAATTGTCAAACTAGAACAAATTTAATGATAAATTAAATAAATTTAAATAATTTATTCCTACCTTGCTTTGACAGTGATGAAGGAGCTGCGTGACCTTTTGGAATCTACGAAGATTGACCTTCCTGTTGACATCAATGATCCATACGACCTAGGTCTTCTTCTCAGACATTTACGTCACCATTCAAATCTTCTTGCTCGTATTGGAGACCCCGATGTCAAAAAGGAAGTCCTCAGCGCCATGAATGAAAAC

>BA82-A

GCACGACGTGCCGCAGAGGAAAAACCTCAAAAGGGAAAGAAGGGACGAAAAGGAAAGAAACGACGAAAGGGAAAGAAGGGACGAAAAGGAAAGAAGGGACGAAAAGGAAAGAAACGACGAAAGGGAAAGAAAAGACGAAAGGGAAAGAAAGGACGAAAGGGAAAGAAAGGACGAAAGGGAAAGAAAGGACGAAAGGGAAAGAAAGGACGAAAGGGAAAGAAAGGACGAAAGGGAAAGAAAGGACGAAAGGGAAAGAAAGGACGAAAGGGAAAGAAAGGAAAACCAATCCAACCTGAAGAAACACCAGCCATTCCGACGGAGATAAAAGCTGCAGAAATAGAAAAGGAACCAAAAACAGAAGTGGTTGTGGAACCAGTTATTCCGGAAGCAGATATTGCGGAGACCGAAATACAACAAATTAAAGCAGAAGTAGAACCAGTTGAAACAAAGCCAGAAATAGTTATGGAACCAGATGTGGAACCAGTTATTCCAGAAACGGAACTTACAGAGACCGGAAAAGAAGCGAAAGTAGAACAAGTTAAACCAGTGGAAGGGAAGCTAAACTTA------------------------------------------------------------------------------------------------------------------------------------------------------------------------------------------------------------------------------------------------------------------------------------------------------------------------------------------------------------------------------------------------------------------------------------------------------------------------------GGAAAAGGAAAGGGCAAAGGAAAGAAGGTTAGAAAGGGAAAGAAAGGACGAAAGGGAAAGAAAGGACGAAAGGGAAAGAAAGGACGGAAAGGAAAGAAGGGGCGAAAAGGAAAGAAAAGAAGAAAAGGAAAAAAAGGACGAAAGGGAAAGAAAGGACGAAAGGGAAAGAAAGGACGAAAGGGCAAGAAAGGACGAAAGGGAAAGAAAGGACGAAAGGGAAAGAAAGGACGAAAGGGAAAGAAAGGACGAAGGGGAAAGAAAGGA------------------CGAAAGGGCAAGAAAGGAAATCCAATCAAAACTGAAGAAACACCAGCCATTCTGACGGAGATAAAAGCTGCAGAATTAGAAAAGGAACCAAAAACAGAAATAGTTATGAAACCAGTTATTCCCGAAACGGAACTAACAGAGACCGGAAAAGAAGCAGAAGTAGAACAAGTTAAACCAGTGGAAAGGAAGCTAAAAATAGGAAAAGGAAAGGGCAAAGGAAAGAAG------------------AGGAGAAAGGGAAAGAAAGGACGAAAGGGAAAGAAAGGACGGAAAGGAAAGAAGGGGCGT------------------AAAGGAAAGAAAAGACGAAAAGGAAAGAAACGACGAAAGGGAAAGAAAGGACGAAAGGGAAAAAAAGGACGAAAGGGAAAGAAAGGACGAAAGGGAAAAAAAGGACGAAAGGGAAAAAAAGGACGAAAGGGAAAGAAAGGACGAAAGGGAAAGAAAGGAAAACCAATCCAAACTGAAGAAACACCAGCCATTCCGACGGAGATAAAAGCTGCAGAAATAGAAAAGGAACCAAAAACAGAAGTGGTTGTGGAACCACTTATTCAGGAAAAAGATGTTGCGGAGACCGAAATACAACCCATCGAAGCAGAAGTAGAACCAGTTGAACCAAAGACAGAAATAGTTATGGAACCAGTTATTCCGGAAGCAGATATTGCGGAGACCGAAATACAACCCATCAAAGCAGAAGAAGAACCAGTT------------------------------------------------------------------------------------------------------------GAACCAAAGCCAGAAATAGTTATGGAACCAGATGAGGAACCAGTTATTCCGGAAGCAGATATTGCGGAGACCGAAATACAACCCATCAAAGCAGAAGAAGAACCAGTTGAACCAAAGCCAGAAATAGTTATGGAACCAGATGAGGAACCAGTTATTCCGGAAGCAGATATTGCGGAGACCGAAATACAACCCATCAAAGCAGAAGAAGAACCAGTTGAACCAAAGCCAGAAATAGTTATGGAACCAGATGAGGAACCAGTTATTCCGGAAGCAGATATTGCGGAGACCGAAATACAACCCATCAAAGCAGAAGAAGAACCAGTTGAACCAAAGCCAGAAATAGTTATGGAACCAGATGAGGAACCAGTTATTCCAGAAACAGAACTAACAGAGACAGACAAACAACCAATCGAAGCAGAAGTAGAACCAGTTGAACCAAAAACAGAAGTGGTTGTGGAACCAGTTATTCCGGAAGCAGATATTGCGGAGACCGAAATACAACCCATCGAAGCAGAAGTAGAACCAGTTGAACCAAAGCCAGAAATAGTTATGGAACCAGATGTGGAACCAGTAATTCCAGAAACAGATCTTACAGAGACCGAAAAACAACCAATCGAAGCAGAAGTAGAACCAGTTGAACCGAAGACAGAGATAGTCGAACCAGAAACAGAGCCAGTCGAAGAAGCCGAGGAAGAAGCGGTTGAAGCACCCGTTATGGAACCAGTTATACCTGAGATAGAACCAAAGACAGAACCGGAAGTTGAAGCCGAGGAAAAAGAAGTCGAAACAAACGTTGAACCAGAACTACTGGAGACAGGAAAACCGATTGAAATTGGCCAAGCAACTGAAATCGAGGAGCCACCAGAAAAACAACCGGTGGAATCGGAGTCACAACCAGCGGAAGTTGAGATGGAACAAATTGAAACAAAGGAAGAACCAACTGAACCAAAGGAAGAACTAGCAGGAATTGATGAGAAATGTAAGTTTTAGTAACAATTATTTATTTTATGATTCCTTTTGAGCTGTTTCGGTATGCTTCGATGCAAGTCAAATTTGTGGATGTTTTAAATCCATCATTCAACTATGTTTGCAATGTAGTCATGAAGTTTGTTCGTTTGTTCAACACAGATATGGGACATATACGCATGAGCAGACCTTTTTTGTGCACAGATCTTCGTTCAGGTTAATCGTGCATGGCAACAAAAATTGGTTCATTCCGTTTTATGTCGTGCAGTCTTATTTTGCTTGAATTAAGCATTGCTGTAAAGTTCAAGTTAAAGGTACAGTCCATCATTTGTAATTTGTGCATTTTGTTCGTTTGAAGAAACAAACATGCTCAAAATCTAAAGAAGGCTGCTGAACAACTAACCT---------------------------------------------------------------------------------------------------------------------------------------------------------------------------------------------------------------------------------------------------------------------------------------GATGAAGTTTCAGCTCAGCTTCAGCTGTATAAATTATTTCGAGAAAAACAAAATTCTAGGATCTCCGTTTCAAATAAAAAGTTGCTGAATCTTGTTTTGAATTGGGCCTGAATTCAGCGTCCGAGGCACGTTCTCATCACTGAGCCGATTGCAGCTACGCGATGTCGCGCACGAATTAAAGCAATCGAGGGCGCGTGTGCGTTGTGTCAACGCTTGCGCGCGCTTCGCCAAATAAAAAAAAAAGAACTAAACGGAAATTTCTTTAGACGCATGTCTTTAGACGCACCGTTGGAAAGGCCTACTTTCGTTGCTTTCTTCAAAATGACAGCATGCCAGAAAGAAAAATCTCTTATCTTTCAACGAAGTATGTTTTTTAACAATAGGCCTAATTACGTCAGTTACTTTTTGGATAGTTGCAAATAATGATGGACTGTAGCTTTAAGAAATTTGTGGAACTAAAGTTGTGCTTGGCAAGTTTTACTTTAGAATTCGAAAATGCATCGACACCAAAAAAAAGTGTAATTTTGGTCCTATAGTTGAATTGTCAAACTAGAACAAATTTAATGATAAATTAAATAAATTTAAATAATTTATTCCTACCTTGCTTTGACAGTGATGAAGGAGCTGCGTGACCTTTTGGAATCTACGAAGATTGACCTTCCTGTTGACATCAATGATCCATACGACCTAGGTCTTCTTCTCAGACATTTACGTCACCATTCAAATCTTCTTGCTCGTATTGGAGACCCCGATGTCAAAAAGGAAGTCCTCAGCGCCATGAATGAAAAC

>BA84-B

GCACGACGTGCCGCAGAGGAAAAACCTCAAAAGGGAAAGAAGGGACGAAAAGGAAAGAAACGACGAAAGGGAAAGAAGGGACGAAAAGGAAAGAAGGGACGAAAAGGAAAGAAACGACGAAAGGGAAAGAAAAGACGAAAGGGAAAGAAAGGACGAAAGGGAAAGAAAGGACGAAAGGGAAAGAAAGGACGAAAGGGAAAGAAAGGACGAAAGGGAAAGAAAGGACGAAAGGGAAAGAAAGGACGAAAGGGAAAGAAAGGACGAAAGGGAAAGAAAGGAAAACCAATCCAACCTGAAGAAACACCAGCCATTCCGACGGAGATAAAAGCTGCAGAAATAGAAAAGGAACCAAAAACAGAAGTGGTTGTGGAACCAGTTATTCCGGAAGCAGATATTGCGGAGACCGAAATACAACAAATTAAAGCAGAAGTAGAACCAGTTGAAACAAAGCCAGAAATAGTTATGGAACCAGATGTGGAACCAGTTATTCCAGAAACGGAACTTACAGAGACCGGAAAAGAAGCGAAAGTAGAACAAGTTAAACCAGTGGAAGGGAAGCTAAACTTA------------------------------------------------------------------------------------------------------------------------------------------------------------------------------------------------------------------------------------------------------------------------------------------------------------------------------------------------------------------------------------------------------------------------------------------------------------------------------GGAAAAGGAAAGGGCAAAGGAAAGAAGGTTAGAAAGGGAAAGAAAGGACGAAAGGGAAAGAAAGGACGAAAGGGAAAGAAAGGACGGAAAGGAAAGAAGGGACGAAAAGGAAAGAAAAGAAGAAAAGGAAAAAAAGGACGAAAGGGAAAGAAAGGACGAAAGGGAAAGAAAGGACGAAAGGGCAAGAAAGGACGAAAGGGAAAGAAAGGACGAAAGGGAAAGAAAGGACGAAAGGGAAAGAAAGGACGAAGGGGAAAGAAAGGA------------------CGAAAGGGCAAGAAAGGAAATCCAATCAAAACTGAAGAAACACCAGCCATTCTGACGGAGATAAAAGCTGCAGAATTAGAAAAGGAACCAAAAACAGAAATAGTTATGAAACCAGTTATTCCCGAAACGGAACTAACAGAGACCGGAAAAGAAGCAGAAGTAGAACAAGTTAAACCAGTGGAAAGGAAGCTAAAAATAGGAAAAGGAAAGGGCAAAGGAAAGAAG------------------AGGAGAAAGGGAAAGAAGGGACGAAAGGGAAAGAAAGGACGGAAAGGAAAGAAGGGGCGT------------------AAAGGAAAGAAAAGACGAAAAGGAAAGAAACGACGAAAGGGAAAGAAAGGACGAAAGGGAAAAAAAGGACGAAAGGGAAAGAAAGGACGAAAGGGAAAAAAAGGACGAAAGGGAAAAAAAGGACGAAAGGGAAAGAAAGGACGAAAGGGAAAGAAAGGAAAACCAATCCAAACTGAAGAAACACCAGCCATTCCGACGGAGATAAAAGCTGCAGAAATAGAAAAGGAACCAAAAACAGAAGTGGTTGTGGAACCACTTATTCAGGAAAAAGATGTTGCGGAGACCGAAATACAACCCATCGAAGCAGAAGTAGAACCAGTTGAACCAAAGACAGAAATAGTTATGGAACCAGTTATTCCGGAAGCAGATATTGCGGAGACCGAAATACAACCCATCAAAGCAGAAGAAGAACCAGTT------------------------------------------------------------------------------------------------------------GAACCAAAGCCAGAAATAGTTATGGAACCAGATGAGGAACCAGTTATTCCGGAAGCAGATATTGCGGAGACCGAAATACAACCCATCAAAGCAGAAGAAGAACCAGTTGAACCAAAGCCAGAAATAGTTATGGAACCAGATGAGGAACCAGTTATTCCGGAAGCAGATATTGCGGAGACCGAAATACAACCCATCAAAGCAGAAGAAGAACCAGTTGAACCAAAGCCAGAAATAGTTATGGAACCAGATGAGGAACCAGTTATTCCGGAAGCAGATATTGCGGAGACCGAAATACAACCCATCAGAGCAGAAGAAGAACCAGTTGAACCAAAGCCAGAAATAGTTATGGAACCAGATGAGGAACCAGTCATTCCAGAAACAGAACTAACAGAGACAGACAAACAACCAATCGAAGCAGAAGTAGAACCAGTTGAACCAAAAACAGAAGTGGTTGTGGAACCAGTTATTCCGGAAGCAGATATTGCGGAGACCGAAATACAACCCATCGAAGCAGAAGTAGAACCAGTTGAACCAAAGCCAGAAATAGTTATGGAACCAGATGTGGAACCAGTAATTCCAGAAACAGATCTTACAGAGACCGAAAAACAACCAATCGAAGCAGAAGTAGAACCAGTTGAACCGAAGACAGAGATAGTCGAACCAGAAACAGAGCCAGTCGAAGAAGCCGAGGAAGAAGCGGTTGAAGCACCCGTTATGGAACCAGTTATACCTGAGATAGAACCAAAGACAGAACCGGAAGTTGAAGCCGAGGAAAAAGAAGTCGAAACAAACGTTGAACCAGAACTACTGGAGACAGGAAAACCGTTTGAAATTGGCCAAGCAACTGAAATCGAGGAGCCACCAGAAAAACAACCGGTGGAATCGGAGTCACAACCAGCGGAAGTTGAGATGGAACAAATTGAAACAAAGGAAGAACCAACTGAACCAAAGGAAGAACTAGCAGGAATTGATGAGAAATGTAAGTTTTAGTAACAATTATTTATTTTATGATTCCTTTTGAGCTGTTTCGGTATGCTTCGATGCAAGTCAAATTTGTGGATGTTTTAAATCCATCATTCAATTATGTTTGCAATGTAGTCATGAAGTTTGTTCGTTTGTTCAACACAGATATGGGACATATACGCATGAGCAGACCTTTTTTGTGCACAGATCTTCGTTCAGGTTAATCGTGCATGGCAACAAAAATTGGTTCATTCCGTTTTATGTCGTGCAGTCTTATTTTGCTTGAATTAAGCATTGCTGTAAAGTTCAAGTTAAAGGTACAGTCCATCATTTGTAATTTGTGCATTTTGTTCGTTTGAAGAAACAAACATGCTCAAAATCTAAAGAAGGCTGCTGAACAACTAACCT---------------------------------------------------------------------------------------------------------------------------------------------------------------------------------------------------------------------------------------------------------------------------------------GATGAAGTTTCAGCTCAGCTTCAGCTGTATAAATTATTTCGAGAAAAACAAAATTCTAGGATCTCCGTTTCAAATAAAAAGTTGCTGAATCTTGTTTTGAATTGGGCCTGAATTCAGCGTCCGAGGCACGTTCTCATCACTGAGCCGATTGCAGCTACGCGATGTCGCGCACGAATTAAAGCAATCGAGGGCGCGTGTGCGTTGTGTCAACGCTTGCGCGCGCTTCGCCAAAT--AAAAAAAAGAACTAAACGGAAATTTCTTTAGACGCATGTCTTTAGACGCACCGTTGGAAAGGCCTACTTTCGTTGCTTTCTTCAAAATGACAGCATACCAGAAAGAAAAATCTCTTATCTTTCAACGAAGTATGTTTTTTAACAATAGGCCTAATTACGTCAGTTACTTTTTGGATAGTTGCAAATAATGATGGACTGTAGCTTTAAGAAATTTGTGGAACTAAAGTTGTGCTTGGCAAGTTTTACTTTAGAATTCGAAAATGCATCGACACCAAAAAAAAGTGTAATTTTGGTCCTATAGTTGAAGTGTCAAACTAGAACAAATTTAATGATAAATTAAATAAATTTAAATAATTTATTCCTACCTTGCTTTGACAGTGATGAAGGAGCTGCGTGACCTTTTGGAATCTACGAAGATTGACCTTCCTGTTGACATCAATGATCCATACGACCTAGGTCTTCTTCTCAGACATTTACGTCACCATTCAAATCTTCTTGCTCGTATTGGAGACCCCGATGTCAAAAAGGAAGTCCTCAGCGCCATGAATGAAAAC

>BA86-B

GCACGACGTGCCGCAGAGGAAAAACCTCAAAAGGGAAAGAAGGGACGAAAAGGAAAGAAACGACGAAAGGGAAAGAAGGGACGAAAAGGAAAGAAGGGACGAAAAGGAAAGAAACGACGAAAGGGAAAGAAAAGACGAAAGGGAAAGAAAGGACGAAAGGGAAAGAAAGGACGAAAGGGAAAGAAAGGACGAAAGGGAAAGAAAGGACGAAAGGGAAAGAAAGGACGAAAGGGAAAGAAAGGACGAAAGGGAAAGAAAGGACGAAAGGGAAAGAAAGGAAAACCAATCCAACCTGAAGAAACACCAGCCATTCCGACGGAGATAAAAGCTGCAGAAATAGAAAAGGAACCAAAAACAGAAGTGGTTGTGGAACCAGTTATTCCGGAAGCAGATATTGCGGAGACCGAAATACAACAAATTAAAGCAGAAGTAGAACCAGTTGAAACAAAGCCAGAAATAGTTATGGAACCAGATGTGGAACCAGTTATTCCAGAAACGGAACTTACAGAGACCGGAAAAGAAGCGAAAGTAGAACAAGTTAAACCAGTGGAAGGGAAGCTAAACTTA------------------------------------------------------------------------------------------------------------------------------------------------------------------------------------------------------------------------------------------------------------------------------------------------------------------------------------------------------------------------------------------------------------------------------------------------------------------------------GGAAAAGGAAAGGGCAAAGGAAAGAAGGTTAGAAAGGGAAAGAAAGGACGAAAGGGAAAGAAAGGACGAAAGGGAAAGAAAGGACGGAAAGGAAAGAAGGGGCGAAAAGGAAAGAAAAGAAGAAAAGGAAAAAAAGGACGAAAGGGAAAGAAAGGACGAAAGGGAAAGAAAGGACGAAAGGGCAAGAAAGGACGAAAGGGAAAGAAAGGACGAAAGGGAAAGAAAGGACGAAAGGGAAAGAAAGGACGAAAGGGAAAGAAAGGA------------------CGAAAGGGCAAGAAAGGAAATCCAATCAAAACTGAAGAAACACCAGCCATTCTGACGGAGATAAAAGCTGCAGAATTAGAAAAGGAACCAAAAACAGAAATAGTTATGAAACCAGTTATTCCCGAAACGGAACTAACAGAGACCGGAAAAGAAGCAGAAGTAGAACAAGTTAAACCAGTGGAAAGGAAGCTAAAAATAGGAAAAGGAAAGGGCAAAGGAAAGAAG------------------AGGAGAAAGGGAAAGAAAGGACGAAAGGGAAAGAAAGGACGGAAAGGAAAGAAGGGGCGT------------------AAAGGAAAGAAAAGACGAAAAGGAAAGAAACGACGAAAGGGAAAGAAAGGACGAAAGGGAAAAAAAGGACGAAAGGGAAAGAAAGGACGAAAGGGAAAAAAAGGACGAAAGGGAAAAAAAGGACGAAAGGGAAAGAAAGGACGAAAGGGAAAGAAAGGAAAACCAATCCAAACTGAAGAAACACCAGCCATTCCGACGGAGATAAAAGCTGCAGAAATAGAAAAGGAACCAAAAACAGAAGTGGTTGTGGAACCACTTATTCAGGAAAAAGATGTTGCGGAGACCGAAATACAACCCATCGAAGCAGAAGTAGAACCAGTTGAACCAAAGACAGAAATAGTTATGGAACCAGTTATTCCGGAAGCAGATATTGCGGAGACCGAAATACAACCCATCAAAGCAGAAGAAGAACCAGTT------------------------------------------------------------------------------------------------------------GAACCAAAGCCAGAAATAGTTATGGAACCAGATGAGGAACCAGTTATTCCGGAAGCAGATATTGCGGAGACCGAAATACAACCCATCAAAGCAGAAGAAGAACCAGTTGAACCAAAGCCAGAAATAGTTATGGAACCAGATGAGGAACCAGTTATTCCGGAAGCAGATATTGCGGAGACCGAAATACAACCCATCAAAGCAGAAGAAGAACCAGTTGAACCAAAGCCAGAAATAGTTATGGAACCAGATGAGGAACCAGTTATTCCGGAAGCAGATATTGCGGAGACCGAAATACAACCCATCAGAGCAGAAGAAGAACCAGTTGAACCAAAGCCAGAAATAGTTATGGAACCAGATGAGGAACCAGTCATTCCAGAAACAGAACTAACAGAGACAGACAAACAACCAATCGAAGCAGAAGTAGAACCAGTTGAACCAAAAACAGAAGTGGTTGTGGAACCAGTTATTCCGGAAGCAGATATTGCGGAGACCGAAATACAACCCATCGAAGCAGAAGTAGAACCAGTTGAACCAAAGCCAGAAATAGTTATGGAACCAGATGTGGAACCAGTAATTCCAGAAACAGATCTTACAGAGACCGAAAAACAACCAATCGAAGCAGAAGTAGAACCAGTTGAACCGAAGACAGAGATAGTCGAACCAGAAACAGAGCCAGTCGAAGAAGCCGAGGAAGAAGCGGTTGAAGCACCCGTTATGGAACCAGTTATACCTGAGATAGAACCAAAGACAGAACCGGAAGTTGAAGCCGAGGAAAAAGAAGTCGAAACAAACGTTGAACCAGAACTACTGGAGACAGGAAAACCGATTGAAATTGGCCAAGCAACTGAAATCGAGGAGCCACCAGAAAAACAACCGGTGGAATCGGAGTCACAACCAGCGGAAGTTGAGATGGAACAAATTGAAACAAAGGAAGAACCAACTAAACCAAAGGAAGAACTAGCAGGAATTGATGAGAAATGTAAGTTTTAGTAACAATTATTTATTTTATGATTCCTTTTGAGCTGTTTCGGTATGCTTCGATGCAAGTCAAATTTGTGGATGTTTTAAATCCATCATTCAATTATGTTTGCAATGTAGTCATGAAGTTTGTTCGTTTGTTCAACACAGATATGGGACATATACGCATGAGCAGACCTTTTTTGTGCACAGATCTTCGTTCAGGTTAATCGTGCATGGCAACAAAAATTGGTTCATTCCGTTTTATGTCGTGCAGTCTTATTTTGCTTGAATTAAGCATTGCTGTAAAGTTCAAGTTAAAGGTACAGTCCATCATTTGTAATTTGTGCATTTTGTTCGTTTGAAGAAACAAACATGCTCAAAATCTAAAGAAGGCTGCTGAACAACTAACCT---------------------------------------------------------------------------------------------------------------------------------------------------------------------------------------------------------------------------------------------------------------------------------------GATGAAGTTTCAGCTCAGCTTCAGCTGTATAAATTATTTCGAGAAAAACAAAATTCTAGGATCTCCATTTCAAATAAAAAGTTGCTGAATCTTGTTTTGAATTGGGCCTGAATTCAGCGTCCGAGGCACGTTCTCATCACTGAGCCGATTGCAGCTACGCGATGTCGCGCACGAATTACAGCAATCGAGGGCGCGTGTGCGTTGTGTCAACGCTTGCGCGCGCTTCGCCAAATAAAAAAAAAAGAACTAAACGGAAATTTCTTTAGACGCATGTCTTTAGACGCACCGTTGGAAAGGCCTACTTTCGTTGCTTTCTTCAAAATGACAGCATGCCAGAAAGAAAAATCTCTTATCTTTCAACGAAGTATGTTTTTTAACAATAGGCCTAATTACGTCAGTTACTTTTTGGATAGTTGCAAATAATGATGGACTGTAGCTTTAAGAAATTTGTGGAACTAAAGTTGTGCTTGGCAAGTTTTACTTTAGAATTCGAAAATGCATCGACACCAAAAAAAAGTGTAATTTTGGTCCTATAGTTGAAGTGTCAAACTAGAACAAATTTAATGATAAATTAAATAAATTTAAATAATTTATTCCTACCTTGCTTTGACAGTGATGAAGGAGCTGCGTGACCTTTTGGAATCTACGAAGATTGACCTTCCTGTTGACATCAATGATCCATACGACCTAGGTCTTCTTCTCAGACATTTACGTCACCATTCAAATCTTCTTGCTCGTATTGGAGACCCCGATGTCAAAAAGGAAGTCCTCAGCGCCATGAATGAAAAC

>BA62-B

GCACGACGTGCCGCAGAGGAAAAACCTCAAAAGGGAAAGAAGGGACGAAAAGGAAAGAAACGACGAAAGGGAAAGAAGGGACGAAAAGGAAAGAAGGGACGAAAAGGAAAGAAACGACGAAAGGGAAAGAAAAGACGAAAGGGAAAGAAAGGACGAAAGGGAAAGAAAGGACGAAAGGGAAAGAAAGGACGAAAGGGAAAGAAAGGACGAAAGGGAAAGAAAGGACGAAAGGGAAAGAAAGGACGAAAGGGAAAGAAAGGACGAAAGGGAAAGAAAGGAAAACCAATCCAACCTGAAGAAACACCAGCCATTCCGACGGAGATAAAAGCTGCAGAAATAGAAAAGGAACCAAAAACAGAAGTGGTTGTGGAACCAGTTATTCCGGAAGCAGATATTGCGGAGACCGAAATACAACAAATTAAAGCAGAAGTAGAACCAGTTGAAACAAAGCCAGAAATAGTTATGGAACCAGATGTGGAACCAGTTAATCCAGAAACGGAACTTACAGAGACCGGAAAAGAAGCGAAAGTAGAACAAGTTAAACCAGTGGAAGGGAAGCTAAACTTA------------------------------------------------------------------------------------------------------------------------------------------------------------------------------------------------------------------------------------------------------------------------------------------------------------------------------------------------------------------------------------------------------------------------------------------------------------------------------GGAAAAGGAAAGGGCAAAGGAAAGAAGGTTAGAAAGGGAAAGAAAGGACGAAAGGGAAAGAAAGGACGAAAGGGAAAGAAAGGACGGAAAGGAAAGAAGGGGCGAAAAGGAAAGAAAAGAAGAAAAGGAAAAAAAGGACGAAAGGGAAAGAAAGGACGAAAGGGGAAGAAAGGACGAAAGGGCAAGAAAGGACGAAAGGGAAAGAAAGGACGAAAGGGAAAGAAAGGACGAAAGGGAAAGAAAGGACGAAGGGGAAAGAAAGGA------------------CGAAAGGGCAAGAAAGGAAATCCAATCAAAACTGAAGAAACACCAGCCATTCTGACGGAGATAAAAGCTGCAGAATTAGAAAAGGAACCAAAAACAGAAATAGTTATGAAACCAGTTATTCCCGAAACGGAACTAACAGAGACCGGAAAAGAAGCAGAAGTAGAACAAGTTAAACCAGTGGAAAGGAAGCTAAAAATAGGAAAAGGAAAGGGCAAAGGAAAGAAG------------------AGGAGAAAGGGAAAGAAAGGACGAAAGGGAAAGAAAGGACGGAAAGGAAAGAAGGGGCGT------------------AAAGGAAAGAAAAGACGAAAAGGAAAGAAACGACGAAAGGGAAAGAAAGGACGAAAGGGAAAAAAAGGACGAAAGGGAAAGAAAGGACGAAAGGGAAAAAAAGGACGAAAGGGAAAAAAAGGACGAAAGGGAAAGAAAGGACGAAAGGGAAAGAAAGGAAAACCAATCCAAACTGAAGAAACACCAGCCATTCCGACGGAGATAAAAGCTGCAGAAATAGAAAAGGAACCAAAAACAGAAGTGGTTGTGGAACCACTTATTCAGGAAAAAGATGTTGCGGAGACCGAAATACAACCCATCGAAGCAGAAGTAGAACCAGTTGAACCAAAGACAGAAATAGTTATGGAACCAGTTATTCCGGAAGCAGATATTGCGGAGACCGAAATACAACCCATCAAAGCAGAAGAAGAACCAGTT------------------------------------------------------------------------------------------------------------GAACCAAAGCCAGAAATAGTTATGGAACCAGATGAGGAACCAGTTATTCCGGAAGCAGATATTGCGGAGACCGAAATACAACCCATCAAAGCAGAAGAAGAACCAGTTGAACCAAAGCCAGAAATAGTTATGGAACCAGATGAGGAACCAGTTATTCCGGAAGCAGATATTGCGGAGACCGAAATACAACCCATCAAAGCAGAAGAAGAACCAGTTGAACCAAAGCCAGAAATAGTTATGGAACCAGATGAGGAACCAGTTATTCCGGAAGCAGATATTGCGGAGACCGAAATACAACCCATCAGAGCAGAAGAAGAACCAGTTGAACCAAAGCCAGAAATAGTTATGGAACCAGATGAGGAACCAGTCATTCCAGAAACAGAACTAACAGAGACAGACAAACAACCAATCGAAGCAGAGGTAGAACCAGTTGAACCAAAAACAGAAGTGGTTGTGGAACCAGTTATTCCGGAAGCAGATATTGCGGAGACCGAAATACAACCCATCGAAGCAGAAGTAGAACCAGTTGAACCAAAGCCAGAAATAGTTATGGAACCAGATGTGGAACCAGTAATTCCAGAAACAGATCTTACAGAGACCGAAAAACAACCAATCGAAGCAGAAGTAGAACCAGTTGAACCGAAGACAGAGATAGTCGAACCAGAAACAGAGCCAGTCGAAGAAGCCGAGGAAGAAGCGGTTGAAGCACCCGTTATGGAACCAGTTATACCTGAGA------------CAGAACCGGAAGTTGAAGCCGAGGAAAAAGAAGTCGAAACAAACGTTGAACCAGAACTACTGGAGACAGGAAAACCGATTGAAATTGGCCAAGCAACTGAAATCGAGGAGCCACCAGAAAAACAACCGGTGGAATCGGAGTCACAACCAGCGGAAGTTGAGATGGAACAAATTGAAACAAAGGAAGAACCAACTGAACCAAAGGAAGAACTAGCAGGAATTGATGAGAAATGTAAGTTTTAGTAACAATTATTTATTTTATGATTCCTTTTGAGCTGTTTCGGTATGCTTCGATGCAAGTCAAATTTGTGGATGTTTTAAATCCATCATTCAATTATGTTTGCAATGTAGTCATGAAGTTTGTTCGTTTGTTCAACACAGATATGGGACATATACGCATGAGCAGACCTTTTTTGTGCACAGATCTTCGTTCAGGTTAATCGTGCATGGCAACAAAAATTGGTTCATTCCGTTTTATGTCGTGCAGTCTTATTTTGCTTGAATTAAGCATTGCTGTAAAGTTCAAGTTAAAGGTACAGTCCATCATTTGTAATTTGTGCATTTTGTTCGTTTGAAGAAACAAACATGCTCAAAATCTAAAGAAGGCTGCTGAACAACTAGCCT---------------------------------------------------------------------------------------------------------------------------------------------------------------------------------------------------------------------------------------------------------------------------------------GATGAAGTTTCAGCTCAGCTTCAGCTGTATAAATTATTTCGAGAAAAACAAAATTCTAGGGTCTCCATTTCAAATAAAAAGTTGCTGAATCTTGTTTTGAATTGGGCCTGAATTCAGCGTCCGAGGCACGTTCTCATCACTGAGCCGATTGCAGCTACGCGATGTCGCGCACGAATTAAAGCAATCGAGGGCGCGTGTGCGTTGTGTCAACGCTTGCGCGCGCTTCGCCAAAT-AAAAAAAAAGAACTAAACGGAAATTTCTTTAGACGCATGCCTTTAGACGCACCGTTGGAAAGGCCTACTTTCGTTGCTTTCTTCAAAATGACAGCATGCCAGAAAGAAAAATCTCTTATCTTTCAACGAAGTATGTTTTTTAACAATAGGCCTAATTACGTCAGTTACTTTTTGGATAGTTGCAAATAATGATGGACTGTAGCTTTAAGAAATTTGTGGAACTAAAGTTGTGCTTGGCAAGTTTTACTTTAGAATTCGAAAATGCATCGACACCAAAAAAAAGTGTAATTTTGGTCCTATAGTTGAAGTGTCAAACTAGAACAAATTTAATGATAAATTAAATAAATTTAAATAATTTATTCCTACCTTGCTTTGACAGTGATGAAGGAGCTGCGTGACCTTTTGGAATCTACGAAGATTGACCTTCCTGTTGACATCAATGATCCATACGACCTAGGTCTTCTTCTCAGACATTTACGTCACCATTCAAATCTTCTTGCTCGTATTGGAGACCCCGATGTCAAAAAGGAAGTCCTCAGCGCCATGAATGAAAAC

>BA85-A

GCACGACGTGCCGCAGAGGAAAAACCTCAAAAGGGAAAGAAGGGACGAAAAGGAAAGAAACGACGAAAGGGAAAGAAGGGACGAAAAGGAAAGAAGGGACGAAAAGGAAAGAAACGACGAAAGGGAAAGAAAAGACGAAAGGGAAAGAAAGGACGAAAGGGAAAGAAAGGACGAAAGGGAAAGAAAGGACGAAAGGGAAAGAAAGGACGAAAGGGAAAGAAAGGACGAAAGGGAAAGAAAGGACGAAAGGGAAAGAAAGGACGAAAGGGAAAGAAAGGAAAACCAATCCAACCTGAAGAAACACCAGCCATTCCGACGGAGATAAAAGCTGCAGAAATAGAAAAGGAACCAAAAACAGAAGTGGTTGTGGAACCAGTTATTCCGGAAGCAGATATTGCGGAGACCGAAATACAACAAATTAAAGCAGAAGTAGAACCAGTTGAAACAAAGCCAGAAATAGTTATGGAACCAGATGTGGAACCAGTTATTCCAGAAACGGAACTTACAGAGACCGGAAAAGAAGCGAAAGTAGAACAAGTTAAACCAGTGGAAGGGAAGCTAAACTTA------------------------------------------------------------------------------------------------------------------------------------------------------------------------------------------------------------------------------------------------------------------------------------------------------------------------------------------------------------------------------------------------------------------------------------------------------------------------------GGAAAAGGAAAGGGCAAAGGAAAGAAGGTTAGAAAGGGAAAGAAAGGACGAAAGGGAAAGAAAGGA------------------CGGAAAGGAAAGAAGGGGCGAAAAGGAAAGAAAAGAAGAAAAGGAAAAAAAGGACGAAAGGGAAAGAAAGGACGAAAGGGAAAGAAAGGACGAAAGGGCAAGAAAGGACGAAAGGGAAAGAAAGGACGAAAGGGAAAGAAAGGACGAAAGGGAAAGAAAGGACGAANGGGAAAGAAAGGA------------------CGAAAGGGCAAGAAAGGAAATCCAATCAAAACTGAAGAAACACCAGCCATTCTGACGGAGATAAAAGCTGCAGAATTAGAAAAGGAACCAAAAACAGAAATAGTTATGAAACCAGTTATTCCCGAAACGGAACTAACAGAGACCGGAAAAGAAGCAGAAGTAGAACAAGTTAAACCAGTGGAAAGGAAGCTAAAAATAGGAAAAGGAAAGGGCAAAGGAAAGAAG------------------AGGAGAAAGGGAAAGAAAGGACGAAAGGGAAAGAAAGGACGGAAAGGAAAGAAGGGGCGT------------------AAAGGAAAGAAAAGACGAAAAGGAAAGAAACGACGAAAGGGAAAGAAAGGACGAAAGGGAAAAAAAGGACGAAAGGGAAAGAAAGGACGAAAGGGAAAAAAAGGACGAAAGGGAAAAAAAGGACGAAAGGGAAAGAAAGGACGAAAGGGAAAGAAAGGAAAACCAATCCAAACTGAAGAAACACCAGCCATTCCGACGGAGATAAAAGCTGCAGAAATAGAAAAGGAACCAAAAACAGAAGTGGTTGTGGAACCACTTTTTCAGGAAAAAGATGTTGCGGAGACCGAAATACAACCCATCGAAGCAGAAGTAGAACCAGTTGAACCAAAGACAGAAATAGTTATGGAACCAGTTATTCCGGAAGCAGATATTGCGGAGACCGAAATACAACCCATCAAAGCAGAAGAAGAACCAGTT------------------------------------------------------------------------------------------------------------GAACCAAAGCCAGAAATAGTTATGGAACCAGATGAGGAACCAGTTATTCCGGAAGCAGATATTGCGGAGACCGAAATACAACCCATCAAAGCAGAAGAAGAACCAGTTGAACCAAAGCCAGAAATAGTTATGGAACCAGATGAGGAACCAGTTATTCCGGAAGCAGATATTGCGGAGACCGAAATACAACCCATCAAAGCAGAAGAAGAACCAGTTGAACCAAAGCCAGAAATAGTTATGGAACCAGATGAGGAACCAGTTATTCCGGAAGCAGATATTGCGGAGACCGAAATACAACCCATCAGAGCAGAAGAAGAACCAGTTGAACCAAAGCCAGAAATAGTTATGGAACCAGATGAGGAACCAGTCATTCCAGAAACAGAACTAACAGAGACAGACAAACAACCAATCGAAGCAGAAGTAGAACCAGTTGAACCAAAAACAGAAGTGGTTGTGGAACCAGTTATTCCGGAAGCAGATATTGCGGAGACCGAAATACAACCCATCGAAGCAGAAGTAGAACCAGTTGAACCAAAGCCAGAAATAGTTATGGAACCAGATGTGGAACCAGTAATTCCAGAAACAGATCTTACAGAGACCGAAAAACAACCAATCGAAGCAGAAGTAGAACCAGTTGAACCGAAGACAGAGATAGTCGAACCAGAAACAGAGCCAGTCGAAGAAGCCGAGGAAGAAGCGGTTGAAGCACCCGTTATGGAACCAGTTATACCTGAGATAGAACCAAAGACAGAACCGGAAGTTGAAGCCGAGGAAAAAGAAGTCGAAACAAACGTTGAACCAGAACTACTGGAGACAGGAAAACCGATTGAAATTGGCCAAGCAACTGAAATCGAGGAGCCACCAGAAAAACAACCGGTGGAATCGGAGTCACAACCAGCGGAAGTTGAGATGGAACAAATTGAAACAAAGGAAGAACCAACTAAACCAAAGGAAGAACTAGCAGGAATTGATGAGAAATGTAAGTTTTAGTAACAATTATTTATTTTATGATTCCTTTTGAGCTGTTTCGGTATGCTTCGATGCAAGTCAAATTTGTGGATGTTTTAAATCCATCATTCAATTATGTTTGCAATGTAGTCATGAAGTTTGTTCGTTTGTTCAACACAGATATGGGACATATACGCATGAGCAGACCTTTTTTGTGCACAGATCTTCGTTCAGGTTAATCGTGCATGGCAACAAAAATTGGTTCATTCCGTTTTATGTCGTGCAGTCTTATTTTGCTTGAATTAAGCATTGCTGTAAAGTTCAAGTTAAAGGTACAGTCCATCATTTGTAATTTGTGCATTTTGTTCGTTTGAAGAAACAAACATGCTCAAAATCTAAAGAAGGCTGCTGAACAACTAACCT---------------------------------------------------------------------------------------------------------------------------------------------------------------------------------------------------------------------------------------------------------------------------------------GATGAAGTTTCAGCTCAGCTTCAGCTGTATAAATTATTTCGAGAAAAACAAAATTCTAGGATCTCCATTTCAAATCAAAAGTTGCTGAATCTTGTTTTGAATTGGGCCTGAATTCAGCGTCCGAGGCACGTTCTCATCACTGAGCCGATTGCAGCTACGCGATGTCGCGCACGAATTAAAGCAATCGAGGGCGCGTGTGCGTTGTGTCAACGCTTGCGCGCGCTTCGCCAAATAAAAAAAAAAGAACTAAACGGAAATTTCTTTAGACGCATGTCTTTAGACGCACCGTTGGAAAGGCCTACTTTCGTTGCTTTCTTCAAAATGACAGCATGCCAGAAAGAAAAATCTCTTATCTTTCAACGAAGTATGTTTTTTAACAATAGGCCTAATTACGTCAGTTACTTTTTGGATAGTTGCAAATAATGATGGACTGTAGCTTTAAGAAATTTGTGGAACTAAAGTTGTGCTTGGCAAGTTTTACTTTAGAATTCGAAAATGCATCGACACCAAAAAAA-GTGTAATTTTGGTCCTATAGTTGAAGTGTCAAACTAGAACAAATTTAATGATAAATTAAATAAATTTAAATAATTTATTCCTACCTTGCTTTGACAGTGATGAAGGAGCTGCGTGACCTTTTGGAATCTACGAAGATTGACCTTCCTGTTGACATCAATGATCCATACGACCTAGGTCTTCTTCTCAGACATTTACGTCACCATTCAAATCTTCTTGCTCGTATTGGAGACCCCGATGTCAAAAAGGAAGTCCTCAGCGCCATGAATGAAAAC

>BA76-B

GCACGACGTGCCGCAGAGGAAAAACCTCAAAAGGGAAAGAAGGGACGAAAAGGAAAGAAACGACGAAAGGGAAAGAAGGGACGAAAAGGAAAGAAGGGACGAAAAGGAAAGAAACGACGAAAGGGAAAGAAAAGACGAAAGGGAAAGAAAGGACGAAAGGGAAAGAAAGGACGAAAGGGAAAGAAAGGACGAAAGGGAAAGAAAGGACGAAAGGGAAAGAAAGGACGAAAGGGAAAGAAAGGACGAAAGGGAAAGAAAGGACGAAAGGGAAAGAAAGGAAAACCAATCCAACCTGAAGAAACACCAGCCATTCCGACGGAGATAAAAGCTGCAGAAATAGAAAAGGAACCAAAAACAGAAGTGGTTGTGGAACCAGTTATTCCGGAAGCAGATATTGCGGAGACCGAAATACAACAAATTAAAGCAGAAGTAGAACCAGTTGAAACAAAGCCAGAAATAGTTATGGAACCAGATGTGGAACCAGTTATTCCAGAAACGGAACTTACAGAGACCGGAAAAGAAGCGAAAGCAGAACAAGTTAAACCAGTGGAAGGGAAGCTAAACTTA------------------------------------------------------------------------------------------------------------------------------------------------------------------------------------------------------------------------------------------------------------------------------------------------------------------------------------------------------------------------------------------------------------------------------------------------------------------------------GGAAAAGGAAAGGGCAAAGGAAAGAAGGTTAGAAAGGGAAAGAAAGGACGAAAGGGAAAGAAAGGACGAAAGGGAAAGAAAGGACGGAAAGGAAAGAAGGGGCGAAAAGGAAAGAAAAGAAGAAAAGGAAAAAAAGGACGAAAGGGAAAGAAAGGACGAAAGGGAAAGAAAGGACGAAAGGGCAAGAAAGGACGAAAGGGAAAGAAAGGACGAAAGGGAAAGAAAGGACGAAAGGGAAAGAAAGGACGAAGGGGAAAGAAAGGA------------------CGAAAGGGCAAGAAAGGAAATCCAATCAAAACTGAAGAAACACCAGCCATTCTGACGGAGATAAAAGCTGCAGAATTAGAAAAGGAACCAAAAACAGAAATAGTTATGAAACCAGTTATTCCCGAAACGGAACTAACAGAGACCGGAAAAGAAGCAGAAGTAGAACAAGTTAAACCAGTGGAAAGGAAGCTAAAAATAGGAAAAGGAAAGGGCAAAGGAAAGAAG------------------AGGAGAAAGGGAAAGAAAGGACGAAAGGGAAAGAAAGGACGGAAAGGAAAGAAGGGGCGT------------------AAAGGAAAGAAAAGACGAAAAGGAAAGAAACGACGAAAGGGAAAGAAAGGACGAAAGGGAAAAAAAGGACGAAAGGGAAAGAAAGGACGAAAGGGAAAAAAAGGACGAAAGGGAAAAAAAGGACGAAAGGGAAAGAAAGGACGAAAGGGAAAGAAAGGAAAACCAATCCAAACTGAAGAAACACCAGCCATTCCGACGGAGATAAAAGCTGCAGAAATAGAAAAGGAACCAAAAACAGAAGTGGTTGTGGAACCACTTATTCAGGAAAAAGATGTTGCGGAGACCGAAATACAACCCATCGAAGCAGAAGTAGAACCAGTTGAACCAAAGACAGAAATAGTTATGGAACCAGTTATTCCGGAAGCAGATATTGCGGAGACCGAAATACAACCCATCAAAGCAGAAGAAGAACCAGTT------------------------------------------------------------------------------------------------------------------------------------------------------------------------------------------------------------------------GAACCAAAGCCAGAAATAGTTATGGAACCAGATGAGGAACCAGTTATTCCGGAAGCAGATATTGCGGAGACCGAAATACAACCCATCAAAGCAGAAGAAGAACCAGTTGAACCAAAGCCAGAAATAGTTATGGAACCAGATGAGGAACCAGTTATTCCGGAAGCAGATATTGCGGAGACCGAAATACAACCCATCAGAGCAGAAGAAGAACCAGTTGAACCAAAGCCAGAAATAGTTATGGAACCAGATGAGGAACCAGTCATTCCAGAAACAGAACTAACAGAGACAGACAAACAACCAATCGAAGCAGAAGTAGAACCAGTTGAACCAAAAACAGAAGTGGTTGTGGAACCAGTTATTCCGGAAGCAGATATTGCGGAGACCGAAATACAACCCATCGAAGCAGAAGTAGAACCAGTTGAACCAAAGCCAGAAATAGTTATGGAACCAGATGTGGAACCAGTAATTCCAGAAACAGATCTTACAGAGACCGAAAAACAACCAATCGAAGCAGAAGTAGAACCAGTTGAACCGAAGACAGAGATAGTCGAACCAGAAACAGAGCCAGTCGAAGAAGCCGAGGAAGAAGCGGTTGAAGCACCCGTTATGGAACCAGTTATACCTGAGATAGAACCAAAGACAGAACCGGAAGTTGAAGCCGAGGAAAAAGAAGTCGAAACAAACGTTGAACCAGAACT---------------------------TGGCCAAGCAACTGAAATCGAGGAGCCACCAGAAAAACAACCGGTGGAATCGGAGTCACAACCAGCGGAAGTTGAGATGGAACAAATTGAAACAAAGGAAGAACCAACTGAACCAAAGGAAGAACTAGCAGGAATTGATGAGAAATGTAAGTTTTAGTAACAATTATTTATTTTATGATTCCTTTTGAGCTGTTTCGGTATGCTTCGATGCAAGTCAAATTTGTGGATGTTTTAAATCCATCATTCAATTATGTTTGCAATGTAGTCACGAAGTTTGTTCGTTTGTTCAACACAGATATGGGACATATACGCATGAGCAGACCTTTTTTGTGCACAGATCTTCGTTCAGGTTAATCGTGCATGGTAACAAAAATTGGTTCATTCCGTTTTATGTCGTGCAGTCTTATTTTGCTTGAATTAAGCATTGCTGTAAAGTTCAAGTTAAAGGTACAGTCCATCATTTGTAATTTGTGCATTTTGTTCGTTTGAAGAAACAAACATGCTCAAAATCTAAAGAAGGCTGCTGAACAACTAACCT---------------------------------------------------------------------------------------------------------------------------------------------------------------------------------------------------------------------------------------------------------------------------------------GATGAAGTTTCAGCTCAGCTTCAGCTGTATAAATTATTTCGAGAAAAACAAAATTCTAGGATCTCCATTTCAAATCAAAAGTTGCTGAATCTTGTTTTGAATTGGGCCTGAATTCAGCGTCCGAGGCACGTTCTCATCACTGAGCCGATTGCAGCTACGCGATGTCGCGCACGAATTAAAGCAATCGAGGGCGCGTGTGCGTTGTGTCAACGCTTGCGCGCGCTTCGCCAAATAAAAAAAAAAGAACTAAACGGAAATTTCTTTAGACGCATGTCTTTAGACGCACCGTTGGAAAGGCCTACTTTCGTTGCTTTCTTCAAAATGACAGCATGCCAGAAAGAAAAATCTCTTATCTTTCAACGAAGTATGTTTTTTAACAATAGGCCTAATTACGTCAGTTACTTTTTGGATAGTTGCAAATAATGATGGACTGTAGCTTTAAGAAATTTGTGGAACTAAAGTTGTGCTTGGCAAGTTTTACTTTAGAATTCGAAAATGCATCGACACCAAAAAAAAGTGTAATTTTGGTCCTATAGTTGAAGTGTCAAACTAGAACAAATTTAATGATAAATTAAATAAATTTAAATAATTTATTCCTACCTTGCTTTGACAGTGATGAAGGAGCTGCGTGACCTTTTGGAATCTACGAAGATTGACCTTCCTGTTGACATCAATGATCCATACGACCTAGGTCTTCTTCTCAGACATTTACGTCACCATTCAAATCTTCTTGCTCGTATTGGAGACCCCGATGTCAAAAAGGAAGTCCTCAGCGCCATGAATGAAAAC

>BA94-B

GCACGACGTGCCGCAGAGGAAAAACCTCAAAAGGGAAAGAAGGGACGAAAAGGAAAGAAACGACGAAAGGGAAAGAAGGGACGAAAAGGAAAGAAGGGACGAAAAGGAAAGAAACGACGAAAGGGAAAGAAAAGACGAAAGGGAAAGAAAGGACGAAAGGGAAAGAAAGGACGAAAGGGAAAGAAAGGACGAAAGGGAAAGAAAGGACGAAAGGGAAAGAAAGGACGAAAGGGAAAGAAAGGACGAAAGGGAAAGAAAGGACGAAAGGGAAAGAAAGGAAAACCAATCCAACCTGAAGAAACACCAGCCATTCCGACGGAGATAAAAGCTGCAGAAATAGAAAAGGAACCAAAAACAGAAGTGGTTGTGGAACCAGTTATTCCGGAAGCAGATATTGCGGAGACCGAAATACAACAAATTAAAGCAGAAGTAGAACCAGTTGAAACAAAGCCAGAAATAGTTATGGAACCAGATGTGGAACCAGTTATTCCAGAAACGGAACTTACAGAGACCGGAAAAGAAGCGAAAGTAGAACAAGTTAAACCAGTGGAAGGGAAGCTAAACTTA------------------------------------------------------------------------------------------------------------------------------------------------------------------------------------------------------------------------------------------------------------------------------------------------------------------------------------------------------------------------------------------------------------------------------------------------------------------------------GGAAAAGGAAAGGGCAAAGGAAAGAAGGTTAGAAAGGGAAAGAAAGGACGAAAGGGAAAGAAAGGACGAAAGGGAAAGAAAGGACGGAAAGGAAAGAAGGGGCGAAAAGGAAAGAAAAGAAGAAAAGGAAAAAAAAGACGAAAGGGAAAGAAAGGACGAAAGGGAAAGAAAGGACGAAAGGGCAAGAAAGGACGAAAGGGAAAGAAAGGACGAAAGGGAAAGAAAGGACGAAAGGGAAAGAAAGGACGAAAGGGAAAGAAAGGA------------------CGAAAGGGCAAGAAAGGAAATCCAATCAAAACTGAAGAAACACCAGCCATTCTGACGGAGATAAAAGCTGCAGAATTAGAAAAGGAACCAAAAACAGAAATAGTTATGAAACCAGTTATTCCCGAAACGGAACTAACAGAGACCGGAAAAGAAGCAGAAGTAGAACAAGTTAAACCAGTGGAAAGGAAGCTAAAAATAGGAAAAGGAAAGGGCAAAGGAAAGAAG------------------AGGAGAAAGGGAAAGAAAGGACGAAAGGGAAAGAAAGGACGGAAAGGAAAGAAGGGGCGT------------------AAAGGAAAGAAAAGACGAAAAGGAAAGAAACGA------------------CGAAAGGGAAAAAAAGGACGAAAGGGAAAGAAAGGACGAAAGGGAAAAAAAGGACGAAAGGGAAAAAAAGGACGAAAGGGAAAGAAAGGACGAAAGGGAAAGAAAGGAAAACCAATCCAAACTGAAGAAACACCAGCCATTCCGACGGAGATAAAAGCTGCAGAAATAGAAAAGGAACCAAAAACAGAAGTGGTTGTGGAACCACTTATTCAGGAAAAAGATGTTGCGGAGACCGAAATACAACCCATCGAAGCAGAAGTAGAACCAGTTGAACCAAAGACAGAAATAGTTATGGAACCAGTTATTCCGGAAGCAGATATTGCGGAGACCGAAATACAACCCATCAAAGCAGAAGAAGAACCAGTT------------------------------------------------------------------------------------------------------------------------------------------------------------------------------------------------------------------------GAACCAAAGCCAGAAATAGTTATGGAACCAGATGAGGAACCAGTTATTCCGGAAGCAGATATTGCGGAGACCGAAATACAACCCATCAAAGCAGAAGAAGAACCAGTTGAACCAAAGCCAGAAATAGTTATGGAACCAGATGAGGAACCAGTTATTCCGGAAGCAGATATTGCGGAGACCGAAATACAACCCATCAGAGCAGAAGAAGAACCAGTTGAACCAAAGCCAGAAATAGTTATGGAACCAGATGAGGAACCAGTCATTCCAGAAACAGAACTAACAGAGACAGACAAACAACCAATCGAAGCAGAAGTAGAACCAGTTGAACCAAAAACAGAAGTGGTTGTGGAACCAGTTATTCCGGAAGCAGATATTGCGGAGACCGAAATACAACCCATCGAAGCAGAAGTAGAACCAGTTGAACCAAAGCCAGAAATAGTTATGGAACCAGATGTGGAACCAGTAATTCCAGAAACAGATCTTACAGAGACCGAAAAACAACCAATCGAAGCAGAAGTAGAACCAGTTGAACCGAAGACAGAGATAGTCGAACCAGAAACAGAGCCAGTCGAAGAAGCCGAGGAAGAAGCGGTTGAAGCACCCGTTATGGAACCAGTTATACCTGAGATAGAACCAAAGACAGAACCGGAAGTTGAAGCCGAGGAAAAAGAAGTCGAAACAAACGTTGAACCAGAACTACTGGAGACAGGAAAACCGATTGAAATTGGCCAAGCAACTGAAATCGAGGAGCCACCAGAAAAACAACCGGTGGAATCGGAGTCACAACCAGCGGAAGTTGAGATGGAACAAATTGAAACAAAGGAAGAACCAACTGAACCAAAGGAAGAACTAGCAGGAATTGATGAGAAATGTAAGTTTTAGTAACAATTATTTATTTTATGATTCCTTTTGAGCTGTTTCGGTATGCTTCGATGCAAGTCAAATTTGTGGATGTTTTAAATCCATCATTCAATTATGTTTGCAATGTAGTCATGAAGTTTGTTCGTTTGTTCAACACAGATATGGGACATATACGCATGAGCAGACCTTTTTTGTGCACAGATCTTCGTTCAGGTTAATCGTGCATGGCAACAAAAATTGGTTCATTCCGTTTTATGTCGTGCAGTCTTATTTTGCTTGAATTAAGCATTGCTGTAAAGTTCAAGTTAAAGGTACAGTCCATCATTTGTAATTTGTGCATTTTGTTCGTTTGAAGAAACAAACATGCTCAAAATCTAAAGAAGGCTGCTGAACAACTAACCT---------------------------------------------------------------------------------------------------------------------------------------------------------------------------------------------------------------------------------------------------------------------------------------GATGAAGTTTCAGCTCAGCTTCAGCTGTATAAATTATTTCGAGAAAAACAAAATTCTAGGATCTCCGTTTCAAATAAAAAGTTGCTGAATCTTGTTTTGAATTGGGCCTGAATTCAGCGTCCGAGGCACGTTCTCATCACTGAGCCGATTGCAGCTACGCGATGTCGCGCACGAATTAAAGCAATCGAGGGCGCGTGTGCGTTGTGTCAACGCTTGCGCGCGCTTCGCCAAATAAAAAAAA--GAACTAAACGGAAATTTCTTTAGACGCATGTCTTTAGACGCACCGTTGGAAAGGCCTACTTTCGTTGCTTTCTTCAAAATGACAGCATGCCAGAAAGAAAAATCTCTTATCTTTCAACGAAGTATGTTTTTTAACAATAGGCCTAATTACGTCAGTTACTTTTTGGATAGTTGCAAATAATGATGGACTGTAGCTTTAAGAAATTTGTGGAACTAAAGTTGTGCTTGGCAAGTTTTACTTTAGAATTCGAAAATGCATCGNCACCAAAAAAAAGTGTAATTTTGGTCCTATAGTTGAAGTGTCAAACTAGAACAAATTTAATGATAAATTAAATAAATTTAAATAATTTATTCCTACCTTGCTTTGACAGTGATGAAGGAGCTGCGTGACCTTTTGGAATCTACGAAGATTGACCTTCCTGTTGACATCAATGATCCATACGACCTAGGTCTTCTTCTCAGACATTTACGTCACCATTCAAATCTTCTTGCTCGTATTGGAGACCCCGATGTCAAAAAGGAAGTCCTCAGCGCCATGAATGAAAAC

>BA71-A

GCACGACGTGCCGCAGAGGAAAAACCTCAAAAGGGAAAGAAGGGACGAAAAGGAAAGAAACGACGAAAGGGAAAGAAGGGACGAAAAGGAAAGAAGGGACGAAAAGGAAAGAAACGACGAAAGGGAAAGAAAAGACGAAAGGGAAAGAAAGGACGAAAGGGAAAGAAAGGACGAAAGGGAAAGAAAGGACGAAAGGGAAAGAAAGGACGAAAGGGAAAGAAAGGACGAAAGGGAAAGAAAGGACGAAAGGGAAAGAAAGGACGAAAGGGAAAGAAAGGAAAACCAATCCAACCTGAAGAAACACCAGCCATTCCGACGGAGATAAAAGCTGCAGAAATAGAAAAGGAACCAAAAACAGAAGTGGTTGTGGAACCAGTTATTCCGGAAGCAGATATTGCGGAGACCGAAATACAACAAATTAAAGCAGAAGTAGAACCAGTTGAAACAAAGCCAGAAATAGTTATGGAACCAGATGTGGAACCAGTTATTCCAGAAACGGAACTTACAGAGACCGGAAAAGAAGCGAAAGTAGAACAAGTTAAACCAGTGGAAGGGAAGCTAAACTTA------------------------------------------------------------------------------------------------------------------------------------------------------------------------------------------------------------------------------------------------------------------------------------------------------------------------------------------------------------------------------------------------------------------------------------------------------------------------------GGAAAAGGAAAGGGCAAAGGAAAGAAGGTTAGAAAGGGAAAGAAAGGACGAAAGGGAAAGAAAGGACGAAAGGGAAAGAAAGGACGGAAAGGAAAGAAGGGGCGAAAAGGAAAGAAAAGAAGAAAAGGAAAAAAAGGACGAAAGGGAAAGAAAGGACGAAAGGGAAAGAAAGGACGAAAGGGCAAGAAAGGACGAAAGGGAAAGAAAGGACGAAAGGGAAAGAAAGGACGAAAGGGAAAGAAAGGACGAAGGGGAAAGAAAGGA------------------CGAAAGGGCAAGAAAGGAAATCCAATCAAAACTGAAGAAACACCAGCCATTCTGACGGAGATAAAAGCTGCAGAATTAGAAAAGGAACCAAAAACAGAAATAGTTATGAAACCAGTTATTCCCGAAACGGAACTAACAGAGACCGGAAAAGAAGCAGAAGTAGAACAAGTTAAACCAGTGGAAAGGAAGCTAAAAATAGGAAAAGGAAAGGGCAAAGGAAAGAAG------------------AGGAGAAAGGGAAAGAAGGGACGAAAGGGAAAGAAAGGACGGAAAGGAAAGAAGGGGCGT------------------AAAGGAAAGAAAAGACGAAAAGGAAAGAAACGACGAAAGGGAAAGAAAGGACGAAAGGGAAAAAAAGGACGAAAGGGAAAGAAAGGACGAAAGGGAAAAAAAGGACGAAAGGGAAAAAAAGGACGAAAGGGAAAGAAAGGACGAAAGGGAAAGAAAGGAAAACCAATCCAAACTGAAGAAACACCAGCCATTCCGACGGAGATAAAAGCTGCAGAAATAGAAAAGGAACCAAAAACAGAAGTGGTTGTGGAACCACTTATTCAGGAAAAAGATGTTGCGGAGACCGAAATACAACCCATCGAAGCAGAAGTAGAACCAGTTGAACCAAAGACAGAAATAGTTATGGAACCAGTTATTCCGGAAGCAGATATTGCGGAGACCGAAATACAACCCATCAAAGCAGAAGAAGAACCAGTT------------------------------------------------------------------------------------------------------------GAACCAAAGCCAGAAATAGTTATGGAACCAGATGAGGAACCAGTTATTCCGGAAGCAGATATTGCGGAGACCGAAATACAACCCATCAAAGCAGAAGAAGAACCAGTTGAACCAAAGCCAGAAATAGTTATGGAACCAGATGAGGAACCAGTTATTCCGGAAGCAGATATTGCGGAGACCGAAATACAACCCATCAAAGCAGAAGAAGAACCAGTTGAACCAAAGCCAGAAATAGTTATGGAACCAGATGAGGAACCAGTTATTCCGGAAGCAGATATTGCGGAGACCGAAATACAACCCATCAGAGCAGAAGAAGAACCAGTTGAACCAAAGCCAGAAATAGTTATGGAACCAGATGAGGAACCAGTCATTCCAGAAACAGAACTAACAGAGACAGACAAACAACCAATCGAAGCAGAAGTAGAACCAGTTGAACCAAAAACAGAAGTGGTTGTGGAACCAGTTATTCCGGAAGCAGATATTGCGGAGACCGAAATACAACCCATNGAAGCAGAAGTAGAACCAGTTGAACCAAAGCCAGAAATAGTTATGGAACCAGATGTGGAACCAGTAATTCCAGAAACAGATCTTACAGAGACCGAAAAACAACCAATCGAAGCAGAAGTAGAACCAGTTGAACCGAAGACAGAGATAGTCGAACCAGAAACAGAGCCAGTCGAAGAAGCCGAGGAAGAAGCGGTTGAAGCACCCGTTATGGAACCAGTTATACCTGAGATAGAACCAAAGACAGAACCGGAAGTTGAAGCCGAGGAAAAAGAAGTCGAAACAAACGTTGAACCAGAACTACTGGAGACAGGAAAACCGTTTGAAATTGGCCAAGCAACTGAAATCGAGGAGCCACCAGAAAAACAACCGGTGGAATCGGAGTCACAACCAGCGGAAGTTGAGATGGAACAAATTGAAACAAAGGAAGAACCAACTGAACCAAAGGAAGAACTAGCAGGAATTGATGAGAAATGTAAGTTTTAGTAACAATTATTTATTTTATGATTCCTTTTGAGCTGTTTCGGTATGCTTCGATGCAAGTCAAATTTGTGGATGTTTTAAATCCATCATTCAATTATGTTTGCAATGTAGTCATGAAGTTTGTTCGTTTGTTCAACACAGATATGGGACATATACGCATGAGCAGACCTTTTTTGTGCACAGATCTTCGTTCAGGTTAATCGTGCATGGCAACAAAAATTGGTTCATTCCGTTTTATGTCGTGCAGTCTTATTTTGCTTGAATTAAGCATTGCTGTAAAGTTCAAGTTAAAGGTACAGTCCATCATTTGTAATTTGTGCATTTTGTTCGTTTGAAGAAACAAACATGCTCAAAATCTAAAGAAGGCTGCTGAACAACTAACCT---------------------------------------------------------------------------------------------------------------------------------------------------------------------------------------------------------------------------------------------------------------------------------------GATGAAGTTTCAGCTCAGCTTCAGCTGTATAAATTATTTCGAGAAAAACAAAATTCTAGGATCTCCGTTTCAAATAAAAAGTTGCTGAATCTTGTTTTGAATTGGGCCTGAATTCAGCGTCCGAGGCACGTTCTCATCACTGAGCCGATTGCAGCTACGCGATGTCGCGCACGAATTAAAGCAATCGAGGGCGCGTGTGCGTTGTGTCAACGCTTGCGCGCGCTTCGCCAAATAAAAAAAAAAGAACTAAACGGAAATTTCTTTAGACGCATGTCTTTAGACGCACCGTTGGAAAGGCCTACTTTCGTTGCTTTCTTCAAAATGACAGCATACCAGAAAGAAAAATCTCTTATCTTTCAACGAAGTATGTTTTTTAACAATAGGCCTAATTACGTCAGTTACTTTTTGGATAGTTGCAAATAATGATGGACTGTAGCTTTAAGAAATTTGTGGAACTAAAGTTGTGCTTGGCAAGTTTTACTTTAGAATTCGAAAATGCATCGACACCAAAAAAAAGTGTAATTTTGGTCCTATAGTTGAAGTGTCAAACTAGAACAAATTTAATGATAAATTAAATAAATTTAAATAATTTATTCCTACCTTGCTTTGACAGTGATGAAGGAGCTGCGTGACCTTTTGGAATCTACGAAGATTGACCTTCCTGTTGACATCAATGATCCATACGACCTAGGTCTTCTTCTCAGACATTTACGTCACCATTCAAATCTTCTTGCTCGTATTGGAGACCCCGATGTCAAAAAGGAAGTCCTCAGCGCCATGAATGAAAAC

>BA40-B

GCACGACGTGCCGCAGAGGAAAAACCTCAAAAGGGAAAGAAGGGACGAAAAGGAAAGAAACGACGAAAGGGAAAGAAGGGACGAAAAGGAAAGAAGGGACGAAAAGGAAAGAAACGACGAAAGGGAAAGAAAAGACGAAAGGGAAAGAAAGGACGAAAGGGAAAGAAAGGACGAAAGGGAAAGAAAGGACGAAAGGGAAAGAAAGGACGAAAGGGAAAGAAAGGACGAAAGGGAAAGAAAGGACGAAAGGGAAAGAAAGGACGAAAGGGAAAGAAAGGAAAACCAATCCAACCTGAAGAAACACCAGCCATTCCGACGGAGATAAAAGCTGCAGAAATAGAAAAGGAACCAAAAACAGAAGTGGTTGTGGAACCAGTTATTCCGGAAGCAGATATTGCGGAGACCGAAATACAACAAATTAAAGCAGAAGTAGAACCAGTTGAAACAAAGCCAGAAATAGTTATGGAACCAGATGTGGAACCAGTTATTCCAGAAACGGAACTTACAGAGACCGGAAAAGAAGCGAAAGTAGAACAAGTTAAACCAGTGGAAGGGAAGCTAAACTTA------------------------------------------------------------------------------------------------------------------------------------------------------------------------------------------------------------------------------------------------------------------------------------------------------------------------------------------------------------------------------------------------------------------------------------------------------------------------------GGAAAAGGAAAGGGCAAAGGAAAGAAGGTTAGAAAGGGAAAGAAAGGACGAAAGGGAAAGAAAGGACGAAAGGGAAAGAAAGGACGGAAAGGAAAGAAGGGGCGAAAAGGAAAGAAAAGAAGAAAAGGAAAAAAAGGACGAAAGGGAAAGAAAGGACGAAAGGGAAAGAAAGGACGAAAGGGCAAGAAAGGACGAAAGGGAAAGAAAGGACGAAAGGGAAAGAAAGGACGAAAGGGAAAGAAAGGACGAAGGGGAAAGAAAGGA------------------CGAAAGGGCAAGAAAGGAAATCCAATCAAAACTGAAGAAACACCAGCCATTCTG------------------------------------------------------------------------------------------------------------------------------------------------------------------------------------------------------------------------------------------------------------------------------------------------------------------------------------------------------------------------------------------------------------------------------------------------------------------------------ACGGAGATAAAAGCTGCAGAAATAGAAAAGGAACCAAAAACAGAAGTGGTTGTGGAACCACTTATTCAGGAAAAAGATGTTGCGGAGACCGAAATACAACCCATCGAAGCAGAAGTAGAACCAGTTGAACCAAAGACAGAAATAGTTATGGAACCAGTTATTCCGGAAGCAGATATTGCGGAGACCGAAATACAACCCATCAAAGCAGAAGAAGAACCAGTT------------------------------------------------------------------------------------------------------------GAACCAAAGCCAGAAATAGTTATGGAACCAGATGAGGAACCAGTTATTCCGGAAGCAGATATTGCGGAGACCGAAATACAACCCATCAAAGCAGAAGAAGAACCAGTTGAACCAAAGCCAGAAATAGTTATGGAACCAGATGAGGAACCAGTTATTCCGGAAGCAGATATTGCGGAGACCGAAATACAACCCATCAAAGCAGAAGAAGAACCAGTTGAACCAAAGCCAGAAATAGTTATGGAACCAGATGAGGAACCAGTTATTCCGGAAGCAGATATTGCGGAGACCGAAATACAACCCATCAGAGCAGAAGAAGAACCAGTTGAACCAAAGCCAGAAATAGTTATGGAACCAGATGAGGAACCAGTCATTCCAGAAACAGAACTAACAGAGACAGACAAACAACCAATCGAAGCAGAAGTAGAACCAGTTGAACCAAAAACAGAAGTGGTTGTGGAACCAGTTATTCCGGAAGCAGATATTGCGGAGACCGAAATACAACCCATCGAAGCAGAAGTAGAACCAGTTGAACCAAAGCCAGAAATAGTTATGGAACCAGATGTGGAACCAGTAATTCCAGAAACAGATCTTACAGAGACCGAAAAACAACCAATCGAAGCAGAAGTAGAACCAGTTGAACCGAAGACAGAGATAGTCGAACCAGAAACAGAGCCAGTCGAAGAAGCCGAGGAAGAAGCGGTTGAAGCACCCGTTATGGAACCAGTTATACCTGAGATAGAACCAAAGACAGAACCGGAAGTTGAAGCCGAGGAAAAAGAAGTCGAAACAAACGTTGAACCAGAACTACTGGAGACAGGAAAACCGATTGAAATTGGCCAAGCAACTGAAATCGAGGAGCCACCAGAAAAACAACCGGTGGAATCGGAGTCACAACCAGCGGAAGTTGAGATGGAACAAATTGAAACAAAGGAAGAACAAACTGAACCAAAGGAAGAACTAGCAGGAATTGATGAGAAATGTAAGTTTTAGTAACAATTATTTATTTTATGATTCCTTTTGAGCTGTTTCGGTATGCTTCGATGCAAGTCAAATTTGTGGATGTTTTAAATCCATCATTCAATTATGTTTGCAATGTAGTCATGAAGTTTGTTCGTTTGTTCAACACAGATATGGGACATATACGTATGAGCAGACCTTTTTTGTGCACAGATCTTCGTTCAGGTTAATCGTGCATGGCAACAAAAATTGGTTCATTCTGTTTTATGTCGTGCAGTCTTATTTTGCTTGAATTAAGCATTGCTGTAAAGTTCAAGTTAAAGGTACAGTCCATCATTTGTAATTTGTGCATTTTGTTCGTTTGAAGAAACAAACATGCTCAAAATCTAAAGAAGGCTGCTGAACAACTAACCT---------------------------------------------------------------------------------------------------------------------------------------------------------------------------------------------------------------------------------------------------------------------------------------GATGAAGTTTCAGCTCAGCTTCAGCTGTATAAATTATTTCGAGAAAAACAAAATTCTAGGATCTCCATTTCAAATCAAAAGTTGCTGAATCTTGTTTTGAATTGGGCCTGAATTCAGCGTCCGAGGCACGTTCTCATCACTGAGCCGATTGCAGCTACGCGATGTCGCGCACGAATTAAAGCAATCGAGGGCGCGTGTGCGTTGTGTCAACGCTTGCGCGCGCTTCGCCAAATAAAAAAAAAAGAACTAAACGGAAATTTCTTTAGACGCATGTCTTTAGACGCACCGTTGGAAAGGCCTACTTTCGTTGCTTTCTTCAAAATGACAGCATGCCANAAAGAAAAATCTCTTATCTTTCAACGAAGTATGTTTTTTAACAATAGGCCTAATTACGTCAGTTACTTTTTGGATAGTTGCAAATAATGATGGACTGTAGCCTTAAGAAATTTGTGGAACTCAAGTTGTGCTTGGCAAGTTTTGCTTTAGAATTCGAAAATGCATCGACACCAAAAAAAAGTGTAATTTTGGTCCTATAGTTGAAGTGTCAAACTAGAACAAATTTAATGATAAATTAAATAAATTTAAATAATTTATTCCTACCTTGCTTTGACAGTGATGAAGGAGCTGCGTGACCTTTTGGAATCTACGAAGATTGACCTTCCTGTTGACATCAATGATCCATACGACCTAGGTCTTCTTCTCAGACATTTACGTCACCATTCAAATCTTCTTGCTCGTATTGGAGACCCCGATGTCAAAAAGGAAGTCCTCAGCGCCATGAATGAAAAC

>BA74-B

GCACGACGTGCCGCAGAGGAAAAACCTCAAAAGGGAAAGAAGGGACGAAAAGGAAAGAAACGACGAAAGGGAAAGAAGGGACGAAAAGGAAAGAAGGGACGAAAAGGAAAGAAACGACGAAAGGGAAAGAAAAGACGAAAGGGAAAGAAAGGACGAAAGGGAAAGAAAGGACGAAAGGGAAAGAAAGGACGAAAGGGAAAGAAAGGACGAAAGGGAAAGAAAGGACGAAAGGGAAAGAAAGGACGAAAGGGAAAGAAAGGACGAAAGGGAAAGAAAGGAAAACCAATCCAACCTGAAGAAACACCAGCCATTCCGACGGAGATAAAAGCTGCAGAAATAGAAAAGGAACCAAAAACAGAAGTGGTTGTGGAACCAGTTATTCCGGAAGCAGATATTGCGGAGACCGAAATACAACAAATTAAAGCAGAAGTAGAACCAGTTGAAACAAAGCCAGAAATAGTTATGGAACCAGATGTGGAACCAGTTATTCCAGAAACGGAACTTACAGAGACCGGAAAAGAAGCGAAAGTAGAACAAGTTAAACCAGTGGAAGGGAAGCTAAACTTA------------------------------------------------------------------------------------------------------------------------------------------------------------------------------------------------------------------------------------------------------------------------------------------------------------------------------------------------------------------------------------------------------------------------------------------------------------------------------GGAAAAGGAAAGGGCAAAGGAAAGAAGGTTAGAAAGGGAAAGAAAGGACGAAAGGGAAAGAAAGGACGAAAGGGAAAGAAAGGACGGAAAGGAAAGAAGGGGCGAAAAGGAAAGAAAAGAAGAAAAGGAAAAAAAGGACGAAAGGGAAAGAAAGGACGAAAGGGAAAGAAAGGACGAAAGGGCAAGAAAGGACGAAAGGGAAAGAAAGGACGAAAGGGAAAGAAAGGACGAAAGGGAAAGAAAGGACGAAGGGGAAAGAAAGGA------------------CGAAAGGGCAAGAAAGGAAATCCAATCAAAACTGAAGAAACACCAGCCATTCTG------------------------------------------------------------------------------------------------------------------------------------------------------------------------------------------------------------------------------------------------------------------------------------------------------------------------------------------------------------------------------------------------------------------------------------------------------------------------------ACGGAGATAAAAGCTGCAGAAATAGAAAAGGAACCAAAAACAGAAGTGGTTGTGGAACCACTTATTCAGGAAAAAGATGTTGCGGAGACCGAAATACAACCCATCGAAGCAGAAGTAGAACCAGTTGAACCAAAGACAGAAATAGTTATGGAACCAGTTATTCCGGAAGCAGATATTGCGGAGACCGAAATACAACCCATCAAAGCAGAAGAAGAACCAGTTGAACCAAAGCCAGAAATAGTTATGGAACCAGATGAGGAACCAGTTATTCCGGAAGCAGATATTGCGGAGACCGAAATACAACCCATCAAAGCAGAAGAAGAACCAGTTGAACCAAAGCCAGAAATAGTTATGGAACCAGATGAGGAACCAGTTATTCCGGAAGCAGATATTGCGGAGACCGAAATACAACCCATCAAAGCAGAAGAAGAACCAGTTGAACCAAAGCCAGAAATAGTTATGGAACCAGATGAGGAACCAGTTATTCCGGAAGCAGATATTGCGGAGACCGAAATACAACCCATCAAAGCAGAAGAAGAACCAGTTGAACCAAAGCCAGAAATAGTTATGGAACCAGATGAGGAACCAGTTATTCCGGAAGCAGATATTGCGGAGACCGAAATACAACCCATCAGAGCAGAAGAAGAACCAGTTGAACCAAAGCCAGAAATAGTTATGGAACCAGATGAGGAACCAGTCATTCCAGAAACAGAACTAACAGAGACAGACAAACAACCAATCGAAGCAGAAGTAGAACCAGTTGAACCAAAAACAGAAGTGGTTGTGGAACCAGTTATTCCGGAAGCAGATATTGCGGAGACCGAAATACAACCCATCGAAGCAGAAGTAGAACCAGTTGAACCAAAGCCAGAAATAGTTATGGAACCAGATGTGGAACCAGTAATTCCAGAAACAGATCTTACAGAGACCGAAAAACAACCAATCGAAGCAGAAGTAGAACCAGTTGAACCGAAGACAGAGATAGTCGAACCAGAAACAGAGCCAGTCGAAGAAGCCGAGGAAGAAGCGGTTGAAGCACCCGTTATGGAACCAGTTATACCTGAGATAGAACCAAAGACAGAACCGGAAGTTGAAGCCGAGGAAAAAGAAGTCGAAACAAACGTTGAACCAGAACTACTGGAGACAGGAAAACCGATTGAAATTGGCCAAGCAACTGAAATCGAGGAGCCACCAGAAAAACAACCGGTGGAATCGGAGTCACAACCAGCGGAAGTTGAGATGGAACAAATTGAAACAAAGGAAGAACAAACTGAACCAAAGGAAGAACTAGCAGGAATTGATGAGAAATGTAAGTTTTAGTAACAATTATTTATTTTATGATTCCTTTTGAGCTGTTTCGGTATGCTTCGATGCAAGTCAAATTTGTGGATGTTTTAAATCCATCATTCAATTATGTTTGCAATGTAGTCATGAAGTTTGTTCGTTTGTTCAACACAGATATGGGACATATACGCATGAGCAGACCTTTTTTGTGCACAGATCTTCGTTCAGGTTAATCGTGCATGGCAACAAAAATTGGTTCATTCCGTTTTATGTCGTGCAGTCTTATTTTGCTTGAATTAAGCATTGCTGTAAAGTTCAAGTTAAAGGTACATTCCATCATTTGTAATTTGTGCATTTTGTTCGTTTGAAGAAACAAACATGCTCAAAATCTAAAGAAGGCTGCTGAACAACTAACCT---------------------------------------------------------------------------------------------------------------------------------------------------------------------------------------------------------------------------------------------------------------------------------------GATGAAGTTTCAGCTCAGCTTCAGCTGTATAAATTATTTCGAGAAAAACAAAATTCTAGGATCTCCATTTCAAATCAAAAGTTGCTGAATCTTGTTTTGAATTGGGCCTGAATTCAGCGTCCGAGGCACGTTCTCATCACTGAGCCGATTGCAGCTACGCGATGTCGCGCACGAATTAAAGCAATCGAGGGCGCGTGTGCGTTGTGTCAACGCTTGCGCGCGCTTCGCCAAATAAAAAAAAAAGAACTAAACGGAAATTTCTTTAGACGCATGTCTTTAGACGCACCGTTGGAAAGGCCTACTTTCGTTGCTTTCTTCAAAATGACAGCATGCCAGAAAGAAAAATCTCTTATCTTTCAACGAAGTATGTTTTTTAACAATAGGCCTAATTACGTCAGTTGCTTTTTGGATAGTTGCAAATAATGATGGACTGTAGCTTTAAGAAATTTGTGGAACTCAAGTTGTGCTTGGCAAGTTTTACTTTAGAATTCGAAAATGCATCGACACCAAAAAAAAGTGTAATTTTGGTCCTATAGTTGAAGTGTCAAACTAGAACAAATTTAATGATAAATTAAATAAATTTAAATAATTTATTCCTACCTTGCTTTGACAGTGATGAAGGAGCTGCGTGACCTTTTGGAATCTACGAAGATTGACCTTCCTGTTGACATCAATGATCCATACGACCTAGGTCTTCTTCTCAGACATTTACGTCACCATTCAAATCTTCTTGCTCGTATTGGAGACCCCGATGTCAAAAAGGAAGTCCTCAGCGCCATGAATGAAAAC

>BA80-A

GCACGACGTGCCGCAGAGGAAAAACCTCAAAAGGGAAAGAAGGGACGAAAAGGAAAGAAACGACGAAAGGGAAAGAAGGGACGAAAAGGAAAGAAGGGACGAAAAGGAAAGAAACGACGAAAGGGAAAGAAAAGACGAAAGGGAAAGAAAGGACGAAAGGGAAAGAAAGGACGAAAGGGAAAGAAAGGACGAAAGGGAAAGAAAGGACGAAAGGGAAAGAAAGGACGAAAGGGAAAGAAAGGACGAAAGGGAAAGAAAGGACGAAAGGGAAAGAAAGGAAAACCAATCCAACCTGAAGAAACACCAGCCATTCCGACGGAGATAAAAGCTGCAGAAATAGAAAAGGAACCAAAAACAGAAGTGGTTGTGGAACCAGTTATTCCGGAAGCAGATATTGCGGAGACCGAAATACAACAAATTAAAGCAGAAGTAGAACCAGTTGAAACAAAGCCAGAAATAGTTATGGAACCAGATGTGGAACCAGTTATTCCAGAAACGGAACTTACAGAGACCGGAAAAGAAGCGAAAGTAGAACAAGTTAAACCAGTGGAAGGGAAGCTAAACTTA------------------------------------------------------------------------------------------------------------------------------------------------------------------------------------------------------------------------------------------------------------------------------------------------------------------------------------------------------------------------------------------------------------------------------------------------------------------------------GGAAAAGGAAAGGGCAAAGGAAAGAAGGTTAGAAAGGGAAAGAAAGGACGAAAGGGAAAGAAAGGACGAAAGGGAAAGAAAGGACGGAAAGGAAAGAAGGGGCGAAAAGGAAAGAAAAGAAGAAAAGGA------------------------------------------------------------------------------------------------------------------------------------------------------------------------------------------------------------------------------------------------------------------------------------------------------------------------------------------------------------------------------------------------------------------------------------------------------------------------------------------------AAGAAAAGACGAAAAGGAAAGAAACGACGAAAGGGAAAGAAAGGACGAAAGGGAAAAAAAGGACGAAAGGGAAAGAAAGGACGAAAGGGAAAAAAAGGACGAAAGGGAAAAAAAGGACGAAAGGGAAAGAAAGGACGAAAGGGAAAGAAAGGCAAACCAATCCAAACTGAAGAAACACCAGCCATTCCGACGGAGATAAAAGCTGCAGAAATAGAAAAGGAACCAAAAACAGAAGTGGTTGTGGAACCACTTATTCAGGAAAAAGATGTTGCGGAGACCGAAATACAACCCATCGAAGCAGAAGTAGAACCAGTTGAACCAAAGACAGAAATAGTTATGGAACCAGTTATTCCGGAAGCAGATATTGCGGAGACCGAAATACAACCCATCAAAGCAGAAGAAGAACCAGTT------------------------------------------------------------------------------------------------------------GAACCAAAGCCAGAAATAGTTATGGAACCAGATGAGGAACCAGTTATTCCGGAAGCAGATATTGCGGAGACCGAAATACAACCCATCAAAGCAGAAGAAGAACCAGTTGAACCAAAGCCAGAAATAGTTATGGAACCAGATGAGGAACCAGTTATTCCGGAAGCAGATATTGCGGAGACCGAAATACAACCCATCAAAGCAGAAGAAGAACCAGTTGAACCAAAGCCAGAAATAGTTATGGAACCAGATGAGGAACCAGTTATTCCGGAAGCAGATATTGCGGAGACCGAAATACAACCCATCAAAGCAGAAGAAGAACCAGTTGAACCAAAGCCAGAAATAGTTATGGAACCAGATGAGGAACCAGTCATTCCAGAAACAGAACTAACAGAGACGGACAAACAACCAATCGAAGCAGAAGTAGAACCAGTTGAACCAAAAACAGAAGTGGTTGTGGAACCAGTTATTCCGGAAGCAGATATTGCGGAGACCGAAATACAACCCATCGAAGCAGAAGTAGAACCAGTTGAACCAAAGCCAGAAATAGTTATGGAACCAGATGTGGAACCAGTAATTCCAGAAACAGATCTTACAGAGACCGAAAAACAACCAATCGAAGCAGAAGTAGAACCAGTTGAACCGAAGACAGAGATAGTCGAACCAGAAACAGAGCCAGTCGAAGAAGCCGAGGAAGAAGCGGTTGAAGCACCCGTTATGGAACCAGTTATACCTGAGATAGAACCAAAGACAGAACCGGAAGTTGAAGCCGAGGAAAAAGAAGTCGAAACAAACGTTGAACCAGAACTACTGGAGACAGGAAAACCGATTGAAATTGGCCAAGCAACTGAAATCGAGGAGCCACCAGAAAAACAACCGGTGGAATCGGAGTCACAACCAGCGGAAGTTGAGATGGAACAAATTGAAACAAAGGAAGAACAAACTGAACCAAAGGAAGAACTAGCAGGAATTGATGAGAAATGTAAGTTTTAGTAACAATTATTTATTTTATGATTCCTTTTGAGCTGTTTCGGTATGCTTCGATGCAAGTCAAATTTGTGGATGTTTTAAATCCATCATTCAATTATGTTTGCAATGTAGTCATGAAGTTTGTTCGTTTGTTCAACACAGATATGGGACATATACGCATGAGCAGACCTTTTTTGTGCACAGATCTTCGTTCAGGTTAATCGTGCATGGCAACAAAAATTGGTTCATTCCGTTTTATGTCGTGCAGTCTTATTTTGCTTGAATTAAGCATTGCTGTAAAGTTCAAGTTAAAGGTACAGTCCATCATTTGTAATTTGTGCATTTTGTTCGTTTGAAGAAACAAACATGCTCAAAATCTAAAGAAGGCTGCTGAACAACTAACCT---------------------------------------------------------------------------------------------------------------------------------------------------------------------------------------------------------------------------------------------------------------------------------------GATGAAGTTTCAGCTCAGCTTCAGCTGTATAAATTATTTCGAGAAAAACAAAATTCTAGGATCTCCATTTCAAATCAAAAGTTGCTGAATCTTGTTTTGAATTGGGCCTGAATTCAGCGTCCGAGGCACGTTCTCATCACTGAGCCGATTGCAGCTACGCGATGTCGCGCACGAATTAAAGCAATCGAGGGCGCGTGTGCGTTGTGTCAACGCTTGCGCGCGCTTCGCC-AATAAAAAAAAAAGAACTAAACGGAAATTTCTTTAGACGCATGTCTTTAGACGCACCGTTGGAAAGGCCTACTTTCGTTGCTTTCTTCAAAATGACAGCATGCCAGAAAGAAAAATCTCTTATCTTTCAACGAAGTATGTTTTTTAACAATAGGCCTAATTACGTCAGTTACTTTTTGGATAGTTGCAAATAATGATGGACTGTAGCTTTAAGAAATTTGTGGAACTCAAGTTGTGCTTGGCAAGTTTTACTTTAGAATTCGAAAATGCATCGACACCAAAAAAAAGTGTAATTTTGGTCCTATAGTTGAAGTGTCAAACTAGAACAAATTTAATGATAAATTAAATAAATTTAAATAATTTATTCCTACCTTGCTTTGACAGTGATGAAGGAGCTGCGTGACCTTTTGGAATCTACGAAGATTGACCTTCCTGTTGACATCAATGATCCATACGACCTAGGTCTTCTTCTCAGACATTTACGTCACCATTCAAATCTTCTTGCTCGTATTGGAGACCCCGATGTCAAAAAGGAAGTCCTCAGCGCCATGAATGAAAAC

>BA94-A

GCACGACGTGCCGCAGAGGAAAAACCTCAAAAGGGAAAGAAGGGACGAAAAGGAAAGAAACGACGAAAGGGAAAGAAGGGACGAAAAGGAAAGAAGGGACGAAAAGGAAAGAAACGACGAAAGGGAAAGAAAAGACGAAAGGGAAAGAAAGGACGAAAGGGAAAGAAAGGACGAAAGGGAAAGAAAGGACGAAAGGGAAAGAAAGGACGAAAGGGAAAGAAAGGACGAAAGGGAAAGAAAGGACGAAAGGGAAAGAAAGGACGAAAGGGAAAGAAAGGAAAACCAATCCAACCTGAAGAAACACCAGCCATTCCGACGGAGATAAAAGCTGCAGAAATAGAAAAGGAACCAAAAACAGAAGTGGTTGTGGAACCAGTTATTCCGGAAGCAGATATTGCGGAGACCGAAATACAACAAATTAAAGCAGAAGTAGAACCAGTTGAAACAAAGCCAGAAATAGTTATGGAACCAGATGTGGAACCAGTTATTCCAGAAACGGAACTTACAGAGACCGGAAAAGAAGCGAAAGTAGAACAAGTTAAACCAGTGGAAGGGAAGCTAAACTTA------------------------------------------------------------------------------------------------------------------------------------------------------------------------------------------------------------------------------------------------------------------------------------------------------------------------------------------------------------------------------------------------------------------------------------------------------------------------------GGAAAAGGAAAGGGCAAAGGAAAGAAGGTTAGAAAGGGAAAGAAAGGACGAAAGGGAAAGAAAGGACGAAAGGGAAAGAAAGGACGGAAAGGAAAGAAGGGGCGAAAAGGAAAGAAAAGAAGAAAAGGA------------------------------------------------------------------------------------------------------------------------------------------------------------------------------------------------------------------------------------------------------------------------------------------------------------------------------------------------------------------------------------------------------------------------------------------------------------------------------------------------AAGAAAAGACGAAAAGGAAAGAAACGACGAAAGGGAAAGAAAGGACGAAAGGGAAAAAAAGGACGAAAGGGAAAGAAAGGACGAAAGGGAAAAAAAGGACGAAAGGGAAAAAAAGGACGAAAGGGAAAGAAAGGACGAAAGGGAAAGAAAGGCAAACCAATCCAAACTGAAGAAACACCAGCCATTCCGACGGAGATAAAAGCTGCAGAAATAGAAAAGGAACCAAAAACAGAAGTGGTTGTGGAACCACTTATTCAGGAAAAAGATGTTGCGGAGACCGAAATACAACCCATCGAAGCAGAAGTAGAACCAGTTGAACCAAAGACAGAAATAGTTATGGAACCAGTTATTCCGGAAGCAGATATTGCGGAGACCGAAATACAACCCATCAAAGCAGAAGAAGAACCAGTT------------------------------------------------------------------------------------------------------------------------------------------------------------------------------------------------------------------------------------------------------------------------------------------------------------------------------------------------------------------------------------------------------------------------------------------------------------------------------------------------------------------------------------------------------------------------------------------------------------------------------------------------------------GAACCAAAGCCAGAAATAGTTATGGAACCAGATGTGGAACCAGTAATTCCAGAAACAGATCTTACAGAGACCGAAAAACAACCAATCGAAGCAGAAGTAGAACCAGTTGAACCGAAGACAGAGATAGTCGAACCAGAAACAGAGCCAGTCGAAGAAGCCGAGGAAGAAGCGGTTGAAGCACCCGTTATGGAACCAGTTATACCTGAGATAGAACCAAAGACAGAACCGGAAGTTGAAGCCGAGGAAAAAGAAGTCGAAACAAACGTTGAACCAGAACTACTGGAGACAGGAAAACCGATTGAAATTGGCCAAGCAACTGAAATCGAGGAGCCACCAGAAAAACAACCGGTGGAATCGGAGTCACAACCAGCGGAAGTTGAGATGGAACAAATTGAAACAAAGGAAGAACAAACTGAACCAAAGGAAGAACTAGCAGGAATTGATGAGAAATGTAAGTTTTAGTAACAATTATTTATTTTATGATTCCTTTTGAGCTGTTTCGGTATGCTTCGATGCAAGTCAAATTTGTGGATGTTTTAAATCCATCATTCAATTATGTTTGCAATGTAGTCATGAAGTTTGTTCGTTTGTTCAACACAGATATGGGACATATACGCATGAGCAGACCTTTTTTGTGCACAGATCTTCGTTCAGGTTAATCGTGCATGGCAACAAAAATTGGTTCATTCCGTTTTATGTCGTGCAGTCTTATTTTGCTTGAATTAAGCATTGCTGTAAAGTTCAAGTTAAAGGTACAGTCCATCATTTGTAATTTGTGCATTTTGTTCGTTTGAAGAAACAAACATGCTCAAAATCTAAAGAAGGCTGCTGTACAACTAACCT---------------------------------------------------------------------------------------------------------------------------------------------------------------------------------------------------------------------------------------------------------------------------------------GATGAAGTTTCAGCTCAGCTTCAGCTGTATAAATTATTTCGAGAAAAACAAAATTCTAGGATCTCCATTTCAAATCAAAAGTTGCTGAATCTTGTTTTGAATTGGGCCTGAATTCAGCGTCCGAGGCACGTTCTCATCACTGAGCCGATTGCAGCTACGCGATGTCGCGCACGAATTAAAGCAATCGAGGGCGCGTGTGCGTTGTGTCAACGCTTGCGCGCGCTTCGCCAAATAAAAAAAAA-GAACTAAACGGAAATTTCTTTAGACGCATGTCTTTAGACGCACCGTTGGAAAGGCCTACTTTCGTCGCTTTCTTCAAAATGACAGCATGCCAGAAAGAAAAATCTCTTATCTTTCAACGAAGTATGTTTTTTAACAATAGGCCTAATTACGTCAGTTACTTTTTGGATAGTTGCAAATAATGATGGACTGTAGCTTTAAGAAATTTGTGGAACTCAAGTTGTGCTTGGCAAGTTTTACTTTAGAATTCGAAAATGCATCGACACCAAAAAAAAGTGTAATTTTGGTCCTATAGTTGAAGTGTCAAACTAGAACAAATTTAATGATAAATTAAATAAATTTAAATAATTTATTCCTACCTTGCTTTGACAGTGATGAAGGAGCTGCGTGACCTTTTGGAATCTACGAAGATTGACCTTCCTGTTGACATCAATGATCCATACGACCTAGGTCTTCTTCTCAGACATTTACGTCACCATTCAAATCTTCTTGCTCGTATTGGAGACCCCGATGTCAAAAAGGAAGTCCTCAGCGCCATGAATGAAAAC

>BA89-B

GCACGACGTGCCGCAGAGGAAAAACCTCAAAAGGGAAAGAAGGGACGAAAAGGAAAGAAACGACGAAAGGGAAAGAAGGGACGAAAAGGAAAGAAGGGACGAAAAGGAAAGAAACGACGAAAGGGAAAGAAAAGACGAAAGGGAAAGAAAGGACGAAAGGGAAAGAAAGGACGAAAGGGAAAGAAAGGACGAAAGGGAAAGAAAGGACGAAAGGGAAAGAAAGGACGAAAGGGAAAGAAAGGACGAAAGGGAAAGAAAGGACGAAAGGGAAAGAAAGGAAAACCAATCCAACCTGAAGAAACACCAGCCATTCCGACGGAGATAAAAGCTGCAGAAATAGAAAAGGAACCAAAAACAGAAGTGGTTGTGGAACCAGTTATTCCGGAAGCAGATATTGCGGAGACCGAAATACAACAAATTAAAGCAGAAGTAGAACCAGTTGAAACAAAGCCAGAAATAGTTATGGAACCAGATGTGGAACCAGTTATTCCAGAAACGGAACTTACAGAGACCGGAAAAGAAGCGAAAGTAGAACAAGTTAAACCAGTGGAAGGGAAGCTAAACTTA------------------------------------------------------------------------------------------------------------------------------------------------------------------------------------------------------------------------------------------------------------------------------------------------------------------------------------------------------------------------------------------------------------------------------------------------------------------------------GGAAAAGGAAAGGGCAAAGGAAAGAAGGTTAGAAAGGGAAAGAAAGGACGAAAGGGAAAGAAAGGACGAAAGGGAAAGAAAGGACGGAAAGGAAAGAAGGGGCGAAAAGGAAAGAAAAGAAGAAAAGGA------------------------------------------------------------------------------------------------------------------------------------------------------------------------------------------------------------------------------------------------------------------------------------------------------------------------------------------------------------------------------------------------------------------------------------------------------------------------------------------------AAGAAAAGACGAAAAGGAAAGAAACGACGAAAGGGAAAGAAAGGACGAAAGGGAAAAAAAGGACGAAAGGGAAAGAAAGGACGAAAGGGAAAAAAAGGACGAAAGGGAAAAAAAGGACGAAAGGGAAAGAAAGGACGAAAGGGAAAGAAAGGCAAACCAATCCAAACTGAAGAAACACCAGCCATTCCGACGGAGATAAAAGCTGCAGAAATAGAAAAGGAACCAAAAACAGAAGTGGTTGTGGAACCACTTATTCAGGGAAAAGATGTTGCGGAGACCGAAATACAACCCATCGAAGCAGAAGTAGAACCAGTTGAACCAAAGACAGAAATAGTTATGGAACCAGTTATTCCGGAAGCAGATATTGCGGAGACCGAAATACAACCCATCAAAGCAGAAGAAGAACCAGTT------------------------------------------------------------------------------------------------------------GAACCAAAGCCAGAAATAGTTATGGAACCAGATGAGGAACCAGTTATTCCGGAAGCAGATATTGCGGAGACCGAAATACAACCCATCAAAGCAGAAGAAGAACCAGTTGAACCAAAGCCAGAAATAGTTATGGAACCAGATGAGGAACCAGTTATTCCGGAAGCAGATATTGCGGAGACCGAAATACAACCCATCAAAGCAGAAGAAGAACCAGTTGAACCAAAGCCAGAAATAGTTATGGAACCAGATGAGGAACCAGTTATTCCGGAAGCAGATATTGCGGAGACCGAAATACAACCCATCAGAGCAGAAGAAGAACCAGTTGAACCAAAGCCAGAAATAGTTATGGAACCAGATGAGGAACCAGTCATTCCAGAAACAGAACTAACAGAGACAGACAAACAACCAATCGAAGCAGAAGTAGAACCAGTTGAACCAAAAACAGAAGTGGTTGTGGAACCAGTTATTCCGGAAGCAGATATTGCGGAGACCGAAATACAACCCATCGAAGCAGAAGTAGAACCAGTTGAACCAAAGCCAGAAATAGTTATGGAACCAGATGTGGAACCAGTAATTCCAGAAACAGATCTTACAGAGACCGAAAAACAACCAATCGAAGCAGAAGTAGAACCAGTTGAACCGAAGACAGAGATAGTCGAACCAGAAACAGAGCCAGTCGAAGAAGCCGAGGAAGAAGCGGTTGAAGCACCCGTTATGGAACCAGTTATACCTGAGATAGAACCAAAGACAGAACCGGAAGTTGAAGCCGAGGAAAAAGAAGTCGAAACAAACGTTGAACCAGAACTACTGGAGACAGGAAAACCGATTGAAATTGGCCAAGCAACTGAAATCGAGGAGCCACCAGAAAAACAACCGGTGGAATCGGAGTCACAACCAGCGGAAGTTGAGATGGAACAAATTGAAACAAAGGAAGAACAAACTGAACCAAAGGAAGAACTAGCAGGAATTGATGAGAAATGTAAGTTTTAGTAACAATTATTTATTTTATGATTCCTTTTGAGCTGTTTCGGTATGCTTCGATGCAAGTCAAATTTGTGGATGTTTTAAATCCATCATTCAATTATGTTTGCAATGTAGTCATGAAGTTTGTTCGTTTGTTCAACACAGATATGGGACATATACGCATGAGCAGACCTTTTTTGTGCACAGATCTTCGTTCAGGTTAATCGTGCACGGCAACAAAAATTGGTTCATTCCGTTTTATGTCGTGCAGTCTTATTTTGCTTGAATTAAGCATTGCTGTAAAGTTCAAGTTAAAGGTACAGTCCATCATTTGTAATTTGTGCATTTTGTTCGTTTGAAGAAACAAACATGCTCAAAATCTAAAGAAGGCTGCTGAACAACTAACCT---------------------------------------------------------------------------------------------------------------------------------------------------------------------------------------------------------------------------------------------------------------------------------------GATGAAGTTTCAGCTCAGCTTCAGCTGTATAAATTATTTCGAGAAAAACAAAATTCTAGGATCTCCATTTCAAATCAAAAGTTGCTGAATCTTGTTTTGAATTGGGCCTGAATTCAGTGTCCGAGGCACGTTCTCATCACTGAGCCGATTGCAGCTACGCGATGTCGCGCACGAATTAAAGCAATCGAGGGCGCGTGTGCGTTGTGTCAACGCTTGCGCGCGCTTCGCCAAAT-AAAAAAAAGGAACTAAACGGAAATTTCTTTAGACGCATGTCTTTAGACGCACCGTTGGAAAGGCCTACTTTCGTTGCTTTCTTCAAAATGACAGCATGCCAGAAAGAAAAATCTCTTATCTTTCAACGAAGTATGTTTTTTAACAATAGGCCTANTTACGTCAGTTACTTTTTGGANAGTTGCAAATAATGATGGACTGTAGCTTTAAGAAATTTGTGGAACTCAAGTTGTGCTTGGCAAGTTTTACTTTAGAATTCGAAAACGCATCGACACCAAAAAAAAGTGTAATTTTGGTCCTATAGTTGAAGTGTCAAACTAGAACAAATTTAATGATAAATTAAATAAATTTAAATAATTTATTCCTACCTTGCTTTGACAGTGATGAAGGAGCTGCGTGACCTTTTGGAATCTACGAAGATTGACCTTCCTGTTGACATCAATGATCCATACGACCTAGGTCTTCTTCTCAGACATTTACGTCACCATTCAAATCTTCTTGCTCGTATTGGAGACCCCGATGTCAAAAAGGAAGTCCTCAGCGCCATGAATGAAAAC

>BA89-A

GCACGACGTGCCGCAGAGGAAAAACCTCAAAAGGGAAAGAAGGGACGAAAAGGAAAGAAACGACGAAAGGGAAAGAAGGGACGAAAAGGAAAGAAGGGACGAAAAGGAAAGAAACGACGAAAGGGAAAGAAAAGACGAAAGGGAAAGAAAGGACGAAAGGGAAAGAAAGGACGAAAGGGAAAGAAAGGACGAAAGGGAAAGAAAGGACGAAAGGGAAAGAAAGGACGAAAGGGAAAGAAAGGACGAAAGGGAAAGAAAGGACGAAAGGGAAAGAAAGGAAAACCAATCCAACCTGAAGAAACACCAGCCATTCCGACGGAGATAAAAGCTGCAGAAATAGAAAAGGAACCAAAAACAGAAGTGGTTGTGGAACCAGTTATTCCGGAAGCAGATATTGCGGAGACCGAAATACAACAAATTAAAGCAGAAGTAGAACCAGTTGAAACAAAGCCAGAAATAGTTATGGAACCAGATGTGGAACCAGTTATTCCAGAAACGGAACTTACAGAGACCGGAAAAGAAGCGAAAGTAGAACAAGTTAAACCAGTGGAAGGGAAGCTAAACTTA------------------------------------------------------------------------------------------------------------------------------------------------------------------------------------------------------------------------------------------------------------------------------------------------------------------------------------------------------------------------------------------------------------------------------------------------------------------------------GGAAAAGGAAAGGGCAAAGGAAAGAAGGTTAGAAAGGGAAAGAAAGGACGAAAGGGAAAGAAAGGACGAAAGGGAAAGAAAGGACGGAAAGGAAAGAAGGGGCGAAAAGGAAAGAAAAGAAGAAAAGGA------------------------------------------------------------------------------------------------------------------------------------------------------------------------------------------------------------------------------------------------------------------------------------------------------------------------------------------------------------------------------------------------------------------------------------------------------------------------------------------------AAGAAAAGACGAAAAGGAAAGAAACGACGAAAGGGAAAGAAAGGACGAAAGGGAAAAAAAGGACGAAAGGGAAAGAAAGGACGAAAGGGAAAAAAAGGACGAAAGGGAAAAAAAGGACGAAAGGGAAAGAAAGGACGAAAGGGAAAGAAAGGCAAACCAATCCAAACTGAAGAAACACCAGCCATTCCGACGGAGATAAAAGCTGCAGAAATAGAAAAGGAACCAAAAACAGAAGTGGTTGTGGAACCACTTATTCAGGAAAAAGATGTTGCGGAGACCGAAATACAACCCATCGAAGCAGAAGTAGAACCAGTTGAACCAAAGACAGAAATAGTTATGGAACCAGTTATTCCGGAAGCAGATATTGCGGAGACCGAAATACAACCCATCAAAGCAGAAGAAGAACCAGTT------------------------------------------------------------------------------------------------------------GAACCAAAGCCAGAAATAGTTATGGAACCAGATGAGGAACCAGTTATTCCGGAAGCAGATATTGCGGAGACCGAAATACAACCCATCAAAGCAGAAGAAGAACCAGTTGAACCAAAGCCAGAAATAGTTATGGAACCAGATGAGGAACCAGTTATTCCGGAAGCAGATATTGCGGAGACCGAAATACAACCCATCAAAGCAGAAGAAGAACCAGTTGAACCAAAGCCAGAAATAGTTATGGAACCAGATGAGGAACCAGTTATTCCGGAAGCAGATATTGCGGAGACCGAAATACAACCCATCAGAGCAGAAGAAGAACCAGTTGAACCAAAGCCAGAAATAGTTATGGAACCAGATGAGGAACCAGTCATTCCAGAAACAGAACTAACAGAGACAGACAAACAACCAATCGAAGCAGAAGTAGAACCAGTTGAACCAAAAACAGAAGTGGTTGTGGAACCAGNTATTCCGGAAGCAGATATTGCGGAGACCGAAATACAACCCATCGAAGCAGAAGTAGAACCAGTTGAACCAAAGCCAGAAATAGTTATGGAACCAGATGTGGAACCAGTAATTCCAGAAACAGATCTTACAGAGACCGAAAAACAACCAATCGAAGCAGAAGTAGAACCAGTTGAACCGAAGACAGAGATAGTCGAACCAGAAACAGAGCCAGTCGAAGAAGCCGAGGAAGAAGCGGTTGAAGCACCCGTTATGGAACCAGTTATACCTGAGATAGAACCAAAGACAGAACCGGAAGTTGAAGCCGAGGAAAAAGAAGTCGAAACAAACGTTGAACCAGAACTACTGGAGACAGGAAAACCGATTGAAATTGGCCAAGCAACTGAAATCGAGGAGCCACCAGAAAAACAACCGGTGGAATCGGAGTCACAACCAGCGGAAGTTGAGATGGAACAAATTGAAACAAAGGAAGAACAAACTGAACCAAAGGAAGAACTAGCAGGAATTGATGAGAAATGTAAGTTTTAGTAACAATTATTTATTTTATGATTCCTTTTGAGCTGTTTCGGTATGCTTCGATGCAAGTCAAATTTGTGGATGTTTTAAATCCATCATTCAATTATGTTTGCAATGTAGTCATGAAGTTTGTTCGTTTGTTCAACACAGATATGGGACATATACGCATGAGCAGACCTTTTTTGTGCACAGATCTTCGTTCAGGTTAATCGTGCATGGCAACAAAAATTGGTTCATTCCGTTTTATGTCGTGCAGTCTTATTTTGCTTGAATTAAGCATTGCTGTAAAGTTCAAGTTAAAGGTACAGTCCATCATTTGTAATTTGTGCATTTTGTTCGTTNGAAGAAACAAACATGCTCAAAATCTAAAGAAGGCTGCTGAACAACTAACCT---------------------------------------------------------------------------------------------------------------------------------------------------------------------------------------------------------------------------------------------------------------------------------------GATGAAGTTTCAGCTCAGCTTCAGCTGTATAAATTATTTCGAGAAAAACAAAATTCTAGGATCTCCATTTCAAATCAAAAGTTGCTGAATCTTGTTTTGAATTGGGCCTGAATTCAGCGTCCGAGGCACGTTCTCATCACTGAGCCGATTGCAGCTACGCGATGTCGCGCACGAATTAAAGCAATCGAGGGCGCGTGTGCGTTGTGTCAACGCTTGCGCGCGCTTCGCCAAATAAAAAAAAAAGAACTAAACGGAAATTTCTTTAGACGCATGTCTTTAGACGCACCGTTGGAAAGGCCTACTTTCGTTGCTTTCTTCAAAATGACAGCATGCCAGAAAGAAAAATCTCTTATCTTTCAACGAAGTATGTTTTTTAACANTAGGCCTAATTACGTCAGTTACTTTTTGGATAGTTGCAAATAATGATGGACTGTAGCTTTAAGAAATTTGTGGAACTCAAGTTGTGCTTGGCAAGTTTTACTTTAGAATTCGAAAATGCATCGACACCAAAAAAAAGTGTAATTTTGGTCCTATAGTTGAAGTGTCAAACTAGAACAAATTTAATGATAAATTAAATAAATTTAAATAATTTATTCCTACCTTGCTTTGACAGTGATGAAGGAGCTGCGTGACCTTTTGGAATCTACGAAGATTGACCTTCCTGTTGACATCAATGATCCATACGACCTAGGTCTTCTTCTCAGACATTTACGTCACCATTCAAATCTTCTTGCTCGTATTGGAGACCCCGATGTCAAAAAGGAAGTCCTCAGCGCCATGAATGAAAAC

>BA58-A

GCACGACGTGCCGCAGAGGAAAAACCTCAAAAGGGAAAGAAGGGACGAAAAGGAAAGAAACGACGAAAGGGAAAGAAGGGACGAAAAGGAAAGAAGGGACGAAAAGGAAAGAAACGACGAAAGGGAAAGAAAAGACGAAAGGGAAAGAAAGGACGAAAGGGAAAGAAAGGACGAAAGGGAAAGAAAGGACGAAAGGGAAAGAAAGGACGAAAGGGAAAGAAAGGACGAAAGGGAAAGAAAGGACGAAAGGGAAAGAAAGGACGAAAGGGAAAGAAAGGAAAACCAATCCAACCTGAAGAAACACCAGCCATTCCGACGGAGATAAAAGCTGCAGAAATAGAAAAGGAACCAAAAACAGAAGTGGTTGTGGAACCAGTTATTCCGGAAGCAGATATTGCGGAGACCGAAATACAACAAATTAAAGCAGAAGTAGAACCAGTTGAAACAAAGCCAGAAATAGTTATGGAACCAGATGTGGAACCAGTTATTCCAGAAACGGAACTTACAGAGACCGGAAAAGAAGCGAAAGTAGAACAAGTTAAACCAGTGGAAGGGAAGCTAAACTTA------------------------------------------------------------------------------------------------------------------------------------------------------------------------------------------------------------------------------------------------------------------------------------------------------------------------------------------------------------------------------------------------------------------------------------------------------------------------------GGAAAAGGAAAGGGCAAAGGAAAGAAGGTTAGAAAGGGAAAGAAAGGACGAAAGGGAAAGAAAGGACGAAAGGGAAAGAAAGGACGGAAAGGAAAGAAGGGGCGAAAAGGAAAGAAAAGAAGAAAAGGA------------------------------------------------------------------------------------------------------------------------------------------------------------------------------------------------------------------------------------------------------------------------------------------------------------------------------------------------------------------------------------------------------------------------------------------------------------------------------------------------AAGAAAAGACGAAAAGGAAAGAAACGACGAAAGGGAAAGAAAGGACGAAAGGGAAAAAAAGGACGAAAGGGAAAGAAAGGACGAAAGGGAAAAAAAGGACGAAAGGGAAAAAAAGGACGAAAGGGAAAGAAAGGACGAAAGGGAAAGAAAGGCAAACCAATCCAAACTGAAGAAACACCAGCCATTCCGACGGAGATAAAAGCTGCAGAAATAGAAAAGGAACCAAAAACAGAAGTGGTTGTGGAACCACTTATTCAGGAAAAAGATGTTGCGGAGACCGAAATACAACCCATCGAAGCAGAAGTAGAACCAGTTGAACCAAAGACAGAAATAGTTATGGAACCAGTTATTCCGGAAGCAGATATTGCGGAGACCGAAATACAACCCATCAAAGCAGAAGAAGAACCAGTT------------------------------------------------------------------------------------------------------------GAACCAAAGCCAGAAATAGTTATGGAACCAGATGAGGAACCAGTTATTCCGGAAGCAGATATTGCGGAGACCGAAATACAACCCATCAAAGCAGAAGAAGAACCAGTTGAACCAAAGCCAGAAATAGTTATGGAACCAGATGAGGAACCAGTTATTCCGGAAGCAGATATTGCGGAGACCGAAATACAACCCATCAAAGCAGAAGAAGAACCAGTTGAACCAAAGCCAGAAATAGTTATGGAACCAGATGAGGAACCAGTTATTCCGGAAGCAGATATTGCGGAGACCGAAATACAACCCATCAGAGCAGAAGAAGAACCAGTTGAACCAAAGCCAGGAATAGTTATGGAACCAGATGAGGAACCAGTCATTCCAGAAACAGAACTAACAGAGACAGACAAACAACCAATCGAAGCAGAAGTAGAACCAGTTGAACCAAAAACAGAAGTGGTTGTGGAACCAGTTATTCCGGAAGCAGATATTGCGGAGACCGAAATACAACCCATCGAAGCAGAAGTAGAACCNGTTGAACCAAAGCCAGAAATAGTTATGGAACCAGATGTGGAACCAGTAATTCCAGAAACAGATCTTACAGAGACCGAAAAACAACCAATCGAAGCAGAAGTAGAACCAGTTGAACCGAAGACAGAGATAGTCGAACCAGAAACAGAGCCAGTCGAAGAAGCCGAGGAAGAAGCGGTTGAAGCACCCGTTATGGAACCAGTTATACCTGAGATAGAACCAAAGACAGAACCGGAAGTTGAAGCCGAGGAAAAAGAAGTNGAAACAAACGTTGAACCAGAACTACTGGAGACAGGAAAACCGATTGAAATTGGCCAAGCAACTGAAATCGAGGAGCCACCAGAAAAACAACCGGTGGAATCGGAGTCACAACCAGCGGAAGTTGAGATGGAACAAATTGAAACAAAGGAAGAACAAACTGAACCAAAGGAAGAACTAGCAGGAATTGATGAGAAATGTAAGTTTTAGTAACAATTATTTATTTTATGATTCCTTTTGAGCTGTTTCGGTATGCTTCGATGCAAGTCAAATTTGTGGATGTTTTAAATCCATCATTCAATTATGTTTGCAATGTAGTCATGAAGTTTGTTCGTTTGTTCAACACAGATATGGGACATATACGCATGAGCAGACCCTTTTTGTGCACAGATCTTCGTTCAGGTTAATCGTGCATGGCAACAAAAATTGGTTCATTCCGTTTTATGTCGTGCAGTCTTATTTTGCTTGAATTAAGCATTGCTGTAAAGTTCAAGTTAAAGGTACAGTCCATCATTTGTAATTTGTGCATTTTGTTCGTTTGAAGAAACAAACATGCTCAAAATCTAAAGAAGGCTGCTGAACAACTAACCT---------------------------------------------------------------------------------------------------------------------------------------------------------------------------------------------------------------------------------------------------------------------------------------GATGAAGTTTCAGCTCAGCTTCAGCTGTATAAATTATTTCGAGAAAAACAAAATTCTAGGATCTCCATTTCAAATCAAAAGTTGCTGAATTTTGTTTTGAATTGGGCCTGAATTCAGCGTCCGAGGCACGTTCTCATCACTGAGCCGATTGCAGCTACGCGATGTCGCGCGCGAATTAAAGCAATCGAGGGCGCGTGTGCGTTGTGTCAACGCTTGCGCGCGCTTCGCCAAATAAAAAAAAAAGAACTAAACGGAAATTTCTTTAGACGCATGTCTTTAGACGCACCGTTGGAAAGGCCTACTTTCGTTGCTTTCTTCAAAATGACAGCATGCCAGAAAGAAAAATCTCTTATCTTTCAACGAAGTATGTTTTTTAACAATAGGCCTAATTACGTCAGTTACTTTTTGGATAGTTGCAAATAATGATGGACTGTAGCTTTAAGAAATTTGTGGAACTCAAGTTGTGCTTGGCAAGTTTTACTTTAGAATTCGAAAATGCATCGACACCAAAAAAAAGTGTAATTTTGGTCCTATAGTTGAAGTGTCAAACTAGAACAAATTTAATGATAAATTAAATAAATTTAAATAATTTATTCCTACCTTGCTTTGACAGTGATGAAGGAGCTGCGTGACCTTTTGGAATCTACGAAGATTGACCTTCCTGTTGACATCAATGATCCATACGACCTAGGTCTTCTTCTCAGACATTTACGTCACCATTCAAATCTTCTTGCTCGTATTGGAGACCCCGATGTCAAAAAGGAAGTCCTCAGCGCCATGAATGAAAAC

>BA96-B

GCACGACGTGCCGCAGAGGAAAAACCTCAAAAGGGAAAGAAGGGACGAAAAGGAAAGAAACGACGAAAGGGAAAGAAGGGACGAAAAGGAAAGAAGGGACGAAAAGGAAAGAAACGACGAAAGGGAAAGAAAAGACGAAAGGGAAAGAAAGGACGAAAGGGAAAGAAAGGACGAAAGGGAAAGAAAGGACGAAAGGGAAAGAAAGGACGAAAGGGAAAGAAAGGACGAAAGGGAAAGAAAGGACGAAAGGGAAAGAAAGGACGAAAGGGAAAGAAAGGAAAACCAATCCAACCTGAAGAAACACCAGCCATTCCGACGGAGATAAAAGCTGCAGAAATAGAAAAGGAACCAAAAACAGAAGTGGTTGTGGAACCAGTTATTCCGGAAGCAGATATTGCGGAGACCGAAATACAACAAATTAAAGCAGAAGTAGAACCAGTTGAAACAAAGCCAGAAATAGTTATGGAACCAGATGTGGAACCAGTTATTCCAGAAACGGAACTTACAGAGACCGGAAAAGAAGCGAAAGTAGAACAAGTTAAACCAGTGGAAGGGAAGCTAAACTTA------------------------------------------------------------------------------------------------------------------------------------------------------------------------------------------------------------------------------------------------------------------------------------------------------------------------------------------------------------------------------------------------------------------------------------------------------------------------------GGAAAAGGAAAGGGCAAAGGAAAGAAGGTTAGAAAGGGAAAGAAAGGACGAAAGGGAAAGAAAGGACGAAAGGGAAAGAAAGGACGGAAAGGAAAGAAGGGGCGAAAAGGAAAGAAAAGAAGAAAAGGA------------------------------------------------------------------------------------------------------------------------------------------------------------------------------------------------------------------------------------------------------------------------------------------------------------------------------------------------------------------------------------------------------------------------------------------------------------------------------------------------AAGAAAAGACGAAAAGGAAAGAAACGACGAAAGGGAAAGAAAGGACGAAAGGGAAAAAAAGGACGAAAGGGAAAGAAAGGACGAAAGGGAAAAAAAGGACGAAAGGGAAAAAAAGGACGAAAGGGAAAGAAAGGACGAAAGGGAAAGAAAGGCAAACCAATCCAAACTGAAGAAACACCAGCCATTCCGACGGAGATAAAAGCTGCAGAAATAGAAAAGGAACCAAAAACAGAAGTGGTTGTGGAACCACTTATTCAGGAAAAAGATGTTGCGGAGACCGAAATACAACCCATCGAAGCAGAAGTAGAACCAGTTGAACCAAAGACAGAAATAGTTATGGAACCAGTTATTCCGGAAGCAGATATTGCGGAGACCGAAATACAACCCATCAAAGCAGAAGAAGAACCAGTT------------------------------------------------------------------------------------------------------------GAACCAAAGCCAGAAATAGTTATGGAACCAGATGAGGAACCAGTTATTCCGGAAGCAGATATTGCGGAGACCGAAATACAACCCATCAAAGCAGAAGAAGAACCAGTTGAACCAAAGCCAGAAATAGTTATGGAACCAGATGAGGAACCAGTTATTCCGGAAGCAGATATTGCGGAGACCGAAATACAACCCATCAAAGCAGAAGAAGAACCAGTTGAACCAAAGCCAGAAATAGTTATGGAACCAGATGAGGAACCAGTTATTCCGGAAGCAGATATTGCGGAGACCGAAATACAACCCATCAGAGCAGAAGAAGAACCAGTTGAACCAAAGCCAGAAATAGTTATGGAACCAGATGAGGAACCAGTCATTCCAGAAACAGAACTAACAGAGACAGACAAACAACCAATCGAAGCAGAAGTAGAACCAGTTGAACCAAAAACAGAAGTGGTTGTGGAACCAGTTATTCCGGAAGCAGATATTGCGGAGACCGAAATACAACCCATCGAAGCAGAAGTAGAACCAGTTGAACCAAAGCCAGAAATAGTTATGGAACCAGATGTGGAACCAGTAATTCCAGAAACAGATCTTACAGAGACCGAAAAACAACCAATCGAAGCAGAAGTAGAACCAGTTGAACCGAAGACAGAGATAGTCGAACCAGAAACAGAGCCAGTCGAAGAAGCCGAGGAAGAAGCGGTTGAAGCACCCGTTATGGAACCAGTTATACCTGAGATAGAACCAAAGACAGAACCGGAAGTTGAAGCCGAGGAAAAAGAAGTCGAAACAAACGTTGAACCAGAACTACTGGAGACAGGAAAACCGATTGAAATTGGCCAAGCAACTGAAATCGAGGAGCCACCAGAAAAACAACCGGTGGAATCGGAGTCACAACCAGCGGAAGTTGAGATGGAACAAATTGAAACAAAGGAAGAACAAACTGAACCAAAGGAAGAACTAGCAGGAATTGATGAGAAATGTAAGTTTTAGTAACAATTATTTATTTTATGATTCCTTTTGAGCTGTTTCGGTATGCTTCGATGCAAGTCAAATTTGTGGATGTTTTAAATCCATCATTCAATTATGTTTGCAATGTAGTCATGAAGTTTGTTCGTTTGTTCAACGCAGATATGGGACATATACGCATGGGCAGACCTTTTTTGTGCACAGATCTTCGTTCAGGTTAATCGTGCATGGCAACAAAAATTGGTTCATTCCGTTTTATGTCGTGCAGTCTTATTTTGCTTGAATTAAGCATTGCTGTAAAGTTCAAGTTAAAGGTACAGTCCATCATTTGTAATTTGTGCATTTTGTTCGTTTGAAGAAACAAACATGCTCAAAATCTAAAGAAGGCTGCTGAACAACTAACCT---------------------------------------------------------------------------------------------------------------------------------------------------------------------------------------------------------------------------------------------------------------------------------------GATGAAGTTTCAGCTCAGCTTCAGCTGTATAAATTATTTCGAGAAAAACAAAATTCTAGGATCTCCATTTCAAATCAAAAGTTGCTGAATCTTGTTTTGAATTGGGCCTGAATTCAGCGTCCGAGGCACGTTCTCATCACTGAGCCGATTGCAGCTACGCGATGTCGCGCACGAATTAAAGCAATCGAGGGCGCGTGTGCGTTGTGTCAACGCTTGCGCGCGCTTCGCCAAATAAAAAAAAAAGAACTAAACGGAAATTTCTTTAGACGCATGTCTTTAGACGCACCGTTGGAAAGGCCTACTTTCGTTGCTTTCTTCAAAATGACAGCATGCCAGAAAGAAAAATCTCTTATCTTTCAACGAAGTATGTTTTTTAACAATAGGCCTAATTACGTCAGTTACTTTTTGGATAGTTGCAAATAATGATGGACTGTAGCTTTAAGAAATTTGTGGAACTCAAGTTGTGCTTGGCAAGTTTTACTTTAGAATTCGAAAATGCATCGACACCAAAAAAAAGTGTAATTTTGGTCCTATAGTTGAAGTGTCAAACTAGAACAAATTTAATGATAAATTAAATAAATTTAAATAATTTATTCCTACCTTGCTTTGACAGTGATGAAGGAGCTGCGTGACCTTTTGGAATCTACGAAGATTGACCTTCCTGTTGACATCAATGATCCATACGACCTAGGTCTTCTTCTCAGACATTTACGTCACCATTCAAATCTTCTTGCTCGTATTGGAGACCCCGATGTCAAAAAGGAAGTCCTCAGCGCCATGAATGAAAAC

>BA72-A

GCACGACGTGCCGCAGAGGAAAAACCTCAAAAGGGAAAGAAGGGACGAAAAGGAAAGAAACGACGAAAGGGAAAGAAGGGACGAAAAGGAAAGAAGGGACGAAAAGGAAAGAAACGACGAAAGGGAAAGAAAAGACGAAAGGGAAAGAAAGGACGAAAGGGAAAGAAAGGACGAAAGGGAAAGAAAGGACGAAAGGGAAAGAAAGGACGAAAGGGAAAGAAAGGACGAAAGGGAAAGAAAGGACGAAAGGGAAAGAAAGGACGAAAGGGAAAGAAAGGA------------------------------------------------------------------------------------------------------------------------------------------------------------------------------------------------------------------------------------------------------------------------------------------------------------------------------------------------------------------------------------------------------------------------------------------------------------------------------------------------------------------------------------------------------------------------------------------------------------------------------------------------------------------------------------------------------------------------------------------------------------------------------------------------------------------------------------------------------------------------------------------------------------------------------------------------------------------------------------------------------------------------------------------------------------------------------------------------------------------------------------------------------------------------------------------------------------------------------------------------------------------------------------------------------------------------------------------------------------------------------------------------------------------------------------------------------------------------------------------------------------------------------------------------------------------------AAACCAATCCAAACTGAAGAAACACCAGCCATTCCGACGGAGATAAAAGCTGCAGAAATAGAAAAGGAACCAAAAACAGAAGTGGTTGTGGAACCACTTATTCAGGAAAAAGATGTTGCGGAGACCGAAATACAACCCATCGAAGCAGAAGTAGAACCAGTTGAACCAAAGACAGAAATAGTTATGGAACCAGTTATTCCGGAAGCAGATATTGCGGAGACCGAAATACAACCCATCAAAGCAGAAGAAGAACCAGTT------------------------------------------------------------------------------------------------------------GAACCAAAGCCAGAAATAGTTATGGAACCAGATGAGGAACCAGTTATTCCGGAAGCAGATATTGCGGAGACCGAAATACAACCCATCAAAGCAGAAGAAGAACCAGTTGAACCAAAGCCAGAAATAGTTATGGAACCAGATGAGGAACCAGTTATTCCGGAAGCAGATATTGCGGAGACCGAAATACAACCCATCAAAGCAGAAGAAGAACCAGTTGAACCAAAGCCAGAAATAGTTATGGAACCAGATGAGGAACCAGTTATTCCGGAAGCAGATATTGCGGAGACCGAAATACAACCCATCAGAGCAGAAGAAGAACCAGTTGAACCAAAGCCAGAAATAGTTATGGAACCAGATGAGGAACCAGTCATTCCAGAAACAGAACTAACAGAGACAGACAAACAACCAATCGAAGCAGAAGTAGAACCAGTTGAACCAAAAACAGAAGTGGTTGTGGAACCAGTTATTCCGGAAGCAGATATTGCGGAGACCGAAATACAACCCATCGAAGCAGAAGTAGAACCAGTTGAACCAAAGCCAGAAATAGTTATGGAACCAGATGTGGAACCAGTAATTCCAGAAACAGATCTTACAGAGACCGAAAAACAACCAATCGAAGCAGAAGTAGAACCAGTTGAACCGAAGACAGAGATAGTCGAACCAGAAACAGAGCCAGTCGAAGAAGCCGAGGAAGAAGCGGTTGAAGCACCCGTTATGGAACCAGTTATACCTGAGATAGAACCAAAGACAGAACCGGAAGTTGAAGCCGAGGAAAAAGAAGTCGAAACAAACGTTGAACCAGAACTACTGGAGACAGGAAAACCGATTGAAATTGGCCAAGCAACTGAAATCGAGGAGCCACCAGAAAAACAACCGGTGGAATCGGAGTCACAACCAGCGGAAGTTGAGATGGAACAAATTGAAACAAAGGAAGAACCAACTGAACCAAAGGAAGAACTAGCAGGAATTGATGAGAAATGTAAGTTTTAGTAACAATTATTTATTTTATGATTCCTTTTGAGCTGTTTCGGTATGCTTCGATGCAAGTCAAATTTGTGGATGTTTTAAATCCATCATTCAATTATGTTTGCAATGTAGTCATGAAGTTTGTTCGTTTGTTCAACACAGATATGGGACATATACGCATGAGCAGACCTTTTTTGTGCACAGATCTTCGTTCAGGTTAATCGTGCATGGCAACAAAAATTGGTTCATTCCGTTTTATGTCGTGCAGTCTTATTTTGCTTGAATTAAGCATTGCTGTAAAGTTCAAGTTAAAGGTACAGTCCATCATTTGTAATTTGTGCATTTTGTTCGTTTGAAGAAACAAACATGCTCAAAATCTAAAGAAGGCTGCTGAACAACTAACCT---------------------------------------------------------------------------------------------------------------------------------------------------------------------------------------------------------------------------------------------------------------------------------------GATGAAGTTTCAGCTCAGCTTCAGCTGTATAAATTATTTCGAGAAAAACAAAATTCTAGGATCTCCATTTCAAATAAAAAGTTGCTGAATCTTGTTTTGAATTGGGCCTGAATTCAGCGTCCGAGGCACGTTCTCATCACTGAGCCGATTGCAGCTACGCGATGTCGCGCACGAATTACAGCAATCGAGGGCGCGTGTGCGTTGTGTCAACGCTTGCGCGCGCTTCGCCAAATAAAAAAAAAAGAACTAAACGGAAATTTCTTTAGACGCATGTCTTTAGACGCACCGTTGGAAAGGCCTACTTTCGTTGCTTTCTTCAAAATGACAGCATGCCAGAAAGAAAAATCTCTTATCTTTCAACGAAGTATGTTTTTTAACAATAGGCCTAATTACGTCAGTTACTTTTTGGATAGTTGCAAATAATGATGGACTGTAGCTTTAAGAAATTTGTGGAACTAAAGTTGTGCTTGGCAAGTTTTACTTTAGAATTCGAAAATGCATCGACACCAAAAAAAAGTGTAATTTTGGTCCTATAGTTGAAGTGTCAAACTAGAACAAATTTAATGATAAATTAAATAAATTTAAATAATTTATTCCTACCTTGCTTTGACAGTGATGAAGGAGCTGCGTGACCTTTTGGAATCTACGAAGATTGACCTTCCTGTTGACATCAATGATCCATACGACCTAGGTCTTCTTCTCAGACATTTACGTCACCATTCAAATCTTCTTGCTCGTATTGGAGACCCCGATGTCAAAAAGGAAGTCCTCAGCGCCATGAATGAAAAC

>BA33-A

GCACGACGTGCCGCAGAGGAAAAACCTCAAAAGGGAAAGAAGGGACGAAAAGGAAAGAAACGACGAAAGGGAAAGAAGGGACGAAAAGGAAAGAAGGGACGAAAAGGAAAGAAACGACGAAAGGGAAAGAAAAGACGAAAGGGAAAGAAAGGACGAAAGGGAAAGAAAGGACGAAAGGGAAAGAAAGGACGAAAGGGAAAGAAAGGACGAAAGGGAAAGAAAGGACGAAAGGGAAAGAAAGGACGAAAGGGAAAGAAAGGACGAAAGGGAAAGAAAGGAAAACCAATCCAACCTGAAGAAACACCAGCCATTCCGACGGAGATAAAAGCTGCAGAAATAGAAAAGGAACCAAAAACAGAAGTGGTTGTGGGACCAGTTATTCCGGAAGCAGATATTGCGGAGACCGAAATACAACAAATTAAAGCAGAAGTAGAACCAGTTGAAACAAAGCCAGAAATAGTTATGGAACCAGATGTGGAACCAGTTATTCCAGAAACGGAACTTACAGAGACCGGAAAAGAAGTGGAAGTAGAACAAGTTAAACCAGTGGAAGGGAAGCTAAACTTA------------------------------------------------------------------------------------------------------------------------------------------------------------------------------------------------------------------------------------------------------------------------------------------------------------------------------------------------------------------------------------------------------------------------------------------------------------------------------GGAAAAGGAAAGGGCAAAGGAAAGAAGGTTAGAAAGGGAAAGAAAGGACGAAAGGGAAAGAAAGGACGAAAGGGAAAGAAAGGACGGAAAGGAAAGAAGGGGCGAAAAGGAAAGAAAAGAAGAAAAGGAAAAAAAGGACGAAAGGGAAAGAAAGGACGAAAGGGAAAGAAAGGACGAAAGGGCAAGAAAGGACGAAAGGGAAAGAAAGGACGAAAGGGAAAGAAAGGACGAAAGGGAAAGAAAGGACGAAAGGGAAAGAAAGGA------------------------------------------------------------------------------------------------------------------------------------------------------------------------------------------------------------------------------------------------------------------------------------------------------------------------------------------------------------------------------------------------------------------------------------------------------------------------------------------------------------------AAACCAATCCAAACTGAAGAAACACCAGCCATTCCGACGGAGATAAAAGCTGCAGAAATAGAAAAGGAACCAAAAACAGAAGTGGTTGTGGAACCACTTATTCAGGAAAAAGATGTTGCGGAGACCGAAATACAACCCATCGAAGCAGAAGTAGAACCAGTTGAACCAAAGACAGAAATAGTTATGGAACCAGTTATTCCGGAAGCAGATATTGCGGAGACCGAAATACAACCCATCAAAGCAGAAGAAGAACCAGTT------------------------------------------------------------------------------------------------------------GAACCAAAGCCAGAAATAGTTATGGAACCAGATGAGGAACCAGTTATTCCGGAAGCAGATATTGCGGAGACCGAAATACAACCCATCAAAGCAGAAGAAGAACCAGTTGAACCAAAGCCAGAAATAGTTATGGAACCAGATGAGGAACCAGTTATTCCGGAAGCAGATATTGCGGAGACCGAAATACAACCCATCAAAGCAGAAGAAGAACCAGTTGAACCAAAGCCAGAAATAGTTATGGAACCAGATGAGGAACCAGTTATTCCGGAAGCAGATATTGCGGAGACCGAAATACAACCCATCAGAGCAGAAGAAGAACCAGTTGAACCAAAGCCAGAAATAGTTATGGAACCAGATGAGGAACCAGTCATTCCAGAAACAGAACTAACAGAGACAGACAAACAACCAATCGAAGCAGAAGTAGAACCAGTTGAACCAAAAACAGAAGTGGTTGTGGAACCAGTTATTCCGGAAGCAGATATTGCGGAGACCGAAATACAACCCATCGAAGCAGAAGTAGAACCAGTTGAACCAAAGCCAGAAATAGTTATGGAACCAGATGTGGAACCAGTAATTCCAGAAACAGATCTTACAGAGACCGAAAAACAACCAATCGAAGCAGAAGTAGAACCAGTTGAACCGAAGACAGAGATAGTCGAACCAGAAACAGAGCCAGTCGAAGAAGCCGAGGAAGAAGCGGTTGAAGCACCCGTTATGGAACCAGTTATACCTGAGATAGAACCAAAGACAGAACCGGAAGTTGAAGCCGAGGAAAAAGAAGTCGAAACAAACGTTGAACCAGAACTACTGGAGACAGGAAAACCGATTGAAATTGGCCAAGCAACTGAAATCGAGGAGCCACCAGAAAAACAACCGGTGGAATCGGAGTCACAACCAGCGGAAGTTGAGATGGAACAAATTGAAACAAAGGAAGAACCAACTGAACCAAAGGAAGAACTAGCAGGAATTGATGAGAAATGTAAGTTTTAGTAACAATTATTTATTTTATGATTCCTTTTGAGCTGTTTCGGTATGCTTCGATGCAAGTCAAATTTGTGGATGTTTTAAATCCATCATTCAATTATGTTTGCAATGTAGTCATGAAGTTTGTTCGTTTGTTCAACACAGATATGGGACATATACGCATGAGCATACCTTTTTTGTGCACAGATCTTCGTTCAGGTTAATCGTGCATGGCAACAAAAAATGGTTCATTCCGTTTTATGTCGTGCAGTCTTATTTTGCTTGAATTAAGCATTGCTGTAAAGTTCAAGTTAAAGGTACAGTCCATCATTTGTAATTTGTGCATTTTGTTCGTTTGAAGAAACAAACGTGCTCAAAATCTAAAGAAGGCTGCTGAACAACTAACCT---------------------------------------------------------------------------------------------------------------------------------------------------------------------------------------------------------------------------------------------------------------------------------------GATGAAGTTTCAGCTCAGCTTCAGCTGTATAAATTATTTCGAGAAAAACAAAATTCTAGGATCTCCATTTCAAATAAAAAGTTGCTGAATCTTGTTTTGAATTGGGCCTGAATTCAGCGTCCGAGGCACGTTCTCATCACTGAGCCGATTGCAGCTACGCGATGTCGCGCACGAATTAAAGCAATCGAGGGCGCGTGTGCGTTGTGTCAACGCTTGCGCGCGCTTCGCCAAATAAAAAAAAAAGAACTAAACGGAAATTTCTTTAGACGCATGTCTTTAGACGCACCGTTGGAAAGGCCTACTTTCGTTGCTTTCTTCAAAATGACAGCATGCCAGAAAGAAAAATCTCTTATCTTTCAACGAAGTATGTTTTTTAACAATAGGCCTAATTACGTCAGTTACTTTTTGGATAGTTGCAAATAATGATGGACTGTAGCTTTAAGAAATTTGTGGAACTAAAGTTGTGCTTGGCAAGTTTTACTTTAGAATTCGAAAATGCATCGACACCAAAAAAAAGTGTAATTTTGGTCCTATAGTTGAAGTGTCAAACTAGAACAAATTTAATGATAAATTAAATAAATTTAAATAATTTATTCCTACCTTGCTCTGACAGTGATGAAGGAGCTGCGTGACCTTTTGGAATCTACGAAGATTGACCTTCCTGTTGACATCAATGATCCATACGACCTAGGTCTTCTTCTCAGACATTTACGTCACCATTCAAATCTTCTTGCTCGTATTGGAGACCCCGATGTCAAAAAGGAAGTCCTCAGCGCCATGAATGAAAAC

>BA78-A

GCACGACGTGCCGCAGAGGAAAAACCTCAAAAGGGAAAGAAGGGACGAAAAGGAAAGAAACGACGAAAGGGAAAGAAGGGACGAAAAGGAAAGAAGGGACGAAAAGGAAAGAAACGACGAAAGGGAAAGAAAAGACGAAAGGGAAAGAAAGGACGAAAGGGAAAGAAAGGACGAAAGGGAAAGAAAGGACGAAAGGGAAAGAAAGGACGAAAGGGAAAGAAAGGACGAAAGGGAAAGAAAGGACGAAAGGGAAAGAAAGGACGAAAGGGAAAGAAAGGAAAACCAATCCAACCTGAAGAAACACCAGCCATTCCGACGGAGATAAAAGCTGCAGAAATAGAAAAGGAACCAAAAACAGAAGTGGTTGTGGGACCAGTTATTCCGGAAGCAGATATTGCGGAGACCGAAATACAACAAATTAAAGCAGAAGTAGAACCAGTTGAAACAAAGCCAGAAATAGTTATGGAACCAGATGTGGAACCAGTTATTCCAGAAACGGAACTTACAGAGACCGGAAAAGAAGTGGAAGTAGAACAAGTTAAACCAGTGGAAGGGAAGCTAAACTTA------------------------------------------------------------------------------------------------------------------------------------------------------------------------------------------------------------------------------------------------------------------------------------------------------------------------------------------------------------------------------------------------------------------------------------------------------------------------------GGAAAAGGAAAGGGCAAAGGAAAGAAGGTTAGAAAGGGAAAGAAAGGACGAAAGGGAAAGAAAGGACGAAAGGGAAAGAAAGGACGGAAAGGAAAGAAGGGGCGAAAAGGAAAGAAAAGAAGAAAAGGAAAAAAAGGACGAAAGGGAAAGAAAGGACGAAAGGGAAAGAAAGGACGAAAGGGCAAGAAAGGACGAAAGGGAAAGAAAGGACGAAAGGGAAAGAAAGGACGAAAGGGAAAGAAAGGACGAAAGGGAAAGAAAGGA------------------------------------------------------------------------------------------------------------------------------------------------------------------------------------------------------------------------------------------------------------------------------------------------------------------------------------------------------------------------------------------------------------------------------------------------------------------------------------------------------------------AAACCAATCCAAACTGAAGAAACACCAGCCATTCCGACGGAGATAAAAGCTGCAGAAATAGAAAAGGAACCAAAAACAGAAGTGGTTGTGGAACCACTTATTCAGGAAAAAGATGTTGCGGAGACCGAAATACAACCCATCGAAGCAGAAGTAGAACCAGTTGAACCAAAGACAGAAATAGTTATGGAACCAGTTATTCCGGAAGCAGATATTGCGGAGACCGAAATACAACCCATCAAAGCAGAAGAAGAACCAGTT------------------------------------------------------------------------------------------------------------GAACCAAAGCCAGAAATAGTTATGGAACCAGATGAGGAACCAGTTATTCCGGAAGCAGATATTGCGGAGACCGAAATACAACCCATCAAAGCAGAAGAAGAACCAGTTGAACCAAAGCCAGAAATAGTTATGGAACCAGATGAGGAACCAGTTATTCCGGAAGCAGATATTGCGGAGACCGAAATACAACCCATCAAAGCAGAAGAAGAACCAGTTGAACCAAAGCCAGAAATAGTTATGGAACCAGATGAGGAACCAGTTATTCCGGAAGCAGATATTGCGGAGACCGAAATACAACCCATCAAAGCAGAAGAAGAACCAGTTGAACCAAAGCCAGAAATAGTTATGGAACCAGATGAGGAACCAGTTATTCCAGAAACAGAACTAACAGAGACAGACAAACAACCAATCGAAGCAGAAGTAGAACCAGTTGAACCAAAAACAGAAGTGGTTGTGGAACCAGTTATTCCGGAAGCAGATATTGCGGAGACCGAAATACAACCCATCGAAGCAGAAGTAGAACCAGTTGAACCAAAGCCAGAAATAGTTATGGAACCAGATGTGGAACCAGTAATTCCAGAAACAGATCTTACAGAGACCGAAAAACAACCAATCGAAGCAGAAGTAGAACCAGTTGAACCGAAGACAGAGATAGTCGAACCAGAAACAGAGCCAGTCGAAGAAGCCGAGGAAGAAGCGGTTGAAGCACCCGTTATGGAACCAGTTATACCTGAGATAGAACCAAAGACAGAACCGGAAGTTGAAGCCGAGGAAAAAGAAGTCGAAACAAACGTTGAACCAGAACTACTGGAGACAGGAAAACCGATTGAAATTGGCCAAGCAACTGAAATCGAGGAGCCACCAGAAAAACAACCGGTGGAATCGGAGTCACAACCAGCGGAAGTTGAGATGGAACAAATTGAAACAAAGGAAGAACCAACTGAACCAAAGGAAGAACTAGCAGGAATTGATGAGAAATGTAAGTTTTAGTAACAATTATTTATTTTATGATTCCTTTTGAGCTGTTTCGGTATGCTTCGATGCAAGTCAAATTTGTGGATGTTTTAAATCCATCATTCAATTATGTTTGCAATGTAGTCATGAAGTTTGTTCGTTTGTTCAACACAGATATGGGACATATACGCATGAGCATACCTTTTTTGTGCACAGATCTTCGTTCAGGTTAATCGTGCATGGCAACAAAAAATGGTTCATTCCGTTTTATGTCGTGCAGTCTTATTTTGCTTGAATTAAGCATTGCTGTAAAGTTCAAGTTAAAGGTACAGTCCATCATTTGTAATTTGTGCATTTTGTTCGTTTGAAGAAACAAACATGCTCAAAATCTAAAGAAGGCTGCTGAACAACTAACCT---------------------------------------------------------------------------------------------------------------------------------------------------------------------------------------------------------------------------------------------------------------------------------------GATGAAGTTTCAGCTCAGCTTCAGCTGTATAAATTATTTCGAGAAAAACAAAATTCTAGGATCTCCATTTCAAATAAAAAGTTGCTGAATCTTGTTTTGAATTGGGTCTGAATTCAGCGTCCGAGGCACGTTCTCATCACTGAGCCGATTGCAGCTACGCGATGTCGCGCACGAATTAGAGCAATCGAGGGCGCGTGTGCGTTGTGTCAACGCTTGCGCGCGCTTCGCCAAAT-AAAAAAAAAGAACTAAACGGAAATTTCTTTAGACGCATGTCTTTAGACGCACCGTTGGAAAGGCCTACTTTCGTTGCTTTCTTCAAAATGACAGCATGCCAGAAAGAAAAATCTCTTATCTTTCAACGAAGTATGTTTTTTAACAATAGGCCTAATTACGTCAGTTACTTTTTGGATAGTTGCAAATAATGATGGACTGTAGCTTTAAGAAATTTGTGGAACTAAGGTTGTGCTTGGCAAGTTTTACTTTAGAATTCGAAAATGCATCGACACCAAAAAAAAGTGTAATTTTGGTCCTATAGTTGAAGTGTCAAACTAGAACAAATTTAATGATAAATTAAATAAATTTAAATAATTTATTCCTACCTTGCTTTGACAGTGATGAAGGAGCTGCGTGACCTTTTGGAATCTACGAAGATTGACCTTCCTGTTGACATCAATGATCCATACGACCTAGGTCTTCTTCTCAGACATTTACGTCACCATTCAAATCTTCTTGCTCGTATTGGAGACCCCGATGTCAAAAAGGAAGTCCTCAGCGCCATGAATGAAAAC

>BA78-B

GCACGACGTGCCGCAGAGGAAAAACCTCAAAAGGGAAAGAAGGGACGAAAAGGAAAGAAACGACGAAAGGGAAAGAAGGGACGAAAAGGAAAGAAGGGACGAAAAGGAAAGAAACGACGAAAGGGAAAGAAAAGACGAAAGGGAAAGAAAGGACGAAAGGGAAAGAAAGGACGAAAGGGAAAGAAAGGACGAAAGGGAAAGAAAGGACGAAAGGGAAAGAAAGGACGAAAGGGAAAGAAAGGACGAAAGGGAAAGAAAGGACGAAAGGGAAAGAAAGGAAAACCAATCCAACCTGAAGAAACACCAGCCATTCCGACGGAGATAAAAGCTGCAGAAATAGAAAAGGAACCAAAAACAGAAGTGGTTGTGGGACCAGTTATTCCGGAAGCAGATATTGCGGAGACCGAAATACAACAAATTAAAGCAGAAGTAGAACCAGTTGAAGCAAAGCCAGAAATAGTTATGGAACCAGATGTGGAACCAGTTATTCCAGAAACGGAACTTACAGAGACCGGAAAAGAAGTGGAAGTAGAACAAGTTAAACCAGTGGAAGGGAAGCTAAACTTA------------------------------------------------------------------------------------------------------------------------------------------------------------------------------------------------------------------------------------------------------------------------------------------------------------------------------------------------------------------------------------------------------------------------------------------------------------------------------GGAAAAGGAAAGGGCAAAGGAAAGAAGGTTAGAAAGGGAAAGAAAGGACGAAAGGGAAAGAAAGGACGAAAGGGAAAGAAAGGACGGAAAGGAAAGAAGGGGCGAAAAGGAAAGAAAAGAAGAAAAGGAAAAAAAGGACGAAAGGGAAAGAAAGGACGAAAGGGAAAGAAAGGACGAAAGGGCAAGAAAGGACGAAAGGGAAAGAAAGGACGAAAGGGAAAGAAAGGACGAAAGGGAAAGAAAGGACGAAAGGGAAAGAAAGGA------------------------------------------------------------------------------------------------------------------------------------------------------------------------------------------------------------------------------------------------------------------------------------------------------------------------------------------------------------------------------------------------------------------------------------------------------------------------------------------------------------------AAACCAATCCAAACTGAAGAAACACCAGCCATTCCGACGGAGATAAAAGCTGCAGAAATAGAAAAGGAACCAAAAACAGAAGTGGTTGTGGAACCACTTATTCAGGAAAAAGATGTTGCGGAGACCGAAATACAACCCATCGAAGCAGAAGTAGAACCAGTTGAACCAAAGACAGAAATAGTTATGGAACCAGTTATTCCGGAAGCAGATATTGCGGAGACCGAAATACAACCCATCAAAGCAGAAGAAGAACCAGTT------------------------------------------------------------------------------------------------------------GAACCAAAGCCAGAAATAGTTATGGAACCAGATGAGGAACCAGTTATTCCGGAAGCAGATATTGCGGAGACCGAAATACAACCCATCAAAGCAGAAGAAGAACCAGTTGAACCAAAGCCAGAAATAGTTATGGAACCAGATGAGGAACCAGTTATTCCGGAAGCAGATATTGCGGAGACCGAAATACAACCCATCAAAGCAGAAGAAGAACCAGTTGAACCAAAGCCAGAAATAGTTATGGAACCAGATGAGGAACCAGTTATTCCGGAAGCAGATATTGCGGAGACCGAAATACAACCCATCAAAGCAGAAGAAGAACCAGTTGAACCAAAGCCAGAAATAGTTATGGAACCAGATGAGGAACCAGTTATTCCAGAAACAGAACTAACAGAGACAGACAAACAACCAATCGAAGCAGAAGTAGAACCAGTTGAACCAAAAACAGAAGTGGTTGTGGAACCAGTTATTCCGGAAGCAGATATTGCGGAGACCGAAATACAACCCATCGAAGCAGAAGTAGAACCAGTTGAACCAAAGCCAGAAATAGTTATGGAACCAGATGTGGAACCAGTAATTCCAGAAACAGATCTTACAGAGACCGAAAAACAACCAATCGAAGCAGAAGTAGAACCAGTTGAACCGAAGACAGAGATAGTCGAACCAGAAACAGAGCCAGTCGAAGAAGCCGAGGAAGAAGCGGTTGAAGCACCCGTTATGGAACCAGTTATACCTGAGATAGAACCAAAGACAGAACCGGAAGTTGAAGCCGAGGAAAAAGAAGTCGAAACAAACGTTGAACCAGAACTACTGGAGACAGGAAAACCGATTGAAATTGGCCAAGCAACTGAAATCGAGGAGCCACCAGAAAAACAACCGGTGGAATCGGAGTCACAACCAGCGGAAGTTGAGATGGAACAAATTGAAACAAAGGAAGAACCAACTGAACCAAAGGAAGAACTAGCAGGAATTGATGAGAAATGTAAGTTTTAGTAACAATTATTTATTTTATGATTCCTTTTGAGCTGTTTCGGTATGCTTCGATGCAAGTCAAATTTGTGGATGTTTTAAATCCATCATTCAATTATGTTTGCAATGTAGTCATGAAGTTTGTTCGTTTGTTCAACACAGATATGGGACATATACGCATGAGCATACCTTTTTTGTGCACAGATCTTCGTTCAGGTTAATCGTGCATGGCAACAAAAAATGGTTCATTCCGTTTTATGTCGTGCAGTCTTATTTTGCTTGAATTAAGCATTGCTGTAAAGTTCAAGTTAAAGGTACAGTCCATCATTTGTAATTTGTGCATTTTGTTCGTTTGAAGAAACAAACATGCTCAAAATCTAAAGAAGGCTGCTGAACAACTAACCT---------------------------------------------------------------------------------------------------------------------------------------------------------------------------------------------------------------------------------------------------------------------------------------GATGAAGTTTCAGCTCAGCTTCAGCTGTATAAATTATTTCGAGAAAAACAAAATTCTAGGATCTCCATTTCAAATAAAAAGTTGCTGAATCTTGTTTTGAATTGGGCCTGAATTCAGCGTCCGAGGCACGTTCTCATCACTGAGCCGATTGCAGCTACGCGATGTCGCGCACGAATTAAAGCAATCGAGGGCGCGTGTGCGTTGTGTCAACGCTTGCGCGCGCTTCGCCAAAT-AAAAAAAAAGAACTAAACGGAAATTTCTTTAGACGCATGTCTTTAGACGCACCGTTGGAAAGGCCTACTTTCGTTGCTTTCTTCAAAATGACAGCATGCCAGAAAGAAAAATCTCTTATCTTTCAACGAAGTATGTTTTTTAACAATAGGCCTAATTACGTCAGTTACTTTTTGGATAGTTGCAAATAATGATGGACTGTAGCTTTAAGAAATTTGTGGAACTAAAGTTGTGCTTGGCAAGTTTTACTTTAGAATTCGAAAACGCATCGACACCAAAAAAAAGTGTAATTTTGGTCCTATAGTTGAAGTGTCAAACTAGAACAAATTTAATGATAAATTAAATAAATTTAAATAATTTATTCCTACCTTGCTTTGACAGTGATGAAGGAGCTGCGTGACCTTTTGGAATCTACGAAGATTGACCTTCCTGTTGACATCAATGATCCATACGACCTAGGTCTTCTTCTCAGACATTTACGTCACCATTCAAATCTTCTTGCTCGTATTGGAGACCCCGATGTCAAAAAGGAAGTCCTCAGCGCCATGAATGAAAAC

>BA86-A

GCACGACGTGCCGCAGAGGAAAAACCTCAAAAGGGAAAGAAGGGACGAAAAGGAAAGAAACGACGAAAGGGAAAGAAGGGACGAAAAGGAAAGAAGGGACGAAAAGGAAAGAAACGACGAAAGGGAAAGAAAAGACGAAAGGGAAAGAAAGGACGAAAGGGAAAGAAAGGACGAAAGGGAAAGAAAGGACGAAAGGGAAAGAAAGGACGAAAGGGAAAGAAAGGACGAAAGGGAAAGAAAGGACGAAAGGGAAAGAAAGGACGAAAGGGAAAGAAAGGAAAACCAATCCAACCTGAAGAAACACCAGCCATTCCGACGGAGATAAAAGCTGCAGAAATAGAAAAGGAACCAAAAACAGAAGTGGTTGTGGGACCAGTTATTCCGGAAGCAGATATTGCGGAGACCGAAATACAACAAATTAAAGCAGAAGTAGAACCAGTTGAAACAAAGCCAGAAATAGTTATGGAACCAGATGTGGAACCAGTTATTCCAGAAACGGAACTTACAGAGACCGGAAAAGAAGTGGAAGTAGAACAAGTTAAACCAGTGGAAGGGAAGCTAAACTTA------------------------------------------------------------------------------------------------------------------------------------------------------------------------------------------------------------------------------------------------------------------------------------------------------------------------------------------------------------------------------------------------------------------------------------------------------------------------------GGAAAAGGAAAGGGCAAAGGAAAGAAGGTTAGAAAGGGAAAGAAAGGACGAAAGGGAAAGAAAGGACGAAAGGGAAAGAAAGGACGGAAAGGAAAGAAGGGGCGAAAAGGAAAGAAAAGAAGAAAAGGAAAAAAAGGACGAAAGGGAAAGAAAGGACGAAAGGGAAAGAAAGGACGAAAGGGCAAGAAAGGACGAAAGGGAAAGAAAGGACGAAAGGGAAAGAAAGGACGAAAGGGAAAGAAAGGACGAAAGGGAAAGAAAGGA------------------------------------------------------------------------------------------------------------------------------------------------------------------------------------------------------------------------------------------------------------------------------------------------------------------------------------------------------------------------------------------------------------------------------------------------------------------------------------------------------------------AAACCAATCCAAACTGAAGAAACACCAGCCATTCCGACGGAGATAAAAGCTGCAGAAATAGAAAAGGAACCAAAAACAGAAGTGGTTGTGGAACCACTTATTCAGGAAAAAGATGTTGCGGAGACCGAAATACAACCCATCGAAGCAGAAGTAGAACCAGTTGAACCAAAGACAGAAATAGTTATGGAACCAGTTATTCCGGAAGCAGATATTGCGGAGACCGAAATACAACCCATCAAAGCAGAAGAAGAACCAGTT------------------------------------------------------------------------------------------------------------GAACCAAAGCCAGAAATAGTTATGGAACCAGATGAGGAACCAGTTATTCCGGAAGCAGATATTGCGGAGACCGAAATACAACCCATCAAAGCAGAAGAAGAACCAGTTGAACCAAAGCCAGAAATAGTTATGGAACCAGATGAGGAACCAGTTATTCCGGAAGCAGATATTGCGGAGACCGAAATACAACCCATCAAAGCAGAAGAAGAACCAGTTGAACCAAAGCCAGAAATAGTTATGGAACCAGATGAGGAACCAGTTATTCCGGAAGCAGATATTGCGGAGACCGAAATACAACCCATCAGAGCAGAAGAAGAACCAGTTGAACCAAAGCCAGAAATAGTTATGGAACCAGATGAGGAACCAGTCATTCCAGAAACAGAACTAACAGAGACAGACAAACAACCAATCGAAGCAGAAGTAGAACCAGTTGAACCAAAAACAGAAGTGGTTGTGGAACCAGTTATTCCGGAAGCAGATATTGCGGAGACCGAAATACAACCCATCGAAGCAGAAGTAGAACCAGTTGAACCAAAGCCAGAAATAGTTATGGAACCAGATGTGGAACCAGTAATTCCAGAAACAGATCTTACAGAGACCGAAAAACAACCAATCGAAGCAGAAGTAGAACCAGTTGAACCGAAGACAGAGATAGTCGAACCAGAAACAGAGCCAGTCGAAGAAGCCGAGGAAGAAGCGGTTGAAGCACCCGTTATGGAACCAGTTATACCTGAGATAGAACCAAAGACAGAACCGGAAGTTGAAGCCGAGGAAAAAGAAGTCGAAACAAACGTTGAACCAGAACTACTGGAGACAGGAAAACCGATTGAAATTGGCCAAGCAACTGAAATCGAGGAGCCACCAGAAAAACAACCGGTGGAATCGGAGTCACAACCAGCGGAAGTTGAGATGGAACAAATTGAAACAAAGGAAGAACCAACTGAACCAAAGGAAGAACTAGCAGGAATTGATGAGAAATGTAAGTTTTAGTAACAATTATTTATTTTATGATTCCTTTTGAGCTGTTTCGGTATGCTTCGATGTAAGTCAAATTTGTGGATGTTTTAAATCCATCATTCAATTATGTTTGCAATGTAGTCATGAAGTTTGTTCGTTTGTTCAACACAGATATGGGACATATACGCATGAGCATACCTTTTTTGTGCACAGATCTTCGTTCAGGTTAATCGTGCATGGCAACAAAAAATGGTTCATTCCGTTTTATGTCGTGCAGTCTTATTTTGCTTGAATTAAGCATTGCTGTAAAGTTCAAGTTAAAGGTACAGTCCATCATTTGTAATTTGTGCATTTTGTTCGTTTGAAGAAACAAACATGCTCAAAATCTAAAGAAGGCTGCTGAACAACTAACCT---------------------------------------------------------------------------------------------------------------------------------------------------------------------------------------------------------------------------------------------------------------------------------------GATGAAGTTTCAGCTCAGCTTCAGCTGTATAAATTATTTCGAGAAAAACAAAATTCTAGGATCTCCATTTCAAATAAAAAGTTGCTGAATCTTGTTTTGAATTGGGCCTGAATTCAGCGTCCGAGGCACGTTCTCATCACTGAGCCGATTGCAGCTACGCGGTGTCGCGCACGAATTAAAGCAATCGAGGGCGCGTGTGCGTTGTGTCAACGCTTGCGCGCGCTTCGCCAAATAAAAAAAAAAGAACTAAACGGAAATTTCTTTAGACGCATGTCTTTAGACGCACCGTTGGAAAGGCCTACTTTCGTTGCTTTCTTCNAAATGACAGCATGCCAGAAAGAAAAATCTCTTATCTTTCACCGAAGTATGTTTTTTAACAATAGGCCTAATTACGTCAGTTACTTTTTGGATAGTTGCAAATAATGATGGACTGTAGCTTTAAGAAATTTGTGGAACTAAAGTTGTGCTTGGCAAGTTTTACTTTAGAATTCGAAAATGCATCGACACCAAAAAAAAGTGTAATTTTGGTCCTATAGTTGAAGTGTCAAACTAGAACAAATTTAATGATAAATTAAATAAATTTAAATAATTTATTCCTACCTTGCTTTGACAGTGATGAAGGAGCTGCGTGACCTTTTGGAATCTACGAAGATTGACCTTCCTGTTGACATCAATGATCCATACGACCTAGGTCTTCTTCTCAGACATTTACGTCACCATTCAAATCTTCTTGCTCGTATTGGAGACCCCGATGTCAAAAAGGAAGTCCTCAGCGCCATGAATGAAAAC

>BA31-A

GCACGACGTGCCGCAGAGGAAAAACCTCAAAAGGGAAAGAAGGGACGAAAAGGAAAGAAACGACGAAAGGGAAAGAAGGGACGAAAAGGAAAGAAGGGACGAAAAGGAAAGAAACGACGAAAGGGAAAGAAAAGACGAAAGGGAAAGAAAGGACGAAAGGGAAAGAAAGGACGAAAGGGAAAGAAAGGACGAAAGGGAAAGAAAGGACGAAAGGGAAAGAAAGGACGAAAGGGAAAGAAAGGACGAAAGGGAAAGAAAGGACGAAAGGGAAAGAAAGGAAAACCAATCCAACCTGAAGAAACACCAGCCATTCCGACGGAGATAAAAGCTGCAGAAATAGAAAAGGAACCAAAAACAGAAGTGGTTGTGGGACCAGTTATTCCGGAAGCAGATATTGCGGAGACCGAAATACAACAAATTAAAGCAGAAGTAGAACCAGTTGAAACAAAGCCAGAAATAGTTATGGAACCAGATGTGGAACCAGTTATTCCAGAAACGGAACTTACAGAGACCGGAAAAGAAGTGGAAGTAGAACAAGTTAAACCAGTGGAAGGGAAGCTAAACTTA------------------------------------------------------------------------------------------------------------------------------------------------------------------------------------------------------------------------------------------------------------------------------------------------------------------------------------------------------------------------------------------------------------------------------------------------------------------------------GGAAAAGGAAAGGGCAAAGGAAAGAAGGTTAGAAAGGGAAAGAAAGGACGAAAGGGAAAGAAAGGACGAAAGGGAAAGAAAGGACGGAAAGGAAAGAAGGGGCGAAAAGGAAAGAAAAGAAGAAAAGGAAAAAAAGGACGAAAGGGAAAGAAAGGACGAAAGGGAAAGAAAGGACGAAAGGGCAAGAAAGGACGAAAGGGAAAGAAAGGACGAAAGGGAAAGAAAGGACGAAAGGGAAAGAAAGGACGAAAGGGAAAGAAAGGA------------------------------------------------------------------------------------------------------------------------------------------------------------------------------------------------------------------------------------------------------------------------------------------------------------------------------------------------------------------------------------------------------------------------------------------------------------------------------------------------------------------AAACCAATCCAAACTGAAGAAACACCAGCCATTCCGACGGAGATAAAAGCTGCAGAAATAGAAAAGGAACCAAAAACAGAAGTGGTTGTGGAACCACTTATTCAGGAAAAAGATGTTGCGGAGACCGAAATACAACCCATCGAAGCAGAAGTAGAACCAGTTGAACCAAAGACAGAAATAGTTATGGAACCAGTTATTCCGGAAGCAGATATTGCGGAGACCGAAATACAACCCATCAAAGCAGAAGAAGAACCAGTT------------------------------------------------------------------------------------------------------------GAACCAAAGCCAGAAATAGTTATGGAACCAGATGAGGAACCAGTTATTCCGGAAGCAGATATTGCGGAGACCGAAATACAACCCATCAAAGCAGAAGAAGAACCAGTTGAACCAAAGCCAGAAATAGTTATGGAACCAGATGAGGAACCAGTTATTCCGGAAGCAGATATTGCGGAGACCGAAATACAACCCATCAAAGCAGAAGAAGAACCAGTTGAACCAAAGCCAGAAATAGTTATGGAACCAGATGAGGAACCAGTTATTCCGGAAGCAGATATTGCGGAGACCGAAATACAACCCATCAGAGCAGAAGAAGAACCAGTTGAACCAAAGCCAGAAATAGTTATGGAACCAGATGAGGAACCAGTCATTCCAGAAACAGAACTAACAGAGACAGACAAACAACCAATCGAAGCAGAAGTAGAACCAGTTGAACCAAAAACAGAAGTGGTTGTGGAACCAGTTATTCCGGAAGCAGATATTGCGGAGACCGAAATACAACCCATCGAAGCAGAAGTAGAACCAGTTGAACCAAAGCCAGAAATAGTTATGGAACCAGATGTGGAACCAGTAATTCCAGAAACAGATCTTACAGAGACCGAAAAACAACCAATCGAAGCAGAAGTAGAACCAGTTGAACCGAAGACAGAGATAGTCGAACCAGAAACAGAGCCAGTCGAAGAAGCCGAGGAAGAAGCGGTTGAAGCACCCGTTATGGAACCAGTTATACCTGAGATAGAACCAAAGACAGAACCGGAAGTTGAAGCCGAGGAAAAAGAAGTCGAAACAAACGTTGAACCAGAACTACTGGAGACAGGAAAACCGATTGAAATTGGCCAAGCAACTGAAATCGAGGAGCCACCAGAAAAACAACCGGTGGAATCGGAGTCACAACCAGCGGAAGTTGAGATGGAACAAATTGAAACAAAGGAAGAACCAACTGAACCAAAGGAAGAACTAGCAGGAATTGATGAGAAATGTAAGTTTTAGTAACAATTATTTATTTTATGATTCCTTTTGAGCTGTTTCGGTATGCTTCGATGCAAGTCAAATTTGTGGATGTTTTAAATCCATCATTCAATTATGTTTGCAATGTAGTCATGAAGTTTGTTCGTTTGTTCAACACAGATATGGGACATATACGCATGAGCATACCTTTTTTGTGCACAGATCTTCGTTCAGGTTAATCGTGCATGGCAACAAAAAATGGTTCATTCCGTTTTATGTCGTGCAGTCTTATTTTGCTTGAATTAAGCATTGCTGTAAAGTTCAAGTTAAAGGTACAGTCCATCATTTGTAATTTGTGCATTTTGTTCGTTTGAAGAAACAAACATGCTCAAAATCTAAAGAAGGCTGCTGAACAACTAACCT---------------------------------------------------------------------------------------------------------------------------------------------------------------------------------------------------------------------------------------------------------------------------------------GATGAAGTTTCAGCTCAGCTTCAGCTGTATAAATTATTTCGAGAAAAACAAAATTCTAGGATCTCCATTTCAAATAAAAAGTTGCTGAATCTTGTTTTGAATTGGGCCTGAATTCAGCGTCCGAGGCACGTTCTCATCACTGAGCCGATTGCAGCTACGCGATGTCGCGCACGAATTAAAGCAATCGAGGGCGCGTGTGCGTTGTGTCAACGCTTGCGCGCGCTTCGCCAAAT-AAAAAAAAAGAACTAAACGGAAATTTCTTTAGACGCATGTCTTTAGACGCACCGTTGGAAAGGCCTACTTTCGTTGCTTTCTTCAAAATGACAGCATGCCAGAAAGAAAAATCTCTTATCTTTCAACGAAGTATGTTTTTTAACAATAGGCCTAATTACGTCAGTTACTTTTTGGATAGTTGCAAATAATGATGGACTGTAGCTTTAAGAAATTTGTGGAACTAAAGTTGTGCTTGGCAAGTTTTACTTTAGAATTCGAAAATGCATCGACACCAAAAAAAAGTGTAATTTTGGTCCTATAGTTGAAGTGTCAAACTAGAACAAATTTAATGATAAATTAAATAAATTTAAATAATTTATTCCTACCTTGCTTTGACAGTGATGAAGGAGCTGCGTGACCTTTTGGAATCTACGAAGATTGACCTTCCTGTTGACATCAATGATCCATACGACCTAGGTCTTCTTCTCAGACATTTACGTCACCATTCAAATCTTCTTGCTCGTATTGGAGACCCCGATGTCAAAAAGGAAGTCCTCAGCGCCATGAATGAAAAC

>BA55-B

GCACGACGTGCCGCAGAGGAAAAACCTCAAAAGGGAAAGAAGGGACGAAAAGGAAAGAAACGACGAAAGGGAAAGAAGGGACTAAAAGGAAAGAAGGGACGAAAAGGAAAGAAACGACGAAAGGGAAAGAAAAGACGAAAGGGAAAGAAAGGACGAAAGGGAAAGAAAGGACGAAAGGGAAAGAAAGGACGAAAGGGAAAGAAAGGACGAAAGGGAAAGAAAGGACGAAAGGGAAAGAAAGGACGAAAGGGAAAGAAAGGACGAAAGGGAAAGAAAGGAAAACCAATCCAACCTGAAGAAACACCAGCCATTCCGACGGAGATAAAAGCTGCAGAAATAGAAAAGGAACCAAAAACAGAAGTGGTTGTGGGACCAGTTATTCCGGAAGCAGATATTGCGGAGACCGAAATACAACAAATTAAAGCAGAAGTAGAACCAGTTGAAACAAAGCCAGAAATAGTTATGGAACCAGATGTGGAACCAGTTATTCCAGAAACGGAACTTACAGAGACCGGAAAAGAAGTGGAAGTAGAACAAGTTAAACCAGTGGAAGGGAAGCTAAACTTA------------------------------------------------------------------------------------------------------------------------------------------------------------------------------------------------------------------------------------------------------------------------------------------------------------------------------------------------------------------------------------------------------------------------------------------------------------------------------GGAAAAGGAAAGGGCAAAGGAAAGAAGGTTAGAAAGGGAAAGAAAGGACGAAAGGGAAAGAAAGGACGAAAGGGAAAGAAAGGACGGAAAGGAAAGAAGGGGCGAAAAGGAAAGAAAAGAAGAAAAGGAAAAAAAGGACGAAAGGGAAAGAAAGGACGAAAGGGAAAGAAAGGACGAAAGGGCAAGAAAGGACGAAAGGGAAAGAAAGGACGAAAGGGAAAGAAAGGACGAAAGGGAAAGAAAGGACGAAAGGGAAAGAAAGGA------------------------------------------------------------------------------------------------------------------------------------------------------------------------------------------------------------------------------------------------------------------------------------------------------------------------------------------------------------------------------------------------------------------------------------------------------------------------------------------------------------------AAACCAATCCAAACTGAAGAAACACCAGCCATTCCGACGGAGATAAAAGCTGCAGAAATAGAAAAGGAACCAAAAACAGAAGTGGTTGTGGAACCACTTATTCAGGAAAAAGATGTTGCGGAGACCGAAATACAACCCATCGAAGCAGAAGTAGAACCAGTTGAACCAAAGACAGAAATAGTTATGGAACCAGTTATTCCGGAAGCAGATATTGCGGAGACCGAAATACAACCCATCAAAGCAGAAGAAGAACCAGTT------------------------------------------------------------------------------------------------------------GAACCAAAGCCAGAAATAGTTATGGAACCAGATGAGGAACCAGTTATTCCGGAAGCAGATATTGCGGAGACCGAAATACAACCCATCAAAGCAGAAGAAGAACCAGTTGAACCAAAGCCAGAAATAGTTATGGAACCAGATGAGGAACCAGTTATTCCGGAAGCAGATATTGCGGAGACCGAAATACAACCCATCAAAGCAGAAGAAGAACCAGTTGAACCAAAGCCAGAAATAGTTATGGAACCAGATGAGGAACCAGTTATTCCGGAAGCAGATATTGCGGAGACCGAAATACAACCCATCAGAGCAGAAGAAGAACCAGTTGAACCAAAGCCAGAAATAGTTATGGAACCAGATGAGGAACCAGTCATTCCAGAAACAGAACTAACAGAGACAGACAAACAACCAATCGAAGCAGAAGTAGAACCAGTTGAACCAAAAACAGAAGTGGTTGTGGAACCAGTTATTCCGGAAGCAGATATTGCGGAGACCGAAATACAACCCATCGAAGCAGAAGTAGAACCAGTTGAACCAAAGCCAGAAATAGTTATGGAACCAGATGTGGAACCAGTAATTCCAGAAACAGATCTTACAGAGACCGAAAAACAACCAATCGAAGCAGAAGTAGAACCAGTTGAACCGAAGACAGAGATAGTCGAACCAGAAACAGAGCCAGTCGAAGAAGCCGAGGAAGAAGCGGTTGAAGCACCCGTTATGGAACCAGTTATACCTGAGATAGAACCAAAGACAGAACCGGAAGTTGAAGCCGAGGAAAAAGAAGTCGAAACAAACGTTGAACCAGAACTACTGGAGACAGGAAAACCGATTGAAATTGGCCAAGCAACTGAAATCGAGGAGCCACCAGAAAAACAACCGGTGGAATCGGAGTCACAACCAGCGGAAGTTGAGATGGAACAAATTGAAACAAAGGAAGAACCAACTGAACCAAAGGAAGAACTAGCAGGAATTGATGAGAAATGTAAGTTTTAGTAACAATTATTTATTTTATGATTCCTTTTGAGCTGTTTCGGTATGCTTCGATGCAAGTCAAATTTGTGGATGTTTTAAATCCATCATTCAATTATGTTTGCAATGTAGTCATGAAGTTTGTTCGTTTGTTCAACACAGATATGGGACATATACGCATGAGCATACCTTTTTTGTGCACAGATCTTCGTTCAGGTTAATCGTGCATGGCAACAAAAAATGGTTCATTCCGTTTTATGTCGTGCAGTCTTATTTTGCTTGAATTAAGCATTGCTGTAAAGTTCAAGTTAAAGGTACAGTCCATCATTTGTAATTTGTGCATTTTGTTCGTTTGAAGAAACAAACATGCTCAAAATCTAAAGAAGGCTGCTGAACAACTAACCT---------------------------------------------------------------------------------------------------------------------------------------------------------------------------------------------------------------------------------------------------------------------------------------GATGAAGTTTCAGCCCAGCTTCAGCTGTATAAATTATTTCGAGAAAAACAAAATTCTAGGATCTCCATTTCAAATAAAAAGTTGCTGAATCTTGTTTTGAATTGGGCCTGAATTCAGCGTCCGAGGCACGTTCTCATCACTGAGCCGATTGCAGCTACGCGATGTCGCGCACGAATTAAAGCAATCGAGGGCGCGTGTGCGTTGTGTCAACGCTTGCGCGCGCTTCGCCAAATAAAAAAAAAAGAACTAAACGGAAATTTCTTTAGACGCATGTCTTTAGACGCACCGTTGGAAAGGCCTACTTTCGTTGCTTTCTTCAAAATGACAGCATGCCAGAAAGAAAAATCTCTTATCTTTCAACGAAGTATGTTTTTTAACAATAGGCCTAATTACGTCAGTTACTTTTTGGATAGTTGCAAATAATGATGGACTGTAGCTTTAAGAAATTTGTGGAACTAAAGTTGTGCTTGGCAAGTTTTACTTTAGAATTCGAAAATGCATCGACACCAAAAAAAAGTGTAATTTTGGTCCTATAGTTGAAGTGTCAAACTAGAACAAATTTAATGATAAATTAAATAAATTTAAATAATTTATTCCTACCTTGCTTTGACAGTGATGAAGGAGCTGCGTGACCTTTTGGAATCTACGAAGATTGACCTTCCTGTTGACATCAATGATCCATACGACCTAGGTCTTCTTCTCAGACATTTACGTCACCATTCAAATCTTCTTGCTCGTATTGGAGACCCCGATGTCAAAAAGGAAGTCCTCAGCGCCATGAATGAAAAC

>BA58-B

GCACGACGTGCCGCAGAGGAAAAACCTCAAAAGGGAAAGAAGGGACGAAAAGGAAAGAAACGACGAAAGGGAAAGAAGGGACGAAAAGGAAAGAAGGGACGAAAAGGAAAGAAACGACGAAAGGGAAAGAAAAGACGAAAGGGAAAGAAAGGACGAAAGGGAAAGAAAGGACGAAAGGGAAAGAAAGGACGAAAGGGAAAGAAAGGACGAAAGGGAAAGAAAGGACGAAAGGGAAAGAAAGGACGAAAGGGAAAGAAAGGACGAAAGGGAAAGAAAGGAAAACCAATCCAACCTGAAGAAACACCAGCCATTCCGACGGAGATAAAAGCTGCAGAAATAGAAAAGGAACCAAAAACAGAAGTGGTTGTGGAACCAGTTATTCCGGAAGCAGATATTGCGGAGACCGAAATACAACAAATTAAAGCAGAAGTAGAACCAGTTGAAACAAAGCCAGAAATAGTTATGGAACCAGATGTGGAACCAGTTATTCCAGAAACGGAACTTACAGAGACCGGAAAAGAAGCGAAAGTAGAACAAGTTAAACCAGTGGAAGGGAAGCTAAACTTA------------------------------------------------------------------------------------------------------------------------------------------------------------------------------------------------------------------------------------------------------------------------------------------------------------------------------------------------------------------------------------------------------------------------------------------------------------------------------GGAAAAGGAAAGGGCAAAGGAAAGAAGGTTAGAAAGGGAAAGAAAGGACGAAAGGGAAAGAAAGGACGAAAGGGAAAGAAAGGACGGAAAGGAAAGAAGGGGCGAAAAGGAAAGAAAAGAAGAAAAGGAAAAAAAGGACGAAAGGGAAAGAAAGGACGAAAGGGAAAGAAAGGACGAAAGGGCAAGAAAGGACGAAAGGGAAAGAAAGGACGAAAGGGAAAGAAAGGACGAAAGGGAAAGAAAGGACGAAGGGGAAAGAAAGGA------------------CGAAAGGGCAAGAAAGGAAATCCAATCAAAACTGAAGAAACACCAGCCATTCTGACGGAGATAAAAGCTGCAGAATTAGAAAAGGAACCAAAAACAGAAATAGTTATGAAACCAGTTATTCCCGAAACGGAACTAACAGAGACCGGAAAAGAAGCAGAAGTAGAACAAGTTAAACCAGTGGAAAGGAAGCTAAAAATAGGAAAAGGAAAGGGCAAAGGAAAGAAGGTTAGAAAGGGAAAGAAAGGACGAAAGGGAAAGAAAGGACGAAAGGGAAAGAAGGGACGGAAAGGAAAGAAGGGGCGAAAAGGAAAGAAAAGAAGAAAAGGAAAGAAAAGACGAAAAGGAAAGAAACGACGAAAGGGAAAGAAAGGACGAAAGGGAAAAAAAGGACGAAAGGGAAAGAAAGGACGAAAGGGAAAAAAAGGACGAAAGGGAAAAAAAGGACGAAAGGGAAAGAAAGGACGAAAGGGAAAGAAAGGCAAACCAATCCAAACTGAAGAAACACCAGCCATTCCGACGGAGATAAAAGCTGCAGAAATAGAAAAGGAACCAAAAACAGAAGTGGTTGTGGAACCACTTATTCAGGAAAAAGATGTTGCGGAGACCGAAATACAACCCATCGAAGCAGAAGTAGAACCAGTTGAACCAAAGACAGAAATAGTTATGGAACCAGTTATTCCGGAAGCAGATATTGCGGAGACCGAAATACAACCCATCAAAGCAGAAGAAGAACCAGTT------------------------------------------------------------------------------------------------------------GAACCAAAGCCAGAAATAGTTATGGAACCAGATGAGGAACCAGTTATTCCGGAAGCAGATATTGCGGAGACCGAAATACAACCCATCAAAGCAGAAGAAGAACCAGTTGAACCAAAGCCAGAAATAGTTATGGAACCAGATGAGGAACCAGTTATTCCGGAAGCAGATATTGCGGAGACCGAAATACAACCCATCAAAGCAGAAGAAGAACCAGTTGAACCAAAGCCAGAAATAGTTATGGAACCAGATGAGGAACCAGTTATTCCGGAAGCAGATATTGCGGAGACCGAAATACAACCCATCAGAGCAGAAGAAGAACCAGTTGAACCAAAGCCAGAAATAGTTATGGAACCAGATGAGGAACCAGTTATTCCAGAAACAGAACTAACAGAGACAGACAAACAACCAATCGAAGCAGAAGTAGAACCAGTTGAACCAAAAACAGAAGTGGTTGTGGAACCAGTTATTCCGGAAGCAGATATTGCGGAGACCGAAATACAACCCATCGAAGCAGAAGTAGAACCAGTTGAACCAAAGCCAGAAATAGTTATGGAACCAGATGTGGAACCAGTAATTCCAGAAACAGATCTTACAGAGACCGAAAAACAACCAATCGAAGCAGAAGTAGAACCAGTTGAACCGAAGACAGAGATAGTCGAACCAGAAACAGAGCCAGTCGAAGAAGCCGAGGAAGAAGCGGTTGAAGCACCCGTTATGGAACCAGTTATACCTGAGATAGAACCAAAGACAGAACCGGAAGTTGAAGCCGAGGAAAAAGAAGTCGAAACAAACGTTGAACCAGAACTACTGGAGACAGGAAAACCGATTGAAATTGGCCAAGCAACTGAAATCGAGGAGCCACCAGAAAAACAACCGGTGGAATCGGAGTCACAACCAGCGGAAGTTGAGATGGAACAAATTGAAACAAAGGAAGAACAAACTGAACCAAAGGAAGAACTAGCAGGAATTGATGAGAAATGTAAGTTTTAGTAACAATTATTTATTTTATGATTCCTTTTGAGCTGTTTCGGTATGCTTCGATGCAAGTCAAATTTGTGGATGTTTTAAATCCATCATTCAATTATGTTTGCAATGTAGTCATGAAGTTTGTTCGTTTGTTCAACACAGATATGGGACATATACGCATGAGCAGACCTTTTTTGTGCACAGATCTTCGTTCAGGTTAATCGTGCATGGCAACAAAAATTGGTTCATTCCGTTTTATGTCGTGCAGTCTTATTTTGCTTGAATTAAGCATTGCTGTAAAGTTCAAGTTAAAGGTACAGTCCATCATTTGTAATTTGTGCATTTTGTTCGTTTGAAGAAACAAACATGCTCAAAATCTAAAGAAGGCTGCTGAACAACTAACCT---------------------------------------------------------------------------------------------------------------------------------------------------------------------------------------------------------------------------------------------------------------------------------------GATGAAGTTTCAGCTCAGCTTCAGCTGTATAAATTATTTCGAGAAAAACAAAATTCTAGGATCTCCATTTCAAATAAAAAGTTGCTGAATCTTGTTTTGAATTGGGCCTGAATTCAGCGTCCGAGGCACGTTCTCATCACTGAGCCGATTGCAGCTACGCGATGTCGCGCACGGATTAAAGCAATCGAGGGCGCGTGTGCGTTGTGTCAACGCTTGCGCGCGCTTCGCCAAATAAAAAAAAAAGAACTAAACGGAAATTTCTTTAGACGCATGTCTTTAGACGCACCGTTGGAAAGGCCTACTTTCGTTGCTTTCTTCAAAATGACAGCATGCCAGAAAGAAAAATCTCTTATCTTTCAACGAAGTATGTTTTTTAACAATAGGCCTAATTACGTCAGTTACTTTTTGGATAGTTGCAAATAATGATGGACTGTAGCTTTAAGAAATTTGTGGAACTAAAGTTGTGCTTGGCAAGTTTTACTTTAGAATTCGAAAATGCATCGACACCAAAAAAAAGTGTAATTTTGGTCCTATAGTTGAAGTGTCAAACTAGAACAAATTTAATGATAAATTAAATAAATTTAAATAATTTATTCCTACCTTGCTTTGACAGTGATGAAGGAGCTGCGTGACCTTTTGGAATCTACGAAGATTGACCTTCCTGTTGACATCAATGATCCATACGACCTAGGTCTTCTTCTCAGACATTTACGTCACCATTCAAATCTTCTTGCTCGTATTGGAGACCCCGATGTCAAAAAGGAAGTCCTCAGCGCCATGAATGAAAAC

>BA31-B

GCACGACGTGCCGCANAGGAAAAACCTCAAAAGGGAAAGAAGGGACGAAAAGGAAAGAAACGACGAAAGGGAAAGAAGGGACGAAAAGGAAAGAAGGGACGAAAAGGAAAGAAACGACGAAAGGGAAAGAAAAGACGAAAGGGAAAGAAAGGACGAAAGGGAAAGAAAGGACGAAAGGGAAAGAAAGGACGAAAGGGAAAGAAAGGACGAAAGGGAAAGAAAGGACGAAAGGGAAAGAAAGGACGAAAGGGAAAGAAAGGACGAAAGGGAAAGAAAGGAAAACCAATCCAACCTGAAGAAACACCAGCCATTCCGACGGAGATAAAAGCTGCAGAAATAGAAAAGGAACCAAAAACAGAAGTGGTTGTGGAACCAGTTATTCCGGAAGCAGATATTGCGGAGACCGAAATACAACAAATTAAAGCAGAAGTAGAACCAGTTGAAACAAAGCCAGAAATAGTTATGGAACCAGATGTGGAACCAGTTATTCCAGAAACGGAACTTACAGAGACCGGAAAAGAAGCGAAAGTAGAACAAGTTAAACCAGTGGAAGGGAAGCTAAACTTA------------------------------------------------------------------------------------------------------------------------------------------------------------------------------------------------------------------------------------------------------------------------------------------------------------------------------------------------------------------------------------------------------------------------------------------------------------------------------GGAAAAGGAAAGGGCAAAGGAAAGAAGGTTAGAAAGGGAAAGAAAGGACGAAAGGGAAAGAAAGGACGAAAGGGAAAGAAAGGACGGAAAGGAAAGAAGGGGCGAAAAGGAAAGAAAAGAAGAAAAGGAAAAAAAGGACGAAAGGGAAAGAAAGGACGAAAGGGAAAGAAAGGACGAAAGGGCAAGAAAGGACGAAAGGGAAAGAAAGGACGAAAGGGAAAGAAAGGACGAAAGGGAAAGAAAGGACGAAGGGGAAAGAAAGGA------------------CGAAAGGGCAAGAAAGGAAATCCAATCAAAACTGAAGAAACACCAGCCATTCTGACGGAGATAAAAGCTGCAGAATTAGAAAAGGAACCAAAAACAGAAATAGTTATGAAACCAGTTATTCCCGAAACGGAACTAACAGAGACCGGAAAAGAAGCAGAAGTAGAACAAGTTAAACCAGTGGAAAGGAAGCTAAAAATAGGAAAAGGAAAGGGCAAAGGAAAGAAGGTTAGAAAGGGAAAGAAAGGACGAAAGGGAAAGAAAGGACGAAAGGGAAAGAAAGGACGGAAAGGAAAGAAGGGGCGAAAAGGAAAGAAAAGAAGAAAAGGAAAGAAAAGACGAAAAGGAAAGAAACGACGAAAGGGAAAGAAAGGACGAAAGGGAAAAAAAGGACGAAAGGGAAAGAAAGGACGAAAGGGAAAAAAAGGACGAAAGGGAAAAAAAGGACGAAAGGGAAAGAAAGGACGAAAGGGAAAGAAAGGCAAACCAATCCAAACTGAAGAAACACCAGCCATTCCGACGGAGATAAAAGCTGCAGAAATAGAAAAGGAACCAAAAACAGAAGTGGTTGTGGAACCACTTATTCAGGAAAAAGATGTTGCGGAGACCGAAATACAACCCATCGAAGCAGAAGTAGAACCAGTTGAACCAAAGACAGAAATAGTTATGGAACCAGTTATTCCGGAAGCAGATATTGCGGAGACCGAAATACAACCCATCAAAGCAGAAGAAGAACCAGTT------------------------------------------------------------------------------------------------------------GAACCAAAGCCAGAAATAGTTATGGAACCAGATGAGGAACCAGTTATTCCGGAAGCAGATATTGCGGAGACCGAAATACAACCCATCAAAGCAGAAGAAGAACCAGTTGAACCAAAGCCAGAAATAGTTATGGAACCAGATGAGGAACCAGTTATTCCGGAAGCAGATATTGCGGAGACCGAAATACAACCCATCAAAGCAGAAGAAGAACCAGTTGAACCAAAGCCAGAAATAGTTATGGAACCAGATGAGGAACCAGTTATTCCGGAAGCAGATATTGCGGAGACCGAAATACAACCCATCAGAGCAGAAGAAGAACCAGTTGAACCAAAGCCAGAAATAGTTATGGAACCAGATGAGGAACCAGTCCTTCCAGAAACAGAACTAACAGAGACAGACAAACAACCAATCGAAGCAGAAGTAGAACCAGTTGAACCAAAAACAGAAGTGGTTGTGGAACCAGTTATTCCGGAAGCAGATATTGCGGAGACCGAAATACAACCCATCGAAGCAGAAGTAGAACCAGTTGAACCAAAGCCAGAAATAGTTATGGAACCAGATGTGGAACCAGTAATTCCAGAAACAGATCTTACAGAGACCGAAAAACAACCAATCGAAGCAGAAGTAGAACCAGTTGAACCGAAGACAGAGATAGTCGAACCAGAAACAGAGCCAGTCGAAGAAGCCGAGGAAGAAGCGGTTGAAGCACCCGTTATGGAACCAGTTATACCTGAGATAGAACCAAAGACAGAACCGGAAGTTGAAGCCGAGGAAAAAGAAGTCGAAACAAACGTTGAACCAGAACTACTGGAGACAGGAAAACCGATTGAAATTGGCCAAGCAACTGAAATCGAGGAGCCACCAGAAAAACAACCGGTGGAATCGGAGTCACAACCAGCGGAAGTTGAGATGGAACAAATTGAAACAAAGGAAGAACAAACTGAACCAAAGGAAGAACTAGCAGGAATTGATGAGAAATGTAAGTTTTAGTAACAATTATTTATTTTATGATTCCTTTTGAGCTGTTTCGGTATGCTTCGATGCAAGTCAAATTTGTGGATGTTTTAAATCCATCATTCAATTATGTTTGCAATGTAGTCATGAAGTTTGTTCGTTTGTTCAACACAGATATGGGACATATACGCATGAGCAGACCTTTTTTGTGCACAGATCTTCGTTCAGGTTAATCGTGCATGGTAACAAAAATTGGTTCATTCCGTTTTATGTCGTGCAGTCTTATTTTGCTTGAATTAAGCATTGCTGTAAAGTTCAAGTTAAAGGTACAGTCCATCATTTGTAATTTGTGCATTTTGTTCGTTTGAAGAAACAAACATGCTCAAAATCTAAAGAAGGCTGCTGAACAACTAACCT---------------------------------------------------------------------------------------------------------------------------------------------------------------------------------------------------------------------------------------------------------------------------------------GATGAAGTTTCAGCTCAGCTTCAGCTGTATAAATTATTTCGAGAAAAACAAAATTCTAGGATCTCCATTTCAAATCAAAAGTTGCTGAATCTTGTTTTGAATTGGGCCTGAATTCAGCGTCCGAGGCACGTTCTCATCACTGAGCCGATTGCAGCTACGCGATGTCGCGCACGAATTAAAGCAATCGAGGGCGCGTGTGCGTTGTGTCAACGCTTGCACGCGCTTCGCCAAATAAAAAAAAAAGAACTAAACGGAAATTTCTTTAGACGCATGTCTTTAGACGCACCGTTGGAAAGGCCTACTTTCGTTGCTTTCTTCAAAATGACAGCATGCCAGAAAGAAAAATCTCTTATCTTTCAACGAAGTATGTTTTTTAACAATAGGCCTAATTACGTCAGTTACTTTTTGGATAGTTGCAAATAATGATGGACTGTAGCTTTAAGAAATTTGTGGAACTCAAGTTGTGCTTGGCAAGTTTTACTTTAGAATTCGAAAATGCATCGACACCAAAAAAAAGTGTAATTTTGGTCCTATAGTTGAAGTGTCAAACTAGAACAAATTTAATGATAAATTAAATAAATTTAAATAATTTATTCCTACCTTGCTTTGACAGTGATGAAGGAGCTGCGTGACCTTTTGGAATCTACGAAGATTGACCTTCCTGTTGACATCAATGATCCATACGACCTAGGTCTTCTTCTCAGACATTTACGTCACCATTCAAATCTTCTTGCTCGTATTGGAGACCCCGATGTCAAAAAGGAAGTCCTCAGCGCCATGAATGAAAAC

>BA55-A

GCACGACGTGCCGCAGAGGAAAAACCTCAAAAGGGAAAGAAGGGACGAAAAGGAAAGAAACGACGAAAGGGAAAGAAGGGACGAAAAGGAAAGAAGGGACGAAAAGGAAAGAAACGACGAAAGGGAAAGAAAAGACGAAAGGGAAAGAAAGGACGAAAGGGAAAGAAAGGACGAAAGGGAAAGAAAGGACGAAAGGGAAAGAAAGGACGAAAGGGAAAGAAAGGACGAAAGGGAAAGAAAGGACGAAAGGGAAAGAAAGGACGAAAGGGAAAGAAAGGAAAACCAATCCAACCTGAAGAAACACCAGCCATTCCGACGGAGATAAAAGCTGCAGAAATAGAAAAGGAACCAAAAACAGAAGTGGTTGTGGAACCAGTTATTCCGGAAGCAGATATTGCGGAGACCGAAATACAACAAATTAAAGCAGAAGTAGAACCAGTTGAAACAAAGCCAGAAATAGTTATGGAACCAGATGTGGAACCAGTTATTCCAGAAACGGAACTTACAGAGACCGGAAAAGAAGCGAAAGTAGAACAAGTTAAACCAGTGGAAGGGAAGCTAAACTTA------------------------------------------------------------------------------------------------------------------------------------------------------------------------------------------------------------------------------------------------------------------------------------------------------------------------------------------------------------------------------------------------------------------------------------------------------------------------------GGAAAAGGAAAGGGCAAAGGAAAGAAGGTTAGAAAGGGAAAGAAAGGACGAAAGGGAAAGAAAGGACGAAAGGGAAAGAAAGGACGGAAAGGAAAGAAGGGGCGAAAAGGAAAGAAAAGAAGAAAAGGAAAAAAAGGACGAAAGGGAAAGAAAGGACGAAAGGGAAAGAAAGGACGAAAGGGCAAGAAAGGACGAAAGGGAAAGAAAGGACGAAAGGGAAAGAAAGGACGAAAGGGAAAGAAAGGACGAAGGGGAAAGAAAGGA------------------CGAAAGGGCAAGAAAGGAAATCCAATCAAAACTGAAGAAACACCAGCCATTCTGACGGAGATAAAAGCTGCAGAATTAGAAAAGGAACCAAAAACAGAAATAGTTATGAAACCAGTTATTCCCGAAACGGAACTAACAGAGACCGGAAAAGAAGCAGAAGTAGAACAAGTTAAACCAGTGGAAAGGAAGCTAAAAATAGGAAAAGGAAAGGGCAAAGGAAAGAAGGTTAGAAAGGGAAAGAAAGGACGAAAGGGAAAGAAAGGACGAAAGGGAAAGAAAGGACGGAAAGGAAAGAAGGGGCGAAAAGGAAAGAAAAGAAGAAAAGGAAAGAAAAGACGAAAAGGAAAGAAACGACGAAAGGGAAAGAAAGGACGAAAGGGAAAAAAAGGACGAAAGGGAAAGAAAGGACGAAAGGGAAAAAAAGGACGAAAGGGAAAAAAAGGACGAAAGGGAAAGAAAGGACGAAAGGGAAAGAAAGGCAAACCAATCCAAACTGAAGAAACACCAGCCATTCCGACGGAGATAAAAGCTGCAGAAATAGAAAAGGAACCAAAAACAGAAGTGGTTGTGGAACCACTTATTCAGGAAAAAGATGTTGCGGAGACCGAAATACAACCCATCGAAGCAGAAGTAGAACCAGTTGAACCAAAGACAGAAATAGTTATGGAACCAGTTATTCCGGAAGCAGATATTGCGGAGACCGAAATACAACCCATCAAAGCAGAAGAAGAACCAGTT------------------------------------------------------------------------------------------------------------GAACCAAAGCCAGAAATAGTTATGGAACCAGATGAGGAACCAGTTATTCCGGAAGCAGATATTGCGGAGACCGAAATACAACCCATCAAAGCAGAAGAAGAACCAGTTGAACCAAAGCCAGAAATAGTTATGGAACCAGATGAGGAACCAGTTATTCCGGAAGCAGATATTGCGGAGACCGAAATACAACCCATCAAAGCAGAAGAAGAACCAGTTGAACCAAAGCCAGAAATAGTTATGGAACCAGATGAGGAACCAGTTATTCCGGAAGCAGATATTGCGGAGACCGAAATACAACCCATCAGAGCAGAAGAAGAACCAGTTGAACCAAAGCCAGAAATAGTTATGGAACCAGATGAGGAACCAGTCCTTCCAGAAACAGAACTAACAGAGACAGACAAACAACCAATCGAAGCAGAAGTAGAACCAGTTGAACCAAAAACAGAAGTGGTTGTGGAACCAGTTATTCCGGAAGCAGATATTGCGGAGACCGAAATACAACCCATCGAAGCAGAAGTAGAACCAGTTGAACCAAAGCCAGAAATAGTTATGGAACCAGATGTGGAACCAGTAATTCCAGAAACAGATCTTACAGAGACCGAAAAACAACCAATCGAAGCAGAAGTAGAACCAGTTGAACCGAAGACAGAGATAGTCGAACCAGAAACAGAGCCAGTCGAAGAAGCCGAGGAAGAAGCGGTTGAAGCACCCGTTATGGAACCAGTTATACCTGAGATAGAACCAAAGACAGAACCGGAAGTTGAAGCCGAGGAAAAAGAAGTCGAAACAAACGTTGAACCAGAACTACTGGAGACAGGAAAACCGATTGAAATTGGCCAAGCAACTGAAATCGAGGAGCCACCAGAAAAACAACCGGTGGAATCGGAGTCACAACCAGCGGAAGTTGAGATGGAACAAATTGAAACAAAGGAAGAACAAACTGAACCAAAGGAAGAACTAGCAGGAATTGATGAGAAATGTAAGTTTTAGTAACAATTATTTATTTTATGATTCCTTTTGAGCTGTTTCGGTATGCTTCGATGCAAGTCAAATTTGTGGATGTTTTAAATCCATCATTCAATTATGTTTGCAATGTAGTCATGAAGTTTGTTCGTTTGTTCAACACAGATATGGGACATATACNCATGAGCAGACCTTTTTTGTGCACAGATCTTCGTTCAGGTTAATCGTGCATGGCAACAAAAATTGGTTCATTCCGTTTTATGTCGTGCAGTCTTATTTTGCTTGAATTAAGCATTGCTGTAAAGTTCAAGTTAAAGGTACAGTCCATCATTTGTAATTTGTGCATTTTGTTCGTTTGAAGAAACAAACATGCTCAAAATCTAAAGAAGGCTGCTGAACAACTAACCT---------------------------------------------------------------------------------------------------------------------------------------------------------------------------------------------------------------------------------------------------------------------------------------GATGAAGTTTCAGCCCAGCTTCAGCTGTATAAATTATTTCGAGAAAAACAAAATTCTAGGATCTCCATTTCAAATCAAAAGTTGCTGAATCTTGTTTTGAATTGGGCCTGAATTCAGCGTCCGAGGCACGTTCTCATCACTGAGCCGATTGCAGCTACACGATGTCGCGCACGAATTAAAGCAATCGAGGGCNCGTGTGCGTTGTGTCAACGCTTGCGCGCGCTTCGCCAAATAAAAAAAAAAGAACTAAACGGAAATTTCTTTAGACGCATGTCTTTAGACGCACCGTTGGAAAGGCCTACTTTCGTTGCTTTCTTCAAAATGACAGCATGCCAGAAAGAAAAATCTCTTATCTTTCAACGAAGTATGTTTTTTAACAATAGGCCTAATTACGTCAGTTACTTTTTGGATAGTTGCAAATAATGATGGACTGTAGCTTTAAGAAATTTGTGGAACTCAAGTTGTGCTTGGCAAGTTTTACTTTAGAATTCGAAAATGCATCGACACCAAAAAAAAGTGTAATTTTGGTCCTATAGTTGAAGTGTCAAACTAGAACAAATTTAATGATAAATTAAATAAATTTAAATAATTTATTCCTACCTTGCTTTGACAGTGATGAAGGAGCTGCGTGACCTTTTGGAATCTACGAAGATTGACCTTCCTGTTGACATCAATGATCCATACGACCTAGGTCTTCTTCTCAGACATTTACGTCACCATTCAAATCTTCTTGCTCGTATTGGAGACCCCGATGTCAAAAAGGAAGTCCTCAGCGCCATGAATGAAAAC

>BA96-A

GCACGACGTGCCGCAGAGGAAAAACCTCAAAAGGGAAAGAAGGGACGAAAAGGAAAGAAACGACGAAAGGGAAAGAAGGGACGAAAAGGAAAGAAGGGACGAAAAGGAAAGAAACGACGAAAGGGAAAGAAAAGACGAAAGGGAAAGAAAGGACGAAAGGGAAAGAAAGGACGAAAGGGAAAGAAAGGACGAAAGGGAAAGAAAGGACGAAAGGGAAAGAAAGGACGAAAGGGAAAGAAAGGACGAAAGGGAAAGAAAGGACGAAAGGGAAAGAAAGGAAAACCAATCCAACCTGAAGAAACACCAGCCATTCCGACGGAGATAAAAGCTGCAGAAATAGAAAAGGAACCAAAAACAGAAGTGGTTGTGGAACCAGTTATTCCGGAAGCAGATATTGCGGAGACCGAAATACAACAAATTAAAGCAGAAGTAGAACCAGTTGAAACAAAGCCAGAAATAGTTATGGAACCAGATGTGGAACCAGTTATTCCAGAAACGGAACTTACAGAGACCGGAAAAGAAGCGAAAGTAGAACAAGTTAAACCAGTGGAAGGGAAGCTAAACTTA------------------------------------------------------------------------------------------------------------------------------------------------------------------------------------------------------------------------------------------------------------------------------------------------------------------------------------------------------------------------------------------------------------------------------------------------------------------------------GGAAAAGGAAAGGGCAAAGGAAAGAAGGTTAGAAAGGGAAAGAAAGGACGAAAGGGAAAGAAAGGACGAAAGGGAAAGAAAGGACGGAAAGGAAAGAAGGGGCGAAAAGGAAAGAAAAGAAGAAAAGGAAAAAAAGGACGAAAGGGAAAGAAAGGACGAAAGGGAAAGAAAGGACGAAAGGGCAAGAAAGGACGAAAGGGAAAGAAAGGACGAAAGGGAAAGAAAGGACGAAAGGGAAAGAAAGGACGAAGGGGAAAGAAAGGA------------------CGAAAGGGCAAGAAAGGAAATCCAATCAAAACTGAAGAAACACCAGCCATTCTGACGGAGATAAAAGCTGCAGAATTAGAAAAGGAACCAAAAACAGAAATAGTTATGAAACCAGTTATTCCCGAAACGGAACTAACAGAGACCGGAAAAGAAGCAGAAGTAGAACAAGTTAAACCAGTGGAAAGGAAGCTAAAAATAGGAAAAGGAAAGGGCAAAGGAAAGAAGGTTAGAAAGGGAAAGAAAGGACGAAAGGGAAAGAAAGGACGAAAGGGAAAGAAAGGACGGAAAGGAAAGAAGGGGCGAAAAGGAAAGAAAAGAAGAAAAGGAAAGAAAAGACGAAAAGGAAAGAAACGACGAAAGGGAAAGAAAGGACGAAAGGGAAAAAAAGGACGAAAGGGAAAGAAAGGACGAAAGGGAAAAAAAGGACGAAAGGGAAAAAAAGGACGAAAGGGAAAGAAAGGACGAAAGGGAAAGAAAGGAAAACCAATCCAAACTGAAGAAACACCAGCCATTCCGACGGATATAAAAGCTGCAGAAATAGAAAAGGAACCAAAAACAGAAGTGGTTGTGGAACCACTTATTCAGGAAAAAGATGTTGCGGAGACCGAAATACAACCCATCGAAGCAGAAGTAGAACCAGTTGAACCAAAGACAGAAATAGTTATGGAACCAGTTATTCCGGAAGCAGATATTGCGGAGACCGAAATACAACCCATCAGAGCAGAAGAAGAACCAGTT------------------------------------------------------------------------------------------------------------------------------------------------------------------------------------------------------------------------------------------------------------------------------------------------------------------------------------------------------------------------------------------------------------------------------------------------GAACCAAAGCCAGAAATAGTTATGGAACCAGATGAGGAACCAGTCATTCCAGAAACAGAACTAACAGAGACAGACAAACAACCAATCGAAGCAGAGGTAGAACCAGTTGAACCAAAAACAGAAGTGGTTGTGGAACCAGTTATTCCGGAAGCAGATATTGCGGAGACCGAAATACAACCCATCGAAGCAGAAGTAGAACCAGTTGAACCAAAGCCAGAAATAGTTATGGAACCAGATGTGGAACCAGTAATTCCAGAAACAGATCTTACAGAGACCGAAAAACAACCAATCGAAGCAGAAGTAGAACCAGTTGAACCGAAGACAGAGATAGTCGAACCAGAAACAGAGCCAGTCGAAGAAGCCGAGGAAGAAGCGGTTGAAGCACCCGTTACGGAACCAGTTATACCTGAGATAGAACCAAAGACAGAACCGGAAGTTGAAGCCGAGGAAAAAGAAGTCGAAACAAACGTTGAACCAGAACTACTGGAGACAGGAAAACCGATTGAAATTGGCCAAGCAACTGAAATCGAGGAGCCACCAGAAAAACAACCGGTGGAATCGGAGTCACAACCAGCGGAAGTTGAGATGGAACAAATTGAAACAAAGGAAGAACCAACTGAACCAAAGGAAGAACTAGCAGGAATTGATGAGAAATGTAAGTTTTAGTAACAATTATTTATTTTATGATTCCTTTTGAGCTGTTTCGGTATGCTTCGATGCAAGTCAAATTTGTAGATGTTTTAAATCCATCATTCAATT--GTTTGCAATGTAGTCATGAAGTTTGTTCGTTTGTTCAACACAGATATGGGACATATACGCATGAGCAGACCTTTTTTGTGCACAGATCTTCGTTCAGGTTAATCGTGCATGGCAACAAAAATTGGTTCATTCCGTTTTATGTCGTGCAGTCTTATTTTGCTTGAATTAAGCATTGCTGTAAAGTTCAAGTTAAAGGTACAGTCCATCATTTGTAATTTGTGCATTTTGTTCGTTTGAAGAAACAAACATGCTCAAAATCTAAAGAAGGCTGCTGAACAACTAACCT---------------------------------------------------------------------------------------------------------------------------------------------------------------------------------------------------------------------------------------------------------------------------------------GATGAAGTTTCAGCTCAGCTTCAGCTGTATAAATTATTTCGAGAAAAACAAAATTCTAGGATCTCCATTTCAAATAAAAAGTTGCTGAATCTTGTTTTGAATTGGGCCTGAATTCAGCGTCCGAGGCACGTTCTCATCACTGAGCCGATTGCAGCTACGCGATGTCGCGCACGAATTAAAGCAATCGAGGGCGCGTGCGCGTTGTGTCAACGCTTGCGCGCGCTTCGCCAAATAAAAAAAAAAGAACTAAACGGAAATTTCTTTAGACGCATGTCTTTAGACGCACCGTTGGAAAGGCCTACTTTCGTTGCTTTCTTCAAAATGACAGCATGCCAGAAAGAAAAATCTCTTATCTTTCAACGAAGTATGTTTTTTAACAATAGGCCTAATTACGTCAGTTACTTTTTGGATAGTTGCAAATAATGATGGACTGTAGCTTTAAGAAATTTGTGGAACTAAAGTTGTGCTTGACAAGTTTTACTTTAGAATTCGAAAATGCATCGACACCAAAAAAAAGTGTAATTTTGGTCCTATAGTTGAAGTGTCAAACTAGAACAAATTTAATGATAAATTAAATAAATTTAAATAATTTATTCCTACCCTGCTTTGACAGTGATGAAGGAGCTGCGTGACCTTTTGGAATCTACGAAGATTGACCTTCCTGTTGACATCAATGATCCATACGACCTAGGTCTTCTTCTCAGACATTTACGTCACCATTCAAATCTTCTTGCTCGTATTGGAGACCCCGATGTCAAAAAGGAAGTCCTCAGCGCCATGAATGAAAAC

>BA71-B

GCACGACGTGCCGCAGAGGAAAAACCTCAAAAGGGAAAGAAGGGACGAAAAGGAAAGAAACGACGAAAGGGAAAGAAGGGACGAAAAGGAAAGAAGGGACGAAAAGGAAAGAAACGACGAAAGGGAAAGAAAAGACGAAAGGGAAAGAAAGGACGAAAGGGAAAGAAAGGACGAAAGGGAAAGAAAGGACGAAAGGGAAAGAAAGGACGAAAGGGAAAGAAAGGACGAAAGGGAAAGAAAGGACGAAAGGGAAAGAAAGGACGAAAGGGAAAGAAAGGAAAACCAATCCAACCTGAAGAAACACCAGCCATTCCGACGGAGATAAAAGCTGCAGAAATAGAAAAGGAACCAAAAACAGAAGTGGTTGTGGAACCAGTTATTCCGGAAGCAGATATTGCGGAGACCGAAATACAACAAATTAAAGCAGAAGTAGAACCAGTTGAAACAAAGCCAGAAATAGTTATGGAACCAGATGTGGAACCAGTTATTCCAGAAACGGAACTTACAGAGACCGGAAAAGAAGCGAAAGTAGAACAAGTTAAACCAGTGGAAGGGAAGCTAAACTTA------------------------------------------------------------------------------------------------------------------------------------------------------------------------------------------------------------------------------------------------------------------------------------------------------------------------------------------------------------------------------------------------------------------------------------------------------------------------------GGAAAAGGAAAGGGCAAAGGAAAGAAGGTTAGAAAGGGAAAGAAAGGACGAAAGGGAAAGAAAGGACGAAAGGGAAAGAAAGGACGGAAAGGAAAGAAGGGGCGAAAAGGAAAGAAAAGAAGAAAAGGAAAAAAAGGACGAAAGGGAAAGAAAGGACGAAAGGGAAAGAAAGGACGAAAGGGCAAGAAAGGACGAAAGGGAAAGAAAGGACGAAAGGGAAAGAAAGGACGAAAGGGAAAGAAAGGACGAAGGGGAAAGAAAGGA------------------CGAAAGGGCAAGAAAGGAAATCCAATCAAAACTGAAGAAACACCAGCCATTCTGACGGAGATAAAAGCTGCAGAATTAGAAAAGGAACCAAAAACAGAAATAGTTATGAAACCAGTTATTCCCGAAACGGAACTAACAGAGACCGGAAAAGAAGCAGAAGTAGAACAAGTTAAACCAGTGGAAAGGAAGCTAAAAATAGGAAAAGGAAAGGGCAAAGGAAAGAAGGTTAGAAAGGGAAAGAAAGGACGAAAGGGAAAGAAAGGACGAAAGGGAAAGAAAGGACGGAAAGGAAAGAAGGGGCGAAAAGGAAAGAAAAGAAGAAAAGGAAAGAAAAGACGAAAAGGAAAGAAACGACGAAAGGGAAAGAAAGGACGAAAGGGAAAAAAAGGACGAAAGGGAAAGAAAGGACGAAAGGGAAAAAAAGGACGAAAGGGAAAAAAAGGACGAAAGGGAAAGAAAGGACGAAAGGGAAAGAAAGGCAAACCAATCCAAACTGAAGAAACACCAGCCATTCCNACGGAGATAAAAGCTGCAGAAATAGAAAAGGAACCAAAAACAGAAGTGGTTGTGGAACCACTTATTCAGGAAAAAGATGTTGCGGAGACCGAAATACAACCCATCGAAGCAGAAGTAGAACCAGTTGAACCAAAGACAGAAATAGTTATGGAACCNGTTATTCCGGAAGCAGATATTGCGGAGACCGAAATACAACCCATCAAAGCAGAAGAAGAACCAGTT------------------------------------------------------------------------------------------------------------GAACCAAAGCCAGAAATAGTTATGGAACCAGATGAGGAACCAGTTATTCCGGAAGCAGATATTGCGGAGACCGAAATACAACCCATCAAAGCAGAAGAAGAACCAGTTGAACCAAAGCCAGAAATAGTTATGGAACCAGATGAGGAACCAGTTATTCCGGAAGCAGATATTGCGGAGACCGAAATACAACCCATCAGAGCAGAAGAAGAACCAGTTGAACCAAAGCCAGAAATAGTTATGGAACCAGATGAGGAACCAGTTATTCCGGAAGCAGATATTGCGGAGACCGAAATACAACCCATCAGAGCAGAAGAAGAACCAGTTGAACCAAAGCCAGAAATAGTTATGGAACCAGATGAGGAACCAGTCATTCCAGAAACAGAACTAACAGAGACAGACAAACAACCAATCGAAGCAGAAGTAGAACCAGTTGAACCAAAAACAGAAGTGGTTGTGGAACCAGTTATTCCGGAAGCAGATATTGCGGAGACCGAAATACAACCCATCGAAGCAGAAGTAGAACCAGTTGAACCAAAGCCAGAAATAGTTATGGAACCAGATGTGGAACCAGTAATTCCAGAAACAGATCTTACAGAGACCGAAAAACAACCAATCGAAGCAGAAGTAGAACCAGTTGAACCGAAGACAGAGATAGTCGAACCAGAAACAGAGCCAGTCGAAGAAGCCGAGGAAGAAGCGGTTGAAGCACCCGTTATGGAACCAGTTATACCTGAGATAGAACCAAAGACAGAACCGGAAGTTGAAGCCGAGGAAAAAGAAGTCGAAACAAACGTTGAACCAGAACTACTGGAGACAGGAAAACCGATTGAAATTGGCCAAGCAACTGAAATCGAGGAGCCACCAGAAAAACAACCGGTGGAATCGGAGTCACAACCAGCGGAAGTTGAGATGGAACAAATTGAAACAAAGGAAGAACAAACTGAACCAAAGGAAGAACTAGCAGGAATTGATGAGAAATGTAAGTTTTAGTAACAATTATTTATTTTATGATTCCTTTTGAGCTGTTTCGGTATGCTTCGATGCAAGTCAAATTTGTGGATGTTTTAAATCCATCATTCAATTATGTTTGCAATGTAGTCATGAAGTTTGTTCGTTTGTTCAACACAGATATGGGACATATACGCATGAGCAGACCTTTTTTGTGCACAGATCTTCGTTCAGGTTAATCGTGCATGGCAACAAAAATTGGTTCATTCCGTTTTATGTCGTGCAGTCTTATTTTGCTTGAATTAAGCATTGCTGTAAAGTTCAAGTTAAAGGTACAGTCCATCATTTGTAATTTGTGCATTTTGTTCGTTTGAAGAAACAAACATGCTCAAAATCTAAAGAAGGCTGCTGAACAACTAACCT---------------------------------------------------------------------------------------------------------------------------------------------------------------------------------------------------------------------------------------------------------------------------------------GATGAAGTTTCAGCTCAGCTTCAGCTGTATAAATTATTTCGAGAAAAACAAAATTCTAGGATCTCCATTTCAAATCAAAAGTTGCTGAATCTTGTTTTGAATTGGGCCTGAATTCAGCGTCCGAGGCACGTTCTCATCACTGAGCCGATTGCAGCTACGCGATGTCGCGCACGAATTAAAGCAATCGAGGGCGCGTGTGCGTTGTGTCAACGCTTGCGCGCGCTTCGCCAAATAAAAAAAAAAGAACTAAACGGAAATTTCTTTAGACGCATGTCTTTAGACGCACCGTTGGAAAGGCCTACTTTCGTTGCTTTCTTCAAAATGACAGCATGCCAGAAAGAAAAATCTCTTATCTTTCAACGAAGTATGTTTTTTAACAATAGGCCTAATTACGTCAGTTACTTTTTGGATAGTTGCAAATAATGATGGACTGTAGCTTTAAGAAATTTGTGGAACTCAAGTTGTGCTTGGCAAGTTTTACTTTAGAATTCGAAAATGCATCGACACCAAAAAAAAGTGTAATTTTGGTCCTATAGTTGAAGTGTCAAACTAGAACAAATTTAATGATAAATTAAATAAATTTAAATAATTTATTCCTACCTTGCTTTGACAGTGATGAAGGAGCTGCGTGACCTTTTGGAATCTACGAAGATTGACCTTCCTGTTGACATCAATGATCCATACGACCTAGGTCTTCTTCTCAGACATTTACGTCACCATTCAAATCTTCTTGCTCGTATTGGAGACCCCGATGTCAAAAAGGAAGTCCTCAGCGCCATGAATGAAAAC

>CA02-A

GCACGACGTGCCGCAGAGGAAAAACCTCAAAAGGGAAAGAAGGGACGAAAAGGAAAGAAACGACGAAAGGGAAAGAAGGGACGAAAAGGAAAGAAGGGACGAAAAGGAAAGAAACGACGAAAGGGAAAGAAAAGACGAAAGGGAAAGAAAGGACGAAAGGGAAAGAAAGGACGAAAGGGAAAGAAAGGACGAAAGGGAAAGAAAGGACGAAAGGGAAAGAAAGGACGAAAGGGAAAGAAAGGACGAAAGGGAAAGAAAGGACGAAAGGGAAAGAAAGGAAAACCAATCCAACCTGAAGAAACACCAGCCATTCCGACGGAGATAAAAGCTGCAGAAATAGAAAAGGAACCAAAAACAGAAGTGGTTGTGGAACCAGTTATTCCGGAAGCAGATATTGCGGAGACCGAAATACAACAAATTAAAGCAGAAGTAGAACCAGTTGAAACAAAGCCAGAAATAGTTATGGAACCAGATGTGGAACCAGTTATTCCAGAAACGGAACTTACAGAGACCGGAAAAGAAGCGAAAGTAGAACAAGTTAAACCAGTGGAAGGGAAGCTAAACTTA------------------------------------------------------------------------------------------------------------------------------------------------------------------------------------------------------------------------------------------------------------------------------------------------------------------------------------------------------------------------------------------------------------------------------------------------------------------------------GGAAAAGGAAAGGGCAAAGGAAAGAAGGTTAGAAAGGGAAAGAAAGGACGAAAGGGAAAGAAAGGACGAAAGGGAAAGAAAGGACGGAAAGGAAAGAAGGGGCGAAAAGGAAAGAAAAGAAGAAAAGGAAAAAAAGGACGAAAGGGAAAGAAAGGACGAAAGGGAAAGAAAGGACGAAAGGGCAAGAAAGGACGAAAGGGAAAGAAAGGACGAAAGGGAAAGAAAGGACGAAAGGGAAAGAAAGGACGAAGGGGAAAGAAAGGA------------------CGAAAGGGCAAGAAAGGAAATCCAATCAAAACTGAAGAAACACCAGCCATTCTGACGGAGATAAAAGCTGCAGAATTAGAAAAGGAACCAAAAACAGAAATAGTTATGAAACCAGTTATTCCCGAAACGGAACTAACAGAGACCGGAAAAGAAGCAGAAGTAGAACAAGTTAAACCAGTGGAAAGGAAGCTAAAAATAGGAAAAGGAAAGGGCAAAGGAAAGAAGGTTAGAAAGGGAAAGAAAGGACGAAAGGGAAAGAAAGGACGAAAGGGAAAGAAAGGACGGAAAGGAAAGAAGGGGCGAAAAGGAAAGAAAAGAAGAAAAGGAAAGAAAAGACGAAAAGGAAAGAAACGACGAAAGGGAAAGAAAGGACGAAAGGGAAAAAAAGGACGAAAGGGAAAGAAAGGACGAAAGGGAAAAAAAGGACGAAAGGGAAAAAAAGGACGAAAGGGAAAGAAAGGACGAAAGGGAAAGAAAGGCAAACCAATCCAAACTGAAGAAACACCAGCCATTCCGACGGAGATAAAAGCTGCAGAAATAGAAAAGGAACCAAAAACAGAAGTGGTTGTGGAACCACTTATTCAGGAAAAAGATGTTGCGGAGACCGAAATACAACCCATCGAAGCAGAAGTAGAACCAGTTGAACCAAAGACAGAAATAGTTATGGAACCAGTTATTCCGGAAGCAGATATTGCGGAGACCGAAATACAACCCATCAAAGCAGAAGAAGAACCAGTT------------------------------------------------------------------------------------------------------------GAACCAAAGCCAGAAATAGTTATGGAACCAGATGAGGAACCAGTTATTCCGGAAGCAGATATTGCGGAGACCGAAATACAACCCATCAAAGCAGAAGAAGAACCAGTTGAACCAAAGCCAGAAATAGTTATGGAACCAGATGAGGAACCAGTTATTCCGGAAGCAGATATTGCGGAGACCGAAATACAACCCATCAAAGCAGAAGAAGAACCAGTTGAACCAAAGCCAGAAATAGTTATGGAACCAGATGAGGAACCAGTTATTCCGGAAGCAGATATTGCGGAGACCGAAATACAACCCATCAGAGCAGAAGAAGAACCAGTTGAACCAAAGCCAGAAATAGTTATGGAACCAGATGAGGAACCAGTCATTCCAGAAACAGAACTAACAGAGACAGACAAACAACCAATCGAAGCAGAAGTAGAACCAGTTGAACCAAAAACAGAAGTGGTTGTGGAACCAGTTATTCCGGAAGCAGATATTGCGGAGACCGAAATACAACCCATCGAAGCAGAAGTAGAACCAGTTGAACCAAAGCCAGAAATAGTTATGGAACCAGATGTGGAACCAGTAATTCCAGAAACAGATCTTACAGAGACCGAAAAACAACCAATCGAAGCAGAAGTAGAACCAGTTGAACCGAAGACAGAGATAGTCGAACCAGAAACAGAGCCAGTCGAAGAAGCCGAGGAAGAAGCGGTTGAAGCACCCGTTATGGAACCAGTTATACCTGAGATAGAACCAAAGACAGAACCGGAAGTTGAAGCCGAGGAAAAAGAAGTCGAAACAAACGTTGAACCAGAACTACTGGAGACAGGAAAACCGATTGAAATTGGCCAAGCAACTGAAATCGAGGAGCCACCAGAAAAACAACCGGTGGAATCGGAGTCACAACCAGCGGAAGTTGAGATGGAACAAATTGAAACAAAGGAAGAACAAACTGAACCAAAGGAAGAACTAGCAGGAATTGATGAGAAATGTAAGTTTTAGTAACAATTATTTATTTTATGATTCCTTTTGAGCTGTTTCGGTATGCTTCGATGCAAGTCAAATTTGTGGATGTTTTAAATCCATCATTCAATTATGTTTGCAATGTAGTCATGAAGTTTGTTCGTTTGTTCAACACAGATATGGGACATATACGCATGAGCAGACCTTTTTTGTGCACAGATCTTCGTTCAGGTTAATCGTGCATGGCAACAAAAATTGGTTCATTCTGTTTTATGTCGTGCAGTCTTATTTTGCTTGAATTAAGCATTGCTGTAAAGTTCAAGTTAAAGGTACAGTCCATCATTTGTAATTTGTGCATTTTGTTCGTTTGAAGAAACAAACATGCTCAAAATCTAAAGAAGGCTGCTGAACAACTAACCT---------------------------------------------------------------------------------------------------------------------------------------------------------------------------------------------------------------------------------------------------------------------------------------GATGAAGTTTCAGCTCAGCTTCAGCTGTATAAATTATTTCGAGAAAAACAAAATTCTAGGATCTCCATTTCAAATCAAAAGTTGCTGAATCTTGTTTTGAATTGGGCCTGAATTCAGCGTCCGAGGCACGTTCTCATCACTGAGCCGATTGCAGCTACGCGATGTCGCGCACGAATTAAAGCAATCGAGGGCGCGTGTGCGTTGTGTCAACGCTTGCGCGCGCTTCGCCAAAT-AAAAAAAAAGAACTAAACGGAAATTTCTTTAGACGCATGTCTTTAGACGCACCGTTGGAAAGGCCTACTTTCGTTGCTTTCTTCAAAATGACAGCATGCCAGAAAGAAAAATCTCTTATCTTTCAACGAAGTATGTTTTTTAACAATAGGCCTAATTACGTCAGTTACTTTTTGGATAGTTGCAAATAATGATGGACTGTAGCTTTAAGAAATTTGTGGAACTCAAGTTGTGCTTGGCAAGTTTTACTTTAGAATTCGAAAATGCATCGACACCAAAAAAAAGTGTAATTTTGGTCCTATAGTTGAAGTGTCAAACTAGAACAAATTTAATGATAAATTAAATAAATTTAAATAATTTATTCCTACCTTGCTTTGACAGTGATGAAGGAGCTGCGTGACCTTTTGGAATCTACGAAGATTGACCTTCCTGTTGACATCAATGATCCATACGACCTAGGTCTTCTTCTCAGACATTTACGTCACCATTCAAATCTTCTTGCTCGTATTGGAGACCCCGATGTCAAAAAGGAAGTCCTCAGCGCCATGAATGAAAAC

>CA04-A

GCACGACGTGCCGCAGAGGAAAAACCTCAAAAGGGAAAGAAGGGACGAAAAGGAAAGAAACGACGAAAGGGAAAGAAGGGACGAAAAGGAAAGAAGGGACGAAAAGGAAAGAAACGACGAAAGGGAAAGAAAAGACGAAAGGGAAAGAAAGGACGAAAGGGAAAGAAAGGACGAAAGGGAAAGAAAGGACGAAAGGGAAAGAAAGGACGAAAGGGAAAGAAAGGACGAAAGGGAAAGAAAGGACGAAAGGGAAAGAAAGGACGAAAGGGAAAGAAAGGAAAACCAATCCAACCTGAAGAAACACCAGCCATTCCGACGGAGATAAAAGCTGCAGAAATAGAAAAGGAACCAAAAACAGAAGTGGTTGTGGAACCAGTTATTCCGGAAGCAGATATTGCGGAGACCGAAATACAACAAATTAAAGCAGAAGTAGAACCAGTTGAAACAAAGCCAGAAATAGTTATGGAACCAGATGTGGAACCAGTTATTCCAGAAACGGAACTTACAGAGACCGGAAAAGAAGCGAAAGTAGAACAAGTTAAACCAGTGGAAGGGAAGCTAAACTTA------------------------------------------------------------------------------------------------------------------------------------------------------------------------------------------------------------------------------------------------------------------------------------------------------------------------------------------------------------------------------------------------------------------------------------------------------------------------------GGAAAAGGAAAGGGCAAAGGAAAGAAGGTTAGAAAGGGAAAGAAAGGACGAAAGGGAAAGAAAGGACGAAAGGGAAAGAAAGGACGGAAAGGAAAGAAGGGGCGAAAAGGAAAGAAAAGAAGAAAAGGAAAAAAAGGACGAAAGGGAAAGAAAGGACGAAAGGGAAAGAAAGGACGAAAGGGCAAGAAAGGACGAAAGGGAAAGAAAGGACGAAAGGGAAAGAAAGGACGAAAGGGAAAGAAAGGACGAAGGGGAAAGAAAGGA------------------CGAAAGGGCAAGAAAGGAAATCCAATCAAAACTGAAGAAACACCAGCCATTCTGACGGAGATAAAAGCTGCAGAATTAGAAAAGGAACCAAAAACAGAAATAGTTATGAAACCAGTTATTCCCGAAACGGAACTAACTGAGACCGGAAAAGAAGCAGAAGTAGAACAAGTTAAACCAGTGGAAAGGAAGCTAAAAATAGGAAAAGGAAAGGGCAAAGGAAAGAAGGTTAGAAAGGGAAAGAAAGGACGAAAGGGAAAGAAAGGACGAAAGGGAAAGAAAGGACGGAAAGGAAAGAAGGGGCGAAAAGGAAAGAAAAGAAGAAAAGGAAAGAAAAGACGAAAAGGAAAGAAACGACGAAAGGGAAAGAAAGGACGAAAGGGAAAAAAAGGACGAAAGGGAAAGAAAGGACGAAAGGGAAAAAAAGGACGAAAGGGAAAAAAAGGACGAAAGGGAAAGAAAGGACGAAAGGGAAAGAAAGGCAAACCAATCCAAACTGAAGAAACACCAGCCATTCCGACGGAGATAAAAGCTGCAGAAATAGAAAAGGAACCAAAAACAGAAGTGGTTGTGGAACCACTTATTCAGGAAAAAGATGTTGCGGAGACCGAAATACAACCCATCGAAGCAGAAGTAGAACCAGTTGAACCAAAGACAGAAATAGTTATGGAACCAGTTATTCCGGAAGCAGATATTGCGGAGACCGAAATACAACCCATCAAAGCAGAAGAAGAACCAGTT------------------------------------------------------------------------------------------------------------GAACCAAAGCCAGAAATAGTTATGGAACCAGATGAGGAACCAGTTATTCCGGAAGCAGATATTGCGGAGACCGAAATACAACCCATCAAAGCAGAAGAAGAACCAGTTGAACCAAAGCCAGAAATAGTTATGGAACCAGATGAGGAACCAGTTATTCCGGAAGCAGATATTGCGGAGACCGAAATACAACCCATCAAAGCAGAAGAAGAACCAGTTGAACCAAAGCCAGAAATAGTTATGGAACCAGATGAGGAACCAGTTATTCCGGAAGCAGATATTGCGGAGACCGAAATACAACCCATCAGAGCAGAAGAAGAACCAGTTGAACCAAAGCCAGAAATAGTTATGGAACCAGATGAGGAACCAGTCATTCCAGAAACAGAACTAACAGAGACAGACAAACAACCAATCGAAGCAGAAGTAGAACCAGTTGAACCAAAAACAGAAGTGGTTGTGGAACCAGTTATTCCGGAAGCAGATATTGCGGAGACCGAAATACAACCCATCGAAGCAGAAGTAGAACCAGTTGAACCAAAGCCAGAAATAGTTATGGAACCAGATGTGGAACCAGTAATTCCAGAAACAGATCTTACAGAGACCGAAAAACAACCAATCGAAGCAGAAGTAGAACCAGTTGAACCGAAGACAGAGATAGTCGAACCAGAAACAGAGCCAGTCGAAGAAGCCGAGGAAGAAGCGGTTGAAGCACCCGTTATGGAACCAGTTATACCTGAGATAGAACCAAAGACAGAACCGGAAGTTGAAGCCGAGGAAAAAGAAGTCGAAACAAACGTTGAACCAGAACTACTGGAGACAGGAAAACCGATTGAAATTGGCCAAGCAACTGAAATCGAGGAGCCACCAGAAAAACAACCGGTGGAATCGGAGTCACAACCAGCGGAAGTTGAGATGGAACAAATTGAAACAAAGGAAGAACAAACTGAACCAAAGGAAGAACTAGCAGGAATTGATGAGAAATGTAAGTTTTAGTAACAATTATTTATTTTATGATTCCTTTTGAGCTGTTTCGGTATGCTTCGATGCAAGTCAAATTTGTGGATGTTTTAAATCCATCATTCAATTATGTTTGCAATGTAGTCATGAAGTTTGTTCGTTTGTTCAACACAGATATGGGACATATACGCATGAGCAGACCTTTTTTGTGCACAGATCTTCGTTCAGGTTAATCGTGCATGGCAACAAAAATTGGTTCATTCTGTTTTATGTCGTGCAGTCTTATTTTGCTTGAATTAAGCATTGCTGTAAAGTTCAAGTTAAAGGTACAGTCCATCATTTGTAATTTGTGCATTTTGTTCGTTTGAAGAAACAAACATGCTCAAAATCTAAAGAAGGCTGCTGAACAACTAACCT---------------------------------------------------------------------------------------------------------------------------------------------------------------------------------------------------------------------------------------------------------------------------------------GATGAAGTTTCAGCTCAGCTTCAGCTGTATAAATTATTTCGAGAAAAACAAAATTCTAGGATCTCCATTTCAAATCAAAAGTTGCTGAATCTTGTTTTGAATTGGGCCCGAATTCAGCGTCCGAGGCACGTTCTCATCACTGAGCCGATTGCAGCTACGCGATGTCGCGCACGAATTAAAGCAATCGAGGGCGCGTGTGCGTTGTGTCAACGCTTGCGCGCGCTTCGCCAAAT-AAAAAAAAAGAACTAAACGGAAATTTCTTTAGACGCATGTCTTTAGACGCACCGTTGGAAAGGCCTACTTTCGTTGCTTTCTTCAAGATGACAGCATGCCAGAAAGAAAAATCTCTTATCTTTCAACGAAGTATGTTTTTTAACAATAGGCCTAATTACGTCAGTTACTTTTTGGATAGTTGCAAATAATGATGGACTGTAGCTTTAAGAAATTTGTGGAACTCAAGTTGTGCTTGGCAAGTTTTACTTTAGAATTCGAAAATGCATCGACACCAAAAAAAAGTGTAATTTTGGTCCTATAGTTGAAGTGTCAAACTAGAACAAATTTAATGATAAATTAAATAAATTTAAATAATTTATTCCTACCTTGCTTTGACAGTGATGAAGGAGCTGCGTGACCTTTTGGAATCTACGAAGATTGACCTTCCTGTTGACATCAATGATCCATACGACCTAGGTCTTCTTCTCAGACATTTACGTCACCATTCAAATCTTCTTGCTCGTATTGGAGACCCCGATGTCAAAAAGGAAGTCCTCAGCGCCATGAATGAAAAC

>CA04-B

GCACGACGTGCCGCAGAGGAAAAACCTCAAAAGGGAAAGAAGGGACGAAAAGGAAAGAAACGACGAAAGGGAAAGAAGGGACGAAAAGGAAAGAAGGGACGAAAAGGAAAGAAACGACGAAAGGGAAAGAAAAGACGAAAGGGAAAGAAAGGACGAAAGGGAAAGAAAGGACGAAAGGGAAAGAAAGGACGAAAGGGAAAGAAAGGACGAAAGGGAAAGAAAGGACGAAAGGGAAAGAAAGGACGAAAGGGAAAGAAAGGACGAAAGGGAAAGAAAGGAAAACCAATCCAACCTGAAGAAACACCAGCCATTCCGACGGAGATAAAAGCTGCAGAAATAGAAAAGGAACCAAAAACAGAAGTGGTTGTGGAACCAGTTATTCCGGAAGCAGATATTGCGGAGACCGAAATACAACAAATTAAAGCAGAAGTAGAACCAGTTGAAACAAAGCCAGAAATAGTTATGGAACCAGATGTGGAACCAGTTATTCCAGAAACGGAACTTACAGAGACCGGAAAAGAAGCGAAAGTAGAACAAGTTAAACAAGTGGAAGGGAAGCTAAACTTA------------------------------------------------------------------------------------------------------------------------------------------------------------------------------------------------------------------------------------------------------------------------------------------------------------------------------------------------------------------------------------------------------------------------------------------------------------------------------GGAAAAGGAAAGGGCAAAGGAAAGAAGGTTAGAAAGGGAAAGAAAGGACGAAAGGGAAAGAAAGGACGAAAGGGAAAGAAAGGACGGAAAGGAAAGAAGGGGCGAAAAGGAAAGAAAAGAAGAAAAGGAAAAAAAGGACGAAAGGGAAAGAAAGGACGAAAGGGAAAGAAAGGACGAAAGGGCAAGAAAGGACGAAAGGGAAAGAAAGGACGAAAGGGAAAGAAAGGACGAAAGGGAAAGAAAGGACGAAGGGGAAAGAAAGGA------------------CGAAAGGGCAAGAAAGGAAATCCAATCAAAACTGAAGAAACACCAGCCATTCTGACGGAGATAAAAGCTGCAGAATTAGAAAAGGAACCAAAAACAGAAATAGTTATGAAACCAGTTATTCCCGAAACGGAACTAACAGAGACCGGAAAAGAAGCAGAAGTAGAACAAGTTAAACCAGTGGAAAGGAAGCTAAAAATAGGAAAAGGAAAGGGCAAAGGAAAGAAGGTTAGAAAGGGAAAGAAAGGACGAAAGGGAAAGAAAGGACGAAAGGGAAAGAAAGGACGGAAAGGAAAGAAGGGGCGAAAAGGAAAGAAAAGAAGAAAAGGAAAGAAAAGACGAAAAGGAAAGAAACGACGAAAGGGAAAGAAAGGACGAAAGGGAAAAAAAGGACGAAAGGGAAAGAAAGGACGAAAGGGAAAAAAAGGACGAAAGGGAAAAAAAGGACGAAAGGGAAAGAAAGGACGAAAGGGAAAGAAAGGCAAACCAATCCAAACTGAAGAAACACCAGCCATTCCGACGGAGATAAAAGCTGCAGAAATAGAAAAGGAACCAAAAACAGAAGTGGTTGTGGAACCACTTATTCAGGAAAAAGATGTTGCGGAGACCGAAATACAACCCATCGAAGCAGAAGTAGAACCAGTTGAACCAAAGACAGAAATAGTTATGGAACCAGTTATTCCGGAAGCAGATATTGCGGAGACCGAAATACAACCCATCAAAGCAGAAGAAGAACCAGTT------------------------------------------------------------------------------------------------------------GAACCAAAGCCAGAAATAGTTATGGAACCAGATGAGGAACCAGTTATTCCGGAAGCAGATATTGCGGAGACCGAAATACAACCCATCAAAGCAGAAGAAGAACCAGTTGAACCAAAGCCAGAAATAGTTATGGAACCAGATGAGGAACCAGTTATTCCGGAAGCAGATATTGCGGAGACCGAAATACAACCCATCAAAGCAGAAGAAGAACCAGTTGAACCAAAGCCAGAAATAGTTATGGAACCAGATGAGGAACCAGTTATTCCGGAAGCAGATATTGCGGAGACCGAAATACAACCCATCAGAGCAGAAGAAGAACCAGTTGAACCAAAGCCAGAAATAGTTATGGAACCAGATGAGGAACCAGTCATTCCAGAAACAGAACTAACAGAGACAGACAAACAACCAATCGAAGCAGAAGTAGAACCAGTTGAACCAAAAACAGAAGTGGTTGTGGAACCAGTTATTCCGGAAGCAGATATTGCGGAGACCGAAATACAACCCATCGAAGCAGAAGTAGAACCAGTTGAACCAAAGCCAGAAATAGTTATGGAACCAGATGTGGAACCAGTAATTCCAGAAACAGATCTTACAGAGACCGAAAAACAACCAATCGAAGCAGAAGTAGAACCAGTTGAACCGAAGACAGAGATAGTCGAACCAGAAACAGAGCCAGTCGAAGAAGCCGAGGAAGAAGCGGTTGAAGCACCCGTTATGGAACCAGTTATACCTGAGATAGAACCAAAGACAGAACCGGAAGTTGAAGCCGAGGAAAAAGAAGTCGAAACAAACGTTGAACCAGAACTACTGGAGACAGGAAAACCGATTGAAATTGGCCAAGCAACTGAAATCGAGGAGCCACCAGAAAAACAACCGGTGGAATCGGAGTCACAACCAGCGGAAGTTGAGATGGAACAAATTGAAACAAAGGAAGAACAAACTGAACCAAAGGAAGAACTAGCAGGAATTGATGAGAAATGTAAGTTTTAGTAACAATTATTTATTTTATGATTCCTTTTGAGCTGTTTCGGTATGCTTCGATGCAAGTCAAATTTGTGGATGTTTTAAATCCATCATTCAATTATGTTTGCAATGTAGTCATGAAGTTTGTTCGTTTGTTCAACACAGATATGGGACATATACGCATGAGCAGACCTTTTTTGTGCACAGATCTTCGTTCAGGTTAATCGTGCATGGCAACAAAAATTGGTTCATTCCGTTTTATGTCGTGCAGTCTTATTTTGCTTGAATTAAGCATTGCTGTAAAGTTCAAGTTAAAGGTACAGTCCATCATTTGTAATTTGTGCATTTTGTTCGTTTGAAGAAACAAACATGCTCAAAATCTAAAGAAGGCTGCTGAACAACTAACCT---------------------------------------------------------------------------------------------------------------------------------------------------------------------------------------------------------------------------------------------------------------------------------------GATGAAGTTTCAGCTCAGCTTCAGCTGTATAAATTATTTCGAGAAAAACAAAATTCTAGGATCTCCATTTCAAATCAAAAGTTGCTGAATCTTGTTTTGAATTGGGCCTGAATTCAGCGTCCGAGGCACGTTCTCATCACTGAGCCGATTGCAGCTACGCGATGTCGCGCACGAATTAAAGCAATCGAGGGCGCGTGTGCGTTGTGTCAACGCTTGCGCGCGCTTCGCCAAATAAAAAAAAAAGAACTAAACGGAAATTTCTTTAGACGCATGTCTTTAGACGCACCGTTGGAAAGGCCTACTTTCGTTGCTTTCTTCAAAATGACAGCATGCCAGAAAGAAAAATCTCTTATCTTTCAACGAAGTATGTTTTTTAACAATAGGCCTAATTACGTCAGTTACTTTTTGGATAGTTGCAAATAATGATGGACTGTAGCTTTAAGAAATTTGTGGAACTCAAGTTGTGCTTGGCAAGTTTTACTTTAGAATTCGAAAATGCATCGACACCAAAAAAAAGTGTAATTTTGGTCCTATAGTTGAAGTGTCAAACTAGAACAAATTTAATGATAAATTAAATAAATTTAAATAATTTATTCCTACCTTGCTTTGACAGTGATGAAGGAGCTGCGTGACCTTTTGGAATCTACGAAGATTGACCTTCCTGTTGACATCAATGATCCATACGACCTAGGTCTTCTTCTCAGACATTTACGTCACCATTCAAATCTTCTTGCTCGTATTGGAGACCCCGATGTCAAAAAGGAAGTCCTCAGCGCCATGAATGAAAAC

>CA02-B

GCACGACGTGCCGCAGAGGAAAAACCTCAAAAGGGAAAGAAGGGACGAAAAGGAAAGAAACGACGAAAGGGAAAGAAGGGACGAAAAGGAAAGAAGGGACGAAAAGGAAAGAAACGACGAAAGGGAAAGAAAAGACGAAAGGGAAAGAAAGGACGAAAGGGAAAGAAAGGACGAAAGGGAAAGAAAGGACGAAAGGGAAAGAAAGGACGAAAGGGAAAGAAAGGACGAAAGGGAAAGAAAGGACGAAAGGGAAAGAAAGGACGAAAGGGAAAGAAAGGAAAACCAATCCAACCTGAAGAAACACCAGCCATTCCGACGGAGATAAAAGCTGCAGAAATAGAAAAGGAACCAAAAACAGAAGTGGTTGTGGAACCAGTTATTCCGGAAGCAGATATTGCGGAGACCGAAATACAACAAATTAAAGCAGAAGTAGAACCAGTTGAAACAAAGCCAGAAATAGTTATGGAACCAGATGTGGAACCAGTTATTCCAGAAACGGAACTTACAGAGACCGGAAAAGAAGCGAAAGTAGAACAAGTTAAACCAGTGGAAGGGAAGCTAAACTTA------------------------------------------------------------------------------------------------------------------------------------------------------------------------------------------------------------------------------------------------------------------------------------------------------------------------------------------------------------------------------------------------------------------------------------------------------------------------------GGAAAAGGAAAGGGCAAAGGAAAGAAGGTTAGAAAGGGAAAGAAAGGACGAAAGGGAAAGAAAGGACGAAAGGGAAAGAAAGGACGGAAAGGAAAGAAGGGGCGAAAAGGAAAGAAAAGAAGAAAAGGAAAAAAAGGACGAAAGGGAAAGAAAGGACGAAAGGGAAAGAAAGGACGAAAGGGCAAGAAAGGACGAAAGGGAAAGAAAGGACGAAAGGGAAAGAAAGGACGAAAGGGAAAGAAAGGACGAAGGGGAAAGAAAGGA------------------CGAAAGGGCAAGAAAGGAAATCCAATCAAAACTGAAGAAACACCAGCCATTCTGACGGAGATAAAAGCTGCAGAATTAGAAAAGGAACCAAAAACAGAAATAGTTATGAAACCAGTTATTCCCGAAACGGAACTAACAGAGACCGGAAAAGAAGCAGAAGTAGAACAAGTTAAACCAGTGGAAAGGAAGCTAAAAATAGGAAAAGGAAAGGGCAAAGGAAAGAAG------------------AGGAGAAAGGGAAAGAAAGGACGAAAGGGAAAGAAAGGACGGAAAGGAAAGAAGGGGCGT------------------AAAGGAAAGAAAAGACGAAAAGGAAAGAAACGACGAAAGGGAAAGAAAGGACGAAAGGGAAAAAAAGGACGAAAGGGAAAGAAAGGACGAAAGGGAAAAAAAGGACGAAAGGGAAAAAAAGGACGAAAGGGAAAGAAAGGACGAAAGGGAAAGAAAGGAAAACCAATCCAAACTGAAGAAACACCAGCCATTCCGACGGAGATAAAAGCTGCAGAAATAGAAAAGGAACCAAAAACAGAAGTGGTTGTGGAACCACTTATTCAGGAAAAAGATGTTGCGGAGACCGAAATACAACCCATCGAAGCAGAAGTAGAACCAGTTGAACCAAAGACAGAAATAGTTATGGAACCAGTTATTCCGGAAGCAGATATTGCGGAGACCGAAATACAACCCATCAAAGCAGAAGAAGAACCAGTT------------------------------------------------------------------------------------------------------------GAACCAAAGCCAGAAATAGTTATGGAACCAGATGAGGAACCAGTTATTCCGGAAGCAGATATTGCGGAGACCGAAATACAACCCATCAAAGCAGAAGAAGAACCAGTTGAACCAAAGCCAGAAATAGTTATGGAACCAGATGAGGAACCAGTTATTCCGGAAGCAGATATTGCGGAGACCGAAATACAACCCATCAAAGCAGAAGAAGAACCAGTTGAACCAAAGCCAGAAATAGTTATGGAACCAGATGAGGAACCAGTTATTCCGGAAGCAGATATTGCGGAGACCGAAATACAACCCATCAGAGCAGAAGAAGAACCAGTTGAACCAAAGCCAGAAATAGTTATGGAACCAGATGAGGAACCAGTCATTCCAGAAACAGAACTAACAGAGACAGACAAACAACCAATCGAAGCAGAAGTAGAACCAGTTGAACCAAAAACAGAAGTGGTTGTGGAACCAGTTATTCCGGAAGCAGATATTGCGGAGACCGAAATACAACCCATCGAAGCAGAAGTAGAACCAGTTGAACCAAAGCCAGAAATAGTTATGGAACCAGATGTGGAACCAGTAATTCCAGAAACAGATCTTACAGAGACCGAAAAACAACCAATCGAAGCAGAAGTAGAACCAGTTGAACCGAAGACAGAGATAGTCGAACCAGAAACAGAGCCAGTCGAAGAAGCCGAGGAAGAAGCGGTTGAAGCACCCGTTATGGAACCAGTTATACCTGAGATAGAACCAAAGACAGAACCGGAAGTTGAAGCCGAGGAAAAAGAAGTCGAAACAAACGTTGAACCAGAACTACTGGAGACAGGAAAACCGATTGAAATTGGCCAAGCAACTGAAATCGAGGAGCCACCAGAAAAACAACCGGTGGAATCGGAGTCACAACCAGCGGAAGTTGAGATGGAACAAATTGAAACAAAGGAAGAACCAACTGAACCAAAGGAAGAACTAGCAGGAATTGATGAGAAATGTAAGTTTTAGTAACAATTATTTATTTTATGATTCCTTTTGAGCTGTTTCGGTATGCTTCGATGCAAGTCAAATTTGTGGATGTTTTAAATCCATCATTCAATTATGTTCGCAATGTAGTCATGAAGTTTGTTCGTTTGTTCAACACAGATATGGGACATATACGCATGAGCAGACCTTTTTTGTGCACAGATCTTCGTTCAGGTTAATCGTGCATGGCAACAAAAATTGGTTCATTCCGTTTTATGTCGTGCAGTCTTATTTTGCTTGAATTAAGCATTGCTGTAAAGTTCAAGTTAAAGGTACAGTCCATCATTTGTAATTTGTGCATTTTGTTCGTTTGAAGAAACAAACATGCTCAAAATCTAAAGAAGGCTGCTGAACAACTAACCT---------------------------------------------------------------------------------------------------------------------------------------------------------------------------------------------------------------------------------------------------------------------------------------GATGAAGTTTCAGCTCAGCTTCAGCTGTATAAATTATTTCGAGAAAAACAAAATTCTAGGATCTCCATTTCAAATCAAAAGTTGCTGAATCTTGTTTTGAATTGGGCCTGAATTCAGCGTCCGAGGCACGTTCTCATCACTGAGCCGATTGCGGCTACGCGATGTCGCGCACGAATTAAAGCAATCGAGGGCGCGTGTGCGTTGTGTCAACGCTTGCGCGCGCTTCGCCAAAT-AAAAAAAAAGAACTAAACGGAAATTTCTTTAGACGCATGTCTTTAGACGCACCGTTGGAAAGGCCTACTTTCGTTGCTTTCTTCAAAATGACAGCATGCCAGAAAGAAAAATCTCTTATCTTTCAACGAAGTATGTTTTTTAACAATAGGCCTAATTACGTCAGTTACTTTTTGGATAGTTGCAAATAATGATGGACTGTAGCTTTAAGAAATTTGTGGAACTAAAGTTGTGCTTGGCAAGTTTTACTTTAGAATTCGAAAATGCATCGACACC-AAAAAAAGTGTAATTTTGGTCCTATAGTTGAAGTGTCAAACTAGAACAAATTTAATGATAAATTAAATAAATTTAAATAATTTATTCCTACCTTGCTTTGACAGTGATGAAGGAGCTGCGTGACCTTTTGGAATCTACGAAGATTGACCTTCCTGTTGACATCAATGATCCATACGACCTAGGTCTTCTTCTCAGACATTTACGTCACCATTCAAATCTTCTTGCTCGTATTGGAGACCCCGATGTCAAAAAGGAAGTCCTCAGCGCCATGAATGAAAAC

>SS01-A

GCACGACGTGCCGCAGAGGAAAAACCTCAAAAGGGAAAGAAGGGACGAAAAGGAAAGAAACGACGAAAGGGAAAGAAGGGACGAAAAGGAAAGAAGGGACGAAAAGGAAAGAAACGACGAAAGGGAAAGAAAAGACGAAAGGGAAAGAAAGGACGAAAGGGAAAGAAAGGACGAAAGGGAAAGAAAGGACGAAAGGGAAAGAAAGGACGAAAGGGAAAGAAAGGACGAAAGGGAAAGAAAGGACGAAAGGGAAAGAAAGGACGAAAGGGAAAGAAAGGAAAACCAATCCAACCTGAAGAAACACCAGCCATTCCGACGGAGATAAAAGCTGCAGAAATAGAAAAGGAACCAAAAACAGAAGTGGTTGTGGAACCAGTTATTCCGGAAGCAGATATTGCGGAGACCGAAATACAACAAATTAAAGCAGAAGTAGAACCAGTTGAAACAAAGCCAGAAATAGTTATGGAACCAGATGTGGAACCAGTTATTCCAGAAACGGAACTTACAGAGACCGGAAAAGAAGCGAAAGTAGAACAAGTTAAACCAGTGGAAGGGAAGCTAAACTTA------------------------------------------------------------------------------------------------------------------------------------------------------------------------------------------------------------------------------------------------------------------------------------------------------------------------------------------------------------------------------------------------------------------------------------------------------------------------------GGAAAAGGAAAGGGCAAAGGAAAGAAGGTTAGAAAGGGAAAGAAAGGACGAAAGGGAAAGAAAGGACGAAAGGGAAAGAAAGGACGGAAAGGAAAGAAGGGGCGAAAAGGAAAGAAAAGAAGAAAAGGAAAAAAAGGACGAAAGGGAAAGAAAGGACGAAAGGGAAAGAAAGGACGAAAGGGCAAGAAAGGACGAAAGGGAAAGAAAGGACGAAAGGGAAAGAAAGGACGAAAGGGAAAGAAAGGACGAAGGGGAAAGAAAGGA------------------CGAAAGGGCAAGAAAGGAAATCCAATCAAAACTGAAGAAACACCAGCCATTCTGACGGAGATAAAAGCTGCAGAATTAGAAAAGGAACCAAAAACAGAAATAGTTATGAAACCAGTTATTCCCGAAACGGAACTAACAGAGACCGGAAAAGAAGCAGAAGTAGAACAAGTTAAACCAGTGGAAAGGAAGCTAAAAATAGGAAAAGGAAAGGGCAAAGGAAAGAAGGTTAGAAAGGGAAAGAAAGGACGAAAGGGAAAGAAAGGACGAAAGGGAAAGAAAGGACGGAAAGGAAAGAAGGGGCGAAAAGGAAAGAAAAGAAGAAAAGGAAAGAAAAGACGAAAAGGAAAGAAACGACGAAAGGGAAAGAAAGGACGAAAGGGAAAAAAAGGACGAAAGGGAAAGAAAGGACGAAAGGGAAAAAAAGGACGAAAGGGAAAAAAAGGACGAAAGGGAAAGAAAGGACGAAAGGGAAAGAAAGGCAAACCAATCCAAACTGAAGAAACACCAGCCATTCCGACGGAGATAAAAGCTGCAGAAATAGAAAAGGAACCAAAAACAGAAGTGGTTGTGGAACCACTTATTCAGGAAAAAGATGTTGCGGAGACCGAAATACAACCCATCGAAGCAGAAGTAGAACCAGTTGAACCAAAGACAGAAATAGTTATGGAACCAGTTATTCCGGAAGCAGATATTGCGGAGACCGAAATACAACCCATCAAAGCAGAAGAAGAACCAGTT------------------------------------------------------------------------------------------------------------GAACCAAAGCCAGAAATAGTTATGGAACCAGATGAGGAACCAGTTATTCCGGAAGCAGATATTGCGGAGACCGAAATACAACCCATCAAAGCAGAAGAAGAACCAGTTGAACCAAAGCCAGAAATAGTTATGGAACCAGATGAGGAACCAGTTATTCCGGAAGCAGATATTGCGGAGACCGAAATACAACCCATCAAAGCAGAAGAAGAACCAGTTGAACCAAAGCCAGAAATAGTTATGGAACCAGATGAGGAACCAGTTATTCCGGAAGCAGATATTGCGGAGACCGAAATACAACCCATCAGAGCAGAAGAAGAACCAGTTGAACCAAAGCCAGAAATAGTTATGGAACCAGATGAGGAACCAGTCATTCCAGAAACAGAACTAACAGAGACAGACAAACAACCAATCGAAGCAGAAGTAGAACCAGTTGAACCAAAAACAGAAGTGGTTGTGGAACCAGTTATTCCGGAAGCAGATATTGCGGAGACCGAAATACAACCCATCGAAGCAGAAGTAGAACCAGTTGAACCAAAGCCAGAAATAGTTATGGAACCAGATGTGGAACCAGTAATTCCAGAAACAGATCTTACAGAGACCGAAAAACAACCAATCGAAGCAGAAGTAGAACCAGTTGAACCGAAGACAGAGATAGTCGAACCAGAAACAGAGCCAGTCGAAGAAGCCGAGGAAGAAGCGGTTGAAGCACCCGTTATGGAACCAGTTATACCTGAGATAGAACCAAAGACAGAACCGGAAGTTGAAGCCGAGGAAAAAGAAGTCGAAACAAACGTTGAACCAGAACTACTGGAGACAGGAAAACCGATTGAAATTGGCCAAGCAACTGAAATCGAGGAGCCACCAGAAAAACAACCGGTGGAATCGGAGTCACAACCAGCGGAAGTTGAGATGGAACAAATTGAAACAAAGGAAGAACAAACTGAACCAAAGGAAGAACTAGCAGGAATTGATGAGAAATGTAAGTTTTAGTAACAATTATTTATTTTATGATTCCTTTTGAGCTGTTTCGGTATGCTTCGATGCAAGTCAAATTTGTGGATGTTTTAAATCCATCATTCAATTATGTTTGCAATGTAGTCATGAAGTTTGTTCGTTTGTTCAACACAGATATGGGACATATACGCATGAGCAGACCTTTTTTGTGCACAGATCTTCGTTCAGGTTAATCGTGCATGGCAACAAAAATTGGTTCATTCCGTTTTATGTCGTGCAGTCTTATTTTGCTTGAATTAAGCATTGCTGTTAAGTTCAAGTTAAAGGTACAGTCCATCATTTGTAATTTGTGCATTTTGTTCGTTTGAAGAAACAAACATGCTCAAAATCTAAAGAAGGCTGCTGAACAACTAACCT---------------------------------------------------------------------------------------------------------------------------------------------------------------------------------------------------------------------------------------------------------------------------------------GATGAAGTTTCAGCTCAGCTTCAGCTGTATAAATTATTTCGAGAAAAACAAAATTCTAGGATCTCCATTTCAAATCAAAAGTTGCTGAATCTTGTTTTGAATTGGGCCTGAATTCAGCGTCCGAGGCACGTTCTCATCACTGAGCCGATTGCAGCTACGCGATGTCGCGCACGAATTAAAGCAATCGAGGGCGCGTGTGCGTTGTGTCAACGCTTGCGCGCGCTTCGCCAAATAAAAAAAAAAGAACTAAACGGAAATTTCTTTAGACGCATGTCTTTAGACGCACCGTTGGAAAGGCCTACTTTCGTTGCTTTCTTCAAAATGACAGCATGCCAGAAAGAAAAATCTCTTATCTTTCAACGAAGTATGTTTTTTAACAATAGGCCTAATTACGTCAGTTACTTTTTGGATAGTTGCAAATAATGATGGACTGTAGCTTTAAGAAATTTGTGGAACTCAAGTTGTGCTTGGCAAGTTTTACTTTAGAATTCGAAAATGCATCGACACCAAAAAAAAGTGTAATTTTGGTCCTATAGTTGAAGTGTCAAACTAGAACAAATTTAATGATAAATTAAATAAATTTAAATAATTTATTCCTACCTTGCTTTGACAGTGATGAAGGAGCTGCGTGACCTTTTGGAATCTACGAAGATTGACCTTCCTGTTGACATCAATGATCCATACGACCTAGGTCTTCTTCTCAGACATTTACGTCACCATTCAAATCTTCTTGCTCGTATTGGAGACCCCGATGTCAAAAAGGAAGTCCTCAGCGCCATGAATGAAAAC

>SS01-B

GCACGACGTGCCGCAGAGGAAAAACCTCAAAAGGGAAAGAAGGGACGAAAAGGAAAGAAACGACGAAAGGGAAAGAAGGGACGAAAAGGAAAGAAGGGACGAAAAGGAAAGAAACGACGAAAGGGAAAGAAAAGACGAAAGGGAAAGAAAGGACGAAAGGGAAAGAAAGGACGAAAGGGAAAGAAAGGACGAAAGGGAAAGAAAGGACGAAAGGGAAAGAAAGGACGAAAGGGAAAGAAAGGACGAAAGGGAAAGAAAGGACGAAAGGGAAAGAAAGGAAAACCAATCCAACCTGAAGAAACACCAGCCATTCCGACGGAGATAAAAGCTGCAGAAATAGAAAAGGAACCAAAAACAGAAGTGGTTGTGGAACCAGTTATTCCGGAAGCAGATATTGCGGAGACCGAAATACAACAAATTAAAGCAGAAGTAGAACCAGTTGAAACAAAGCCAGAAATAGTTATGGAACCAGATGTGGAACCAGTTATTCCAGAAACGGAACTTACAGAGACCGGAAAAGAAGCGAAAGTAGAACAAGTTAAACCAGTGGAAGGGAAGCTAAACTTA------------------------------------------------------------------------------------------------------------------------------------------------------------------------------------------------------------------------------------------------------------------------------------------------------------------------------------------------------------------------------------------------------------------------------------------------------------------------------GGAAAAGGAAAGGGCAAAGGAAAGAAGGTTAGAAAGGGAAAGAAAGGACGAAAGGGAAAGAAAGGACGAAAGGGAAAGAAAGGACGGAAAGGAAAGAAGGGGCGAAAAGGAAAGAAAAGAAGAAAAGGAAAAAAAGGACGAAAGGGAAAGAAAGGACGAAAGGGAAAGAAAGGACGAAAGGGCAAGAAAGGACGAAAGGGAAAGAAAGGACGAAAGGGAAAGAAAGGACGAAAGGGAAAGAAAGGACGAAGGGGAAAGAAAGGA------------------CGAAAGGGCAAGAAAGGAAATCCAATCAAAACTGAAGAAACACCAGCCATTCTGACGGAGATAAAAGCTGCAGAATTAGAAAAGGAACCAAAAACAGAAATAGTTATGAAACCAGTTATTCCCGAAACGGAACTAACAGAGACCGGAAAAGAAGCAGAAGTAGAACAAGTTAAACCAGTGGAAAGGAAGCTAAAAATAGGAAAAGGAAAGGGCAAAGGAAAGAAGGTTAGAAAGGGAAAGAAAGGACGAAAGGGAAAGAAAGGACGAAAGGGAAAGAAAGGACGGAAAGGAAAGAAGGGGCGAAAAGGAAAGAAAAGAAGAAAAGGAAAGAAAAGACGAAAAGGAAAGAAACGACGAAAGGGAAAGAAAGGACGAAAGGGAAAAAAAGGACGAAAGGGAAAGAAAGGACGAAAGGGAAAAAAAGGACGAAAGGGAAAAAAAGGACGAAAGGGAAAGAAAGGACGAAAGGGAAAGAAAGGCAAACCAATCCAAACTGAAGAAACACCAGCCATTCCGACGGAGATAAAAGCTGCAGAAATAGAAAAGGAACCAAAAACAGAAGTGGTTGTGGAACCACTTATTCAGGAAAAAGATGTTGCGGAGACCGAAATACAACCCATCGAAGCAGAAGTAGAACCAGTTGAACCAAAGACAGAAATAGTTATGGAACCAGTTATTCCGGAAGCAGATATTGCGGAGACCGAAATACAACCCATCAAAGCAGAAGAAGAACCAGTT------------------------------------------------------------------------------------------------------------GAACCAAAGCCAGAAATAGTTATGGAACCAGATGAGGAACCAGTTATTCCGGAAGCAGATATTGCGGAGACCGAAATACAACCCATCAAAGCAGAAGAAGAACCAGTTGAACCAAAGCCAGAAATAGTTATGGAACCAGATGAGGAACCAGTTATTCCGGAAGCAGATATTGCGGAGACCGAAATACAACCCATCAAAGCAGAAGAAGAACCAGTTGAACCAAAGCCAGAAATAGTTATGGAACCAGATGAGGAACCAGTTATTCCGGAAGCAGATATTGCGGAGACCGAAATACAACCCATCAGAGCAGAAGAAGAACCAGTTGAACCAAAGCCAGAAATAGTTATGGAACCAGATGAGGAACCAGTCATTCCAGAAACAGAACTAACAGAGACAGACAAACAACCAATCGAAGCAGAAGTAGAACCAGTTGAACCAAAAACAGAAGTGGTTGTGGAACCAGTTATTCCGGAAGCAGATATTGCGGAGACCGAAATACAACCCATCGAAGCAGAAGTAGAACCAGTTGAACCAAAGCCAGAAATAGTTATGGAACCAGATGTGGAACCAGTAATTCCAGAAACAGATCTTACAGAGACCGAAAAACAACCAATCGAAGCAGAAGTAGAACCAGTTGAACCGAAGACAGAGATAGTCGAACCAGAAACAGAGCCAGTCGAAGAAGCCGAGGAAGAAGCGGTTGAAGCACCCGTTATGGAACCAGTTATACCTGAGATAGAACCAAAGACAGAACCGGAAGTTGAAGCCGAGGAAAAAGAAGTCGAAACAAACGTTGAACCAGAACTACTGGAGACAGGAAAACCGATTGAAATTGGCCAAGCAACTGAAATCGAGGAGCCACCAGAAAAACAACCGGTGGAATCGGAGTCACAACCAGCGGAAGTTGAGATGGAACAAATTGAAACAAAGGAAGAACCAACTGAACCAAAGGAAGAACTAGCAGGAATTGATGAGAAATGTAAGTTTTAGTAACAATTATTTATTTTATGATTCCTTTTGAGCTGTTTCGGTATGCTTCGATGCAAGTCAAATTTGTGGATGTTTTAAATCCATCATTCAATT--GTTTGCAATGTAGTCATGAAGTTTGTTCGTTTGTTCAACACAGATATGGGACATATACGCATGAGCAGACCTTTTTTGTGCACAGATCTTCGTTCAGGTTAATCGTGCATGGCAACAAAAATTGGTTCATTCCGTTTTATGTCGTGCAGTCTTATTTTGCTTGAATTAAGCATTGCTGTAAAGTTCAAGTTAAAGGTACAGTCCATCATTTGTAATTTGTGCATTTTGTTCGTTTGAAGAAACAAACATGCTCAAAATCTAAAGAAGGCTGCTGAACAACTAACCT---------------------------------------------------------------------------------------------------------------------------------------------------------------------------------------------------------------------------------------------------------------------------------------GATGAAGTTTCAGCTCAGCTTCAGCTGTATAAATTATTTCGAGAAAAACAAAATTCTAGGATCTCCATTTCAAATCAAAAGTTGCTGAATCTTGTTTTGAATTGGGCCTGAATTCAGCGTCCGAGGCACGTTCTCATCACTGAGCCGATTGCAGCTACGCGATGTCGCGCACGAATTAAAGCAATCGAGGGCGCGTGTGCGTTGTGTCAACGCTTGCGCGCGCTTCGCCAAATAAAAAAAAAAGAACTAAACGGAAATTTCTTTAGACGCATGTCTTTAGACGCACCGTTGGAAAGGCCTACTTTCGTTGCTTTCTTCAAAATGACAGCATGCCAGAAAGAAAAATCTCTTATCTTTCAACGAAGTATGTTTTTTAACAATAGGCCTAATTACGTCAGTTACTTTTTGGATAGTTGCAAATAATGATGGACTGTAGCTTTAAGAAATTTGTGGAACTAAAGTTGTGCTTGGCAAGTTTTACTTTAGAATTCGAAAATGCATCGACACC-AAAAAAAGTGTAATTTTGGTCCTATAGTTGAAGTGTCAAACTAGAACAAATTTAATGATAAATTAAATAAATTTAAATAATTTATTCCTACCTTGCTTTGACAGTGATGAAGGAGCTGCGTGACCTTTTGGAATCTACGAAGATTGACCTTCCTGTTGACATCAATGATCCATACGACCTAGGTCTTCTTCTCAGACATTTACGTCACCATTCAAATCTTCTTGCTCGTATTGGAGACCCCGATGTCAAAAAGGAAGTCCTCAGCGCCATGAATGAAAAC

>SS29-A

GCACGACGTGCCGCAGAGGAAAAACCTCAAAAGGGAAAGAAGGGACGAAAAGGAAAGAAACGACGAAAGGGAAAGAAGGGACGAAAAGGAAAGAAGGGACGAAAAGGAAAGAAACGACGAAAGGGAAAGAAAAGACGAAAGGGAAAGAAAGGACGAAAGGGAAAGAAAGGACGAAAGGGAAAGAAAGGACGAAAGGGAAAGAAAGGACGAAAGGGAAAGAAAGGACGAAAGGGAAAGAAAGGACGAAAGGGAAAGAAAGGACGAAAGGGAAAGAAAGGAAAACCAATCCAACCTGAAGAAACACCAGCCATTCCGACGGAGATAAAAGCTGCAGAAATAGAAAAGGAACCAAAAACAGAAGTGGTTGTGGAACCAGTTATTCCGGAAGCAGATATTGCGGAGACCGAAATACAACAAATTAAAGCAGAAGTAGAACCAGTTGAAACAAAGCCAGAAATAGTTATGGAACCAGATGTGGAACCAGTTATTCCAGAAACGGAACTTACAGAGACCGGAAAAGAAGCGAAAGTAGAACAAGTTAAACCAGTGGAAGGGAAGCTAAACTTA------------------------------------------------------------------------------------------------------------------------------------------------------------------------------------------------------------------------------------------------------------------------------------------------------------------------------------------------------------------------------------------------------------------------------------------------------------------------------GGAAAAGGAAAGGGCAAAGGAAAGAAGGTTAGAAAGGGAAAGAAAGGACGAAAGGGAAAGAAAGGACGAAAGGGAAAGAAAGGACGGAAAGGAAAGAAGGGGCGAAAAGGAAAGAAAAGAAGAAAAGGAAAAAAAGGACGAAAGGGAAAGAAAGGACGAAAGGGAAAGAAAGGACGAAAGGGCAAGAAAGGACGAAAGGGAAAGAAAGGACGAAAGGGAAAGAAAGGACGAAAGGGAAAGAAAGGACGAAGGGGAAAGAAAGGA------------------CGAAAGGGCAAGAAAGGAAATCCAATCAAAACTGAAGAAACACCAGCCATTCTGACGGAGATAAAAGCTGCAGAATTAGAAAAGGAACCAAAAACAGAAATAGTTATGAAACCAGTTATTCCCGAAACGGAACTAACTGAGACCGGAAAAGAAGCAGAAGTAGAACAAGTTAAACCAGTGGAAAGGAAGCTAAAAATAGGAAAAGGAAAGGGCAAAGGAAAGAAGGTTAGAAAGGGAAAGAAAGGACGAAAGGGAAAGAAAGGACGAAAGGGAAAGAAAGGACGGAAAGGAAAGAAGGGGCGAAAAGGAAAGAAAAGAAGAAAAGGAAAGAAAAGACGAAAAGGAAAGAAACGACGAAAGGGAAAGAAAGGACGAAAGGGAAAAAAAGGACGAAAGGGAAAGAAAGGACGAAAGGGAAAAAAAGGACGAAAGGGAAAAAAAGGACGAAAGGGAAAGAAAGGACGAAAGGGAAAGAAAGGCAAACCAATCCAAACTGAAGAAACACCAGCCATTCCGACGGAGATAAAAGCTACAGAAATAGAAAAGGAACCAAAAACAGAAGTGGTTGTGGAACCACTTATTCAGGAAAAAGATGTTGCGGAGACCGAAATACAACCCATCGAAGCAGAAGTAGAACCAGTTGAACCAAAGACAGAAATAGTTATGGAACCAGTTATTCCGGAAGCAGATATTGCGGAGACCGAAATACAACCCATCAAAGCAGAAGAAGAACCAGTT------------------------------------------------------------------------------------------------------------GAACCAAAGCCAGAAATAGTTATGGAACCAGATGAGGAACCAGTTATTCCGGAAGCAGATATTGCGGAGACCGAAATACAACCCATCAAAGCAGAAGAAGAACCAGTTGAACCAAAGCCAGAAATAGTTATGGAACCAGATGAGGAACCAGTTATTCCGGAAGCAGATATTGCGGAGACCGAAATACAACCCATCAAAGCAGAAGAAGAACCAGTTGAACCAAAGCCAGAAATAGTTATGGAACCAGATGAGGAACCAGTTATTCCGGAAGCAGATATTGCGGAGACCGAAATACAACCCATCAGAGCAGAAGAAGAACCAGTTGAACCAAAGCCAGAAATAGTTATGGAACCAGATGAGGAACCAGTCATTCCAGAAACAGAACTAACAGAGACAGACAAACAACCAATCGAAGCAGAAGTAGAACCAGTTGAACCAAAAACAGAAGTGGTTGTGGAACCAGTTATTCCGGAAGCAGATATTGCGGAGACCGAAATACAACCCATCGAAGCAGAAGTAGAACCAGTTGAACCAAAGCCAGAAATAGTTATGGAACCAGATGTGGAACCAGTAATTCCAGAAACAGATCTTACAGAGACCGAAAAACAACCAATCGAAGCAGAAGTAGAACCAGTTGAACCGAAGACAGAGATAGTCGAACCAGAAACAGAGCCAGTCGAAGAAGCCGAGGAAGAAGCGGTTGAAGCACCCGTTATGGAACCAGTTATACCTGAGATAGAACCAAAGACAGAACCGGAAGTTGAAGCCGAGGAAAAAGAAGTCGAAACAAACGTTGAACCAGAACTACTGGAGACAGGAAAACCGATTGAAATTGGCCAAGCAACTGAAATCGAGGAGCCACCAGAAAAACAACCGGTGGAATCGGAGTCACAACCAGCGGAAGTTGAGATGGAACAAATTGAAACAAAGGAAGAACAAACTGAACCAAAGGAAGAACTAGCAGGAATTGATGAGAAATGTAAGTTTTAGTAACAATTATTTATTTTATGATTCCTTTTGAGCTGTTTCGGTATGCTTCGATGCAAGTCAAATTTGTGGATGTTTTAAATCCACCATTCAATTATGTTTGCAATGTAGTCATGAAGTTTGTTCGTTTGTTCAACACAGATATGGGACATATACGCATGAGCAGACCTTTTTTGTGCACAGATCTTCGTTCAGGTTAATCGTGCATGGCAACAAAAATTGGTTCATTCTGTTTTATGTCGTGCAGTCTTATTTTGCTTGAATTAAGCATTGCTGTAAAGTTCAAGTTAAAGGTACAGTCCATCATTTGTAATTTGTGCATTTTGTTCGTTTGAAGAAACAAACATGCTCAAAATCTAAAGAAGGCTGCTGAACAACTAACCTG-GGCCCAATTTCATAGCACTGCTTAACAGTAAGCAGAAAAGTTGTGCTTACTATAGCAGAAAATCATGCTGCTGCTTAAGCACTACTTCATGCCGTGAATGTGCTTACAGTTGCGCGCGCATGGAGATTTCTATTGTTACGTCACTGATTTAAGCAAAGTTTTCTTCTGGGGTTAAGCGTGCTTTTGCTGTGCTTACTGCTTAACAGTCCCTATGAAATAGATGGAGGCTCGGTAAGCACAAAGTCGGCTGTTAAGCAGCGCTATGAAATTGGGCCCTGATGAAGTTTCAGCTCAGCTTCAGCTGTATAAATTATTTCGAGAAAAACAAAATTCTAGGATCTCCATTTCAAATCAAAAGTTGCTGAATCTTGTTTTGAATTGGGCCTGAATTCAGCGTCCGAGGCACGTTCTCATCACTGAGCCGATTGCAGCTACGCGATGTCGCGCACGAATTAAAGCAATCGAGGGCGCGTGTGCGTTGTGTCAACGCTTGCGCGCGCTTCGCCAAATAAAAAAAAAAGAACTAAACGGAAATTTCTTTAGACGCATGTCTTTAGACGCACCGTTGGAAAGGCCTACTTTCGTTGCTTTCTTCAAAATGACAGCATGCCAGAAAGAAAAATCTCTTATCTTTCAACGAAGTATGTTTTTTAACAATAGGCCTAATTACGTCAGTTACTTTTTGGATAGTTGCAAATAATGATGGACTGTAGCTTTAAGAAATTTGTGGAACTCAAGTTGTGCTTGGCAAGTTTTACTTTAGAATTCGAAAATGCATCGACACCAAAAAAAAGTGTAATTTTGGTCCTATAGTTGAAGTGTCAAACTAGAACAAATTTAATGATAAATTAAATAAATTTAAATAATTTATTCCTACCTTGCTTTGACAGTGATGAAGGAGCTGCGTGACCTTTTGGAATCTACGAAGATTGACCTTCCTGTTGACATCAATGATCCATACGACCTAGGTCTTCTTCTCAGACATTTACGTCACCATTCAAATCTTCTTGCTCGTATTGGAGACCCCGATGTCAAAAAGGAAGTCCTCAGCGCCATGAATGAAAAC

>SS29-B

GCACGACGTGCCGCAGAGGAAAAACCTCAAAAGGGAAAGAAGGGACGAAAAGGAAAGAAACGACGAAAGGGAAAGAAGGGACGAAAAGGAAAGAAGGGACGAAAAGGAAAGAAACGACGAAAGGGAAAGAAAAGACGAAAGGGAAAGAAAGGACGAAAGGGAAAGAAAGGACGAAAGGGAAAGAAAGGACGAAAGGGAAAGAAAGGACGAAAGGGAAAGAAAGGACGAAAGGGAAAGAAAGGACGAAAGGGAAAGAAAGGACGAAAGGGAAAGAAAGGAAAACCAATCCAACCTGAAGAAACACCAGCCATTCCGACGGAGATAAAAGCTGCAGAAATAGAAAAGGAACCAAAAACAGAAGTGGTTGTGGAACCAGTTATTCCGGAAGCAGATATTGCGGAGACCGAAATACAACAAATTAAAGCAGAAGTAGAACCAGTTGAAACAAAGCCAGAAATAGTTATGGAACCAGATGTGGAACCAGTTATTCCAGAAACGGAACTTACAGAGACCGGAAAAGAAGCGAAAGTAGAACAAGTTAAACCAGTGGAAGGGAAGCTAAACTTA------------------------------------------------------------------------------------------------------------------------------------------------------------------------------------------------------------------------------------------------------------------------------------------------------------------------------------------------------------------------------------------------------------------------------------------------------------------------------GGAAAAGGAAAGGGCAAAGGAAAGAAGGTTAGAAAGGGAAAGAAAGGACGAAAGGGAAAGAAAGGACGAAAGGGAAAGAAAGGACGGAAAGGAAAGAAGGGGCGAAAAGGAAAGAAAAGAAGAAAAGGAAAAAAAGGACGAAAGGGAAAGAAAGGACGAAAGGGAAAGAAAGGACGAAAGGGCAAGAAAGGACGAAAGGGAAAGAAAGGACGAAAGGGAAAGAAAGGACGAAAGGGAAAGAAAGGACGAAGGGGAAAGAAAGGA------------------CGAAAGGGCAAGAAAGGAAATCCAATCAAAACTGAAGAAACACCAGCCATTCTGACGGAGATAAAAGCTGCAGAATTAGAAAAGGAACCAAAAACAGAAATAGTTATGAAACCAGTTATTCCCGAAACGGAACTAACAGAGACCGGAAAAGAAGCAGAAGTAGAACAAGTTAAACCAGTGGAAAGGAAGCTAAAAATAGGAAAAGGAAAGGGCAAAGGAAAGAAGGTTAGAAAGGGAAAGAAAGGACGAAAGGGAAAGAAAGGACGAAAGGGAAAGAAAGGACGGAAAGGAAAGAAGGGGCGAAAAGGAAAGAAAAGAAGAAAAGGAAAGAAAAGACGAAAAGGAAAGAAACGACGAAAGGGAAAGAAAGGACGAAAGGGAAAAAAAGGACGAAAGGGAAAGAAAGGACGAAAGGGAAAAAAAGGACGAAAGGGAAAAAAAGGACGAAAGGGAAAGAAAGGACGAAAGGGAAAGAAAGGCAAACCAATCCAAACTGAAGAAACACCAGCCATTCCGACGGAGATAAAAGCTGCAGAAATAGAAAAGGAACCAAAAACAGAAGTGGTTGTGGAACCACTTATTCAGGAAAAAGATGTTGCGGAGACCGAAATACAACCCATCGAAGCAGAAGTAGAACCAGTTGAACCAAAGACAGAAATAGTTATGGAACCAGTTATTCCGGAAGCAGATATTGCGGAGACCGAAATACAACCCATCAAAGCAGAAGAAGAACCAGTT------------------------------------------------------------------------------------------------------------GAACCAAAGCCAGAAATAGTTATGGAACCAGATGAGGAACCAGTTATTCCGGAAGCAGATATTGCGGAGACCGAAATACAACCCATCAAAGCAGAAGAAGAACCAGTTGAACCAAAGCCAGAAATAGTTATGGAACCAGATGAGGAACCAGTTATTCCGGAAGCAGATATTGCGGAGACCGAAATACAACCCATCAAAGCAGAAGAAGAACCAGTTGAACCAAAGCCAGAAATAGTTATGGAACCAGATGAGGAACCAGTTATTCCGGAAGCAGATATTGCGGAGACCGAAATACAACCCATCAGAGCAGAAGAAGAACCAGTTGAACCAAAGCCAGAAATAGTTATGGAACCAGATGAGGAACCAGTCATTCCAGAAACAGAACTAACAGAGACAGACAAACAACCAATCGAAGCAGAAGTAGAACCAGTTGAACCAAAAACAGAAGTGGTTGTGGAACCAGTTATTCCGGAAGCAGATATTGCGGAGACCGAAATACAACCCATCGAAGCAGAAGTAGAACCAGTTGAACCAAAGCCAGAAATAGTTATGGAACCAGATGTGGAACCAGTAATTCCAGAAACAGATCTTACAGAGACCGAAAAACAACCAATCGAAGCAGAAGTAGAACCAGTTGAACCGAAGACAGAGATAGTCGAACCAGAAACAGAGCCAGTCGAAGAAGCCGAGGAAGAAGCGGTTGAAGCACCCGTTATGGAACCAGTTATACCTGAGATAGAACCAAAGACAGAACCGGAAGTTGAAGCCGAGGAAAAAGAAGTCGAAACAAACGTTGAACCAGAACTACTGGAGACAGGAAAACCGATTGAAATTGGCCAAGCAACTGAAATCGAGGAGCCACCAGAAAAACAACCGGTGGAATCGGAGTCACAACCAGCGGAAGTTGAGATGGAACAAATTGAAACAAAGGAAGAACAAACTGAACCAAAGGAAGAACTAGCAGGAATTGATGAGAAATGTAAGTTTTAGTAACAATTATTTATTTTATGATTCCTTTTGAGCTGTTTCGGTATGCTTCGATGCAAGTCAAATTTGTGGATGTTTTAAATCCATCATTCAATTATGTTTGCAATGTAGTCATGAAGTTTGTTCGTTTGTTCAACACAGATATGGGACATATACGCATGAGCAGACCTTTTTTGTGCACAGATCTTCGTTCAGGTTAATCGTGCATGGCAACAAAAATTGGTTCATTCCGTTTTATGTCGTGCAGTCTTATTTTGCTTGAATTAAGCATTGCTGTAAAGTTCAAGTTAAAGGTACAGTCCATCATTTGTAATTTGTGCATTTTGTTCGTTTGAAGAAACAAACATGCTCAAAATCTAAAGAAGGCTGCTGAACAACTAACCT---------------------------------------------------------------------------------------------------------------------------------------------------------------------------------------------------------------------------------------------------------------------------------------GATGAAGTTTCAGCTCAGCTTCAGCTGTATAAATTATTTCGAGAAAAACAAAATTCTAGGATCTCCATTTCAAATCAAAAGTTGCTGAATCTTGTTTTGAATTGGGCCTGAATTCAGCGTCCGAGGCACGTTCTCATCACTGAGCCGATTGCAGCTACGCGATGTCGCGCACGAATTAAAGCAATCGAGGGCGCGTGTGCGTTGTGTCAACGCTTGCGCGCGCTTCGCCAAATAAAAAAAAAAGAACTAAACGGAAATTTCTTTAGACGCATGTCTTTAGACGCACCGTTGGAAAGGCCTACTTTCGTTGCTTTCTTCAAAATGACAGCATGCCAGAAAGAAAAATCTCTTATCTTTCAACGAAGTATGTTTTTTAACAATAGGCCTAATTACGTCAGTTACTTTTTGGATAGTTGCAAATAATGATGGACTGTAGCTTTAAGAAATTTGTGGAACTCAAGTTGTGCTTGGCAAGTTTTACTTTAGAATTCGAAAATGCATCGACACCAAAAAAAAGTGTAATTTTGGTCCTATAGTTGAAGTGTCAAACTAGAACAAATTTAATGATAAATTAAATAAATTTAAATAATTTATTCCTACCTTGCTTTGACAGTGATGAAGGAGCTGCGTGACCTTTTGGAATCTACGAAGATTGACCTTCCTGTTGACATCAATGATCCATACGACCTAGGTCTTCTTCTCAGACATTTACGTCACCATTCAAATCTTCTTGCTCGTATTGGAGACCCCGATGTCAAAAAGGAAGTCCTCAGCGCCATGAATGAAAAC

>SS93-A

GCACGACGTGCCGCAGAGGAAAAACCTCAAAAGGGAAAGAAGGGACGAAAAGGAAAGAAACGACGAAAGGGAAAGAAGGGACGAAAAGGAAAGAAGGGACGAAAAGGAAAGAAACGACGAAAGGGAAAGAAAAGACGAAAGGGAAAGAAAGGACGAAAGGGAAAGAAAGGACGAAAGGGAAAGAAAGGACGAAAGGGAAAGAAAGGACGAAAGGGAAAGAAAGGACGAAAGGGAAAGAAAGGACGAAAGGGAAAGAAAGGACGAAAGGGAAAGAAAGGAAAACCAATCCAACCTGAAGAAACACCAGCCATTCCGACGGAGATAAAAGCTGCAGAAATAGAAAAGGAACCAAAAACAGAAGTGGTTGTGGAACCAGTTATTCCGGAAGCAGATATTGCGGAGACCGAAATACAACAAATTAAAGCAGAAGTAGAACCAGTTGAAACAAAGCCAGAAATAGTTATGGAACCAGATGTGGAACCAGTTATTCCAGAAACGGAACTTACAGAGACCGGAAAAGAAGCGAAAGTAGAACAAGTTAAACCAGTGGAAGGGAAGCTAAACTTA------------------------------------------------------------------------------------------------------------------------------------------------------------------------------------------------------------------------------------------------------------------------------------------------------------------------------------------------------------------------------------------------------------------------------------------------------------------------------GGAAAAGGAAAGGGCAAAGGAAAGAAGGTTAGAAAGGGAAAGAAAGGACGAAAGGGAAAGAAAGGACGAAAGGGAAAGAAAGGACGGAAAGGAAAGAAGGGGCGAAAAGGAAAGAAAAGAAGAAAAGGAAAAAAAGGACGAAAGGGAAAGAAAGGACGAAAGGGAAAGAAAGGACGAAAGGGCAAGAAAGGACGAAAGGGAAAGAAAGGACGAAAGGGAAAGAAAGGACGAAAGGGAAAGAAAGGACGAAGGGGAAAGAAAGGA------------------CGAAAGGGCAAGAAAGGAAATCCAATCAAAACTGAAGAAACACCAGCCATTCTGACGGAGATAAAAGCTGCAGAATTAGAAAAGGAACCAAAAACAGAAATAGTTATGAAACCAGTTATTCCCGAAACGGAACTAACTGAGACCGGAAAAGAAGCAGAAGTAGAACAAGTTAAACCAGTGGAAAGGAAGCTAAAAATAGGAAAAGGAAAGGGCAAAGGAAAGAAGGTTAGAAAGGGAAAGAAAGGACGAAAGGGAAAGAAAGGACGAAAGGGAAAGAAAGGACGGAAAGGAAAGAAGGGGCGAAAAGGAAAGAAAAGAAGAAAAGGAAAGAAAAGACGAAAAGGAAAGAAACGACGAAAGGGAAAGAAAGGACGAAAGGGAAAAAAAGGACGAAAGGGAAAGAAAGGACGAAAGGGAAAAAAAGGACGAAAGGGAAAAAAAGGACGAAAGGGAAAGAAAGGACGAAAGGGAAAGAAAGGCAAACCAATCCAAACTGAAGAAACACCAGCCATTCCGACGGAGATAAAAGCTACAGAAATAGAAAAGGAACCAAAAACAGAAGTGGTTGTGGAACCACTTATTCAGGAAAAAGATGTTGCGGAGACCGAAATACAACCCATCGAAGCAGAAGTAGAACCAGTTGAACCAAAGACAGAAATAGTTATGGAACCAGTTATTCCGGAAGCAGATATTGCGGAGACCGAAATACAACCCATCAAAGCAGAAGAAGAACCAGTT------------------------------------------------------------------------------------------------------------GAACCAAAGCCAGAAATAGTTATGGAACCAGATGAGGAACCAGTTATTCCGGAAGCAGATATTGCGGAGACCGAAATACAACCCATCAAAGCAGAAGAAGAACCAGTTGAACCAAAGCCAGAAATAGTTATGGAACCAGATGAGGAACCAGTTATTCCGGAAGCAGATATTGCGGAGACCGAAATACAACCCATCAAAGCAGAAGAAGAACCAGTTGAACCAAAGCCAGAAATAGTTATGGAACCAGATGAGGAACCAGTTATTCCGGAAGCAGATATTGCGGAGACCGAAATACAACCCATCAGAGCAGAAGAAGAACCAGTTGAACCAAAGCCAGAAATAGTTATGGAACCAGATGAGGAACCAGTCATTCCAGAAACAGAACTAACAGAGACAGACAAACAACCAATCGAAGCAGAAGTAGAACCAGTTGAACCAAAAACAGAAGTGGTTGTGGAACCAGTTATTCCGGAAGCAGATATTGCGGAGACCGAAATACAACCCATCGAAGCAGAAGTAGAACCAGTTGAACCAAAGCCAGAAATAGTTATGGAACCAGATGTGGAACCAGTAATTCCAGAAACAGATCTTACAGAGACCGAAAAACAACCAATCGAAGCAGAAGTAGAACCAGTTGAACCGAAGACAGAGATAGTCGAACCAGAAACAGAGCCAGTCGAAGAAGCCGAGGAAGAAGCGGTTGAAGCACCCGTTATGGAACCAGTTATACCTGAGATAGAACCAAAGACAGAACCGGAAGTTGAAGCCGAGGAAAAAGAAGTCGAAACAAACGTTGAACCAGAACTACTGGAGACAGGAAAACCGATTGAAATTGGCCAAGCAACTGAAATCGAGGAGCCACCAGAAAAACAACCGGTGGAATCGGAGTCACAACCAGCGGAAGTTGAGATGGAACAAATTGAAACAAAGGAAGAACAAACTGAACCAAAGGAAGAACTAGCAGGAATTGATGAGAAATGTAAGTTTTAGTAACAATTATTTATTTTATGATTCCTTTTGAGCTGTTTCGGTATGCTTCGATGCAAGTCAAATTTGTGGATGTTTTAAATCCATCATTCAATTATGTTTGCAATGTAGTCATGAAGTTTGTTCGTTTGTTCAACACAGATATGGGACATATACGCATGAGCAGACCTTTTTTGTGCACAGATCTTCGTTCAGGTTAATCGTGCATGGCAACAAAAATTGGTTCATTCTGTTTTATGTCGTGCAGTCTTATTTTGCTTGAATTAAGCATTGCTGTAAAGTTCAAGTTAAAGGTACAGTCCATCATTTGTAATTTGTGCATTTTGTTCGTTTGAAGAAACAAACATGCTCAAAATCTAAAGAAGGCTGCTGAACAACTAACCTG-GGCCCAATTTCATAGCACTGCTTAACAGTAAGCGGAAAAGTTGTGCTTACTATAGCAGAAAATCATGCTGCTGCTTAAGCACTACTTCATGCCGTGAATGTGCTTACAGTTGCGCGCGCATGGAGATTTCTATTGTTACGTCACTGATTTAAGCAAAGTTTTCTTCTGGGGTTAAGCGTGCTTTTGCTGTGCTTACTGCTTAACAGTCCCTATGAAATAGATGGAGGCTCGGTAAGCACAAAGTCGGCTGTTGAGCAGCGCTATGAAATTGGGCCCTGATGAAGTTTCAGCTCAGCTTCAGCTGTATAAATTATTTCGAGAAAAACAAAATTCTAGGATCTCCATTTCAAATCAAAAGTTGCTGAATCTTGTTTTGAATTGGGCCTGAATTCAGCGTCCGAGGCACGTTCTCATCACTGAGCCGATTGCAGCTACGCGATGTCGCGCACGAATTAAAGCAATCGAGGGCGCGTGTGCGTTGTGTCAACGCTTGCGCGCGCTTCGCCAAATAAAAAAAAAAGAACTAAACGGAAATTTCTTTAGACGCATGTCTTTAGACGCACCGTTGGAAAGGCCTACTTTCGTTGCTTTCTTCAAAATGACAGCATGCCAGAAAGAAAAATCTCTTATCTTTCAACGAAGTATGTTTTTTAACAATAGGCCTAATTACGTCAGTTACTTTTTGGATAGTTGCAAATAATGATGGACTGTAGCTTTAAGAAATTTGTGGAACTCAAGTTGTGCTTGGCAAGTTTTACTTTAGAATTCGAAAATGCATCGACACCAAAAAAAAGTGTAATTTTGGTCCTATAGTTGAAGTGTCAAACTAGAACAAATTTAATGATAAATTAAATAAATTTAAATAATTTATTCCTACCTTGCTTTGACAGTGATGAAGGAGCTGCGTGACCTTTTGGAATCTACGAAGATTGACCTTCCTGTTGACATCAATGATCCATACGACCTAGGTCTTCTTCTCAGACATTTACGTCACCATTCAAATCTTCTTGCTCGTATTGGAGACCCCGATGTCAAAAAGGAAGTCCTCAGCGCCATGAATGAAAAC

>SS88-A

GCACGACGTGCCGCAGAGGAAAAACCTCAAAAGGGAAAGAAGGGACGAAAAGGAAAGAAACGACGAAAGGGAAAGAAGGGACGAAAAGGAAAGAAGGGACGAAAAGGAAAGAAACGACGAAAGGGAAAGAAAAGACGAAAGGGAAAGAAAGGACGAAAGGGAAAGAAAGGACGAAAGGGAAAGAAAGGACGAAAGGGAAAGAAAGGACGAAAGGGAAAGAAAGGACGAAAGGGAAAGAAAGGACGAAAGGGAAAGAAAGGACGAAAGGGAAAGAAAGGAAAACCAATCCAACCTGAAGAAACACCAGCCATTCCGACGGAGATAAAAGCTGCAGAAATAGAAAAGGAACCAAAAACAGAAGTGGTTGTGGAACCAGTTATTCCGGAAGCAGATATTGCGGAGACCGAAATACAACAAATTAAAGCAGAAGTAGAACCAGTTGAAACAAAGCCAGAAATAGTTATGGAACCAGATGTGGAACCAGTTATTCCAGAAACGGAACTTACAGAGACCGGAAAAGAAGCGAAAGTAGAACAAGTTAAACCAGTGGAAGGGAAGCTAAACTTA------------------------------------------------------------------------------------------------------------------------------------------------------------------------------------------------------------------------------------------------------------------------------------------------------------------------------------------------------------------------------------------------------------------------------------------------------------------------------GGAAAAGGAAAGGGCAAAGGAAAGAAGGTTAGAAAGGGAAAGAAAGGACGAAAGGGAAAGAAAGGACGAAAGGGAAAGAAAGGACGGAAAGGAAAGAAGGGGCGAAAAGGAAAGAAAAGAAGAAAAGGAAAAAAAGGACGAAAGGGAAAGAAAGGACGAAAGGGAAAGAAAGGACGAAAGGGCAAGAAAGGACGAAAGGGAAAGAAAGGACGAAAGGGAAAGAAAGGACGAAAGGGAAAGAAAGGACGAAGGGGAAAGAAAGGA------------------CGAAAGGGCAAGAAAGGAAATCCAATCAAAACTGAAGAAACACCAGCCATTCTGACGGAGATAAAAGCTGCAGAATTAGAAAAGGAACCAAAAACAGAAATAGTTATGAAACCAGTTATTCCCGAAACGGAACTAACTGAGACCGGAAAAGAAGCAGAAGTAGAACAAGTTAAACCAGTGGAAAGGAAGCTAAAAATAGGAAAAGGAAAGGGCAAAGGAAAGAAGGTTAGAAAGGGAAAGAAAGGACGAAAGGGAAAGAAAGGACGAAAGGGAAAGAAAGGACGGAAAGGAAAGAAGGGGCGAAAAGGAAAGAAAAGAAGAAAAGGAAAGAAAAGACGAAAAGGAAAGAAACGACGAAAGGGAAAGAAAGGACGAAAGGGAAAAAAAGGACGAAAGGGAAAGAAAGGACGAAAGGGAAAAAAAGGACGAAAGGGAAAAAAAGGACGAAAGGGAAAGAAAGGACGAAAGGGAAAGAAAGGCAAACCAATCCAAACTGAAGAAACACCAGCCATTCCGACGGAGATAAAAGCTACAGAAATAGAAAAGGAACCAAAAACAGAAGTGGTTGTGGAACCACTTATTCAGGAAAAAGATGTTGCGGAGACCGAAATACAACCCATCGAAGCAGAAGTAGAACCAGTTGAACCAAAGACAGAAATAGTTATGGAACCAGTTATTCCGGAAGCAGATATTGCGGAGACCGAAATACAACCCATCAAAGCAGAAGAAGAACCAGTT------------------------------------------------------------------------------------------------------------GAACCAAAGCCAGAAATAGTTATGGAACCAGATGAGGAACCAGTTATTCCGGAAGCAGATATTGCGGAGACCGAAATACAACCCATCAAAGCAGAAGAAGAACCAGTTGAACCAAAGCCAGAAATAGTTATGGAACCAGATGAGGAACCAGTTATTCCGGAAGCAGATATTGCGGAGACCGAAATACAACCCATCAAAGCAGAAGAAGAACCAGTTGAACCAAAGCCAGAAATAGTTATGGAACCAGATGAGGAACCAGTTATTCCGGAAGCAGATATTGCGGAGACCGAAATACAACCCATCAGAGCAGAAGAAGAACCAGTTGAACCAAAGCCAGAAATAGTTATGGAACCAGATGAGGAACCAGTCATTCCAGAAACAGAACTAACAGAGACAGACAAACAACCAATCGAAGCAGAAGTAGAACCAGTTGAACCAAAAACAGAAGTGGTTGTGGAACCAGTTATTCCGGAAGCAGATATTGCGGAGACCGAAATACAACCCATCGAAGCAGAAGTAGAACCAGTTGAACCAAAGCCAGAAATAGTTATGGAACCAGATGTGGAACCAGTAATTCCAGAAACAGATCTTACAGAGACCGAAAAACAACCAATCGAAGCAGAAGTAGAACCAGTTGAACCGAAGACAGAGATAGTCGAACCAGAAACAGAGCCAGTCGAAGAAGCCGAGGAAGAAGCGGTTGAAGCACCCGTTATGGAACCAGTTATACCTGAGATAGAACCAAAGACAGAACCGGAAGTTGAAGCCGAGGAAAAAGAAGTCGAAACAAACGTTGAACCAGAACTACTGGAGACAGGAAAACCGATTGAAATTGGCCAAGCAACTGAAATCGAGGAGCCACCAGAAAAACAACCGGTGGAATCGGAGTCACAACCAGCGGAAGTTGAGATGGAACAAATTGAAACAAAGGAAGAACAAACTGAACCAAAGGAAGAACTAGCAGGAATTGATGAGAAATGTAAGTTTTANTAACAATTATTTATTTTATGATTCCTTTTGAGCTGTTTCGGTATGCTTCGATGCAAGTCAAATTTGTGGATGTTTTAAATCCATCATTCAATTATGTTTGCAATGTAGTCATGAAGTTTGTTCGTTTGTTCAACACAGATATGGGACATATACGCATGAGCAGACCTTTTTTGTGCACAGATCTTCGTTCAGGTTAATCGTGCATGGCAACAAAAATTGGTTCATTCTGTTTTATGTCGTGCAGTCTTATTTTGCTTGAATTAAGCATTGCTGTAAAGTTCAAGTTAAAGGTACAGTCCATCATTTGTAATTTGTGCATTTTGTTCGTTTGAAGAAACAAACATGCTCAAAATCTAAAGAAGGCTGCTGAACAACTAACCTG-GGCCCAATTTCATAGCACTGCTTAACAGTAAGCAGAAAAGTTGTGCTTACTATAGCAGAAAATCATGCTGCTGCTTAAGCACTACTTCATGCCGTGAATGTGCTTACAGTTGCGCGCGCATGGAGATTTCTATTGTTACGTCACTGATTTAAGCAAAGTTTTCTTCTGGGGTTAAGCGTGCTTTTGCTGTGCTTACTGCTTAACAGTCCCTATGAAATAGATGGAGGCTCGGTAAGCACAAAGTCGGCTGTTAAGCAGCGCTATGAAATTGGGCCCTGATGAAGTTTCAGCTCAGCTTCAGCTGTATAAATTATTTCGAGAAAAACAAAATTCTAGGATCTCCATTTCAAATCAAAAGTTGCTGAATCTTGTTTTGAATTGGGCCTGAATTCAGCGTCCGAGGCACGTTCTCATCACTGAGCCGATTGCAGCTACGCGATGTCGCGCACGAATTAAAGCAATCGAGGGCGCGTGTGCGTTGTGTCAACGCTTGCGCGCGCTTCGCCAAATAAAAAAAAAAGAACTAAACGGAAATTTCTTTAGACGCATGTCTTTAGACGCACCGTTGGAAAGGCCTACTTTCGTTGCTTTCTTCAAAATGACAGCATGCCAGAAAGAAAAATCTCTTATCTTTCAACGAAGTATGTTTTTTAACAATAGGCCTAATTACGTCAGTTACTTTTTGGATAGTTGCAAATAATGATGGACTGTAGCTTTAAGAAATTTGTGGAACTCAAGTTGTGCTTGGCAAGTTTTACTTTAGAATTCGAAAATGCATCGACACCAAAAAAAAGTGTAATTTTGGTCCTATAGTTGAAGTGTCAAACTAGAACAAATTTAATGATAAATTAAATAAATTTAAATAATTTATTCCTACCTTGCTTTGACAGTGATGAAGGAGCTGCGTGACCTTTTGGAATCTACGAAGATTGACCTTCCTGTTGACATCAATGATCCATACGACCTAGGTCTTCTTCTCAGACATTTACGTCACCATTCAAATCTTCTTGCTCGTATTGGAGACCCCGATGTCAAAAAGGAAGTCCTCAGCGCCATGAATGAAAAC

>SS88-B

GCACGACGTGCCGCAGAGGAAAAACCTCAAAAGGGAAAGAAGGGACGAAAAGGAAAGAAACGACGAAAGGGAAAGAAGGGACGAAAAGGAAAGAAGGGACGAAAAGGAAAGAAACGACGAAAGGGAAAGAAAAGACGAAAGGGAAAGAAAGGACGAAAGGGAAAGAAAGGACGAAAGGGAAAGAAAGGACGAAAGGGAAAGAAAGGACGAAAGGGAAAGAAAGGACGAAAGGGAAAGAAAGGACGAAAGGGAAAGAAAGGACGAAAGGGAAAGAAAGGAAAACCAATCCAACCTGAAGAAACACCAGCCATTCCGACGGAGATAAAAGCTGCAGAAATAGAAAAGGAACCAAAAACAGAAGTGGTTGTGGAACCAGTTATTCCGGAAGCAGATATTGCGGAGACCGAAATACAACAAATTAAAGCAGAAGTAGAACCAGTTGAAACAAAGCCAGAAATAGTTATGGAACCAGATGTGGAACCAGTTATTCCAGAAACGGAACTTACAGAGACCGGAAAAGAAGCGAAAGTAGAACAAGTTAAACCAGTGGAAGGGAAGCTAAACTTA------------------------------------------------------------------------------------------------------------------------------------------------------------------------------------------------------------------------------------------------------------------------------------------------------------------------------------------------------------------------------------------------------------------------------------------------------------------------------GGAAAAGGAAAGGGCAAAGGAAAGAAGGTTAGAAAGGGAAAGAAAGGACGAAAGGGAAAGAAAGGACGAAAGGGAAAGAAAGGACGGAAAGGAAAGAAGGGGCGAAAAGGAAAGAAAAGAAGAAAAGGAAAAAAAGGACGAAAGGGAAAGAAAGGACGAAAGGGAAAGAAAGGACGAAAGGGCAAGAAAGGACGAAAGGGAAAGAAAGGACGAAAGGGAAAGAAAGGACGAAAGGGAAAGAAAGGACGAAGGGGAAAGAAAGGA------------------CGAAAGGGCAAGAAAGGAAATCCAATCAAAACTGAAGAAACACCAGCCATTCTGACGGAGATAAAAGCTGCAGAATTAGAAAAGGAACCAAAAACAGAAATAGTTATGAAACCAGTTATTCCCGAAACGGAACTAACTGAGACCGGAAAAGAAGCAGAAGTAGAACAAGTTAAACCAGTGGAAAGGAAGCTAAAAATAGGAAAAGGAAAGGGCAAAGGAAAGAAGGTTAGAAAGGGAAAGAAAGGACGAAAGGGAAAGAAAGGACGAAAGGGAAAGAAAGGACGGAAAGGAAAGAAGGGGCGAAAAGGAAAGAAAAGAAGAAAAGGAAAGAAAAGACGAAAAGGAAAGAAACGACGAAAGGGAAAGAAAGGACGAAAGGGAAAAAAAGGACGAAAGGGAAAGAAAGGACGAAAGGGAAAAAAAGGACGAAAGGGAAAAAAAGGACGAAAGGGAAAGAAAGGACGAAAGGGAAAGAAAGGCAAACCAATCCAAACTGAAGAAACACCAGCCATTCCGACGGAGATAAAAGCTACAGAAATAGAAAAGGAACCAAAAACAGAAGTGGTTGTGGAACCACTTATTCAGGAAAAAGATGTTGCGGAGACCGAAATACAACCCATCGAAGCAGAAGTAGAACCAGTTGAACCAAAGACAGAAATAGTTATGGAACCAGTTATTCCGGAAGCAGATATTGCGGAGACCGAAATACAACCCATCAAAGCAGAAGAAGAACCAGTT------------------------------------------------------------------------------------------------------------GAACCAAAGCCAGAAATAGTTATGGAACCAGATGAGGAACCAGTTATTCCGGAAGCAGATATTGCGGAGACCGAAATACAACCCATCAAAGCAGAAGAAGAACCAGTTGAACCAAAGCCAGAAATAGTTATGGAACCAGATGAGGAACCAGTTATTCCGGAAGCAGATATTGCGGAGACCGAAATACAACCCATCAAAGCAGAAGAAGAACCAGTTGAACCAAAGCCAGAAATAGTTATGGAACCAGATGAGGAACCAGTTATTCCGGAAGCAGATATTGCGGAGACCGAAATACAACCCATCAGAGCAGAAGAAGAACCAGTTGAACCAAAGCCAGAAATAGTTATGGAACCAGATGAGGAACCAGTCATTCCAGAAACAGAACTAACAGAGACAGACAAACAACCAATCGAAGCAGAAGTAGAACCAGTTGAACCAAAAACAGAAGTGGTTGTGGAACCAGTTATTCCGGAAGCAGATATTGCGGAGACCGAAATACAACCCATCGAAGCAGAAGTAGAACCAGTTGAACCAAAGCCAGAAATAGTTATGGAACCAGATGTGGAACCAGTAATTCCAGAAACAGATCTTACAGAGACCGAAAAACAACCAATCGAAGCAGAAGTAGAACCAGTTGAACCGAAGACAGAGATAGTCGAACCAGAAACAGAGCCAGTCGAAGAAGCCGAGGAAGAAGCGGTTGAAGCACCCGTTATGGAACCAGTTATACCTGAGATAGAACCAAAGACAGAACCGGAAGTTGAAGCCGAGGAAAAAGAAGTCGAAACAAACGTTGAACCAGAACTACTGGAGACAGGAAAACCGATTGAAATTGGCCAAGCAACTGAAATCGAGGAGCCACCAGAAAAACAACCGGTGGAATCGGAGTCACAACCAGCGGAAGTTGAGATGGAACAAATTGAAACAAAGGAAGAACAAACTGAACCAAAGGAAGAACTAGCAGGAATTGATGAGAAATGTAAGTTTTAGTAACAATTATTTATCTTATGATTCCTTTTGAGCTGTTTCGGTATGCTTCGATGCAAGTCAAATTTGTGGATGTTTTAAATCCATCATTCAATTATGTTTGCAATGTAGTCATGAAGTTTGTTCGTTTGTTCAACACAGATATGGGACATATACGCATGAGCAGACCTTTTTTGTGCACAGATCTTCGTTCAGGTTAATCGTGCATGGCAACAAAAATTGGTTCATTCTGTTTTATGTCGTGCAGTCTTATTTTGCTTGAATTAAGCATTGCTGTAAAGTTCAAGTTAAAGGTACAGTCCATCATTTGTAATTTGTGCATTTTGTTCGTTTGAAGAAACAAACATGCTCAAAATCTAAAGAAGGCTGCTGAACAACTAACCT---------------------------------------------------------------------------------------------------------------------------------------------------------------------------------------------------------------------------------------------------------------------------------------GATGAAGTTTCAGCTCAGCTTCAGCTGTATAAATTATTTCGAGAAAAACAAAATTCTAGGATCTCCATTTCAAATCAAAAGTTGCTGAATCTTGTTTTGAATTGGGCCTGAATTCAGCGTCCGAGGCACGTTCTCATCACTGAGCCGATTGCAGCTACGCGATGTCGCGCACGAATTAAAGCAATCGAGGGCGCGTGTGCGTTGTGTCAACGCTTGCGCGCGCTTCGCCAAATAAAAAAAAAAGAACTAAACGGAAATTTCTTTAGACGCATGTCTTTAGACGCACCGTTGGAAAGGCCTACTTTCGTTGCTTTCTTCAAAATGACAGCATGCCAGAAAGAAAAATCTCTTATCTTTCAACGAAGTATGTTTTCTAACAATAGGCCTAATTACGTCAGTTACTTTTTGGATAGTTGCAAATAATGATGGACTGTAGCTTTAAGAAATTTGTGGAACTCAAGTTGTGCTTGGCAAGTTTTACTTTAGAATTCGAAAATGCATCGACACCAAAAAAAAGTGTAATTTTGGTCCTATAGTTGAAGTGTCAAACTAGAACAAATTTAATGATAAATTAAATAAATTTAAATAATTTATTCCTACCTTGCTTTGACAGTGATGAAGGAGCTGCGTGACCTTTTGGAATCTACGAAGATTGACCTTCCTGTTGACATCAATGATCCATACGACCTAGGTCTTCTTCTCAGACATTTACGTCACCATTCAAATCTTCTTGCTCGTATTGGAGACCCCGATGTCAAAAAGGAAGTCCTCAGCGCCATGAATGAAAAC

>SS26-A

GCACGACGTGCCGCAGAGGAAAAACCTCAAAAGGGAAAGAAGGGACGAAAAGGAAAGAAACGACGAAAGGGAAAGAAGGGACGAAAAGGAAAGAAGGGACGAAAAGGAAAGAAACGACGAAAGGGAAAGAAAAGACGAAAGGGAAAGAAAGGACGAAAGGGAAAGAAAGGACGAAAGGGAAAGAAAGGACGAAAGGGAAAGAAAGGACGAAAGGGAAAGAAAGGACGAAAGGGAAAGAAAGGACGAAAGGGAAAGAAAGGACGAAAGGGAAAGAAAGGAAAACCAATCCAACCTGAAGAAACACCAGCCATTCCGACGGAGATAAAAGCTGCAGAAATAGAAAAGGAACCAAAAACAGAAGTGGTTGTGGAACCAGTTATTCCGGAAGCAGATATTGCGGAGACCGAAATACAACAAATTAAAGCAGAAGTAGAACCAGTTGAAACAAAGCCAGAAATAGTTATGGAACCAGATGTGGAACCAGTTATTCCAGAAACGGAACTTACAGAGACCGGAAAAGAAGCGAAAGTAGAACAAGTTAAACAAGTGGAAGGGAAGCTAAACTTA------------------------------------------------------------------------------------------------------------------------------------------------------------------------------------------------------------------------------------------------------------------------------------------------------------------------------------------------------------------------------------------------------------------------------------------------------------------------------GGAAAAGGAAAGGGCAAAGGAAAGAAGGTTAGAAAGGGAAAGAAAGGACGAAAGGGAAAGAAACGACGAAAGGGAAAGAAAGGACGGAAAGGAAAGAAGGGGCGAAAAGGAAAGAAAAGAAGAAAAGGAAAAAAAGGACGAAAGGGAAAGAAAGGACGAAAGGGAAAGAAAGGACGAAAGGGCAAGAAAGGACGAAAGGGAAAGAAAGGACGAAAGGGAAAGAAAGGACGAAAGGGAAAGAAAGGACGAAGGGGAAAGAAAGGA------------------CGAAAGGGCAAGAAAGGAAATCCAATCAAAACTGAAGAAACACCAGCCATTCTGACGGAGATAAAAGCTGCAGAATTAGAAAAGGAACCAAAAACAGAAATAGTTATGAAACCAGTTATTCCCGAAACGGAACTAACAGAGACCGGAAAAGAAGCAGAAGTAGAACAAGTTAAACCAGTGGAAAGGAAGCTAAAAATAGGAAAAGGAAAGGGCAAAGGAAAGAAG------------------AGGAGAAAGGGAAAGAAAGGACGAAAGGGAAAGAAAGGACGGAAAGGAAAGAAGGGGCGT------------------AAAGGAAAGAAAAGACGAAAAGGAAAGAAACGACGAAAGGGAAAGAAAGGACGAAAGGGAAAAAAAGGACGAAAGGGAAAGAAAGGACGAAAGGGAAAAAAAGGACGAAAGGGAAAAAAAGGACGAAAGGGAAAGAAAGGACGAAAGGGAAAGAAAGGAAAACCAATCCAAACTGAAGAAACACCAGCCATTCCGACGGATATAAAAGCTGCAGAAATAGAAAAGGAACCAAAAACAGAAGTGGTTGTGGAACCACTTATTCAGGAAAAAGATGTTGCGGAGACCGAAATACAACCCATCGAAGCAGAAGTAGAACCAGTTGAACCAAAGACAGAAATAGTTATGGAACCAGTTATTCCGGAAGCAGATATTGCGGAGACCGAAATACAACCCATCAAAGCAGAAGAAGAACCAGTT------------------------------------------------------------------------------------------------------------GAACCAAAGCCAGAAATAGTTATGGAACCAGATGAGGAACCAGTTATTCCGGAAGCAGATATTGCGGAGACCGAAATACAACCCATCAAAGCAGAAGAAGAACCAGTTGAACCAAAGCCAGAAATAGTTATGGAACCAGATGAGGAACCAGTTATTCCGGAAGCAGATATTGCGGAGACCGAAATACAACCCATCAAAGCAGAAGAAGAACCAGTTGAACCAAAGCCAGAAATAGTTATGGAACCAGATGAGGAACCAGTTATTCCGGAAGCAGATATTGCGGAGACCGAAATACAACCCATCAGAGCAGAAGAAGAACCAGTTGAACCAAAGCCAGAAATAGTTATGGAACCAGATGAGGAACCAGTCATTCCAGAAACAGAACTAACAGAGACAGACAAACAACCAATCGAAGCAGAAGTAGAACCAGTTGAACCAAAAACAGAAGTGGTTGTGGAACCAGTTATTCCGGAAGCAGATATTGCGGAGACCGAAATACAACCCATCGAAGCAGAAGTAGAACCAGTTGAACCAAAGCCAGAAATAGTTATGGAACCAGATGTGGAACCAGTAATTCCAGAAACAGATCTTACAGAGACCGAAAAACAACCAATCGAAGCAGAAGTAGAACCAGTTGAACCGAAGACAGAGATAGTCGAACCAGAAACAGAGCCAGTCGAAGAAGCCGAGGAAGAAGCGGTTGAAGCACCCGTTATGGAACCAGTTATACCTGAGATAGAACCAAAGACAGAACCGGAAGTTGAAGCCGAGGAAAAAGAAGTCGAAACAAACGTTGAACCAGAACTACTGGAGACAGGAAAACCGATTGAAATTGGCCAAGCAACTGAAATCGAGGAGCCACCAGAAAAACAACCGGTGGAATCGGAGTCACAACCAGCGGAAGTTGAGATGGAACAAATTGAAACAAAGGAAGAACCAACTGAACCAAAGGAAGAACTAGCAGGAATTGATGAGAAATGTAAGTTTTAGTAACAATTATTTATTTTATGATTCCTTTTGAGCTGTTTCGGTATGCTTCGATGCAAGTCAAATTTGTGGATGTTTTAAATCCATCATTCAATT--GTTTGCAATGTAGTCATGAAGTTTGTTCGTTTGTTCAACACAGATATGGGACATATACGCATGAGCAGACCTTTTTTGTGCACAGATCTTCGTTCAGGTTAATCGTGCATGGCAACAAAAATTGGTTCATTCCGTTTTATGTCGTGCAGTCTTATTTTGCTTGAATTAAGCATTGCTGTAAAGTTCAAGTTAAAGGTACAGTCCATCATTTGTAATTTGTGCATTTTGTTCGTTTGAAGAAACAAACATGCTCAAAATCTAAAGAAGGCTGCTGAACAACTAACCT---------------------------------------------------------------------------------------------------------------------------------------------------------------------------------------------------------------------------------------------------------------------------------------GATGAAGTTTCAGCTCAGCTTCAGCTGTATAAATTATTTCGAGAAAAACAAAATTCTAGGATCTCCATTTCAAATCAAAAGTTGCTGAATCTTGTTTTGAATTGGGCCTGAATTCAGCGTCCGAGGCACGTTCTCATCACTGAGCCGATTGCAGCCACGCGATGTCGCGCACGAATTAAAGCAATCGAGGGCGCGTGTGCGTTGTGTCAACACTTGCGCGCGCTTCGCCAAATAAAAAAAAAAGAACTAAACGGAAATTTCTTTAGACGCATGTCTTTAGACGCACCGTTGGAAAGGCCTACTTTCGTTGCTTTCTTCAAAATGACAGCATGCCAGAAAGAAAAATCTCTTATCTTTCAACGAAGTATGTTTTTTAACAATAGGCCTAATTACGTCAGTTACTTTTTGGATAGTTGCAAATAATGATGGACTGTAGCTTTAAGAAATTTGTGGAACTAAAGTTGTGCTTGGCAAGTTTTACTTTAGAATTCGAAAATGCATCGACACC-AAAAAAAGTGTAATTTTGGTCCTATAGTTGAAGTGTCAAACTAGAACAAATTTAATGATAAATTAAATAAATTTAAATAATTTATTCCTACCTTGCTTTGACAGTGATGAAGGAGCTGCGTGACCTTTTGGAATCTACGAAGATTGACCTTCCTGTTGACATCAATGATCCATACGACCTAGGTCTTCTTCTCAGACATTTACGTCACCATTCAAATCTTCTTGCTCGTATTGGAGACCCCGATGTCAAAAAGGAAGTCCTCAGCGCCATGAATGAAAAC

>SS85-A

GCACGACGTGCCGCAGAGGAAAAACCTCAAAAGGGAAAGAAGGGACGAAAAGGAAAGAAACGACGAAAGGGAAAGAAGGGACGAAAAGGAAAGAAGGGACGAAAAGGAAAGAAACGACGAAAGGGAAAGAAAAGACGAAAGGGAAAGAAAGGACGAAAGGGAAAGAAAGGACGAAAGGGAAAGAAAGGACGAAAGGGAAAGAAAGGACGAAAGGGAAAGAAAGGACGAAAGGGAAAGAAAGGACGAAAGGGAAAGAAAGGACGAAAGGGAAAGAAAGGAAAACCAATCCAACCTGAAGAAACACCAGCCATTCCGACGGAGATAAAAGCTGCAGAAATAGAAAAGGAACCAAAAACAGAAGTGGTTGTGGAACCAGTTATTCCGGAAGCAGATATTGCGGAGACCGAAATACAACAAATTAAAGCAGAAGTAGAACCAGTTGAAACAAAGCCAGAAATAGTTATGGAACCAGATGTGGAACCAGTTATTCCAGAAACGGAACTTACAGAGACCGGAAAAGAAGCGAAAGTAGAACAAGTTAAACAAGTGGAAGGGAAGCTAAACTTA------------------------------------------------------------------------------------------------------------------------------------------------------------------------------------------------------------------------------------------------------------------------------------------------------------------------------------------------------------------------------------------------------------------------------------------------------------------------------GGAAAAGGAAAGGGCAAAGGAAAGAAGGTTAGAAAGGGAAAGAAAGGACGAAAGGGAAAGAAACGACGAAAGGGAAAGAAAGGACGGAAAGGAAAGAAGGGGCGAAAAGGAAAGAAAAGAAGAAAAGGAAAAAAAGGACGAAAGGGAAAGAAAGGACGAAAGGGAAAGAAAGGACGAAAGGGCAAGAAAGGACGAAAGGGAAAGAAAGGACGAAAGGGAAAGAAAGGACGAAAGGGAAAGAAAGGACGAAGGGGAAAGAAAGGA------------------CGAAAGGGCAAGAAAGGAAATCCAATCAAAACTGAAGAAACACCAGCCATTCTGACGGAGATAAAAGCTGCAGAATTAGAAAAGGAACCAAAAACAGAAATAGTTATGAAACCAGTTATTCCCGAAACGGAACTAACAGAGACCGGAAAAGAAGCAGAAGTAGAACAAGTTAAACCAGTGGAAAGGAAGCTAAAAATAGGAAAAGGAAAGGGCAAAGGAAAGAAG------------------AGGAGAAAGGGAAAGAAAGGACGAAAGGGAAAGAAAGGACGGAAAGGAAAGAAGGGGCGT------------------AAAGGAAAGAAAAGACGAAAAGGAAAGAAACGACGAAAGGGAAAGAAAGGACGAAAGGGAAAAAAAGGACGAAAGGGAAAGAAAGGACGAAAGGGAAAAAAAGGACGAAAGGGAAAAAAAGGACGAAAGGGAAAGAAAGGACGAAAGGGAAAGAAAGGAAAACCAATCCAAACTGAAGAAACACCAGCCATTCCGACGGATATAAAAGCTGCAGAAATAGAAAAGGAACCAAAAACAGAAGTGGTTGTGGAACCACTTATTCAGGAAAAAGATGTTGCGGAGACCGAAATACAACCCATCGAAGCAGAAGTAGAACCAGTTGAACCAAAGACAGAAATAGTTATGGAACCAGTTATTCCGGAAGCAGATATTGCGGAGACCGAAATACAACCCATCAAAGCAGAAGAAGAACCAGTT------------------------------------------------------------------------------------------------------------GAACCAAAGCCAGAAATAGTTATGGAACCAGATGAGGAACCAGTTATTCCGGAAGCAGATATTGCGGAGACCGAAATACAACCCATCAAAGCAGAAGAAGAACCAGTTGAACCAAAGCCAGAAATAGTTATGGAACCAGATGAGGAACCAGTTATTCCGGAAGCAGATATTGCGGAGACCGAAATACAACCCATCAAAGCAGAAGAAGAACCAGTTGAACCAAAGCCAGAAATAGTTATGGAACCAGATGAGGAACCAGTTATTCCGGAAGCAGATATTGCGGAGACCGAAATACAACCCATCAGAGCAGAAGAAGAACCAGTTGAACCAAAGCCAGAAATAGTTATGGAACCAGATGAGGAACCAGTCATTCCAGAAACAGAACTAACAGAGACAGACAAACAACCAATCGAAGCAGAAGTAGAACCAGTTGAACCAAAAACAGAAGTGGTTGTGGAACCAGTTATTCCGGAAGCAGATATTGCGGAGACCGAAATACAACCCATCGAAGCAGAAGTAGAACCAGTTGAACCAAAGCCAGAAATAGTTATGGAACCAGATGTGGAACCAGTAATTCCAGAAACAGATCTTACAGAGACCGAAAAACAACCAATCGAAGCAGAAGTAGAACCAGTTGAACCGAAGACAGAGATAGTCGAACCAGAAACAGAGCCAGTCGAAGAAGCCGAGGAAGAAGCGGTTGAAGCACCCGTTATGGAACCAGTTATACCTGAGATAGAACCAAAGACAGAACCGGAAGTTGAAGCCGAGGAAAAAGAAGTCGAAACAAACGTTGAACCAGAACTACTGGAGACAGGAAAACCGATTGAAATTGGCCAAGCAACTGAAATCGAGGAGCCACCAGAAAAACAACCGGTGGAATCGGAGTCACAACCAGCGGAAGTTGAGATGGAACAAATTGAAACAAAGGAAGAACCAACTGAACCAAAGGAAGAACTAGCAGGAATTGATGAGAAATGTAAGTTTTAGTAACAATTATTTATTTTATGATTCCTTTTGAGCTGTTTCGGTATGCTTCGATGCAAGTCAAATTTGTGGATGTTTTAAATCCATCATTCAATT--GTTTGCAATGTAGTCATGAAGTTTGTTCGTTTGTTCAACACAGATATGGGACATATACGCATGAGCAGACCTTTTTTGTGCACAGATCTTCGTTCAGGTTAATCGTGCATGGCAACAAAAATTGGTTCATTCCGTTTTATGTCGTGCAGTCTTATTTTGCTTGAATTAAGCATTGCTGTAAAGTTCAAGTTAAAGGTACAGTCCATCATTTGTAATTTGTGCATTTTGTTCGTTTGAAGAAACAAACATGCTCAAAATCTAAAGAAGGCTGCTGAACAACTAACCT---------------------------------------------------------------------------------------------------------------------------------------------------------------------------------------------------------------------------------------------------------------------------------------GATGAAGTTTCAGCTCAGCTTCAGCTGTATAAATTATTTCGAGAAAAACAAAATTCTAGGATCTCCATTTCAAATCAAAAGTTGCTGAATCTTGTTTTGAATTGGGCCTGAATTCAGCGTCCGAGGCACGTTCTCATCACTGAGCCGATTGCAGCTACGCGATGTCGCGCACGAATTAAAGCAATCGAGGGCGCGTGTGCGTTGTGTCAACGCTTGCGCGCGCTTCGCCAAATAAAAAAAAAAGAACTAAACGGAAATTTCTTTAGACGCATGTCTTTAGACGCACCGTTGGAAAGGCCTACTTTCGTTGCTTTCTTCAAAATGACAGCATGCCAGAAAGAAAAATCTCTTATCTTTCAACGAAGTATGTTTTTTAACAATANGCCTAATTACGTCAGTTACTTTTTGGATAGTTGCAAATAATGATGGACTGTAGCTTTAAGAAATTTGTGGAACTAAAGTTGTGCTTGGCAAGTTTTACTTTAGAATTCGAAAATGCATCGACACCAAAAAAA-GTGTAATTTTGGTCCTATAGTTGAAGTGTCAAACTAGAACAAATTTAATGATAAATTAAATAAATTTAAATAATTTATTCCTACCTTGCTTTGACAGTGATGAAGGAGCTGCGTGACCTTTTGGAATCTACGAAGATTGACCTTCCTGTTGACATCAATGATCCATACGACCTAGGTCTTCTTCTCAGACATTTACGTCACCATTCAAATCTTCTTGCTCGTATTGGAGACCCCGATGTCAAAAAGGAAGTCCTCAGCGCCATGAATGAAAAC

>SS80-A

GCACGACGTGCCGCAGAGGAAAAACCTCAAAAGGGAAAGAAGGGACGAAAAGGAAAGAAACGACGAAAGGGAAAGAAGGGACGAAAAGGAAAGAAGGGACGAAAAGGAAAGAAACGACGAAAGGGAAAGAAAAGACGAAAGGGAAAGAAAGGACGAAAGGGAAAGAAAGGACGAAAGGGAAAGAAAGGACGAAAGGGAAAGAAAGGACGAAAGGGAAAGAAAGGACGAAAGGGAAAGAAAGGACGAAAGGGAAAGAAAGGACGAAAGGGAAAGAAAGGAAAACCAATCCAACCTGAAGAAACACCAGCCATTCCGACGGAGATAAAAGCTGCAGAAATAGAAAAGGAACCAAAAACAGAAGTGGTTGTGGAACCAGTTATTCCGGAAGCAGATATTGCGGAGACCGAAATACAACAAATTAAAGCAGAAGTAGAACCAGTTGAAACAAAGCCAGAAATAGTTATGGAACCAGATGTGGAACCAGTTATTCCAGAAACGGAACTTACAGAGACCGGAAAAGAAGCGAAAGTAGAACAAGTTAAACAAGTGGAAGGGAAGCTAAACTTA------------------------------------------------------------------------------------------------------------------------------------------------------------------------------------------------------------------------------------------------------------------------------------------------------------------------------------------------------------------------------------------------------------------------------------------------------------------------------GGAAAAGGAAAGGGCAAAGGAAAGAAGGTTAGAAAGGGAAAGAAAGGACGAAAGGGAAAGAAACGACGAAAGGGAAAGAAAGGACGGAAAGGAAAGAAGGGGCGAAAAGGAAAGAAAAGAAGAAAAGGAAAAAAAGGACGAAAGGGAAAGAAAGGACGAAAGGGAAAGAAAGGACGAAAGGGCAAGAAAGGACGAAAGGGAAAGAAAGGACGAAAGGGAAAGAAAGGACGAAAGGGAAAGAAAGGACGAAGGGGAAAGAAAGGA------------------CGAAAGGGCAAGAAAGGAAATCCAATCAAAACTGAAGAAACACCAGCCATTCTGACGGAGATAAAAGCTGCAGAATTAGAAAAGGAACCAAAAACAGAAATAGTTATGAAACCAGTTATTCCCGAAACGGAACTAACAGAGACCGGAAAAGAAGCAGAAGTAGAACAAGTTAAACCAGTGGAAAGGAAGCTAAAAATAGGAAAAGGAAAGGGCAAAGGAAAGAAG------------------AGGAGAAAGGGAAAGAAAGGACGAAAGGGAAAGAAAGGACGGAAAGGAAAGAAGGGGCGT------------------AAAGGAAAGAAAAGACGAAAAGGAAAGAAACGACGAAAGGGAAAGAAAGGACGAAAGGGAAAAAAAGGACGAAAGGGAAAGAAAGGACGAAAGGGAAAAAAAGGACGAAAGGGAAAAAAAGGACGAAAGGGAAAGAAAGGACGAAAGGGAAAGAAAGGAAAACCAATCCAAACTGAAGAAACACCAGCCATTCCGACGGATATAAAAGCTGCAGAAATAGAAAAGGAACCAAAAACAGAAGTGGTTGTGGAACCACTTATTCAGGAAAAAGATGTTGCGGAGACCGAAATACAACCCATCGAAGCAGAAGTAGAACCAGTTGAACCAAAGACAGAAATAGTTATGGAACCAGTTATTCCGGAAGCAGATATTGCGGAGACCGAAATACAACCCATCAAAGCAGAAGAAGAACCAGTT------------------------------------------------------------------------------------------------------------GAACCAAAGCCAGAAATAGTTATGGAACCAGATGAGGAACCAGTTATTCCGGAAGCAGATATTGCGGAGACCGAAATACAACCCATCAAAGCAGAAGAAGAACCAGTTGAACCAAAGCCAGAAATAGTTATGGAACCAGATGAGGAACCAGTTATTCCGGAAGCAGATATTGCGGAGACCGAAATACAACCCATCAAAGCAGAAGAAGAACCAGTTGAACCAAAGCCAGAAATAGTTATGGAACCAGATGAGGAACCAGTTATTCCGGAAGCAGATATTGCGGAGACCGAAATACAACCCATCAGAGCAGAAGAAGAACCAGTTGAACCAAAGCCAGAAATAGTTATGGAACCAGATGAGGAACCAGTCATTCCAGAAACAGAACTAACAGAGACAGACAAACAACCAATCGAAGCAGAAGTAGAACCAGTTGAACCAAAAACAGAAGTGGTTGTGGAACCAGTTATTCCGGAAGCAGATATTGCGGAGACCGAAATACAACCCATCGAAGCAGAAGTAGAACCAGTTGAACCAAAGCCAGAAATAGTTATGGAACCAGATGTGGAACCAGTAATTCCAGAAACAGATCTTACAGAGACCGAAAAACAACCAATCGAAGCAGAAGTAGAACCAGTTGAACCGAAGACAGAGATAGTCGAACCAGAAACAGAGCCAGTCGAAGAAGCCGAGGAAGAAGCGGTTGAAGCACCCGTTATGGAACCAGTTATACCTGAGATAGAACCAAAGACAGAACCGGAAGTTGAAGCCGAGGAAAAAGAAGTCGAAACAAACGTTGAACCAGAACTACTGGAGACAGGAAAACCGATTGAAATTGGCCAAGCAACTGAAATCGAGGAGCCACCAGAAAAACAACCGGTGGAATCGGAGTCACAACCAGCGGAAGTTGAGATGGAACAAATTGAAACAAAGGAAGAACCAACTGAACCAAAGGAAGAACTAGCAGGAATTGATGAGAAATGTAAGTTTTAGTAACAATTATTTATTTTATGATTCCTTTTGAGCTGTTTCGGTATGCTTCGATGCAAGTCAAATTTGTGGATGTTTTAAATCCATCATTCAATT--GTTTGCAATGTAGTCATGAAGTTTGTTCGTTTGTTCAACACAGATATGGGACATATACGCATGAGCAGACCTTTTTTGTGCACAGATCTTCGTTCAGGTTAATCGTGCATGGCAACAAAAATTGGTTCATTCCGTTTTATGTCGTGCAGTCTTATTTTGCTTGAATTAAGCATTGCTGTAAAGTTCAAGTTAAAGGTACAGTCCATCATTTGTAATTTGTGCATTTTGTTCGTTTGAAGAAACAAACATGCTCAAAATCTAAAGAAGGCTGCTGAACAACTAACCT---------------------------------------------------------------------------------------------------------------------------------------------------------------------------------------------------------------------------------------------------------------------------------------GATGAAGTTTCAGCTCAGCTTCAGCTGTATAAATTATTTCGAGAAAAACAAAATTCTAGGATCTCCATTTCAAATCAAAAGTTGCTGAATCTTGTTTTGAATTGGGCCTGAATTCAGCGTCCGAGGCACGTTCTCATCACTGAGCCGATTGCAGCTACGCGATGTCGCGCACGAATTAAAGCAATCGAGGGCGCGTGTGCGTTGTGTCAACGCTTGCGCGCGCTTCGCCAAATAAAAAAAAAAGAACTAAACGGAAATTTCTTTAGACGCATGTCTTTAGACGCACCGTTGGAAAGGCCTACTTTCGTTGCTTTCTTCAAAATGACAGCATGCCAGAAAGAAAAATCTCTTATCTTTCAACGAAGTATGTTTTTTAACAATAGGCCTAATTACGTCAGTTACTTTTTGGATAGTTGCAAATAATGATGGACTGTAGCTTTAAGAAATTTGTGGAACTAAAGTTGTGCTTGGCAAGTTTTACTTTAGAATTCGAAAATGCATCGACACCAAAAAAAAGTGTAATTTTGGTCCTATAGTTGAAGTGTCAAACTAGAACAAATTTAATGATAAATTAAATAAATTTAAATAATTTATTCCTACCTTGCTTTGACAGTGATGAAGGAGCTGCGTGACCTTTTGGAATCTACGAAGATTGACCTTCCTGTTGACATCAATGATCCATACGACCTAGGTCTTCTTCTCAGACATTTACGTCACCATTCAAATCTTCTTGCTCGTATTGGAGACCCCGATGTCAAAAAGGAAGTCCTCAGCGCCATGAATGAAAAC

>SS12-B

GCACGACGTGCCGCAGAGGAAAAACCTCAAAAGGGAAAGAAGGGACGAAAAGGAAAGAAACGACGAAAGGGAAAGAAGGGACGAAAAGGAAAGAAGGGACGAAAAGGAAAGAAACGACGAAAGGGAAAGAAAAGACGAAAGGGAAAGAAAGGACGAAAGGGAAAGAAAGGACGAAAGGGAAAGAAAGGACGAAAGGGAAAGAAAGGACGAAAGGGAAAGAAAGGACGAAAGGGAAAGAAAGGACGAAAGGGAAAGAAAGGACGAAAGGGAAAGAAAGGAAAACCAATCCAACCTGAAGAAACACCAGCCATTCCGACGGAGATAAAAGCTGCAGAAATAGAAAAGGAACCAAAAACAGAAGTGGTTGTGGAACCAGTTATTCCGGAAGCAGATATTGCGGAGACCGAAATACAACAAATTAAAGCAGAAGTAGAACCAGTTGAAACAAAGCCAGAAATAGTTATGGAACCAGATGTGGAACCAGTTATTCCAGAAACGGAACTTACAGAGACCGGAAAAGAAGCGAAAGTAGAACAAGTTAAACCAGTGGAAGGGAAGCTAAACTTA------------------------------------------------------------------------------------------------------------------------------------------------------------------------------------------------------------------------------------------------------------------------------------------------------------------------------------------------------------------------------------------------------------------------------------------------------------------------------GGAAAAGGAAAGGGCAAAGGAAAGAAGGTTAGAAAGGGAAAGAAAGGACGAAAGGGAAAGAAAGGACGAAAGGGAAAGAAAGGACGGAAAGGAAAGAAGGGGCGAAAAGGAAAGAAAAGAAGAAAAGGAAAAAAAGGACGAAAGGGAAAGAAAGGACGAAAGGGAAAGAAAGGACGAAAGGGCAAGAAAGGACGAAAGGGAAAGAAAGGACGAAAGGGAAAGAAAGGACGAAAGGGAAAGAAAGGACGAAGGGGAAAGAAAGGA------------------CGAAAGGGCAAGAAAGGAAATCCAATCAAAACTGAAGAAACACCAGCCATTCTGACGGAGATAAAAGCTGCAGAATTAGAAAAGGAACCAAAAACAGAAATAGTTATGAAACCAGTTATTCCCGAAACGGAACTAACAGAGACCGGAAAAGAAGCAGAAGTAGAACAAGTTAAACCAGTGGAAAGGAAGCTAAAAATAGGAAAAGGAAAGGGCAAAGGAAAGAAG------------------AGGAGAAAGGGAAAGAAAGGACGAAAGGGAAAGAAAGGACGGAAAGGAAAGAAGGGGCGT------------------AAAGGAAAGAAAAGACGAAAAGGAAAGAAACGACGAAAGGGAAAGAAAGGACGAAAGGGAAAAAAAGGACGAAAGGGAAAGAAAGGACGAAAGGGAAAAAAAGGACGAAAGGGAAAAAAAGGACGAAAGGGAAAGAAAGGACGAAAGGGAAAGAAAGGAAAACCAATCCAAACTGAAGAAACACCAGCCATTCCGACGGAGATAAAAGCTGCAGAAATAGAAAAGGAACCAAAAACAGAAGTGGTTGTGGAACCACTTATTCAGGAAAAAGATGTTGCGGAGACCGAAATACAACCCATCGAAGCAGAAGTAGAACCAGTTGAACCAAAGACAGAAATAGTTATGGAACCAGTTATTCCGGAAGCAGATATTGCGGAGACCGAAATACAACCCATCAAAGCAGAAGAAGAACCAGTT------------------------------------------------------------------------------------------------------------GAACCAAAGCCAGAAATAGTTATGGAACCAGATGAGGAACCAGTTATTCCGGAAGCAGATATTGCGGAGACCGAAATACAACCCATCAAAGCAGAAGAAGAACCAGTTGAACCAAAGCCAGAAATAGTTATGGAACCAGATGAGGAACCAGTTATTCCGGAAGCAGATATTGCGGAGACCGAAATACAACCCATCAAAGCAGAAGAAGAACCAGTTGAACCAAAGCCAGAAATAGTTATGGAACCAGATGAGGAACCAGTTATTCCGGAAGCAGATATTGCGGAGACCGAAATACAACCCATCAGAGCAGAAGAAGAACCAGTTGAACCAAAGCCAGAAATAGTTATGGAACCAGATGAGGAACCAGTCATTCCAGAAACAGAACTAACAGAGACAGACAAACAACCAATCGAAGCAGAAGTAGAACCAGTTGAACCAAAAACAGAAGTGGTTGTGGAACCAGTTATTCCGGAAGCAGATATTGCGGAGACCGAAATACAACCCATCGAAGCAGAAGTAGAACCAGTTGAACCAAAGCCAGAAATAGTTATGGAACCAGATGTGGAACCAGTAATTCCAGAAACAGATCTTACAGAGACCGAAAAACAACCAATCGAAGCAGAAGTAGAACCAGTTGAACCGAAGACAGAGATAGTCGAACCAGAAACAGAGCCAGTCGAAGAAGCCGAGGAAGAAGCGGTTGAAGCACCCGTTATGGAACCAGTTATACCTGAGATAGAACCAAAGACAGAACCGGAAGTTGAAGCCGAGGAAAAAGAAGTCGAAACAAACGTTGAACCAGAACTACTGGAGACAGGAAAACCGATTGAAATTGGCCAAGCAACTGAAATCGAGGAGCCACCAGAAAAACAACCGGTGGAATCGGAGTCACAACCAGCGGAAGTTGAGATGGAACAAATTGAAACAAAGGAAGAACCAACTGAACCAAAGGAAGAACTAGCAGGAATTGATGAGAAATGTAAGTTTTAGTAACAATTATTTATTTTATGATTCCTTTTGAGCTGTTTCGGTATGCTTCGATGCAAGTCAAATTTGTGGATGTTTTAAATCCATCATTCAATTATGTTTGTAATGTAGTCATGAAGTTTGTTCGTTTGTTCAACACAGATATGGGACATATACGCATGAGCAGACCTTTTTTGTGCACAGATCTTCGTTCAGGTTAATCGTGCATGGCAACAAAAATTGGTTCATTCCGTTTTATGTCGTGCAGTCTTATTTTGCTTGAATTAAGCATTGCTGTAAAGTTCAAGTTAAAGGTACAGTCCATCATTTGTAATTTGTGCATTTTGTTCGTTTGAAGAAACAAACATGCTCAAAATCTAAAGAAGGCTGCTGAACAACTAACCT---------------------------------------------------------------------------------------------------------------------------------------------------------------------------------------------------------------------------------------------------------------------------------------GATGAAGTTTCAGCTCAGCTTCAGCTGTATAAATTATTTCGAGAAAAACAAAATTCTAGGATCTCCATTTCAAATAAAAAGTTGCTGAATCTTGTTTTGAATTGGGCCTGAATTCAGCGTCCGAGGCACGTTCTCATCACTGAGCCGATTGCAGCTACGCGATGTCGCGCACGAATTAAAGCAATCGAGGGCGCGTGTGCGTTGTGTCAACGCTTGCGCGCGCTTCGCCAAATAAAAAAAAAAGAACTAAACGGAAATTTCTTTAGACGCATGTCTTTAGACGCACCGTTGGAAAGGCCTACTTTCGTTGCTTTCTTCAAAATGACAGCATGCCAGAAAGAAAAATCTCTTATCTTTCAACGAAGTATGTTTTTTAACAATAGGCCTAATTACGTCAGTTACTTTTTGGATAGTTGCAAATAATGATGGACTGTAGCTTTAAGAAATTTGTGGAACTAAAGTTGTGCTTGGCAAGTTTTACTTTAGAATTCGAAAATGCATCGACACCAAAAAAAAGTGTAATTTTGGTCCTATAGTTGAAGTGTCAAACTAGAACAAATTTAATGATAAATTAAATAAATTTAAATAATTTATTCCTACCTTGCTTTGACAGTGATGAAGGAGCTGCGTGACCTTTTGGAATCTACGAAGATTGACCTTCCTGTTGACATCAATGATCCATACGACCTAGGTCTTCTTCTCAGACATTTACGTCACCATTCAAATCTTCTTGCTCGTATTGGAGACCCCGATGTCAAAAAGGAAGTCCTCAGCGCCATGAATGAAAAC

>SS89-A

GCACGACGTGCCGCAGAGGAAAAACCTCAAAAGGGAAAGAAGGGACGAAAAGGAAAGAAACGACGAAAGGGAAAGAAGGGACGAAAAGGAAAGAAGGGACGAAAAGGAAAGAAACGACGAAAGGGAAAGAAAAGACGAAAGGGAAAGAAAGGACGAAAGGGAAAGAAAGGACGAAAGGGAAAGAAAGGACGAAAGGGAAAGAAAGGACGAAAGGGAAAGAAAGGACGAAAGGGAAAGAAAGGACGAAAGGGAAAGAAAGGACGAAAGGGAAAGAAAGGAAAACCAATCCAACCTGAAGAAACACCAGCCATTCCGACGGAGATAAAAGCTGCAGAAATAGAAAAGGAACCAAAAACAGAAGTGGTTGTGGAACCAGTTATTCCGGAAGCAGATATTGCGGAGACCGAAATACAACAAATTAAAGCAGAAGTAGAACCAGTTGAAACAAAGCCAGAAATAGTTATGGAACCAGATGTGGAACCAGTTATTCCAGAAACGGAACTTACAGAGACCGGAAAAGAAGCGAAAGCAGAACAAGTTAAACCAGTGGAAGGGAAGCTAAACTTA------------------------------------------------------------------------------------------------------------------------------------------------------------------------------------------------------------------------------------------------------------------------------------------------------------------------------------------------------------------------------------------------------------------------------------------------------------------------------GGAAAAGGAAAGGGCAAAGGAAAGAAGGTTAGAAAGGGAAAGAAAGGACGAAAGGGAAAGAAAGGACGAAAGGGAAAGAAAGGACGGAAAGGAAAGAAGGGGCGAAAAGGAAAGAAAAGAAGAAAAGGAAAAAAAGGACGAAAGGGAAAGAAAGGACGAAAGGGAAAGAAAGGACGAAAGGGCAAGAAAGGACGAAAGGGAAAGAAAGGACGAAAGGGAAAGAAAGGACGAAAGGGAAAGAAAGGACGAAGGGGAAAGAAAGGA------------------CGAAAGGGCAAGAAAGGAAATCCAATCAAAACTGAAGAAACACCAGCCATTCTGACGGAGATAAAAGCTGCAGAATTAGAAAAGGAACCAAAAACAGAAATAGTTATGAAACCAGTTATTCCCGAAACGGAACTAACAGAGACCGGAAAAGAAGCAGAAGTAGAACAAGTTAAACCAGTGGAAAGGAAGCTAAAAATAGGAAAAGGAAAGGGCAAAGGAAAGAAG------------------AGGAGAAAGGGAAAGAAAGGACGAAAGGGAAAGAAAGGACGGAAAGGAAAGAAGGGGCGT------------------AAAGGAAAGAAAAGACGAAAAGGAAAGAAACGACGAAAGGGAAAGAAAGGACGAAAGGGAAAAAAAGGACGAAAGGGAAAGAAAGGACGAAAGGGAAAAAAAGGACGAAAGGGAAAAAAAGGACGAAAGGGAAAGAAAGGACGAAAGGGAAAGAAAGGAAAACCAATCCAAACTGAAGAAACACCAGCCATTCCGACGGAGATAAAAGCTGCAGAAATAGAAAAGGAACCAAAAACAGAAGTGGTTGTGGAACCACTTATTCAGGAAAAAGATGTTGCGGAGANCGAAATACAACCCATCGAAGCAGAAGTAGAACCAGTTGAACCAAAGACAGAAATAGTTATGGAACCAGTTATTCCGGAAGCAGATATTGCGGAGACCGAAATACAACCCATCAAAGCAGAAGAAGAACCAGTT------------------------------------------------------------------------------------------------------------GAACCAAAGCCAGAAATAGTTATGGAACCAGATGAGGAACCAGTTATTCCGGAAGCAGATATTGCGGAGACCGAAATACAACCCATCAAAGCAGAAGAAGAACCAGTTGAACCAAAGCCAGAAATAGTTATGGAACCAGATGAGGAACCAGTTATTCCGGAAGCAGATATTGCGGAGACCGAAATACAACCCATCAAAGCAGAAGAAGAACCAGTTGAACCAAAGCCAGAAATAGTTATGGAACCAGATGAGGAACCAGTTATTCCGGAAGCAGATATTGCGGAGACCGAAATACAACCCATCAGAGCAGAAGAAGAACCAGTTGAACCAAAGCCAGAAATAGTTATGGAACCAGATGAGGAACCAGTCATTCCAGAAACAGAACTAACAGAGACAGACAAACAACCAATCGAAGCAGAAGTAGAACCAGTTGAACCAAAAACAGAAGTGGTTGTGGAACCAGTTATTCCGGAAGCAGATATTGCGGAGACCGAAATACAACCCATCGAAGCAGAAGTAGAACCAGTTGAACCAAAGCCAGAAATAGTTATGGAACCAGATGTGGAACCAGTAATTCCAGAAACAGATCTTACAGAGACCGAAAAACAACCAATCGAAGCAGAAGTAGAACCAGTTGAACCGAAGACAGAGATAGTCGAACCAGAAACAGAGCCAGTCGAAGAAGCCGAGGAAGAAGCGGTTGAAGCACCCGTTATGGAACCAGTTATACCTGAGATAGAACCAAAGACAGAACCGGAAGTTGAAGCCGAGGAAAAAGAAGTCGAAACAAACGTTGAACCAGAACT---------------------------TGGCCAAGCAACTGAAATCGAGGAGCCACCAGAAAAACAACCGGTGGAATCGGAGTCACAACCAGCGGAAGTTGAGATGGAACAAATTGAAACAAAGGAAGAACCAACTGAACCAAAGGAAGAACTAGCAGGAATTGATGAGAAATGTAAGTTTTAGTAACAATTATTTATTTTATGATTCCTTTTGAGCTGTTTCGGTATGCTTCGATGCAAGTCAAATTTGTGGATGTTTTAAATCCATCATTCAATTATGTTTGCAATGTAGTCACGAAGTTTGTTCGTTTGTTCAACACAGATATGGGACATATACGCATGAGCAGACCTTTTTTGTGCACAGATCTTCGTTCAGGTTAATCGTGCATGGCAACAAAAATTGGTTCATTCCGTTTTATGTCGTGCAGTCTTATTTTGCTTGAATTAAGCATTGCTGTAAAGTTCAAGTTAAAGGTACAGTCCATCATTTGTAATTTGTGCATTTTGTTCGTTTGAAGAAACAAACATGCTCAAAATCTAAAGAAGGCTGCTGAACAACTAACCT---------------------------------------------------------------------------------------------------------------------------------------------------------------------------------------------------------------------------------------------------------------------------------------GATGAAGTTTCAGCTCAGCTTCAGCTGTATAAATTATTTCGAGAAAAACAAAATTCTAGGATCTCCATTTCAAATCAAAAGTTGCTGAATCTTGTTTTGAATTGGGCCTGAATTCAGCGTCCGAGGCACGTTCTCATCACTGAGCCGATTGCAGCTACGCGATGTCGCGCACGAATTAAAGCAATCGAGGGCGCGTGTGCGTTGTGTCAACGCTTGCGCGCGCTTCGCCAAATAAAAAAAAAAGAACTAAACGGAAATTTCTTTAGACGCATGTCTTTAGACGCACCGTTGGAAAGGCCTACTTTCGTTGCTTTCTTCAAAATGACAGCATGCCAGAAAGAAAAATCTCTTATCTTTCAACGAAGTATGTTTTTTAACAATAGGCCTAATTACGTCAGTTACTTTTTGGATAGTTGCAAATAATGATGGACTGTAGCTTTAAGAAATTTGTGGAACTAAAGTTGTGCTTGGCAAGTTTTACTTTAGAATTCGAAAATGCATCGACACCAAAAAAAAGTGTAATTTTGGTCCTATAGTTGAAGTGTCAAACTAGAACAAATTTAATGATAAATTAAATAAATTTAAATAATTTATTCCTACCTTGCTTTGACAGTGATGAAGGAGCTGCGTGACCTTTTGGAATCTACGAAGATTGACCTTCCTGTTGACATCA--------------------------------------------------------------------------------------------------------------------

>SS89-B

GCACGACGTGCCGCAGAGGAAAAACCTCAAAAGGGAAAGAAGGGACGAAAAGGAAAGAAACGACGAAAGGGAAAGAAGGGACGAAAAGGAAAGAAGGGACGAAAAGGAAAGAAACGACGAAAGGGAAAGAAAAGACGAAAGGGAAAGAAAGGACGAAAGGGAAAGAAAGGACGAAAGGGAAAGAAAGGACGAAAGGGAAAGAAAGGACGAAAGGGAAAGAAAGGACGAAAGGGAAAGAAAGGACGAAAGGGAAAGAAAGGACGAAAGGGAAAGAAAGGAAAACCAATCCAACCTGAAGAAACACCAGCCATTCCGACGGAGATAAAAGCTGCAGAAATAGAAAAGGAACCAAAAACAGAAGTGGTTGTGGAACCAGTTATTCCGGAAGCAGATATTGCGGAGACCGAAATACAACAAATTAAAGCAGAAGTAGAACCAGTTGAAACAAAGCCAGAAATAGTTATGGAACCAGATGTGGAACCAGTTATTCCAGAAACGGAACTTACAGAGACCGGAAAAGAAGCGAAAGTAGAACAAGTTAAACCAGTGGAAGGGAAGCTAAACTTA------------------------------------------------------------------------------------------------------------------------------------------------------------------------------------------------------------------------------------------------------------------------------------------------------------------------------------------------------------------------------------------------------------------------------------------------------------------------------GGAAAAGGAAAGGGCAAAGGAAAGAAGGTTAGAAAGGGAAAGAAAGGACGAAAGGGAAAGAAAGGACGAAAGGGAAAGAAAGGACGGAAAGGAAAGAAGGGGCGAAAAGGAAAGAAAAGAAGAAAAGGAAAGAAAAGACGAAAAGGAAAGAAAGGACGAAAGGGAAAGAAAGGACGAAAGGGCAAGAAAGGACGAAAGGGAAAGAAAGGACGAAAGGGAAAGAAAGGACGAAAGGGAAAGAAAGGACGAANGGGAAAGAAAGGA------------------CGAAAGGGCAAGAAAGGAAATCCAATCAAAACTGAAGAAACACCAGCCATTCTGACGGAGATAAAAGCTGCAGAATTAGAAAAGGAACCAAAAACAGAAATAGTTATGAAACCAGTTATTCCCGAAACGGAACTAACAGAGACCGGAAAAGAAGCAGAAGTAGAACAAGTTAAACCAGTGGAAAGGAAGCTAAAAATAGGAAAAGGAAAGGGCAAAGGAAAGAAG------------------AGGAGAAAGGGAAAGAAAGGACGAAAGGGAAAGAAAGGACGGAAAGGAAAGAAGGGGCGT------------------AAAGGAAAGAAAAGACGAAAAGGAAAGAAACGACGAAAGGGAAAGAAAGGACGAAAGGGAAAAAAAGGACGAAAGGGAAAGAAAGGACGAAAGGGAAAAAAAGGACGAAAGGGAAAAAAAGGACGAAAGGGAAAGAAAGGACGAAAGGGAAAGAAAGGAAAACCAATCCAAACTGAAGAAACACCAGCCATTCCGACGGAGATAAAAGCTGCAGAAATAGAAAAGGAACCAAAAACAGAAGTGGTTGTGGAACCACTTATTCAGGAAAAAGATGTTGCGGAGACCGAAATACAACCCATCGAAGCAGAAGTAGAACCAGTTGAACCAAAGACAGAAATAGTTATGGAACCAGTTATTCCGGAAGCAGATATTGCGGAGACCGAAATACAACCCATCAAAGCAGAAGAAGAACCAGTT------------------------------------------------------------------------------------------------------------GAACCAAAGCCAGAAATAGTTATGGAACCAGATGAGGAACCAGTTATTCCGGAAGCAGATATTGCGGAGACCGAAATACAACCCATCAAAGCAGAAGAAGAACCAGTTGAACCAAAGCCAGAAATAGTTATGGAACCAGATGAGGAACCAGTTATTCCGGAAGCAGATATTGCGGAGACCGAAATACAACCCATCAAAGCAGAAGAAGAACCAGTTGAACCAAAGCCAGAAATAGTTATGGAACCAGATGAGGAACCAGTTATTCCGGAAGCAGATATTGCGGAGACCGAAATACAACCCATCAGAGCAGAAGAAGAACCAGTTGAACCAAAGCCAGAAATAGTTATGGAACCAGATGAGGAACCAGTCATTCCAGAAACAGAACTAACAGAGACAGACAAACAACCAATCGAAGCAGAAGTAGAACCAGTTGAACCAAAAACAGAAGTGGTTGTGGAACCAGTTATTCCGGAAGCAGATATTGCGGAGACCGAAATACAACCCATCGAAGCAGAAGTAGAACCAGTTGAACCAAAGCCAGAAATAGTTATGGAACCAGATGTGGAACCAGTAATTCCAGAAACAGATCTTACAGAGACCGAAAAACAACCAATCGAAGCAGAAGTAGAACCAGTTGAACCGAAGACAGAGATAGTCGAACCAGAAACAGAGCCAGTCGAAGAAGCCGAGGAAGAAGCGGTTGAAGCACCCGTTATGGAACCAGTTATACCTGAGATAGAACCAAAGACAGAACCGGAAGTTGAAGCCGAGGAAAAAGAAGTCGAAACAAACGTTGAACCAGAACTACTGGAGACAGGAAAACCGATTGAAATTGGCCAAGCAACTGAAATCGAGGAGCCACCAGAAAAACAACCGGTGGAATCGGAGTCACAACCAGCGGAAGTTGAGATGGAACAAATTGAAACAAAGGAAGAACAAACTGAACCAAAGGAAGAACTAGCAGGAATTGATGAGAAATGTAAGTTTTAGTAACAATTATTTATTTTATGATTCCTTTTGAGCTGTTTCGGTATGCTTCGATGCAAGTCAAATTTGTGGATGTTTTAAATCCATCATTCAATTATGTTTGCAATGTAGTCATGAAGTTTGTTCGTTTGTTCAACACAGATATGGGACATATACGCATGAGCAGACCTTTTTTGTGCACAGATCTTCGTTCAGGTTAATCGTGCATGGCAACAAAAATTGGTTCATTCCGTTTTATGTCGTGCAGTCTTATTTTGCTTGAATTAAGCATTGCTGTAAAGTTCAAGTTAAAGGTACAGTCCATCATTTGTAATTTGTGCATTTTGTTCGTTTGAAGAAACAAACATGCTCAAAATCTAAAGAAGGCTGCTGAACAACTAACCT---------------------------------------------------------------------------------------------------------------------------------------------------------------------------------------------------------------------------------------------------------------------------------------GATGAAGTTTCAGCTCAGCTTCAGCTGTATAAATTATTTCGAGAAAAACAAAATTCTAGGATCTCCATTTCAAATCAAAAGTTGCTGAATCTTGTCTTGAATTGGGCCTGAATTCAGCGTCCGAGGCACGTTCTCATCACTGAGCCGATTGCAGCTACGCGATGTCGCGCACGAATTAAAGCAATCGAGGGCGCGTGTGCGTTGTGTCAACGCTTGCGCGCGCTTCGCCAAATAAAAAAAAAAGAACTAAACGGAAATTTCTTTAGACGCATGTCTTTAGACGCACCGTTGGAAAGGCCTACTTTCGTTGCTTTCTTCAAAATGACAGCATGCCAGAAAGAAAAATCTCTTATCTTTCAACGAAGTATGTTTTTTAACAATAGGCCTAATTACGTCAGTTACTTTTTGGATAGTTGCAAATAATGATGGACTGTAGCTTTAAGAAATTTGTGGAACTCAAGTTGTGCTTGGCAAGTTTTACTTTAGAATTCGAAAATGCATCGACACCAAAAAAAAGTGTAATTTTGGTCCTATAGTTGAAGTGTCAAACTAGAACAAATTTAATGATAAATTAAATAAATTTAAATAATTTATTCCTACCTTGCTTTGACAGTGATGAAGGAGCTGCGTGACCTTTTGGAATCTACGAAGATTGACCTTCCTGTTGACATCAATGATCCATACGACCTAGGTCTTCTTCTCAGACATTTACGTCACCATTCAAATCTTCTTGCTCGTATTGGAGACCCCGATGTCAAAAAGGAAGTCCTCAGCGCCATGAATGAAAAC

>SS76-B

GCACGACGTGCCGCAGAGGAAAAACCTCAAAAGGGAAAGAAGGGACGAAAAGGAAAGAAACGACGAAAGGGAAAGAAGGGACGAAAAGGAAAGAAGGGACGAAAAGGAAAGAAACGACGAAAGGGAAAGAAAAGACGAAAGGGAAAGAAAGGACGAAAGGGAAAGAAAGGACGAAAGGGAAAGAAAGGACGAAAGGGAAAGAAAGGACGAAAGGGAAAGAAAGGACGAAAGGGAAAGAAAGGACGAAAGGGAAAGAAAGGACGAAAGGGAAAGAAAGGAAAACCAATCCAACCTGAAGAAACACCAGCCATTCCGACGGAGATAAAAGCTGCAGAAATAGAAAAGGAACCAAAAACAGAAGTGGTTGTGGAACCAGTTATTCCGGAAGCAGATATTGCGGAGACCGAAATACAACAAATTAAAGCAGAAGTAGAACCAGTTGAAACAAAGCCAGAAATAGTTATGGAACCAGATGTGGAACCAGTTATTCCAGAAACGGAACTTACAGAGACCGGAAAAGAAGCGAAAGCAGAACAAGTTAAACCAGTGGAAGGGAAGCTAAACTTA------------------------------------------------------------------------------------------------------------------------------------------------------------------------------------------------------------------------------------------------------------------------------------------------------------------------------------------------------------------------------------------------------------------------------------------------------------------------------GGAAAAGGAAAGGGCAAAGGAAAGAAGGTTAGAAAGGGAAAGAAAGGACGAAAGGGAAAGAAAGGACGAAAGGGAAAGAAAGGACGGAAAGGAAAGAAGGGGCGAAAAGGAAAGAAAAGAAGAAAAGGAAAAAAAGGACGAAAGGGAAAGAAAGGACGAAAGGGAAAGAAAGGACGAAAGGGCAAGAAAGGACGAAAGGGAAAGAAAGGACGAAAGGGAAAGAAAGGACGAAAGGGAAAGAAAGGACGAAGGGGAAAGAAAGGA------------------CGAAAGGGCAAGAAAGGAAATCCAATCAAAACTGAAGAAACACCAGCCATTCTGACGGAGATAAAAGCTGCAGAATTAGAAAAGGAACCAAAAACAGAAATAGTTATGAAACCAGTTATTCCCGAAACGGAACTAACAGAGACCGGAAAAGAAGCAGAAGTAGAACAAGTTAAACCAGTGGAAAGGAAGCTAAAAATAGGAAAAGGAAAGGGCAAAGGAAAGAAG------------------AGGAGAAAGGGAAAGAAAGGACGAAAGGGAAAGAAAGGACGGAAAGGAAAGAAGGGGCGT------------------AAAGGAAAGAAAAGACGAAAAGGAAAGAAACGACGAAAGGGAAAGAAAGGACGAAAGGGAAAAAAAGGACGAAAGGGAAAGAAAGGACGAAAGGGAAAAAAAGGACGAAAGGGAAAAAAAGGACGAAAGGGAAAGAAAGGACGAAAGGGAAAGAAAGGAAAACCAATCCAAACTGAAGAAACACCAGCCATTCCGACGGAGATAAAAGCTGCAGAAATAGAAAAGGAACCAAAAACAGAAGTGGTTGTGGAACCACTTATTCAGGAAAAAGATGTTGCGGAGACCGAAATACAACCCATCGAAGCAGAAGTAGAACCAGTTGAACCAAAGACAGAAATAGTTATGGAACCAGTTATTCCGGAAGCAGATATTGCGGAGACCGAAATACAACCCATCAAAGCAGAA------CCAGTT------------------------------------------------------------------------------------------------------------GAACCAAAGCCAGAAATAGTTATGGAACCAGATGAGGAACCAGTTATTCCGGAAGCAGATATTGCGGAGACCGAAATACAACCCATCAAAGCAGAAGAAGAACCAGTTGAACCAAAGCCAGAAATAGTTATGGAACCAGATGAGGAACCAGTTATTCCGGAAGCAGATATTGCGGAGACCGAAATACAACCCATCAAAGCAGAAGAAGAACCAGTTGAACCAAAGCCAGAAATAGTTATGGAACCAGATGAGGAACCAGTTATTCCGGAAGCAGATATTGCGGAGACCGAAATACAACCCATCAGAGCAGAAGAAGAACCAGTTGAACCAAAGCCAGAAATAGTTATGGAACCAGATGAGGAACCAGTCATTCCAGAAACAGAACTAACAGAGACAGACAAACAACCAATCGAAGCAGAAGTAGAACCAGTTGAACCAAAAACAGAAGTGGTTGTGGAACCAGTTATTCCGGAAGCAGATATTGCGGAGACCGAAATACAACCCATCGAAGCAGAAGTAGAACCAGTTGAACCAAAGCCAGAAATAGTTATGGAACCAGATGTGGAACCAGTAATTCCAGAAACAGATCTTACAGAGACCGAAAAACAACCAATCGAAGCAGAAGTAGAACCAGTTGAACCGAAGACAGAGATAGTCGAACCAGAAACAGAGCCAGTCGAAGAAGCCGAGGAAGAAGCGGTTGAAGCACCCGTTATGGAACCAGTTATACCTGAGATAGAACCAAAGACAGAACCGGAAGTTGAAGCCGAGGAAAAAGAAGTCGAAACAAACGTTGAACCAGAACT---------------------------TGGCCAAGCAACTGAAATCGAGGAGCCACCAGAAAAACAACCGGTGGAATCGGAGTCACAACCAGCGGAAGTTGAGATGGAACAAATTGAAACAAAGGAAGAACCAACTGAACCAAAGGAAGAACTAGCAGGAATTGATGAGAAATGTAAGTTTTAGTAACAATTATTTATTTTATGATTCCTTTTGAGCTGTTTCGGTATGCTTCGATGCAAGTCAAACTTGTGGATGTTTTAAATCCATCATTCAATTATGTTTGCAATGTAGTCACGAAGTTTGTTCGTTTGTTCAACACAGATATGGGACATATACGCATGAGCAGACCTTTTTTGTGCACAGATCTTCGTTCAGGTTAATCGTGCATGGCAACAAAAATTGGCTCATTCCGTTTTATGTCGTGCAGTCTTATTTTGCTTGAATTAAGCATTGCTGTAAAGTTCAAGTTAAAGGTACAGTCCATCATTTGTAATTTGTGCATTTTGTTCGTTTGAAGAAACAAACATGCTCAAAATCTAAAGAAGGCTGCTGAACAACTAACCT---------------------------------------------------------------------------------------------------------------------------------------------------------------------------------------------------------------------------------------------------------------------------------------GATGAAGTTTCAGCTCAGCTTCAGCTGTATAAATTATTTCGAGAAAAACAAAATTCTAGGATCTCCATTTCAAATCAAAAGTTGCTGAATCTTGTTTTGAATTGGGCCTGAATTCAGCGTCCGAGGCACGTTCTCATCACTGAGCCGATTGCAGCTACGCGATGTCGCGCACGAATTAAAGCAATCGAGGGCGCGTGTGCGTTGTGTCAACGCTTGCGCGCGCTTCGCCAAATAAAAAAAAAAGAACTAAACGGAAATTTCTTTAGACGCATGTCTTTAGACGCACCGTTGGAAAGGCCTACTTTCGTTGCTTTCTTCAAAATGACAGCATGCCAGAAAGAAAAATCTCTTATCTTTCAACGAAGTATGNTTTTTAACAATAGGCCTAATTACGTCAGTTACTTTTTGGATAGTTGCAAATAATGATGGACTGTAGCTTTAAGAAATTTGTGGAACTAAAGTTGTGCTTGGCAAGTTTTACTTTAGAATTCGAAAATGCATCGACACCAAAAAAAAGTGTAATTTTGGTCCTATAGTTGAAGTGTCAAACTAGAACAAATTTAATGATAAATTAAATAAATTTAAATAATTTATTCCTACCTTGCTTTGACAGTGATGAAGGAGCTGCGTGACCTTTTGGAATCTACGAAGATTGACCTTCCTGTTGACATCAATGATCCATACGACCTAGGTCTTCTTCTCAGACATTTACGTCACCATTCAAATCTTCTTGCTCGTATTGGAGACCCCGATGTCAAAAAGGAAGTCCTCAGCGCCATGAATGAAAAC

>SS74-B

GCACGACGTGCCGCAGAGGAAAAACCTCAAAAGGGAAAGAAGGGACGAAAAGGAAAGAAACGACGAAAGGGAAAGAAGGGACGAAAAGGAAAGAAGGGACGAAAAGGAAAGAAACGACGAAAGGGAAAGAAAAGACGAAAGGGAAAGAAAGGACGAAAGGGAAAGAAAGGACGAAAGGGAAAGAAAGGACGAAAGGGAAAGAAAGGACGAAAGGGAAAGAAAGGACGAAAGGGAAAGAAAGGACGAAAGGGAAAGAAAGGACGAAAGGGAAAGAAAGGAAAACCAATCCAACCTGAAGAAACACCAGCCATTCCGACGGAGATAAAAGCTGCAGAAATAGAAAAGGAACCAAAAACAGAAGTGGTTGTGGAACCAGTTATTCCGGAAGCAGATATTGCGGAGACCGAAATACAACAAATTAAAGCAGAAGTAGAACCAGTTGAAACAAAGCCAGAAATAGTTATGGAACCAGATGTGGAACCAGTTATTCCAGAAACGGAACTTACAGAGACCGGAAAAGAAGCGAAAGCAGAACAAGTTAAACCAGTGGAAGGGAAGCTAAACTTA------------------------------------------------------------------------------------------------------------------------------------------------------------------------------------------------------------------------------------------------------------------------------------------------------------------------------------------------------------------------------------------------------------------------------------------------------------------------------GGAAAAGGAAAGGGCAAAGGAAAGAAGGTTAGAAAGGGAAAGAAAGGACGAAAGGGAAAGAAAGGACGAAAGGGAAAGAAAGGACGGAAAGGAAAGAAGGGGCGAAAAGGAAAGAAAAGAAGAAAAGGAAAAAAAGGACGAAAGGGAAAGAAAGGACGAAAGGGAAAGAAAGGACGAAAGGGCAAGAAAGGACGAAAGGGAAAGAAAGGACGAAAGGGAAAGAAAGGACGAAAGGGAAAGAAAGGACGAAGGGGAAAGAAAGGA------------------CGAAAGGGCAAGAAAGGAAATCCAATCAAAACTGAAGAAACACCAGCCATTCTGACGGAGATAAAAGCTGCAGAATTAGAAAAGGAACCAAAAACAGAAATAGTTATGAAACCAGTTATTCCCGAAACGGAACTAACAGAGACCGGAAAAGAAGCAGAAGTAGAACAAGTTAAACCAGTGGAAAGGAAGCTAAAAATAGGAAAAGGAAAGGGCAAAGGAAAGAAG------------------AGGAGAAAGGGAAAGAAAGGACGAAAGGGAAAGAAAGGACGGAAAGGAAAGAAGGGGCGT------------------AAAGGAAAGAAAAGACGAAAAGGAAAGAAACGACGAAAGGGAAAGAAAGGACGAAAGGGAAAAAAAGGACGAAAGGGAAAGAAAGGACGAAAGGGAAAAAAAGGACGAAAGGGAAAAAAAGGACGAAAGGGAAAGAAAGGACGAAAGGGAAAGAAAGGAAAACCAATCCAAACTGAAGAAACACCAGCCATTCCGACGGAGATAAAAGCTGCAGAAATAGAAAAGGAACCAAAAACAGAAGTGGTTGTGGAACCACTTATTCAGGAAAAAGATGTTGCGGAGACCGAAATACAACCCATCGAAGCAGAAGTAGAACCAGTTGAACCAAAGACAGAAATAGTTATGGAACCAGTTATTCCGGAAGCAGATATTGCGGAGACCGAAATACAACCCATCAAAGCAGAAGAAGAACCAGTT------------------------------------------------------------------------------------------------------------GAACCAAAGCCAGAAATAGTTATGGAACCAGATGAGGAACCAGTTATTCCGGAAGCAGATATTGCGGAGACCGAAATACAACCCATCAAAGCAGAAGAAGAACCAGTTGAACCAAAGCCAGAAATAGTTATGGAACCAGATGAGGAACCAGTTATTCCGGAAGCAGATATTGCGGAGACCGAAATACAACCCATCAAAGCAGAAGAAGAACCAGTTGAACCAAAGCCAGAAATAGTTATGGAACCAGATGAGGAACCAGTTATTCCGGAAGCAGATATTGCGGAGACCGAAATACAACCCATCAGAGCAGAAGAAGAACCAGTTGAACCAAAGCCAGAAATAGTTATGGAACCAGATGAGGAACCAGTCATTCCAGAAACAGAACTAACAGAGACAGACAAACAACCAATCGAAGCAGAAGTAGAACCAGTTGAACCAAAAACAGAAGTGGTTGTGGAACCAGTTATTCCGGAAGCAGATATTGCGGAGACCGAAATACAACCCATCGAAGCAGAAGTAGAACCAGTTGAACCAAAGCCAGAAATAGTTATGGAACCAGATGTGGAACCAGTAATTCCAGAAACAGATCTTACAGAGACCGAAAAACAACCAATCGAAGCAGAAGTAGAACCAGTTGAACCGAAGACAGAGATAGTCGAACCAGAAACAGAGCCAGTCGAAGAAGCCGAGGAAGAAGCGGTTGAAGCACCCGTTATGGAACCAGTTATACCTGAGATAGAACCAAAGACAGAACCGGAAGTTGAAGCCGAGGAAAAAGAAGTCGAAACAAACGTTGAACCAGAACT---------------------------TGGCCAAGCAACTGAAATCGAGGAGCCACCAGAAAAACAACCGGTGGAATCGGAGTCACAACCAGCGGAAGTTGAGATGGAACAAATTGAAACAAAGGAAGAACCAACTGAACCAAAGGAAGAACTAGCAGGAATTGATGAGAAATGTAAGTTTTAGTAACAATTATTTATTTTATGATTCCTTTTGAGCTGTTTCGGTATGCTTCGATGCAAGTCAAATTTGTGGATGTTTTAAATCCATCATTCAATTATGTTTGCAATGTAGTCACGAAGTTTGTTCGTTTGTTCAACACAGATATGGGACATATACGCATGAGCAGACCTTTTTTGTGCACAGATCTTCGTTCAGGTTAATCGTGCATGGCAACAAAAATTGGTTCATTCCGTTTTATGTCGTGCAGTCTTATTTTGCTTGAATTAAGCATTGCTGTAAAGTTCAAGTTAAAGGTACAGTCCATCATTTGTAATTTGTGCATTTTGTTCGTTTGAAGAAACAAACATGCTCAAAATCTAAAGAAGGCTGCTGAACAACTAACCT---------------------------------------------------------------------------------------------------------------------------------------------------------------------------------------------------------------------------------------------------------------------------------------GATGAAGTTTCAGCTCAGCTTCAGCTGTATAAATTATTTCGAGAAAAACAAAATTCTAGGATCTCCATTTCAAATCAAAAGTTGCTGAATCTTGTTTTGAATTGGGCCTGAATTCAGCGTCCGAGGCACGTTCTCATCACTGAGCCGATTGCAGCTACGCGATGTCGCGCACGAATTAAAGCAATCGAGGGCGCGTGTGCGTTGTGTCAACGCTTGCGCGCGCTTCGCCAAATAAAAAAAAAAGAACTAAACGGAAATTTCTTTAGACGCATGTCTTTAGACGCACCGTTGGAAAGGCCTACTTTCGTTGCTTTCTTCAAAATGACAGCATGCCAGAAAGAAAAATCTCTTATCTTTCAACGAAGTATGTTTTTTAACAATAGGCCTAATTACGTCAGTTACTTTTTGGATAGTTGCAAATAATGATGGACTGTAGCTTTAAGAAATTTGTGGAACTAAAGTTGTGCTTGGCAAGTTTTACTTTAGAATTCGAAAATGCATCGACACCAAAAAAAAGTGTAATTTTGGTCCTATAGTTGAAGTGTCAAACTAGAACAAATTTAATGATAAATTAAATAAATTTAAATAATTTATTCCTACCTTGCTTTGACAGTGATGAAGGAGCTGCGTGACCTTTTGGAATCTACGAAGATTGACCTTCCTGTTGACATCAATGATCCATACGACCTAGGTCTTCTTCTCAGACATTTACGTCACCATTCAAATCTTCTTGCTCGTATTGGAGACCCCGATGTCAAAAAGGAAGTCCTCAGCGCCATGAATGAAAAC

>SS17-A

GCACGACGTGCCGCAGAGGAAAAACCTCAAAAGGGAAAGAAGGGACGAAAAGGAAAGAAACGACGAAAGGGAAAGAAGGGACGAAAAGGAAAGAAGGGACGAAAAGGAAAGAAACGACGAAAGGGAAAGAAAAGACGAAAGGGAAAGAAAGGACGAAAGGGAAAGAAAGGACGAAAGGGAAAGAAAGGACGAAAGGGAAAGAAAGGACGAAAGGGAAAGAAAGGACGAAAGGGAAAGAAAGGACGAAAGGGAAAGAAAGGACGAAAGGGAAAGAAAGGAAAACCAATCCAACCTGAAGAAACACCAGCCATTCCGACGGAGATAAAAGCTGCAGAAATAGAAAAGGAACCAAAAACAGAAGTGGTTGTGGAACCAGTTATTCCGGAAGCAGATATTGCGGAGACCGAAATACAACAAATTAAAGCAGAAGTAGAACCAGTTGAAACAAAGCCAGAAATAGTTATGGAACCAGATGTGGAACCAGTTATTCCAGAAACGGAACTTACAGAGACCGGAAAAGAAGCGAAAGTAGAACAAGTTAAACCAGTGGAAGGGAAGCTAAACTTA------------------------------------------------------------------------------------------------------------------------------------------------------------------------------------------------------------------------------------------------------------------------------------------------------------------------------------------------------------------------------------------------------------------------------------------------------------------------------GGAAAAGGAAAGGGCAAAGGAAAGAAGGTTAGAAAGGGAAAGAAAGGACGAAAGGGAAAGAAAGGACGAAAGGGAAAGAAAGGACGGAAAGGAAAGAAGGGGCGAAAAGGAAAGAAGAGAAGAAAAGGAAAAAAAGGACGAAAGGGAAAGAAAGGACGAAAGGGAAAGAAAGGACGAAAGGGCAAGAAAGGACGAAAGGGAAAGAAAGGACGAAAGGGAAAGAAAGGACGAAAGGGAAAGAAAGGACGAAGGGGAAAGAAAGGA------------------CGAAAGGGCAAGAAAGGAAATCCAATCAAAACTGAAGAAACACCAGCCATTCTGACGGAGATAAAAGCTGCAGAATTAGAAAAGGAACCAAAAACAGAAATAGTTATGAAACCAGTTATTCCCGAAACGGAACTAACAGAGACCGGAAAAGAAGCAGAAGTAGAACAAGTTAAACCAGTGGAAAGGAAGCTAAAAATAGGAAAAGGAAAGGGCAAAGGAAAGAAGGTTAGAAAGGGAAAGAAAGGACGAAAGGGAAAGAAAGGACGAAAGGGAAAGAAAGGACGGAAAGGAAAGAAGGGGCGAAAAGGAAAGAAAAGAAGAAAAGGAAAGAAAAGACGAAAAGGAAAGAAACGACGAAAGGGAAAGAAAGGACGAAAGGGAAAAAAAGGACGAAAGGGAAAGAAAGGACGAAAGGGAAAAAAAGGACGAAAGGGAAAAAAAGGACGAAAGGGAAAGAAAGGACGAAAGGGAAAGAAAGGCAAACCAATCCAAACTGAAGAAACACCAGCCATTCCGACGGAGATAAAAGCTGCAGAAATAGAAAAGGAACCAAAAACAGAAGTGGTTGTGGAACCACTTATTCAGGAAAAAGATGTTGCGGAGACCGAAATACAACCCATCGAAGCAGAAGTAGAACCAGTTGAACCAAAGACAGAAATAGTTATGGAACCAGTTATTCCGGAAGCAGATATTGCGGAGACCGAAATACAACCCATCAAAGCAGAAGAAGAACCAGTT------------------------------------------------------------------------------------------------------------GAACCAAAGCCAGAAATAGTTATGGAACCAGATGAGGAACCAGTTATTCCGGAAGCAGATATTGCGGAGACCGAAATACAACCCATCAAAGCAGAAGAAGAACCAGTTGAACCAAAGCCAGAAATAGTTATGGAACCAGATGAGGAACCAGTTATTCCGGAAGCAGATATTGCGGAGACCGAAATACAACCCATCAAAGCAGAAGAAGAACCAGTTGAACCAAAGCCAGAAATAGTTATGGAACCAGATGAGGAACCAGTTATTCCGGAAGCAGATATTGCGGAGACCGAAATACAACCCATCAGAGCAGAAGAAGAACCAGTTGAACCAAAGCCAGAAATAGTTATGGAACCAGATGAGGAACCAGTCATTCCAGAAACAGAACTAACAGAGACAGACAAACAACCAATCGAAGCAGAAGTAGAACCAGTTGAACCAAAAACAGAAGTGGTTGTGGAACCAGTTATTCCGGAAGCAGATATTGCGGAGACCGAAATACAACCCATCGAAGCAGAAGTAGAACCAGTTGAACCAAAGCCAGAAATAGTTATGGAACCAGATGTGGAACCAGTAATTCCAGAAACAGATCTTACAGAGACCGAAAAACAACCAATCGAAGCAGAAGTAGAACCAGTTGAACCGAAGACAGAGATAGTCGAACCAGAAACAGAGCCAGTCGAAGAAGCCGAGGAAGAAGCGGTTGAAGCACCCGTTATGGAACCAGTTATACCTGAGATAGAACCAAAGACAGAACCGGAAGTTGAAGCCGAGGAAAAAGAAGTCGAAACAAACGTTGAACCAGAACTACTGGAGACAGGAAAACCGATTGAAATTGGCCAAGCAACTGAAATCGAGGAGCCACCAGAAAAACAACCGGTGGAATCGGAGTCACAACCAGCGGAAGTTGAGATGGAACAAATTGAAACAAAGGAAGAACAAACTGAACCAAAGGAAGAACTAGCAGGAATTGATGAGAAATGTAAGTTTTAGTAACAATTATTTATTTTATGATTCCTTTTGAGCTGTTTCGGTATGCTTCGATGCAAGTCAAATTTGTGGATGTTTTAAATCCATCATTCAATTATGTTTGCAATGTAGTCATGAAGTTTGTTCGTTTGTTCAACACAGATATGGGACATATACGCATGAGCAGACCTTTTTTGTGCACAGATCTTCGTTCAGGTTAATCGTGCATGGCAACAAAAATTGGTTCATTCCGTTTTATGTCGTGCAGTCTTATTTTGCTTGAATTAAGCATTGCTGTAAAGTTCGAGTTAAAGGTACAGTCCATCATTTGTAATTTGTGCATTTTGTTCGTTTGAAGAAACAAACATGCTCAAAATCTAAAGAAGGCTGCTGAACAACTAACCT---------------------------------------------------------------------------------------------------------------------------------------------------------------------------------------------------------------------------------------------------------------------------------------GATGAAGTTTCAGCTCAGCTTCAGCTGTATAAATTATTTCGAGAAAAACAAAATTCTAGGATCTCCATTTCAAATCAAAAGTTGCTGAATCTTGTTTTGAATTGGGCCTGAATTCAGCGTCCGAGGCACGTTCTCATCACTGAGCCGATTGCAGCTACGCGATGTCGCGCACGAATTAAAGCAATCGAAGGCGCGTGTGCGTTGTGTCAACGCTTGCGCGCGCTTCGCCAAATAAAAAAAAAAGAACTAAACGGAAATTTCTTTAGACGCATGTCTTTAGACGTACCGTTGGAAAGGCCTACTTTCGTTGCTTTCTTCAAAATGACAGCATGCCAGAAAGAAAAATCTCTTATCTTTCAACGAAGTATGTTTTTTAACAATAGGCCTAATTACGTCAGTTACTTTTTGGATAGTTGCAAATAATGATGGACTGTAGCTTTAAGAAATTTGTGGAACTCAAGTTGTGCTTGGCAAGGTTTACTTTAGAATTCGAAAATGCATCGACACCAAAAAAAAGTGTAATTTTGGTCCTATAGTTGAAGTGTCAAACTAGAACAAATTTAATGATAAATTAAATAAATTTAAATAATTTATTCCTACCTTGCTTTGACAGTGATGAAGGAGCTGCGTGACCTTTTGGAATCTACGAAGATTGACCTTCCTGTTGACATCAATGATCCATACGACCTAGGTCTTCTTCTCAGACATTTACGTCACCATTCAAATCTTCTTGCTCGTATTGGAGACCCCGATGTCAAAAAGGAAGTCCTCAGCGCCATGAATGAAAAC

>SS79-A

GCACGACGTGCCGCAGAGGAAAAACCTCAAAAGGGAAAGAAGGGACGAAAAGGAAAGAAACGACGAAAGGGAAAGAAGGGACGAAAAGGAAAGAAGGGACGAAAAGGAAAGAAACGACGAAAGGGAAAGAAAAGACGAAAGGGAAAGAAAGGACGAAAGGGAAAGAAAGGACGAAAGGGAAAGAAAGGACGAAAGGGAAAGAAAGGACGAAAGGGAAAGAAAGGACGAAAGGGAAAGAAAGGACGAAAGGGAAAGAAAGGACGAAAGGGAAAGAAAGGAAAACCAATCCAACCTGAAGAAACACCAGCCATTCCGACGGAGATAAAAGCTGCAGAAATAGAAAAGGAACCAAAAACAGAAGTGGTTGTGGAACCAGTTATTCCGGAAGCAGATATTGCGGAGACCGAAATACAACAAATTAAAGCAGAAGTAGAACCAGTTGAAACAAAGCCAGAAATAGTTATGGAACCAGATGTGGAACCAGTTATTCCAGAAACGGAACTTACAGAGACCGGAAAAGAAGCGAAAGTAGAACAAGTTAAACCAGTGGAAGGGAAGCTAAACTTA------------------------------------------------------------------------------------------------------------------------------------------------------------------------------------------------------------------------------------------------------------------------------------------------------------------------------------------------------------------------------------------------------------------------------------------------------------------------------GGAAAAGGAAAGGGCAAAGGAAAGAAGGTTAGAAAGGGAAAGAAAGGACGAAAGGGAAAGAAAGGACGAAAGGGAAAGAAAGGACGGAAAGGAAAGAAGGGGCGAAAAGGAAAGAAAAGAAGAAAAGGAAAAAAAGGACGAAAGGGAAAGAAAGGACGAAAGGGAAAGAAAGGACGAAAGGGCAAGAAAGGACGAAAGGGAAAGAAAGGACGAAAGGGAAAGAAAGGACGAAAGGGAAAGAAAGGACGAAGGGGAAAGAAAGGA------------------CGAAAGGGCAAGAAAGGAAATCCAATCAAAACTGAAGAAACACCAGCCATTCTGACGGAGATAAAAGCTGCAGAATTAGAAAAGGAACCAAAAACAGAAATAGTTATGAAACCAGTTATTCCCGAAACGGAACTAACTGAGACCGGAAAAGAAGCAGAAGTAGAACAAGTTAAACCAGTGGAAAGGAAGCTAAAAATAGGAAAAGGAAAGGGCAAAGGAAAGAAGGTTAGAAAGGGAAAGAAAGGACGAAAGGGAAAGAAAGGACGAAAGGGAAAGAAAGGGCGGAAAGGAAAGAAGGGGCGAAAAGGAAAGAAAAGAAGAAAAGGAAAGAAAAGACGAAAAGGAAAGAAACGACGAAAGGGAAAGAAAGGACGAAAGGGAAAAAAAGGACGAAAGGGAAAGAAAGGACGAAAGGGAAAAAAAGGACGAAAGGGAAAAAAAGGACGAAAGGGAAAGAAAGGACGAAAGGGAAAGAAAGGCAAACCAATCCAAACTGAAGAAACACCAGCCATTCCGACGGAGATAAAAGCTACAGAAATAGAAAAGGAACCAAAAACAGAAGTGGTTGTGGAACCACTTATTCAGGAAAAAGATGTTGCGGAGACCGAAATACAACCCATCGAAGCAGAAGTAGAACCAGTTGAACCAAAGACAGAAATAGTTATGGAACCAGTTATTCCGGAAGCAGATATTGCGGAGACCGAAATACAACCCATCAAAGCAGAAGAAGAACCAGTT------------------------------------------------------------------------------------------------------------GAACCAAAGCCAGAAATAGTTATGGAACCAGATGAGGAACCAGTTATTCCGGAAGCAGATATTGCGGAGACCGAAATACAACCCATCAAAGCAGAAGAAGAACCAGTTGAACCAAAGCCAGAAATAGTTATGGAACCAGATGAGGAACCAGTTATTCCGGAAGCAGATATTGCGGAGACCGAAATACAACCCATCAAAGCAGAAGAAGAACCAGTTGAACCAAAGCCAGAAATAGTTATGGAACCAGATGAGGAACCAGTTATTCCGGAAGCAGATATTGCGGAGACCGAAATACAACCCATCAGAGCAGAAGAAGAACCAGTTGAACCAAAGCCAGAAATAGTTATGGAACCAGATGAGGAACCAGTCATTCCAGAAACAGAACTAACAGAGACAGACAAACAACCAATCGAAGCAGAAGTAGAACCAGTTGAACCAAAAACAGAAGTGGTTGTGGAACCAGTTATTCCGGAAGCAGATATTGCGGAGACCGAAATACAACCCATCGAAGCAGAAGTAGAACCAGTTGAACCAAAGCCAGAAATAGTTATGGAACCAGATGTGGAACCAGTAATTCCAGAAACAGATCTTACAGAGACCGAAAAACAACCAATCGAAGCAGAAGTAGAACCAGTTGAACCGAAGACAGAGATAGTCGAACCAGAAACAGAGCCAGTCGAAGAAGCCGAGGAAGAAGCGGTTGAAGCACCCGTTATGGAACCAGTTATACCTGAGATAGAACCAAAGACAGAACCGGAAGTTGAAGCCGAGGAAAAAGAAGTCGAAACAAACGTTGAACCAGAACTACTGGAGACAGGAAAACCGATTGAAATTGGCCAAGCAACTGAAATCGAGGAGCCACCAGAAAAACAACCGGTGGAATCGGAGTCACAACCAGCGGAAGTTGAGATGGAACAAATTGAAACAAAGGAAGAACAAACTGAACCAAAGGAAGAACTAGCAGGAATTGATGAGAAATGTAAGTTTTAGTAACAATTATTTATTTTATGATTCCTTTTGAGCTGTTTCGGTATGCTTCGATGCAAGTCAAATTTGTGGATGTTTTAAATCCATCATTCAATTATGTTTGCAATGTAGTCATGAAGTTTGTTCGTTTGTTCAACACAGATATGGGACATATACGCATGAGCAGACCTTTTTTGTGCACAGATCTTCGTTCAGGTTAATCGTGCATGGCAACAAAAATTGGTTCATTCTGTTTTATGTCGTGCAGTCTTATTTTGCTTGAATTAAGCATTGCTGTAAAGTTCAAGTTAAAGGTACAGTCCATCATTTGTAATTTGTGCATTTTGTTCGTTTGAAGAAACAAACATGCTCAAAATCTAAAGAAGGCTGCTGAACAACTAACCTG-GGCCCAATTTCATAGCACTGCTTAACAGTAAGCAGAAAAGTTGTGCTTACTATAGCAGAAAATCATGCTGCTGCTTAAGCACTACTTCATGCCGTGAATGTGCTTACAGTTGCGCGCGCATGGAGATTTCTATTGTTACGTCACTGATTTAAGCAAAGTTTTCTTCTGGGGTTAAGCGTGCTTTTACTGTGCTTACTGCTTAACAGTCCCTATGAAATAGATGGAGGCTCAGTAAGCACAAAGTCGGCTGTTAAGCAGCGCTATGAAATTGGGCCCTGATGAAGTTTCAGCTCAGCTTCAGCTGTATAAATTATTTCGAGAAAAACAAAATTCTAGGATCTCCATTTCAAATCAAAAGTTGCTGAATCTTGTTTTGAATCGGGCCTGAATTCAGCGTCCGAGGCACGTTCTCATCACTGAGCCGATTGCAGCTACGCGATGTCGCGCACGAATTAAAGCAATCGAGGGCGCGTGTGCGTTGTGTCAACGCTTGCGCGCGCTTCGCCAAATAAAAAAAAAAGAACTAAACGGAAATTTCTTTAGACGCATGTCTTTAGACGCACCGTTGGAAAGGCCTACTTTCGTTGCTTTCTTCAAAATGACAGCATGCCAGAAAGAAAAATCTCTTATCTTTCAACGAAGTATGTTTTTTAACAATAGGCCTAATTACGTCAGTTACTTTTTGGATAGTTGCAAATAATGATGGACTGTAGCTTTAAGAAATTTGTGGAACTCAAGTTGTGCTTGGCAAGTTTTACTTTAGAATTCGAAAATGCATCGACACCAAAAAAAAGTGTAATTTTGGTCCTATAGTTGAAGTGTCAAACTAGAACAAATTTAATGATAAATTAAATAAATTTAAATAATTTATTCCTACCTTGCTTTGACAGTGATGAAGGAGCTGCGTGACCTTTTGGAATCTACGAAGATTGACCTTCCTGTTGACATCAATGATCCATACGACCTAGGTCTTCTTCTCAGACATTTACGTCACCATTCAAATCTTCTTGCTCGTATTGGAGACCCCGATGTCAAAAAGGAAGTCCTCAGCGCCATGAATGAAAAC

>SS79-B

GCACGACGTGCCGCAGAGGAAAAACCTCAAAAGGGAAAGAAGGGACGAAAAGGAAAGAAACGACGAAAGGGAAAGAAGGGACGAAAAGGAAAGAAGGGACGAAAAGGAAAGAAACGACGAAAGGGAAAGAAAAGACGAAAGGGAAAGAAAGGACGAAAGGGAAAGAAAGGACGAAAGGGAAAGAAAGGACGAAAGGGAAAGAAAGGACGAAAGGGAAAGAAAGGACGAAAGGGAAAGAAAGGACGAAAGGGAAAGAAAGGACGAAAGGGAAAGAAAGGAAAACCAATCCAACCTGAAGAAACACCAGCCATTCCGACGGAGATAAAAGCTGCAGAAATAGAAAAGGAACCAAAAACAGAAGTGGTTGTGGAACCAGTTATTCCGGAAGCAGATATTGCGGAGACCGAAATACAACAAATTAAAGCAGAAGTAGAACCAGTTGAAACAAAGCCAGAAATAGTTATGGAACCAGATGTGGAACCAGTTATTCCAGAAACGGAACTTACAGAGACCGGAAAAGAAGCGAAAGTAGAACAAGTTAAACCAGTGGAAGGGAAGCTAAACTTA------------------------------------------------------------------------------------------------------------------------------------------------------------------------------------------------------------------------------------------------------------------------------------------------------------------------------------------------------------------------------------------------------------------------------------------------------------------------------GGAAAAGGAAAGGGCAAAGGAAAGAAGGTTAGAAAGGGAAAGAAAGGACGAAAGGGAAAGAAAGGACGAAAGGGAAAGAAAGGACGGAAAGGAAAGAAGGGGCGAAAAGGAAAGAAAAGAAGAAAAGGAAAAAAAGGACGAAAGGGAAAGAAAGGACGAAAGGGAAAGAAAGGACGAAAGGGCAAGAAAGGACGAAAGGGAAAGAAAGGACGAAAGGGAAAGAAAGGACGAAAGGGAAAGAAAGGACGAAGGGGAAAGAAAGGA------------------CGAAAGGGCAAGAAAGGAAATCCAATCAAAACTGAAGAAACACCAGCCATTCTGACGGAGATAAAAGCTGCAGAATTAGAAAAGGAACCAAAAACAGAAATAGTTATGAAACCAGTTATTCCCGAAACGGAACTAACTGAGACCGGAAAAGAAGCAGAAGTAGAACAAGTTAAACCAGTGGAAAGGAAGCTAAAAATAGGAAAAGGAAAGGGCAAAGGAAAGAAGGTTAGAAAGGGAAAGAAAGGACGAAAGGGAAAGAAAGGACGAAAGGGAAAGAAAGGACGGAAAGGAAAGAAGGGGCGAAAAGGAAAGAAAAGAAGAAAAGGAAAGAAAAGACGAAAAGGAAAGAAACGACGAAAGGGAAAGAAAGGACGAAAGGGAAAAAAAGGACGAAAGGGAAAGAAAGGACGAAAGGGAAAAAAAGGACGAAAGGGAAAAAAAGGACGAAAGGGAAAGAAAGGACGAAAGGGAAAGAAAGGCAAACCAATCCAAACTGAAGAAACACCAGCCATTCCGACGGAGATAAAAGCTACAGAAATAGAAAAGGAACCAAAAACAGAAGTGGTTGTGGAACCACTTATTCAGGAAAAAGATGTTGCGGAGACCGAAATACAACCCATCGAAGCAGAAGTAGAACCAGTTGAACCAAAGACAGAAATAGTTATGGAACCAGTTATTCCGGAAGCAGATATTGCGGAGACCGAAATACAACCCATCAAAGCAGAAGAAGAACCAGTT------------------------------------------------------------------------------------------------------------GAACCAAAGCCAGAAATAGTTATGGAACCAGATGAGGAACCAGTTATTCCGGAAGCAGATATTGCGGAGACCGAAATACAACCCATCAAAGCAGAAGAAGAACCAGTTGAACCAAAGCCAGAAATAGTTATGGAACCAGATGAGGAACCAGTTATTCCGGAAGCAGATATTGCGGAGACCGAAATACAACCCATCAAAGCAGAAGAAGAACCAGTTGAACCAAAGCCAGAAATAGTTATGGAACCAGATGAGGAACCAGTTATTCCGGAAGCAGATATTGCGGAGACCGAAATACAACCCATCAGAGCAGAAGAAGAACCAGTTGAACCAAAGCCAGAAATAGTTATGGAACCAGATGAGGAACCAGTCATTCCAGAAACAGAACTAACAGAGACAGACAAACAACCAATCGAAGCAGAAGTAGAACCAGTTGAACCAAAAACAGAAGTGGTTGTGGAACCAGTTATTCCGGAAGCAGATATTGCGGAGACCGAAATACAACCCATCGAAGCAGAAGTAGAACCAGTTGAACCAAAGCCAGAAATAGTTATGGAACCAGATGTGGAACCAGTAATTCCAGAAACAGATCTTACAGAGACCGAAAAACAACCAATCGAAGCAGAAGTAGAACCAGTTGAACCGAAGACAGAGATAGTCGAACCAGAAACAGAGCCAGTCGAAGAAGCCGAGGAAGAAGCGGTTGAAGCACCCGTTATGGAACCAGTTATACCTGAGATAGAACCAAAGACAGAACCGGAAGTTGAAGCCGAGGAAAAAGAAGTCGAAACAAACGTTGAACCAGAACTACTGGAGACAGGAAAACCGATTGAAATTGGCCAAGCAACTGAAATCGAGGAGCCACCAGAAAAACAACCGGTGGAATCGGAGTCACAACCAGCGGAAGTTGAGATGGAACAAATTGAAACAAAGGAAGAACAAACTGAACCAAAGGAAGAACTAGCAGGAATTGATGAGAAATGTAAGTTTTAGTAACAATTATTTATTTTATGATTCCTTTTGAGCTGTTTCGGTATGCTTCGATGCAAGTCAAATTTGTGGATGTTTTAAATCCATCATTCAATTATGTTTGCAATGTAGTCATGAAGTTTGTTCGTTTGTTCAACACAGATATGGGACATATACGCATGAGCAGACCTTTTTTGTGCACAGATCTTCGTTCAGGTTAATCGTGCATGGCAACAAAAATTGGTTCATTCTGTTTTATGTCGTGCAGTCTTATTTTGCTTGAATTAAGCATTGCTGTAAAGTTCAAGTTAAAGGTACAGTCCATCATTTGTAATTTGTGCATTTTGTTCGTTTGAAGAAACAAACATGCTCAAAATCTAAAGAAGGCTGCTGAACAACTAACCTG-GGCCCAATTTCATAGCACTGCTTAACAGTAAGCAGAAAAGTTGTGCTTACTATAGCAGAAAATCATGCTGCTGCTTAAGCACTACTTCATGCCGTGAATGTGCTTACAGTTGCGCGCGCATGGAGATTTCTATTGTTACGTCACTGATTTAAGCAAAGTTTTCTTCTGGGGTTAAGCGTGCTTTTGCTGTGCTTACTGCTTAACAGTCCCTGTGAAATAGATGGAGGCTCGGTAAGCACAAAGTCGGCTGTTAAGCAGCGCTATGAAATTGGGCCCTGATGAAGTTTCAGCTCAGCTTCAGCTGTATAAATTATTTCGAGAAAAACAAAATTCTAGGATCTCCATTTCAAATCAAAAGTTGCTGAATCTTGTTTTGAATTGGGCCTGAATTCAGCGTCCGAGGCACGTTCTCATCACTGAGCCGATTGCAGCTACGCGATGTCGCGCACGAATTAAAGCAATCGAGGGCGCGTGTGCGTTGTGTCAACGCTTGCGCGCGCTTCGCCAAATAAAAAAAAAAGAACTAAACGGAAATTTCTTTAGACGCATGTCTTTAGACGCACCGTTGGAAAGGCCTACTTTCGTTGCTTTCTTCAAAATGACAGCATGCCAGAAAGAAAAATCTCTTATCTTTCAACGAAGTATGTTTTCTAACAATAGGCCTAATTACGTCAGTTACTTTTTGGATAGTTGCAAATAATGATGGACTGTAGCTTTAAGAAATTTGTGGAACTCAAGTTGTGCTTGGCAAGTTTTACTTTAGAATTCGAAAATGCATCGACACCAAAAAAAAGTGTAATTTTGGTCCTATAGTTGAAGTGTCAAACTAGAACAAATTTAATGATAAATTAAATAAATTTAAATAATTTATTCCTACCTTGCTTTGACAGTGATGAAGGAGCTGCGTGACCTTTTGGAATCTACGAAGATTGACCTTCCTGTTGACATCAATGATCCATACGACCTAGGTCTTCTTCTCAGACATTTACGTCACCATTCAAATCTTCTTGCTCGTATTGGAGACCCCGATGTCAAAAAGGAAGTCCTCAGCGCCATGAATGAAAAC

>SS27-A

GCACGACGTGCCGCAGAGGAAAAACCTCAAAAGGGAAAGAAGGGACGAAAAGGAAAGAAACGACGAAAGGGAAAGAAGGGACGAAAAGGAAAGAAGGGACGAAAAGGAAAGAAACGACGAAAGGGAAAGAAAAGACGAAAGGGAAAGAAAGGACGAAAGGGAAAGAAAGGACGAAAGGGAAAGAAAGGACGAAAGGGAAAGAAAGGACGAAAGGGAAAGAAAGGACGAAAGGGAAAGAAAGGACGAAAGGGAAAGAAAGGACGAAAGGGAAAGAAAGGAAAACCAATCCAACCTGAAGAAACACCAGCCATTCCGACGGAGATAAAAGCTGCAGAAATAGAAAAGGAACCAAAAACAGAAGTGGTTGTGGAACCAGTTATTCCGGAAGCAGATATTGCGGAGACCGAAATACAACAAATTAAAGCAGAAGTAGAACCAGTTGAAACAAAGCCAGAAATAGTTATGGAACCAGATGTGGAACCAGTTATTCCAGAAACGGAACTTACAGAGACCGGAAAAGAAGCGAAAGTAGAACAAGTTAAACCAGTGGAAGGGAAGCTAAACTTA------------------------------------------------------------------------------------------------------------------------------------------------------------------------------------------------------------------------------------------------------------------------------------------------------------------------------------------------------------------------------------------------------------------------------------------------------------------------------GGAAAAGGAAAGGGCAAAGGAAAGAAGGTTAGAAAGGGAAAGAAAGGACGAAAGGGAAAGAAAGGACGAAAGGGAAAGAAAGGACGGAAAGGAAAGAAGGGGCGAAAAGGAAAGAAAAGAAGAAAAGGAAAAAAAGGACGAAAGGGAAAGAAAGGACGAAAGGGAAAGAAAGGACGAAAGGGCAAGAAAGGACGAAAGGGAAAGAAAGGACGAAAGGGAAAGAAAGGACGAAAGGGAAAGAAAGGACGAAGGGGAAAGAAAGGA------------------CGAAAGGGCAAGAAAGGAAATCCAATCAAAACTGAAGAAACACCAGCCATTCTGACGGAGATAAAAGCTGCAGAATTAGAAAAGGAACCAAAAACAGAAATAGTTATGAAACCAGTTATTCCCGAAACGGAACTAACTGAGACCGGAAAAGAAGCANAAGTAGAACAAGTTAAACCAGTGGAAAGGAAGCTAAAAATAGGAAAAGGAAAGGGCAAAGGAAAGAAGGTTAGAAAGGGAAAGAAAGGACGAAAGGGAAAGAAAGGACGAAAGGGAAAGAAAGGACGGAAAGGAAAGAAGGGGCGAAAAGGAAAGAAAAGAAGAAAAGGAAAGAAAAGACGAAAAGGAAAGAAACGACGAAAGGGAAAGAAAGGACGAAAGGGAAAAAAAGGACGAAAGGGAAAGAAAGGACGAAAGGGAAAAAAAGGACGAAAGGGAAAAAAAGGACGAAAGGGAAAGAAAGGACGAAAGGGAAAGAAAGGCAAACCAATCCAAACTGAAGAAACACCAGCCATTCCGACGGAGATAAAAGCTACAGAAATAGAAAAGGAACCAAAAACAGAAGTGGTTGTGGAACCACTTATTCAGGAAAAAGATGTTGCGGAGACCGAAATACAACCCATCGAAGCAGAAGTAGAACCAGTTGAACCAAAGACAGAAATAGTTATGGAACCAGTTATTCCGGAAGCAGATATTGCGGAGACCGAAATACAACCCATCAAAGCAGAAGAAGAACCAGTT------------------------------------------------------------------------------------------------------------------------------------------------------------------------------------------------------------------------------------------------------------------------------------------------------------------------------------GAACCAAAGCCAGAAATAGTTATGGAACCAGATGAGGAACCAGTTATTCCGGAAGCAGATATTGCGGAGACCGAAATACAACCCATCAGAGCAGAAGAAGAACCAGTTGAACCAAAGCCAGAAATAGTTATGGAACCAGATGAGGAACCAGTCATTCCAGAAACAGAACTAACAGAGACAGACAAACAACCAATCGAAGCAGAAGTAGAACCAGTTGAACCAAAAACAGAAGTGGTTGTGGAACCAGTTATTCCGGAAGCAGATATTGCGGAGACCGAAATACAACCCATCGAAGCAGAAGTAGAACCAGTTGAACCAAAGCCAGAAATAGTTATGGAACCAGATGTGGAACCAGTAATTCCAGAAACAGATCTTACAGAGACCGAAAAACAACCAATCGAAGCAGAAGTAGAACCAGTTGAACCGAAGACAGAGATAGTCGAACCAGAAACAGAGCCAGTCGAAGAAGCCGAGGAAGAAGCGGTTGAAGCACCCGTTATGGAACCAGTTATACCTGAGATAGAACCAAAGACAGAACCGGAAGTTGAAGCCGAGGAAAAAGAAGTCGAAACAAACGTTGAACCAGAACTACTGGAGACAGGAAAACCGATTGAAATTGGCCAAGCAACTGAAATCGAGGAGCCACCAGAAAAACAACCGGTGGAATCGGAGTCACAACCAGCGGAAGTTGAGATGGAACAAATTGAAACAAAGGAAGAACAAACTGAACCAAAGGAAGAACTAGCAGGAATTGATGAGAAATGTAAGTTTTAGTAACAATTATTTATTTTATGATTCCTTTTGAGCTGTTTCGGTATGCTTCGATGCAAGTCAAATTTGTGGATGTTTTAAATCCATCATTCAATTATGTTTGCAATGTAGTCATGAAGTTTGTTCGTTTGTTCAACACAGATATGGGACATATACGCATGAGCAGACCTTTTTTGTGCACAGATCTTCGTTCAGGTTAATCGTGCATGGCAACAAAAATTGGTTCATTCTGTTTTATGTCGTGCAGTCCTATTTTGCTTGAATTAAGCATTGCTGTAAAGTTCAAGTTAAAGGTACAGTCCATCATTTGTAATTTGTGCATTTTGTTCGTTTGAAGAAACAAACATGCTCAAA-TCTAAAGAAGGCTGCTGAACAACTAACCTG-GGCCCAATTTCATAGCACTGCTTAACAGTAAGCAGAAAAGTTGTGCTTACTATAGCAGAAAATCATGCTGCTGCTTAAGCACTACTTCATGCCGTGAATGTGCTTACAGTTGCGCGCGCATGGAGATTTCTATTGTTACGTCACTGATTTAAGCAAAGTTTTCTTCTGGGGTTAAGCGTGCTTTTGCTGTGCTTACTGCTTAACAGTCCCTATGAAATAGATGGAGGCTCGGTAAGCACAAAGTCGGCTGTTAAGCAGCGCTATGAAATTGGGCCCTGATGAAGTTTCAGCTCAGCTTCAGCTGTATAAATTATTTCGAGAAAAACAAAATTCTAGGATCTCCATTTCAAATCAAAAGTTGCTGAATCTCGTTTTGAATTGGGCCTGAATTCAGCGTCCGAGGCACGTTCTCATCACTGAGCCGATTGCAGCTACGCGATGTCGCGCACGAATTAAAGCAATCGAGGGCGCGTGTGCGTTGTGTCAACGCTTGCGCGCGCTTCGCCAAAT-AAAAAAAAAGAACTAAACGGAAATTTCTTTAGACGCATGTCTTTAGACGCACCGTTGGAAAGGCCTACTTTCGTTGCTTTCTTCAAAATGACAGCATGCCAGAAAGAAAAATCTCTTATCTTTCAACGAAGTATGTTTTTTAACAATAGGCCTAATTACGTCAGTTACTTTTTGGATAGTTGCAAATAATGATGGACTGTAGCTTTAAGAAATTTGTGGAACTCAAGTTGTGCTTGGCAAGTTTTACTTTAGAATTCGAAAATGCATCGACACCAAAAAAAAGTGTAATTTTGGTCCTATAGTTGAAGTGTCAAACTAGAACAAATTTAATGATAAATTAAATAAATTTAAATAATTTATTCCTACCTTGCTTTGACAGTGATGAAGGAGCTGCGTGACCTTTTGGAATCTACGAAGATTGACCTTCCTGTTGACATCAATGATCCATACGACCTAGGTCTTCTTCTCAGACATTTACGTCACCATTCAAATCTTCTTGCTCGTATTGGAGACCCCGATGTCAAAAAGGAAGTCCTCAGCGCCATGAATGAAAAC

>SS27-B

GCACGACGTGCCGCAGAGGAAAAACCTCAAAAGGGAAAGAAGGGACGAAAAGGAAAGAAACGACGAAAGGGAAAGAAGGGACGAAAAGGAAAGAAGGGACGAAAAGGAAAGAAACGACGAAAGGGAAAGAAAAGACGAAAGGGAAAGAAAGGACGAAAGGGAAAGAAAGGACGAAAGGGAAAGAAAGGACGAAAGGGAAAGAAAGGACGAAAGGGAAAGAAAGGACGAAAGGGAAAGAAAGGACGAAAGGGAAAGAAAGGACGAAAGGGAAAGAAAGGAAAACCAATCCAACCTGAAGAAACACCAGCCATTCCGACGGAGATAAAAGCTGCAGAAATAGAAAAGGAACCAAAAACAGAAGTGGTTGTGGAACCAGTTATTCCGGAAGCAGATATTGCGGAGACCGAAATACAACAAATTAAAGCAGAAGTAGAACCAGTTGAAACAAAGCCAGAAATAGTTATGGAACCAGATGTGGAACCAGTTATTCCAGAAACGGAACTTACAGAGACCGGAAAAGAAGCGAAAGTAGAACAAGTTAAACCAGTGGAAGGGAAGCTAAACTTA------------------------------------------------------------------------------------------------------------------------------------------------------------------------------------------------------------------------------------------------------------------------------------------------------------------------------------------------------------------------------------------------------------------------------------------------------------------------------GGAAAAGGAAAGGGCAAAGGAAAGAAGGTTAGAAAGGGAAAGAAAGGACGAAAGGGAAAGAAAGGACGAAAGGGAAAGAAAGGACGGAAAGGAAAGAAGGGGCGAAAAGGAAAGAAAAGAAGAAAAGGAAAAAAAGGACGAAAGGGAAAGAAAGGACGAAAGGGAAAGAAAGGACGAAAGGGCAAGAAAGGACGAAAGGGAAAGAAAGGACGAAAGGGAAAGAAAGGACGAAAGGGAAAGAAAGGACGAAGGGGAAAGAAAGGA------------------CGAAAGGGCAAGAAAGGAAATCCAATCAAAACTGAAGAAACACCAGCCATTCTGACGGAGATAAAAGCTGCAGAATTAGAAAAGGAACCAAAAACAGAAATAGTTATGAAACCAGTTATTCCCGAAACGGAACTAACTGAGACCGGAAAAGAAGCAGAAGTAGAACAAGTTAAACCAGTGGAAAGGAAGCTAAAAATAGGAAAAGGAAAGGGCAAAGGAAAGAAGGTTAGAAAGGGAAAGAAAGGACGAAAGGGAAAGAAAGGACGAAAGGGAAAGAAAGGACGGAAAGGAAAGAAGGGGCGAAAAGGAAAGAAAAGAAGAAAAGGAAAGAAAAGACGAAAAGGAAAGAAACGACGAAAGGGAAAGAAAGGACGAAAGGGAAAAAAAGGACGAAAGGGAAAGAAAGGACGAAAGGGAAAAAAAGGACGAAAGGGAAAAAAAGGACGAAAGGGAAAGAAAGGACGAAAGGGAAAGAAAGGCAAACCAATCCAAACTGAAGAAACACCAGCCATTCCGACGGAGATAAAAGCTACAGAAATAGAAAAGGAACCAAAAACAGAAGTGGTTGTGGAACCACTTATTCAGGAAAAAGATGTTGCGGAGACCGAAATACAACCCATCGAAGCAGAAGTAGAACCAGTTGAACCAAAGACAGAAATAGTTATGGAACCAGTTATTCCGGAAGCAGATATTGCGGAGACCGAAATACAACCCATCAAAGCAGAAGAAGAACCAGTT------------------------------------------------------------------------------------------------------------GAACCAAAGCCAGAAATAGTTATGGAACCAGATGAGGAACCAGTTATTCCGGAAGCAGATATTGCGGAGACCGAAATACAACCCATCAAAGCAGAAGAAGAACCAGTTGAACCAAAGCCAGAAATAGTTATGGAACCAGATGAGGAACCAGTTATTCCGGAAGCAGATATTGCGGAGACCGAAATACAACCCATCAAAGCAGAAGAAGAACCAGTTGAACCAAAGCCAGAAATAGTTATGGAACCAGATGAGGAACCAGTTATTCCGGAAGCAGATATTGCGGAGACCGAAATACAACCCATCAGAGCAGAAGAAGAACCAGTTGAACCAAAGCCAGAAATAGTTATGGAACCAGATGAGGAACCAGTCATTCCAGAAACAGAACTAACAGAGACAGACAAACAACCAATCGAAGCAGAAGTAGAACCAGTTGAACCAAAAACAGAAGTGGTTGTGGAACCAGTTATTCCGGAAGCAGATATTGCGGAGACCGAAATACAACCCATCGAAGCAGAAGTAGAACCAGTTGAACCAAAGCCAGAAATAGTTATGGAACCAGATGTGGAACCAGTAATTCCAGAAACAGATCTTACAGAGACCGAAAAACAACCAATCGAAGCAGAAGTAGAACCAGTTGAACCGAAGACAGAGATAGTCGAACCAGAAACAGAGCCAGTCGAAGAAGCCGAGGAAGAAGCGGTTGAAGCACCCGTTATGGAACCAGTTATACCTGAGATAGAACCAAAGACAGAACCGGAAGTTGAAGCCGAGGAAAAAGAAGTCGAAACAAACGTTGAACCAGAACTACTGGAGACAGGAAAACCGATTGAAATTGGCCAAGCAACTGAAATCGAGGAGCCACCAGAAAAACAACCGGTGGAATCGGAGTCACAACCAGCGGAAGTTGAGATGGAACAAATTGAAACAAAGGAAGAACAAACTGAACCAAAGGAAGAACTAGCAGGAATTGATGAGAAATGTAAGTTTTAGTAACAATTATTTATTTTATGATTCCTTTTGAGCTGTTTCGGTATGCTTCGATGCAAGTCAAATTTGTGGATGTTTTAAATCCATCATTCAATTATGTTTGCAATGTAGTCATGAAGTTTGTTCGTTTGTTCAACACAGATATGGGACATATACGCATGAGCAGACCTTTTTTGTGCACAGATCTTCGTTCAGGTTAATCGTGCATGGCAACAAAAATTGGTTCATTCTGTTTTATGTCGTGCAGTCTTATTTTGCTTGAATTAAGCATTGCTGTAAAGTTCAAGTTAAAGGTACAGTCCATCATTTGTAATTTGTGCATTTTGTTCGTTTGAAGAAACAAACATGCTCAAAATCTAAAGAAGGCTGCTGAACAACTAACCTG-GGCCCAATTTCATAGCACTGCTTAACAGTAAGCAGAAAAGTTGTGCTTACTATAGCAGAAAATCATGCTGCTGCTTAAGCACTACTTCATGCCGTGAATGTGCTTACAGTTGCGCGCGCATGGAGATTTCTATTGTTACGTCACTGATTTAAGCAAAGTTTTCTTCTGGGGTTAAGCGTGCTTTTGCTGTGCTTACTGCTTAACAGTCCCTATGAAATAGATGGAGGCTCGGTAAGCACAAAGTCGGCTGTTAAGCAGCGCTATGAAATTGGGCCCTGATGAAGTTTCAGCTCAGCTTCAGCTGTATAAATTATTTCGAGAAAAACAAAATTCTAGGATCTCCATTTCAAATCAAAAGTTGCTGAATCTTGTTTTGAATTGGGCCTGAATTCAGCGTCCGAGGCACGTTCTCATCACTGAGCCGATTGCAGCTACGCGATGTCGCGCACGAATTAAAGCAATCGAGGGCGCGTGTGCGTTGTGTCAACGCTTGCGCGCGCTTCGCCAAATAAAAAAAAAAGAACTAAACGGAAATTTCTTTAGACGCATGTCTTTAGACGCACCGTTGGAAAGGCCTACTTTCGTTGCTTTCTTCAAAATGACAGCATGCCAGAAAGAAAAATCTCTTATCTTTCAACGAAGTATGTTTTTTAACAATAGGCCTAATTACGTCAGTTACTTTTTGGATAGTTGCAAATAATGATGGACTGTAGCTTTAAGAAATTTGTGGAACTCAAGTTGTGCTTGGCAAGTTTTACTTTAGAATTCGAAAATGCATCGACACCAAAAAAAAGTGTAATTTTGGTCCTATAGTTGAAGTGTCAAACTAGAACAAATTTAATGATAAATTAAATAAATTTAAATAATTTATTCCTACCTTGCTTTGACAGTGATGAAGGAGCTGCGTGACCTTTTGGAATCTACGAAGATTGACCTTCCTGTTGACATCAATGATCCATACGACCTAGGTCTTCTTCTCAGACATTTACGTCACCATTCAAATCTTCTTGCTCGTATTGGAGACCCCGATGTCAAAAAGGAAGTCCTCAGCGCCATGAATGAAAAC

>SS85-B

GCACGACGTGCCGCAGAGGAAAAACCTCAAAAGGGAAAGAAGGGACGAAAAGGAAAGAAACGACGAAAGGGAAAGAAGGGACGAAAAGGAAAGAAGGGACGAAAAGGAAAGAAACGACGAAAGGGAAAGAAAAGACGAAAGGGAAAGAAAGGACGAAAGGGAAAGAAAGGACGAAAGGGAAAGAAAGGACGAAAGGGAAAGAAAGGACGAAAGGGAAAGAAAGGACGAAAGGGAAAGAAAGGACGAAAGGGAAAGAAAGGACGAAAGGGAAAGAAAGGAAAACCAATCCAACCTGAAGAAACACCAGCCATTCCGACGGAGATAAAAGCTGCAGAAATAGAAAAGGAACCAAAAACAGAAGTGGTTGTGGAACCAGTTATTCCGGAAGCAGATATTGCGGAGACCGAAATACAACAAATTAAAGCAGAAGTAGAACCAGTTGAAACAAAGCCAGAAATAGTTATGGAACCAGATGTGGAACCAGTTATTCCAGAAACGGAACTTACAGAGACCGGAAAAGAAGCGAAAGTAGAACAAGTTAAACAAGTGGAAGGGAAGCTAAACTTA------------------------------------------------------------------------------------------------------------------------------------------------------------------------------------------------------------------------------------------------------------------------------------------------------------------------------------------------------------------------------------------------------------------------------------------------------------------------------GGAAAAGGAAAGGGCAAAGGAAAGAAGGTTAGAAAGGGAAAGAAAGGACGAAAGGGAAAGAAAGGACGAAAGGGAAAGAAAGGACGGAAAGGAAAGAAGGGGCGAAAAGGAAAGAAAAGAAGAAAAGGAAAAAAAGGACGAAAGGGAAAGAAAGGACGAAAGGGAAAGAAAGGACGAAAGGGCAAGAAAGGACGAAAGGGAAAGAAAGGACGAAAGGGAAAGAAAGGACGAAAGGGAAAGAAAGGACGAAGGGGAAAGAAAGGA------------------CGAAAGGGCAAGAAAGGAAATCCAATCAAAACTGAAGAAACACCAGCCATTCTGACGGAGATAAAAGCTGCAGAATTAGAAAAGGAACCAAAAACAGAAATAGTTATGAAACCAGTTATTCCCGAAACGGAACTAACTGAGACCGGAAAAGAAGCAGAAGTAGAACAAGTTAAACCAGTGGAAAGGAAGCTAAAAATAGGAAAAGGAAAGGGCAAAGGAAAGAAGGTTAGAAAGGGAAAGAAAGGACGAAAGGGAAAGAAAGGACGAAAGGGAAAGAAAGGACGGAAAGGAAAGAAGGGGCGAAAAGGAAAGAAAAGAAGAAAAGGAAAGAAAAGACGAAAAGGAAAGAAACGACGAAAGGGAAAGAAAGGACGAAAGGGAAAAAAAGGACGAAAGGGAAAGAAAGGACGAAAGGGAAAAAAAGGACGAAAGGGAAAAAAAGGACGAAAGGGAAAGAAAGGACGAAAGGGAAAGAAAGGCAAACCAATCCAAACTGAAGAAACACCAGCCATTCCGACGGAGATAAAAGCTACAGAAATAGAAAAGGAACCAAAAACAGAAGTGGTTGTGGAACCACTTATTCAGGAAAAAGATGTTGCGGAGACCGAAATACAACCCATCGAAGCAGAAGTAGAACCAGTTGAACCAAAGACAGAAATAGTTATGGAACCAGTTATTCCGGAAGCAGATATTGCGGAGACCGAAATACAACCCATCAAAGCAGAAGAAGAACCAGTT------------------------------------------------------------------------------------------------------------GAACCAAAGCCAGAAATAGTTATGGAACCAGATGAGGAACCAGTTATTCCGGAAGCAGATATTGCGGAGACCGAAATACAACCCATCAAAGCAGAAGAAGAACCAGTTGAACCAAAGCCAGAAATAGTTATGGAACCAGATGAGGAACCAGTTATTCCGGAAGCAGATATTGCGGAGACCGAAATACAACCCATCAAAGCAGAAGAAGAACCAGTTGAACCAAAGCCAGAAATAGTTATGGAACCAGATGAGGAACCAGTTATTCCGGAAGCAGATATTGCGGAGACCGAAATACAACCCATCAGAGCAGAAGAAGAACCAGTTGAACCAAAGCCAGAAATAGTTATGGAACCAGATGAGGAACCAGTCATTCCAGAAACAGAACTAACAGAGACAGACAAACAACCAATCGAAGCAGAAGTAGAACCAGTTGAACCAAAAACAGAAGTGGTTGTGGAACCAGTTATTCCGGAAGCAGATATTGCGGAGACCGAAATACAACCCATCGAAGCAGAAGTAGAACCAGTTGAACCAAAGCCAGAAATAGTTATGGAACCAGATGTGGAACCAGTAATTCCAGAAACAGATCTTACAGAGACCGAAAAACAACCAATCGAAGCAGAAGTAGAACCAGTTGAACCGAAGACAGAGATAGTCGAACCAGAAACAGAGCCAGTCGAAGAAGCCGAGGAAGAAGCGGTTGAAGCACCCGTTATGGAACCAGTTATACCTGAGATAGAACCAAAGACAGAACCGGAAGTTGAAGCCGAGGAAAAAGAAGTCGAAACAAACGTTGAACCAGAACTACTGGAGACAGGAAAACCGATTGAAATTGGCCAAGCAACTGAAATCGAGGAGCCACCAGAAAAACAACCGGTGGAATCGGAGTCACAACCAGCGGAAGTTGAGATGGAACAAATTGAAACAAAGGAAGAACAAACTGAACCAAAGGAAGAACTAGCAGGAATTGATGAGAAATGTAAGTTTTAGTAACAATTATTTATTTTATGATTCCTTTTGAGCTGTTTCGGTATGCTTCGATGCAAGTCAAATTTGTGGATGTTTTAAATCCATCATTCAATTATGTTTGCAATGTAGTCATGAAGTTTGTTCGTTTGTTCAACACAGATATGGGACATATACGCATGAGCAGACCTTTTTGGTGCACAGATCTTCGTTCAGGTTAATCGTGCATGGCAACAAAAATTGGTTCATTCTGTTTTATGTCGTGCAGTCTTATTTTGCTTGAATTAAGCATTGCTGTAAAGTTCAAGTTAAAGGTACAGTCCATCATTTGTAATTTGTGCATTTTGTTCGTTTGAAGAAACAAACATGCTCAAAATCTAAAGAAGGCTGCTGAACAACTAACCTG-GACCCAATTTCATAGCACTGCTTAACAGTAAGCAGAAAAGTTGTGCTTACTATAGCAGAAAATCATGCTGCTGCTTAAGCACTACTTCATGCCGTGAATGTGCTTACAGTTGCGCGCGCATGGAGATTTCTATTGTTACGTCACTGATTTAAGCAAAGTTTTCTTCTGGGGTTAAGCGTGCTTTTGCTGTGCTTACTGCTTAACAGTCCCTATGAAATAGATGGAGGCTCGGTAAGCACAAAGTCGGCTGTTAAGCAGCGCTATGAAATTGGGCCCTGATGAAGTTTCAGCTCAGCTTCAGCTGTATAAATTATTTCGAGAAAAACAAAATTCTAGGATCTCCATTTCAAATCAAAAGTTGCTGAATCTTGTTTTGAATTGGGCCTGAATTCAGCGTCCGAGGCACGTTCTCATCACTGAGCCGATTGCAGCTACGCGATGTCGCGCACGAATTAAAGCAATCGAGGGCGCGTGTGCGTTGTGTCAACGCTTGCGCGCGCTTCGCCAAATAAAAAAAAAAGAACTAAACGGAAATTTCTTTAGACGCATGTCTTTAGACGCACCGTTGGAAAGGCCTACTTTCGTTGCTTTCTTCAAAATGACAGCATGCCAGAAAGAAAAATCTCTTATCTTTCAACGAAGTATGTTTTTTAACAATAGGCCTAATTACGTCAGTTACTTTTTGGATAGTTGCAAATAATGATGGACTGTAGCTTTAAGAAATTTGTGGAACTCAAGTTGTGCTTGGCAAGTTTTACTTTAGAATTCGAAAATGCATCGACACCAAAAAAAAGTGTAATTTTGGTCCTATAGTTGAAGTGTCAAACTAGAACAAATTTAATGATAAATTAAATAAATTTAAATAATTTATTCCTACCTTGCTTTGACAGTGATGAAGGAGCTGCGTGACCTTTTGGAATCTACGAAGATTGACCTTCCTGTTGACATCAATGATCCATACGACCTAGGTCTTCTTCTCAGACATTTACGTCACCATTCAAATCTTCTTGCTCGTATTGGAGACCCCGATGTCAAAAAGGAAGTCCTCAGCGCCATGAATGAAAAC

>SS26-B

GCACGACGTGCCGCAGAGGAAAAACCTCAAAAGGGAAAGAAGGGACGAAAAGGAAAGAAACGACGAAAGGGAAAGAAGGGACGAAAAGGAAAGAAGGGACGAAAAGGAAAGAAACGACGAAAGGGAAAGAAAAGACGAAAGGGAAAGAAAGGACGAAAGGGAAAGAAAGGACGAAAGGGAAAGAAAGGACGAAAGGGAAAGAAAGGACGAAAGGGAAAGAAAGGACGAAAGGGAAAGAAAGGACGAAAGGGAAAGAAAGGACGAAAGGGAAAGAAAGGAAAACCAATCCAACCTGAAGAAACACCAGCCATTCCGACGGAGATAAAAGCTGCAGAAATAGAAAAGGAACCAAAAACAGAAGTGGTTGTGGAACCAGTTATTCCGGAAGCAGATATTGCGGAGACCGAAATACAACAAATTAAAGCAGAAGTAGAACCAGTTGAAACAAAGCCAGAAATAGTTATGGAACCAGATGTGGAACCAGTTATTCCAGAAACGGAACTTACAGAGACCGGAAAAGAAGCGAAAGTAGAACAAGTTAAACCAGTGGAAGGGAAGCTAAACTTA------------------------------------------------------------------------------------------------------------------------------------------------------------------------------------------------------------------------------------------------------------------------------------------------------------------------------------------------------------------------------------------------------------------------------------------------------------------------------GGAAAAGGAAAGGGCAAAGGAAAGAAGGTTAGAAAGGGAAAGAAAGGACGAAAGGGAAAGAAAGGACGAAAGGGAAAGAAAGGACGGAAAGGAAAGAAGGGGCGAAAAGGAAAGAAAAGAAGAAAAGGAAAAAAAGGACGAAAGGGAAAGAAAGGACGAAAGGGAAAGAAAGGACGAAAGGGCAAGAAAGGACGAAAGGGAAAGAAAGGACGAAAGGGAAAGAAAGGACGAAAGGGAAAGAAAGGACGAAGGGGAAAGAAAGGA------------------CGAAAGGGCAAGAAAGGAAATCCAATCAAAACTGAAGAAACACCAGCCATTCTGACGGAGATAAAAGCTGCAGAATTAGAAAAGGAACCAAAAACAGAAATAGTTATGAAACCAGTTATTCCCGAAACGGAACTAACTGAGACCGGAAAAGAAGCAGAAGTAGAACAAGTTAAACCAGTGGAAAGGAAGCTAAAAATAGGAAAAGGAAAGGGCAAAGGAAAGAAGGTTAGAAAGGGAAAGAAAGGACGAAAGGGAAAGAAAGGACGAAAGGGAAAGAAAGGACGGAAAGGAAAGAAGGGGCGAAAAGGAAAGAAAAGAAGAAAAGGAAAGAAAAGACGAAAAGGAAAGAAACGACGAAAGGGAAAGAAAGGACGAAAGGGAAAAAAAGGACGAAAGGGAAAGAAAGGACGAAAGGGAAAAAAAGGACGAAAGGGAAAAAAAGGACGAAAGGGAAAGAAAGGACGAAAGGGAAAGAAAGGCAAACCAATCCAAACTGAAGAAACACCAGCCATTCCGACGGAGATAAAAGCTACAGAAATAGAAAAGGAACCAAAAACAGAAGTGGTTGTGGAACCACTTATTCAGGAAAAAGATGTTGCGGAGACCGAAATACAACCCATCGAAGCAGAAGTAGAACCAGTTGAACCAAAGACAGAAATAGTTATGGAACCAGTTATTCCGGAAGCAGATATTGCGGAGACCGAAATACAACCCATCAAAGCAGAAGAAGAACCAGTT------------------------------------------------------------------------------------------------------------GAACCAAAGCCAGAAATAGTTATGGAACCAGATGAGGAACCAGTTATTCCGGAAGCAGATATTGCGGAGACCGAAATACAACCCATCAAAGCAGAAGAAGAACCAGTTGAACCAAAGCCAGAAATAGTTATGGAACCAGATGAGGAACCAGTTATTCCGGAAGCAGATATTGCGGAGACCGAAATACAACCCATCAAAGCAGAAGAAGAACCAGTTGAACCAAAGCCAGAAATAGTTATGGAACCAGATGAGGAACCAGTTATTCCGGAAGCAGATATTGCGGAGACCGAAATACAACCCATCAGAGCAGAAGAAGAACCAGTTGAACCAAAGCCAGAAATAGTTATGGAACCAGATGAGGAACCAGTCATTCCAGAAACAGAACTAACAGAGACAGACAAACAACCAATCGAAGCAGAAGTAGAACCAGTTGAACCAAAAACAGAAGTGGTTGTGGAACCAGTTATTCCGGAAGCAGATATTGCGGAGACCGAAATACAACCCATCGAAGCAGAAGTAGAACCAGTTGAACCAAAGCCAGAAATAGTTATGGAACCAGATGTGGAACCAGTAATTCCAGAAACAGATCTTACAGAGACCGAAAAACAACCAATCGAAGCAGAAGTAGAACCAGTTGAACCGAAGACAGAGATAGTCGAACCAGAAACAGAGCCAGTCGAAGAAGCCGAGGAAGAAGCGGTTGAAGCACCCGTTATGGAACCAGTTATACCTGAGATAGAACCAAAGACAGAACCGGAAGTTGAAGCCGAGGAAAAAGAAGTCGAAACAAACGTTGAACCAGAACTACTGGAGACAGGAAAACCGATTGAAATTGGCCAAGCAACTGAAATCGAGGAGCCACCAGAAAAACAACCGGTGGAATCGGAGTCACAACCAGCGGAAGTTGAGATGGAACAAATTGAAACAAAGGAAGAACCAACTGAACCAAAGGAAGAACTAGCAGGAATTGATGAGAAATGTAAGTTTTAGTAACAATTATTTATTTTATGATTCCTTTTGAGCTGTTTCGGTATGCTTCGATGCAAGTCAAATTTGTGGATGTTTTAAATCCATCATTCAATTATGTTTGCAATGTAGTCATGAAGTTTGTTCGTTTGTTCAACACAGATATGGGACATATACGCATGAGCAGACCTTTTTTGTGCACAGATCTTCGTTCAGGTTAATCGTGCATGGCAACAAAAATTGGTTCATTCTGTTTTATGTCGTGCAGTCTTATTTTGCTTGAATTAAGCATTGCTGTAAAGTTCAAGTTAAAGGTACAGTCCATCATTTGTAATTTGTGCATTTTGTTCGTTTGAAGAAACAAACATGCTCAAAATCTAAAGAAGGCTGCTGAACAACTAACCTG-GGCCCAATTTCATAGCACTGCTTAACAGTAAGCAGAAAAGTTGTGCTTACTATAGCAGAAAATCATGCTGCTGCTTAAGCACTACTTCATGCCGTGAATGTGCTTACAGTTGCGCGCGCATGGAGATTTCTATTGTTACGTCACTGATTTAAGCAAAGTTTTCTTCTGGGGTTAAGCGTGCTTTTGCTGTGCTTACTGCTTAACAGTCCCTATGAAATAGATGGAGGCTCGGTAAGCACAAAGTCGGCTGTTAAGCAGCGCTATGAAATTGGGCCCTGATGAAGTTTCAGCTCAGCTTCAGCTGTATAAATTATTTCGAGAAAAACAAAATTCTAGGATCTCCATTTCAAATCAAAAGTTGCTGAATCTTGTTTTGAATTGGGCCTGAATTCAGCGTCCGAGGCACGTTCTCATCACTGAGCCGATTGCAGCTACGCGATGTCGCGCACGAATTAAAGCAATCGAGGGCGCGTGTGCGTTGTGTCAACGCTTGCGCGCGCTTCGCCAATAAAAAAAAAAAGAACTAAACGGAAATTTCTTTAGACGCATGTCTTTAGACGCACCGTTGGAAAGGCCTACTTTCGTTGCTTTCTTCAAAATGACAGCATGCCAGAAAGAAAA-TCTCTTATCTTTCAACGAAGTATGTTTTTTAACAATAGGCCTAATTACGTCAGTTACTTTTTGGATAGTTGCAAATAATGATGGACTGTAGCTTTAAGAAATTTGTGGAACTCAAGTTGTGCTTGGCAAGTTTTACTTTAGAATTCGAAAATGCATCGACACC-AAAAAAAGTGTAATTTTGGTCCTATAGTTGAAGTGTCAAACTAGAACAAATTTAATGATAAATTAAATAAATTTAAATAATTTATTCCTACCTTGCTTTGACAGTGATGAAGGAGCTGCGTGACCTTTTGGAATCTACGAAGATTGACCTTCCTGTTGACATCAATGATCCATACGACCTAGGTCTTCTTCTCAGACATTTACGTCACCATTCAAATCTTCTTGCTCGTATTGGAGACCCCGATGTCAAAAAGGAAGTCCTCAGCGCCATGAATGAAAAC

>SS98-A

GCACGACGTGCCGCAGAGGAAAAACCTCAAAAGGGAAAGAAGGGACGAAAAGGAAAGAAACGACGAAAGGGAAAGAAGGGACGAAAAGGAAAGAAGGGACGAAAAGGAAAGAAACGACGAAAGGGAAAGAAAAGACGAAAGGGAAAGAAAGGACGAAAGGGAAAGAAAGGACGAAAGGGAAAGAAAGGACGAAAGGGAAAGAAAGGACGAAAGGGAAAGAAAGGACGAAAGGGAAAGAAAGGACGAAAGGGAAAGAAAGGACGAAAGGGAAAGAAAGGAAAACCAATCCAACCTGAAGAAACACCAGCCATTCCGACGGAGATAAAAGCTGCAGAAATAGAAAAGGAACCAAAAACAGAAGTGGTTGTGGAACCAGTTATTCCGGAAGCAGATATTGCGGAGACCGAAATACAACAAATTAAAGCAGAAGTAGAACCAGTTGAAACAAAGCCAGAAATAGTTATGGAACCAGATGTGGAACCAGTTATTCCAGAAACGGAACTTACAGAGACCGGAAAAGAAGCGAAAGTAGAACAAGTTAAACCAGTGGAAGGGAAGCTAAACTTA------------------------------------------------------------------------------------------------------------------------------------------------------------------------------------------------------------------------------------------------------------------------------------------------------------------------------------------------------------------------------------------------------------------------------------------------------------------------------GGAAAAGGAAAGGGCAAAGGAAAGAAGGTTAGAAAGGGAAAGAAAGGACGAAAGGGAAAGAAAGGACGAAAGGGAAAGAAAGGACGGAAAGGAAAGAAGGGGCGAAAAGGAAAGAAAAGAAGAAAAGGAAAAAAAGGACGAAAGGGAAAGAAAGGACGAAAGGGAAAGAAAGGACGAAAGGGCAAGAAAGGACGAAAGGGAAAGAAAGGACGAAAGGGAAAGAAAGGACGAAAGGGAAAGAAAGGACGAANGGGAAAGAAAGGA------------------CGAAAGGGCAAGAAAGGAAATCCAATCAAAACTGAAGAAACACCAGCCATTCTGACGGAGATAAAAGCTGCAGAATTAGAAAAGGAACCAAAAACAGAAATAGTTATGAAACCAGTTATTCCCGAAACGGAACTAACTGAGACCGGAAAAGAAGCAGAAGTAGAACAAGTTAAACCAGTGGAAAGGAAGCTAAAAATAGGAAAAGGAAAGGGCAAAGGAAAGAAGGTTAGAAAGGGAAAGAAAGGACGAAAGGGAAAGAAAGGACGAAAGGGAAAGAAAGGACGGAAAGGAAAGAAGGGGCGAAAAGGAAAGAAAAGAAGAAAAGGAAAGAAAAGACGAAAAGGAAAGAAACGACGAAAGGGAAAGAAAGGACGAAAGGGAAAAAAAGGACGAAAGGGAAAGAAAGGACGAAAGGGAAAAAAAGGACGAAAGGGAAAAAAAGGACGAAAGGGAAAGAAAGGACGAAAGGGAAAGAAAGGCAAACCAATCCAAACTGAAGAAACACCAGCCATTCCGACGGAGATAAAAGCTACAGAAATAGAAAAGGAACCAAAAACAGAAGTGGTTGTGGAACCACTTATTCAGGAAAAAGATGTTGCGGAGACCGAAATACAACCCATCGAAGCAGAAGTAGAACCAGTTGAACCAAAGACAGAAATAGTTATGGAACCAGTTATTCCGGAAGCAGATATTGCGGAGACCGAAATACAACCCATCAAAGCAGAAGAAGAACCAGTT------------------------------------------------------------------------------------------------------------GAACCAAAGCCAGAAATAGTTATGGAACCAGATGAGGAACCAGTTATTCCGGAAGCAGATATTGCGGAGACCGAAATACAACCCATCAAAGCAGAAGAAGAACCAGTTGAACCAAAGCCAGAAATAGTTATGGAACCAGATGAGGAACCAGTTATTCCGGAAGCAGATATTGCGGAGACCGAAATACAACCCATCAAAGCAGAAGAAGAACCAGTTGAACCAAAGCCAGAAATAGTTATGGAACCAGATGAGGAACCAGTTATTCCGGAAGCAGATATTGCGGAGACCGAAATACAACCCATCAGAGCAGAAGAAGAACCAGTTGAACCAAAGCCAGAAATAGTTATGGAACCAGATGAGGAACCAGTCATTCCAGAAACAGAACTAACAGAGACAGACAAACAACCAATCGAAGCAGAAGTAGAACCAGTTGAACCAAAAACAGAAGTGGTTGTGGAACCAGTTATTCCGGAAGCAGATATTGCGGAGACCGAAATACAACCCATCGAAGCAGAAGTAGAACCAGTTGAACCAAAGCCAGAAATAGTTATGGAACCAGATGTGGAACCAGTAATTCCAGAAACAGATCTTACAGAGACCGAAAAACAACCAATCGAAGCAGAAGTAGAACCAGTTGAACCGAAGACAGAGATAGTCGAACCAGAAACAGAGCCAGTCGAAGAAGCCGAGGAAGAAGCGGTTGAAGCACCCGTTATGGAACCAGTTATACCTGAGATAGAACCAAAGACAGAACCGGAAGTTGAAGCCGAGGAAAAAGAAGTCGAAACAAACGTTGAACCAGAACTACTGGAGACAGGAAAACCGATTGAAATTGGCCAAGCAACTGAAATCGAGGAGCCACCAGAAAAACAACCGGTGGAATCGGAGTCACAACCAGCGGAAGTTGAGATGGAACAAATTGAAACAAAGGAAGAACAAACTGAACCAAAGGAAGAACTAGCAGGAATTGATGAGAAATGTAAGTTTTAGTAACAATTATTTATTTTATGATTCCTTTTGAGCTGTTTCGGTATGCTTCGATGCAAGTCAAATTTGTGGATGTTTTAAATCCATCATTCAATTATGTTTGCAATGTAGTCATGAAGTTTGTTCGTTTGTTCAACACAGATATGGGACATATACGCATGAGCAGACCTTTTTTGTGCACAGATCTTCGTTCAGGTTAATCGTGCATGGCAACAAAAATTGGTTCATTCTGTTTTATGTCGTGCAGTCTTATTTTGCTTGAATTAAGCATTGCTGTAAAGTTCAAGTTAAAGGTACAGTCCATCATTTGTAATTTGTGCATTTTGTTCGTTTGAAGAAACAAACATGCTCAAAATCTAAAGAAGGCTGCTGAACAACTAACCTG-GGCCCAATTTCATAGCACTGCTTAACAGTAAGCAGAAAAGTTGTGCTTACTATAGCAGAAAATCATGCTGCTGCTTAAGCACTACTTCATGCCGTGAATGTGCTTACAGTTGCGCGCGCATGGAGATTTCTATTGTTACGTCACTGATTTAAGCAAAGTTTTCTTCTGGGGTTAAGCGTGCTTTTGCTGTGCTTACTGCTTAACAGTCCCTATGAAATAGATGGAGGCTCGGTAAGCACAAAGTCGGCTGTTAAGCAGCGCTATGAAATTGGGCCCTGATGAAGTTTCAGCTCAGCTTCAGCTGTATAAATTATTTCGAGAAAAACAAAATTCTAGGATCTCCATTTCAAATCAAAAGTTGCTGAATCTTGTTTTGAATTGGGCCTGAATTCAGCGTCCGAGGCACGTTCTCATCACTGAGCCGATTGCAGCTACGCGATGTCGCGCACGAATTAAAGCAATCGAGGGGCGCGTGTGCGTTGTGTCACGCTTGCGCGCGCTTCNCCAAATAAAAAAAAAAGAACTAAACGGAAATTTCTTTAGACGCATGTCTTTAGACGCACCGTTGGAAAGGCCTACTTTCGTTGCTTTCTTCAAAATGACAGCATGCCAGAAAGAAAAATCTCTTATCTTTCAACGAAGTATGTTTTTTAACAATAGGCCTAATTACGTCAGTTACTTTTTGGATAGTTGCAAATAATGATGGACTGTAGCTTTAAGAAATTTGTGGAACTCAAGTTGTGCTTGGCAAGTTTTACTTTAGAATTCGAAAATGCATCGACACCAAAAAAAAGTGTAATTTTGGTCCTATAGTTGAAGTGTCAAACTAGAACAAATTTAATGATAAATTAAATAAATTTAAATAATTTATTCCTACCTTGCTTTGACAGTGATGAAGGAGCTGCGTGACCTTTTGGAATCTACGAAGATTGACCTTCCTGTTGACATCAATGATCCATACGACCTAGGTCTTCTTCTCAGACATTTACGTCACCATTCAAATCTTCTTGCTCGTATTGGAGACCCCGATGTCAAAAAGGAAGTCCTCAGCGCCATGAATGAAAAC

>SS17-B

GCACGACGTGCCGCAGAGGAAAAACCTCAAAAGGGAAAGAAGGGACGAAAAGGAAAGAAACGACGAAAGGGAAAGAAGGGACGAAAAGGAAAGAAGGGACGAAAAGGAAAGAAACGACGAAAGGGAAAGAAAAGACGAAAGGGAAAGAAAGGACGAAAGGGAAAGAAAGGACGAAAGGGAAAGAAAGGACGAAAGGGAAAGAAAGGACGAAAGGGAAAGAAAGGACGAAAGGGAAAGAAAGGACGAAAGGGAAAGAAAGGACGAAAGGGAAAGAAAGGAAAACCAATCCAACCTGAAGAAACACCAGCCATTCCGACGGAGATAAAAGCTGCAGAAATAGAAAAGGAACCAAAAACAGAAGTGGTTGTGGAACCAGTTATTCCGGAAGCAGATATTGCGGAGACCGAAATACAACAAATTAAAGCAGAAGTAGAACCAGTTGAAACAAAGCCAGAAATAGTTATGGAACCAGATGTGGAACCAGTTATTCCAGAAACGGAACTTACAGAGACCGGAAAAGAAGCGAAAGTAGAACAAGTTAAACCAGTGGAAGGGAAGCTAAACTTA------------------------------------------------------------------------------------------------------------------------------------------------------------------------------------------------------------------------------------------------------------------------------------------------------------------------------------------------------------------------------------------------------------------------------------------------------------------------------GGAAAAGGAAAGGGCAAAGGAAAGAAGGTTAGAAAGGGAAAGAAAGGACGAAAGGGAAAGAAAGGACGAAAGGGAAAGAAAGGACGGAAAGGAAAGAAGGGGCGAAAAGGAAAGAAAAGAAGAAAAGGAAAAAAAGGACGAAAGGGAAAGAAAGGACGAAAGGGAAAGAAAGGACGAAAGGGCAAGAAAGGACGAAAGGGAAAGAAAGGACGAAAGGGAAAGAAAGGACGAAAGGGAAAGAAAGGACGAAGGGGAAAGAAAGGA------------------CGAAAGGGCAAGAAAGGAAATCCAATCAAAACTGAAGAAACACCAGCCATTCTGACGGAGATAAAAGCTGCAGAATTAGAAAAGGAACCAAAAACAGAAATAGTTATGAAACCAGTTATTCCCGAAACGGAACTAACTGAGACCGGAAAAGAAGCAGAAGTAGAACAAGTTAAACCAGTGGAAAGGAAGCTAAAAATAGGAAAAGGAAAGGGCAAAGGAAAGAAGGTTAGAAAGGGAAAGAAAGGACGAAAGGGAAAGAAAGGACGAAAGGGAAAGAAAGGGCGGAAAGGAAAGAAGGGGCGAAAAGGAAAGAAAAGAAGAAAAGGAAAGAAAAGACGAAAAGGAAAGAAACGACGAAAGGGAAAGAAAGGACGAAAGGGAAAAAAAGGACGAAAGGGAAAGAAAGGACGAAAGGGAAAAAAAGGACGAAAGGGAAAAAAAGGACGAAAGGGAAAGAAAGGACGAAAGGGAAAGAAAGGCAAACCAATCCAAACTGAAGAAACACCAGCCATTCCGACGGAGATAAAAGCTACAGAAATAGAAAAGGAACCAAAAACAGAAGTGGTTGTGGAACCACTTATTCAGGAAAAAGATGTTGCGGAGACCGAAATACAACCCATCGAAGCAGAAGTAGAACCAGTTGAACCAAAGACAGAAATAGTTATGGAACCAGTTATTCCGGAAGCAGATATTGCGGAGACCGAAATACAACCCATCAAAGCAGAAGAAGAACCAGTT------------------------------------------------------------------------------------------------------------GAACCAAAGCCAGAAATAGTTATGGAACCAGATGAGGAACCAGTTATTCCGGAAGCAGATATTGCGGAGACCGAAATACAACCCATCAAAGCAGAAGAAGAACCAGTTGAACCAAAGCCAGAAATAGTTATGGAACCAGATGAGGAACCAGTTATTCCGGAAGCAGATATTGCGGAGACCGAAATACAACCCATCAAAGCAGAAGAAGAACCAGTTGAACCAAAGCCAGAAATAGTTATGGAACCAGATGAGGAACCAGTTATTCCGGAAGCAGATATTGCGGAGACCGAAATACAACCCATCAGAGCAGAAGAAGAACCAGTTGAACCAAAGCCAGAAATAGTTATGGAACCAGATGAGGAACCAGTCATTCCAGAAACAGAACTAACAGAGACAGACAAACAACCAATCGAAGCAGAAGTAGAACCAGTTGAACCAAAAACAGAAGTGGTTGTGGAACCAGTTATTCCGGAAGCAGATATTGCGGAGACCGAAATACAACCCATCGAAGCAGAAGTAGAACCAGTTGAACCAAAGCCAGAAATAGTTATGGAACCAGATGTGGAACCAGTAATTCCAGAAACAGATCTTACAGAGACCGAAAAACAACCAATCGAAGCAGAAGTAGAACCAGTTGAACCGAAGACAGAGATAGTCGAACCAGAAACAGAGCCAGTCGAAGAAGCCGAGGAAGAAGCGGTTGAAGCACCCGTTATGGAACCAGTTATACCTGAGATAGAACCAAAGACAGAACCGGAAGTTGAAGCCGAGGAAAAAGAAGTCGAAACAAACGTTGAACCAGAACTACTGGAGACAGGAAAACCGATTGAAATTGGCCAAGCAACTGAAATCGAGGAGCCACCAGAAAAACAACCGGTGGAATCGGAGTCACAACCAGCGGAAGTTGAGATGGAACAAATTGAAACAAAGGAAGAACAAACTGAACCAAAGGAAGAACTAGCAGGAATTGATGAGAAATGTAAGTTTTAGTAACAATTATTTATTTTATGATTCCTTTTGAGCTGTTTCGGTATGCTTCGATGCAAGTCAAATTTGTGGATGTTTTAAATCCATCATTCAATTATGTTTGCAATGTAGTCATGAAGTTTGTTCGTTTGTTCAACACAGATATGGGACATATACGCATGAGCAGACCTTTTTTGTGCACAGATCTTCGTTCAGGTTAATCGTGCATGGCAACAAAAATTGGTTCATTCTGTTTTATGTCGTGCAGTCTTATTTTGCTTGAATTAAGCATTGCTGTAAAGTTCAAGTTAAAGGTACAGTCCATCATTTGTAATTTGTGCATTTTGTTCGTTTGAAGAAACAAACATGCTCAAAATCTAAAGAAGGCTGCTGAACAACTAACCTG-GGCCCAATTTCATAGCACTGCTTAACAGTAAGCAGAAAAGTTGTGCTTACTATAGCAGAAAATCATGCTGCTGCTTAAGCACTACTTCATGCCGTGAATGTGCTTACAGTTGCGCGCGCATGGAGATTTCTATTGTTACGTCACTGATTTAAGCAAAGTTTTCTTCTGGGGTTAAGCGTGCTTTTACTGTGCTTACTGCTTAACAGTCCCTATGAAATAGATGGAGGCTCAGTAAGCACAAAGTCGGCTGTTAAGCAGCGCTATGAAATTGGGCCCTGATGAAGTTTCAGCTCAGCTTCAGCTGTATAAATTATTTCGAGAAAAACAAAATTCTAGGATCTCCATTTCAAATCAAAAGTTGCTGAATCTTGTTTTGAATCGGGCCTGAATTCAGCGTCCGAGGCACGTTCTCATCACTGAGCCGATTGCAGCTACGCGATGTCGCGCACGAATTAAAGCAATCGAGGGCGCGTGTGCGTTGTGTCAACGCTTGCGCGCGCTTCGCCAAAT-AAAAAAAAAGAACTAAACGGAAATTTCTTTAGACGCATGTCTTTAGACGCACCGTTGGAAAGGCCTACTTTCGTTGCTTTCTTCAAAATGACAGCATGCCAGAAAGAAAAATCTCTTATCTTTCAACGAAGTATGTTTTTTAACAATAGGCCTAATTACGTCAGTTACTTTTTGGATAGTTGCAAATAATGATGGACTGTAGCTTTAAGAAATTTGTGGAACTCAAGTTGTGCTTGGCAAGTTTTACTTTAGAATTCGAAAATGCATCGACACCAAAAAAAAGTGTAATTTTGGTCCTATAGTTGAAGTGTCAAACTAGAACAAATTTAATGATAAATTAAATAAATTTAAATAATTTATTCCTACCTTGCTTTGACAGTGATGAAGGAGCTGCGTGACCTTTTGGAATCTACGAAGATTGACCTTCCTGTTGACATCAATGATCCATACGACCTAGGTCTTCTTCTCAGACATTTACGTCACCATTCAAATCTTCTTGCTCGTATTGGAGACCCCGATGTCAAAAAGGAAGTCCTCAGCGCCATGAATGAAAAC

>SS04-A

GCACGACGTGCCGCAGAGGAAAAACCTCAAAAGGGAAAGAAGGGACGAAAAGGAAAGAAACGACGAAAGGGAAAGAAGGGACGAAAAGGAAAGAAGGGACGAAAAGGAAAGAAACGACGAAAGGGAAAGAAAAGACGAAAGGGAAAGAAAGGACGAAAGGGAAAGAAAGGACGAAAGGGAAAGAAAGGACGAAAGGGAAAGAAAGGACGAAAGGGAAAGAAAGGACGAAAGGGAAAGAAAGGACGAAAGGGAAAGAAAGGACGAAAGGGAAAGAAAGGAAAACCAATCCAACCTGAAGAAACACCAGCCATTCCGACGGAGATAAAAGCTGCAGAAATAGAAAAGGAACCAAAAACAGAAGTGGTTGTGGAACCAGTTATTCCGGAAGCAGATATTGCGGAGACCGAAATACAACAAATTAAAGCAGAAGTAGAACCAGTTGAAACAAAGCCAGAAATAGTTATGGAACCAGATGTGGAACCAGTTATTCCAGAAACGGAACTTACAGAGACCGGAAAAGAAGCGAAAGTAGAACAAGTTAAACCAGTGGAAGGGAAGCTAAACTTA------------------------------------------------------------------------------------------------------------------------------------------------------------------------------------------------------------------------------------------------------------------------------------------------------------------------------------------------------------------------------------------------------------------------------------------------------------------------------GGAAAAGGAAAGGGCAAAGGAAAGAAGGTTAGAAAGGGAAAGAAAGGACGAAAGGGAAAGAAAGGACGAAAGGGAAAGAAAGGACGGAAAGGAAAGAAGGGGCGAAAAGGAAAGAAAAGAAGAAAAGGAAAAAAAGGACGAAAGGGAAAGAAAGGACGAAAGGGAAAGAAAGGACGAAAGGGCAAGAAAGGACGAAAGGGAAAGAAAGGACGAAAGGGAAAGAAAGGACGAAAGGGAAAGAAAGGACGAAGGGGAAAGAAAGGA------------------CGAAAGGGCAAGAAAGGAAATCCAATCAAAACTGAAGAAACACCAGCCATTCTGACGGAGATAAAAGCTGCAGAATTAGAAAAGGAACCAAAAACAGAAATAGTTATGAAACCAGTTATTCCCGAAACGGAACTAACAGAGACCGGAAAAGAAGCAGAAGTAGAACAAGTTAAACCAGTGGAAAGGAAGCTAAAAATAGGAAAAGGAAAGGGCAAAGGAAAGAAGGTTAGAAAGGGAAAGAAAGGACGAAAGGGAAAGAAAGGACGAAAGGGAAAGAAAGGACGGAAAGGAAAGAAGGGGCGAAAAGGAAAGAAAAGAAGAAAAGGAAAGAAAAGACGAAAAGGAAAGAAACGACGAAAGGGAAAGAAAGGACGAAAGGGAAAAAAAGGACGAAAGGGAAAGAAAGGACGAAAGGGAAAAAAAGGACGAAAGGGAAAAAAAGGACGAAAGGGAAAGAAAGGACGAAAGGGAAAGAAAGGCAAACCAATCCAAACTGAAGAAACACCAGCCATTCCGACGGAGATAAAAGCTGCAGAAATAGAAAAGGAACCAAAAACAGAAGTGGTTGTGGAACCACTTATTCTGGAAAAAGATGTTGCGGAGACCGAAATACAACCCATCGAAGCAGAAGTAGAACCAGTTGAACCAAAGACAGAAATAGTTATGGAACCAGTTATTCCGGAAGCAGATATTGCGGAGACCGAAATACAACCCATCAAAGCAGAAGAAGAACCAGTT------------------------------------------------------------------------------------------------------------GAACCAAAGCCAGAAATAGTTATGGAACCAGATGAGGAACCAGTTATTCCGGAAGCAGATATTGCGGAGACCGAAATACAACCCATCAAAGCAGAAGAAGAACCAGTTGAACCAAAGCCAGAAATAGTTATGGAACCAGATGAGGAACCAGTTATTCCGGAAGCAGATATTGCGGAGACCGAAATACAACCCATCAAAGCAGAAGAAGAACCAGTTGAACCAAAGCCAGAAATAGTTATGGAACCAGATGAGGAACCAGTTATTCCGGAAGCAGATATTGCGGAGACCGAAATACAACCCATCAGAGCAGAAGAAGAACCAGTTGAACCAAAGCCAGAAATAGTTATGGAACCAGATGAGGAACCAGTCATTCCAGAAACAGAACTAACAGAGACAGACAAACAACCAATCGAAGCAGAAGTAGAACCAGTTGAACCAAAAACAGAAGTGGTTGTGGAACCAGTTATTCCGGAAGCAGATATTGCGGAGACCGAAATACAACCCATCGAAGCAGAAGTAGAACCAGTTGAACCAAAGCCAGAAATAGTTATGGAACCAGATGTGGAACCAGTAATTCCAGAAACAGATCTTACAGAGACCGAAAAACAACCAATCGAAGCAGAAGTAGAACCAGTTGAACCGAAGACAGAGATAGTCGAACCAGAAACAGAGCCAGTCGAAGAAGCCGAGGAAGAAGCGGTTGAAGCACCCGTTATGGAACCAGTTATACCTGAGATAGAACCAAAGACAGAACCGGAAGTTGAAGCCGAGGAAAAAGAAGTCGAAACAAACGTTGAACCAGAACTACTGGAGACAGGAAAACCGATTGAAATTGGCCAAGCAACTGAAATCGAGGAGCCACCAGAAAAACAACCGGTGGAATCGGAGTCACAACCAGCGGAAGTTGAGATGGAACAAATTGAAACAAAGGAAGAACAAACTGAACCAAAGGAAGAACTAGCAGGAATTGATGAGAAATGTAAGTNTTAGTAACAATTATTTATTTTATGATTCCTTTTGAGCTGTTTCGGTATGCTTCGATGCAAGTCAAATTTGTGGATGTTTTAAATCCATCATTCAATTATGTTTGCAATGTAGTCATGAAGTTTGTTCGTTTGTTCAACACAGATATGGGACATATACGCATGAGCAGACCTTTTTTGTGCACAGATCTTCGTTCAGGTTAATCGTGCATGGCAACAAAAATTGGTTCATTCCGTTTTATGTCGTGCAGTCTTATTTTGCTTGAATTAAGCATTGCTGCAAAGTTCAAGTTAAAGGTACAGTCCATCATTTGTAATTTGTGCATTTTGTTCGTTTGAAGAAACAAACATGCTCAAAATCTAAAGAAGGCTGCTAAACAACTAACCT---------------------------------------------------------------------------------------------------------------------------------------------------------------------------------------------------------------------------------------------------------------------------------------GATGAAGTTTCAGCTCAGCTTCAGCTGTATAAATTATTTCGAGAAAAACAAAATTCTAGGATCTCCATTTCAAATCAAAAGTTGCTGAATCTTGTTTTGAATTGGGCCTGAATTCAGCGTCCGAGGCACGTTCTCATCACTGAGCCGATTGCAGCTACGCGATGTCGCGCACGAATTAAAGCAATCGAGGGCGCGTGTGCGTTGTGTCAACGCTTGCGCGCGCTTCGCCAAAT---AAAAAAAGAACTAAACGGAAATTTCTTTAGACGCATGTCTTTAGACGCACCGTTGGAAAGGCCTACTTTCGTTGCTTTCTTCAAAATGACAGCATGCCAGAAAGAAAAATCTCTTATCTTTCAACGAAGTATGTTTTTTAACAATAGGCCTAATTACGTCAGTTACTTTTTGGATAGTTGCAAATAATGATGGACTGTAGCTTTAAGAAATTTGGGGAACTCAAGTTGTGCTTGGCAAGTTTTACTTTAGAATTCGAAAATGCATCGACACCAAAAAAAAGTGTAATTTCGGTCCTATAGTTGAAGTGTCAAACTAGAACAAATTTAATGATAAATTAAATAAATTTAAATAATTTATTCCTACCTTGCTTTGACAGTGATGAAGGAGCTGCGTGACCTTTTGGAATCTACGAAGATTGACCTTCCTGTTGACATCAATGATCCATACGACCTAGGTCTTCTTCTCAGACATTTACGTCACCATTCAAATCTTCTTGCTCGTATTGGAGACCCCGATGTCAAAAAGGAAGTCCTCAGCGCCATGAATGAAAAC

>SS04-B

GCACGACGTGCCGCAGAGGAAAAACCTCAAAAGGGAAAGAAGGGACGAAAAGGAAAGAAACGACGAAAGGGAAAGAAGGGACGAAAAGGAAAGAAGGGACGAAAAGGAAAGGAACGACGAAAGGGAAAGAAAAGACGAAAGGGAAAGAAAGGACGAAAGGGAAAGAAAGGACGAAAGGGAAAGAAAGGACGAAAGGGAAAGAAAGGACGAAAGGGAAAGAAAGGACGAAAGGGAAAGAAAGGACGAAAGGGAAAGAAAGGACGAAAGGGAAAGAAAGGAAAACCAATCCAACCTGAAGAAACACCAGCCATTCCGACGGAGATAAAAGCTGCAGAAATAGAAAAGGAACCAAAAACAGAAGTGGTTGTGGAACCAGTTATTCCGGAAGCAGATATTGCGGAGACCGAAATACAACAAATTAAAGCAGAAGTAGAACCAGTTGAAACAAAGCCAGAAATAGTTATGGAACCAGATGTGGAACCAGTTATTCCAGAAACGGAACTTACAGAGACCGGAAAAGAAGCGAAAGTAGAACAAGTTAAACCAGTGGAAGGGAAGCTAAACTTA------------------------------------------------------------------------------------------------------------------------------------------------------------------------------------------------------------------------------------------------------------------------------------------------------------------------------------------------------------------------------------------------------------------------------------------------------------------------------GGAAAAGGAAAGGGCAAAGGAAAGAAGGTTAGAAAGGGAAAGAAAGGACGAAAGGGAAAGAAAGGACGAAAGGGAAAGAAAGGACGGAAAGGAAAGAAGGGGCGAAAAGGAAAGAAAAGAAGAAAAGGAAAAAAAGGACGAAAGGGAAAGAAAGGACGAAAGGGAAAGAAAGGACGAAAGGGCAAGAAAGGACGAAAGGGAAAGAAAGGACGAAAGGGAAAGAAAGGACGAAAGGGAAAGAAAGGACGAAGGGGAAAGAAAGGA------------------CGAAAGGGCAAGAAAGGAAATCCAATCAAAACTGAAGAAACACCAGCCATTCTGACGGAGATAAAAGCTGCAGAATTAGAAAAGGAACCAAAAACAGAAATAGTTATGAAACCAGTTATTCCCGAAACGGAACTAACAGAGACCGGAAAAGAAGCAGAAGTAGAACAAGTTAAACCAGTGGAAAGGAAGCTAAAAATAGGAAAAGGAAAGGGCAAAGGAAAGAAGGTTAGAAAGGGAAAGAAAGGACGAAAGGGAAAGAAAGGACGAAAGGGAAAGAAAGGACGGAAAGGAAAGAAGGGGCGAAAAGGAAAGAAAAGAAGAAAAGGAAAGAAAAGACGAAAAGGAAAGAAACGACGAAAGGGAAAGAAAGGACGAAAGGGAAAAAAAGGACGAAAGGGAAAGAAAGGACGAAAGGGAAAAAAAGGACGAAAGGGAAAAAAAGGACGAAAGGGAAAGAAAGGACGAAAGGGAAAGAAAGGCAAACCAATCCAAACTGAAGAAACACCAGCCATTCCGACGGAGATAAAAGCTGCAGAAATAGAAAAGGAACCAAAAACAGAAGTGGTTGTGGAACCACTTATTCAGGAAAAAGATGTTGCGGAGACCGAAATACAACCCATCGAAGCAGAAGTAGAACCAGTTGAACCAAAGACAGAAATAGTTATGGAACCAGTTATTCCGGAAGCAGATATTGCGGAGACCGAAATACAACCCATCAAAGCAGAAGAAGAACCAGTT------------------------------------------------------------------------------------------------------------GAACCAAAGCCAGAAATAGTTATGGAACCAGATGAGGAACCAGTTATTCCGGAAGCAGATATTGCGGAGACCGAAATACAACCCATCAAAGCAGAAGNAGAACCAGTTGAACCAAAGCCAGAAATAGTTATGGAACCAGATGAGGAACCAGTTATTCCGGAAGCAGATATTGCGGAGACCGAAATACAACCCATCAAAGCAGAAGAAGAACCAGTTGAACCAAAGCCAGAAATAGTTATGGAACCAGATGAGGAACCAGTTATTCCGGAAGCAGATATTGCGGAGACCGAAATACAACCCATCAGAGCAGAAGAAGAACCAGTTGAACCAAAGCCAGAAATAGTTATGGAACCAGATGAGGAACCAGTCATTCCAGAAACAGAACTAACAGAGACAGACAAACAACCAATCGAAGCAGAAGTAGAACCAGTTGAACCAAAAACAGAAGTGGTTGTGGAACCAGTTATTCCGGAAGCAGATATTGCGGAGACCGAAATACAACCCATCGAAGCAGAAGTAGAACCAGTTGAACCAAAGCCAGAAATAGTTATGGAACCAGATGTGGAACCAGTAATTCCAGAAACAGATCTTACAGAGACCGAAAAACAACCAATCGAAGCAGAAGTAGAACCAGTTGAACCGAAGACAGAGATAGTCGAACCAGAAACAGAGCCAGTCGAAGAAGCCGAGGAAGAAGCGGTTGAAGCACCCGTTATGGAACCAGTTATACCTGAGATAGAACCAAAGACAGAACCGGAAGTTGAAGCCGAGGAAAAAGAAGTCGAAACAAACGTTGAACCAGAACTACTGGAGACAGGAAAACCGATTGAAATTGGCCAAGCAACTGAAATCGAGGAGCCACCAGAAAAACAACCGGTGGAATCGGAGTCACAACCAGCGGAAGTTGAGATGGAACAAATTGAAACAAAGGAAGAACAAACTGAACCAAAGGAAGAACTAGCAGGAATTGATGAGAAATGTAAGTTTTAGTAACAATTATTTATTTTATGATTCCTTTTGAGCTGTTTCGGTATGCTTCGATGCAAGTCAAATTTGTGGATGTTTTAAATCCATCATTCAATTATGTTTGCAATGTAGTCATGAAGTTTGTTCGTTTGTTCAACACAGATATGGGACATATACGCATGAGCAGACCTTTTTTGTGCACAGATCTTCGTTCAGGTTAATCGTGCATGGCAACAAAAATTGGTTCATTCTGTTTTATGTCGTGCAGTCTTATTTTGCTTGAATTAAGCATTGCTGTAAAGTTCAAGTTAAAGGTACAGTCCATCATTTGTAATTTGTGCATTTTGTTCGTTTGAAGAAACAAACATGCTCAAAATCTAAAGAAGGCTGCTGAACAACTAACCT---------------------------------------------------------------------------------------------------------------------------------------------------------------------------------------------------------------------------------------------------------------------------------------GATGAAGTTTCAGCTCAGCTTCAGCTGTATAAATTATTTCGAGAAAAACAAAATTCTAGGATCTCCATTTCAAATCAAAAGTTGCTGAATCTTGTTTTGAATTGGGCCTGAATTCAGCGTCCGAGGCACGTTCTCATCACTGAGCCGATTGCAGCTACGCGATGTCGCGCACGAATTAAAGCAATCGAGGGCGCGTGTGCGTTGTGTCAACGCTTGCGCACGCTTCGCCAAATAAAAAAAAAAGAACTAAACGGAAATTTCTTTAGACGCATGTCTTTAGACGCACCGTTGGAAGGGCCTACTTTCGTTGCTTTCTTCAAAATGACAGCATGCCAGAAAGAAAAATCTCTTATCTTTCAACGAAGTATGTTTTTTAACAATAGGNCTAATTACGTCAGTTACTTTTTGGATAGTTGCAAATAATGATGGACTGTAGCTTTAAGAAATTTGTGGAACTCAAGTTGTGCTTGGCAAGTTTTACTTTAGAATTCGAAAATGCATCGACACCAAAAAAAAGTGTAATTTTGGTCCTATAGTTGAAGTGTCAAACTAGAACAAATTTAATGATAAATTAAATAAATTTAAATAATTTATTCCTACCTTGCTTTGACAGTGATGAAGGAGCTGCGTGACCTTTTGGAATCTACGAAGATTGACCTTCCTGTTGACATCAATGATCCATACGACCTAGGTCTTCTTCTCAGACATTTACGTCACCATTCAAATCTTCTTGCTCGTATTGGAGACCCCGATGTCAAAAAGGAAGTCCTCAGCGCCATGAATGAAAAC

>SS93-B

GCACGACGTGCCGCAGAGGAAAAACCTCAAAAGGGAAAGAAGGGACGAAAAGGAAAGAAACGACGAAAGGGAAAGAAGGGACGAAAAGGAAAGAAGGGACGAAAAGGAAAGAAACGACGAAAGGGAAAGAAAAGACGAAAGGGAAAGAAAGGACGAAAGGGAAAGAAAGGACGAAAGGGAAAGAAAGGACGAAAGGGAAAGAAAGGACGAAAGGGAAAGAAAGGACGAAAGGGAAAGAAAGGACGAAAGGGAAAGAAAGGACGAAAGGGAAAGAAAGGAAAACCAATCCAACCTGAAGAAACACCAGCCATTCCGACGGAGATAAAAGCTGCAGAAATAGAAAAGGAACCAAAAACAGAAGTGGTTGTGGAACCAGTTATTCCGGAAGCAGATATTGCGGAGACCGAAATACAACAAATTAAAGCAGAAGTAGAACCAGTTGAAACAAAGCCAGAAATAGTTATGGAACCAGATGTGGAACCAGTTATTCCAGAAACGGAACTTACAGAGACCGGAAAAGAAGCGAAAGTAGAACAAGTTAAACCAGTGGAAGGGAAGCTAAACTTA------------------------------------------------------------------------------------------------------------------------------------------------------------------------------------------------------------------------------------------------------------------------------------------------------------------------------------------------------------------------------------------------------------------------------------------------------------------------------GGAAAAGGAAAGGGCAAAGGAAAGAAGGTTAGAAAGGGAAAGAAAGGACGAAAGGGAAAGAAAGGACGAAAGGGAAAGAAAGGACGGAAAGGAAAGAAGGGGCGAAAAGGAAAGAAAAGAAGAAAAGGAAAAAAAGGACGAAAGGGAAAGAAAGGACGAAAGGGAAAGAAAGGACGAAAGGGCAAGAAAGGACGAAAGGGAAAGAAAGGACGAAAGGGAAAGAAAGGACGAAAGGGAAAGAAAGGACGAAGGGGAAAGAAAGGA------------------CGAAAGGGCAAGAAAGGAAATCCAATCAAAACTGAAGAAACACCAGCCATTCTGACGGAGATAAAAGCTGCAGAATTAGAAAAGGAACCAAAAACAGAAATAGTTATGAAACCAGTTATTCCCGAAACGGAACTAACTGAGACCGGAAAAGAAGCAGAAGTAGAACAAGTTAAACCAGTGGAAAGGAAGCTAAAAATAGGAAAAGGAAAGGGCAAAGGAAAGAAGGTTAGAAAGGGAAAGAAAGGACGAAAGGGAAAGAAAGGACGAAAGGGAAAGAAAGGACGGAAAGGAAAGAAGGGGCGAAAAGGAAAGAAAAGAAGAAAAGGAAAGAAAAGACGAAAAGGAAAGAAACGACGAAAGGGAAAGAAAGGACGAAAGGGAAAAAAAGGACGAAAGGGAAAGAAAGGACGAAAGGGAAAAAAAGGACGAAAGGGAAAAAAAGGACGAAAGGGAAAGAAAGGACGAAAGGGAAAGAAAGGCAAACCAATCCAAACTGAAGAAACACCAGCCATTCCGACGGAGATAAAAGCTGCAGAAATAGAAAAGGAACCAAAAACAGAAGTGGTTGTGGAACCACTTATTCAGGAAAAAGATGTTGCGGAGACCGAAATACAACCCATCGAAGCAGACGTAGAACCAGTTGAACCAAAGACAGAAATAGTTATGGAACCAGTTATTCCGGAAGCAGATATTGCGGAGACCGAAATACAACCCATCAAAGCAGAAGAAGAACCAGTT------------------------------------------------------------------------------------------------------------GAACCAAAGCCAGAAATAGTTATGGAACCAGATGAGGAACCAGTTATTCCGGAAGCAGATATTGCGGAGACCGAAATACAACCCATCAAAGCAGAAGAAGAACCAGTTGAACCAAAGCCAGAAATAGTTATGGAACCAGATGAGGAACCAGTTATTCCGGAAGCAGATATTGCGGAGACCGAAATACAACCCATCAAAGCAGAAGAAGAACCAGTTGAACCAAAGCCAGAAATAGTTATGGAACCAGATGAGGAACCAGTTATTCCGGAAGCAGATATTGCGGAGACCGAAATACAACCCATCAGAGCAGAAGAAGAACCAGTTGAACCAATGCCAGAAATAGTTATGGAACCAGATGAGGAACCAGTCATTCCAGAAACAGAACTAACAGAGACAGACAAACAACCAATCGAAGCAGAAGTAGAACCAGTTGAACCAAAAACAGAAGTGGTTGTGGAACCAGTTATTCCGGAAGCAGATATTGCGGAGACCGAAATACAACCCATCGAAGCAGAAGTAGAACCAGTTGAACCAAAGCCAGAAATAGTTATGGAACCAGATGTGGAACCAGTAATTCCAGAAACAGATCTTACAGAGACCGAAAAACAACCAATCGAAGCAGAAGTAGAACCAGTTGAACCGAAGACAGAGATAGTCGAACCAGAAACAGAGCCAGTCGAAGAAGCCGAGGAAGAAGCGGTTGAAGCACCCGTTATGGAACCAGTTATACCTGAGATAGAACCAAAGACAGAACCGGAAGTTGAAGCCGAGGAAAAAGAAGTCGAAACAAACGTTGAACCAGAACTACTGGAGACAGGAAAACCGATTGAAATTGGCCAAGCAACTGAAATCGAGGAGCCACCAGAAAAACAACCGGTGGAATCGGAGTCACAACCAGCGGAAGTTGAGATGGAACAAATTGAAACAAAGGAAGAACAAACTGAACCAAAGGAAGAACTAGCAGGAATTGATGAGAAATGTAAGTTTTAGTAACAATTATTTATTTTATGATTCCTTTTGAGCTGTTTCGGTATGCTTCGATGCAAGTCAAATTTGTGGATGTTTTAAATCCATCATTCAATTATGTTTGCAATGTAGTCATGAAGTTTGTTCGTTTGTTCAACACAGATATGGGACATATACGCATGAGCAGACCTTTTTTGTGCACAGATCTTCGTTCAGGTTAATCGTGCATGGCAACAAAAATTGGTTCATTCTGTTTTATGTCGTGCAGTCTTATTTTGCTTGAATTAAGCATTGCTGTAAAGTTCAAGTTAAAGGTACAGTCCATCATTTGTAATTTGTGCATTTTGTTCGTTTGAAGAAACAAACATGCTCAAAATCTAAAGAAGGCTGCTGAACAACCAACCT---------------------------------------------------------------------------------------------------------------------------------------------------------------------------------------------------------------------------------------------------------------------------------------GATGAAGTTTCAGCTCAGCTTCAGCTGTATAAATTATTTCGAGAAAAACAAAATTCTAGGATCTCCATTTCAAATCAAAAGTTGCTGAATCTTGTTTTGAATTGGGCCTGAATTCAGCGTCCGAGGCACGTTCTCATCACTGAGCCGATTGCAGCTACGCGATGTCGCGCACGAATTAAAGCAATCGAGGGCGCGTGTGCGTTGTGTCAACGCTTGCGCGCGCTTCGCCAAAT-AAAAAAAAAGAACTAAACGGAAATTTCTTTAGACGCATGTCTTTAGACGCACCGTTGGAAAGGCCTACTTTCGTTGCTTTCTTCAAAATGACAGCATGCCAGAAAGAAAAATCTCTTATCTTTCAACGAAGTATGTTTTCTAACAATAGGCCTAATTACGTCAGTTACTTTTTGGATAGTTGCAAATAATGATGGACTGTAGCTTTAAGAAATTTGTGGAACTCAAGTTGTGCTTGGCAAGTTTTACTTTAGAATTCGAAAATGCATCGACACCAAAAAAAAGTGTAATTTTGGTCCTATAGTTGAAGTGTCAAACTAGAACAAATTTAATGATAAATTAAATAAATTTAAATAATTTATTCCTACCTTGCTTTGACAGTGATGAAGGAGCTGCGTGACCTTTTGGAATCTACGAAGATTGACCTTCCTGTTGACATCAATGATCCATACGACCTAGGTCTTCTTCTCAGACATTTACGTCACCATTCAAATCTTCTTGCTCGTATTGGAGACCCCGATGTCAAAAAGGAAGTCCTCAGCGCCATGAATGAAAAC

>SS86-A

GCACGACGTGCCGCAGAGGAAAAACCTCAAAAGGGAAAGAAGGGACGAAAAGGAAAGAAACGACGAAAGGGAAAGAAGGGACGAAAAGGAAAGAAGGGACGAAAAGGAAAGAAACGACGAAAGGGAAAGAAAAGACGAAAGGGAAAGAAAGGACGAAAGGGAAAGAAAGGACGAAAGGGAAAGAAAGGACGAAAGGGAAAGAAAGGACGAAAGGGAAAGAAAGGACGAAAGGGAAAGAAAGGACGAAAGGGAAAGAAAGGA------------------AAACCAATCCAACCTGAAGAAACACCAGCCATTCCGACGGAGATAAAAGCTGCAGAAATAGAAAAGGAACCAAAAACAGAAGTGGTTGTGGAACCAGTTATTCCGGAAGCAGATATTGCGGAGACCGAAATACAACAAATTAAAGCAGAAGTAGAACCAGTTGAAACAAAGCCAGAAATAGTTATGGAACCAGATGTGGAACCAGTTATTCCAGAAACGGAACTTACAGAGACCGGAAAAGAAGCGAAAGTAGAACAAGTTAAACCAGTGGAAGGGAAGCTAAACTTA------------------------------------------------------------------------------------------------------------------------------------------------------------------------------------------------------------------------------------------------------------------------------------------------------------------------------------------------------------------------------------------------------------------------------------------------------------------------------GGAAAAGGAAAGGGCAAAGGAAAGAAGGTTAGAAAGGGAAAGAAAGGACGAAAGGGAAAGAAAGGACGAAAGGGAAAGAAAGGACGGAAAGGAAAGAAGGGGCGAAAAGGAAAGAAAAGAAGAAAAGGAAAAAAAGGACGAAAGGGAAAGAAAGGACGAAAGGGAAAGAAAGGACGAAAGGGCAAGAAAGGACGAAAGGGAAAGAAAGGACGAAAGGGAAAGAAAGGACGAAAGGGAAAGAAAGGACGAANGGGAAAGAAAGGA------------------CGAAAGGGCAAGAAAGGAAATCCAATCAAAACTGAAGAAACACCAGCCATTCTGACGGAGATAAAAGCTGCAGAATTAGAAAAGGAACCAAAAACAGAAATAGTTATGAAACCAGTTATTCCCGAAACGGAACTAACAGAGACCGGAAAAGAAGCAGAAGTAGAACAAGTTAAACCAGTGGAAAGGAAGCTAAAAATAGGAAAAGGAAAGGGCAAAGGAAAGAAGGTTAGAAAGGGAAAGAAAGGACGAAAGGGAAAGAAAGGACGAAAGGGAAAGAAAGGACGGAAAGGAAAGAAGGGGCGAAAAGGAAAGAAAAGAAGAAAAGGAAAGAAAAGACGAAAAGGAAAGAAACGACGAAAGGGAAAGAAAGGACGAAAGGGAAAAAAAGGACGAAAGGGAAAGAAAGGACGAAAGGGAAAAAAAGGACGAAAGGGAAAAAAAGGACGAAAGGGAAAGAAAGGACGAAAGGGAAAGAAAGGCAAACCAATCCAAACTGAAGAAACACCAGCCATTCCGACGGAGATAAAAGCTGCAGAAATAGAAAAGGAACCAAAAACAGAAGTGGTTGTGGAACCACTTATTCAGGAAAAAGATGTTGCGGAGACCGAAATACAACCCATCGAAGCAGAAGTAGAACCAGTTGAACCAAAGACAGAAATAGTTATGGAACCAGTTATTCCGGAAGCAGATATTGCGGAGACCGAAATACAACCCATCAAAGCAGAAGAAGAACCAGTT------------------------------------------------------------------------------------------------------------GAACCAAAGCCAGAAATAGTTATGGAACCAGATGAGGAACCAGTTATTCCGGAAGCAGATATTGCGGAGACCGAAATACAACCCATCAAAGCAGAAGAAGAACCAGTTGAACCAAAGCCAGAAATAGTTATGGAACCAGATGAGGAACCAGTTATTCCGGAAGCAGATATTGCGGAGACCGAAATACAACCCATCAAAGCAGAAGAAGAACCAGTTGAACCAAAGCCAGAAATAGTTATGGAACCAGATGAGGAACCAGTTATTCCGGAAGCAGATATTGCGGAGACCGAAATACAACCCATCAGAGCAGAAGAAGAACCAGTTGAACCAAAGCCAGAAATAGTTATGGAACCAGATGAGGAACCAGTCATTCCAGAAACAGAACTAACAGAGACAGACAAACAACCAATCGAAGCAGAAGTAGAACCAGTTGAACCAAAAACAGAAGTGGTTGTGGAACCAGTTATTCCGGAAGCAGATATTGCGGAGACCGAAATACAACCCATCGAAGCAGAAGTAGAACCAGTTGAACCAAAGCCAGAAATAGTTATGGAACCAGATGTGGAACCAGTAATTCCAGAAACAGATCTTACAGAGACCGAAAAACAACCAATCGAAGCAGAAGTAGAACCAGTTGAACCGAAGACAGAGATAGTCGAACCAGAAACAGAGCCAGTCGAAGAAGCCGAGGAAGAAGCGGTTGAAGCACCCGTTATGGAACCAGTTATACCTGAGATAGAACCAAAGACAGAACCGGAAGTTGAAGCCGAGGAAAAAGAAGTCGAAACAAACGTTGAACCAGAACTACTGGAGACAGGAAAACCGATTGAAATTGGCCAAGCAACTGAAATCGAGGAGCCACCAGAAAAACAACCGGTGGAATCGGAGTCACAACCAGCGGAAGTTGAGATGGAACAAATTGAAACAAAGGAAGAACAAACTGAACCAAAGGAAGAACTAGCAGGAATTGATGAGAAATGTAAGTTTTAGTAACAATTATTTATTTTATGATTCCTTTTGAGCTGTTTCGGTATGCTTCGATGCAAGTCAAATTTGTGGATGTTTTAAATCCCTCATTCAATTATGTTTGCAATGTAGTCATGAAGTTTGTTCGTTTGTTCAACACAGATATGGGACATATACGCATGAGCAGACCTTTTTTGTGCACAGATCTTCGTTCAGGTTAATCGTGCATGGCAACAAAAATTGGTTCATTCCGTTTTGTGTCGTGCAGTCTTATTTTGCTTGAATTAAGCATTGCTGTAAAGTTCAAGTTAAAGGTACAGTCCATCATTTGTAATTTGTGCATTTTGTTCGTTTGAAGAAACAAACATGCTCAAAATCTAAAGAAGGCTGCTGAACAACTAACCT---------------------------------------------------------------------------------------------------------------------------------------------------------------------------------------------------------------------------------------------------------------------------------------GATGAAGTTTCAGCTCAGCTTCAGCTGTATAAATAATTTCGAGAAAAACAAAATTCTAGGATCTCCATTTCAAATCAAAAGTTGCTGAATCTTGTTTTGAATTGGGCCTGAATTCAGCGTCCGAGGCACGTTCTCATCACTGAGCCGATTGCAGCTACGCGATGTCGCGCACGAATTAAAGCAATCGAGGGCGCGTGTGCGTTGTGTCAACGCTTGCGCGCGCTTCGCCAAAT-AAAAAAAAAGAACTAAACGGAAATTTCTTTAGACGCATGTCTTTAGACGCACCGTTGGAAAGGCCTACTTTCGTTGCTTTCTTCAAAATGACAGCATGCCAGAAAGAAAAATCTCTTATCTTTCAACGAAGTGTGTTTTTTAACAATAGGCCTAATTACGTCAGTTACTTTTTGGATAGTTGCAAATAATGATGGACTGTAGCTTTAAGAAATTTGTGGAACTCAAGTTGTGCTTGGCAAGTTTTACTTTAGAATTCGAAAATGCATCGACACCAAAAAAA-GTGTAATTTTGGTCCTATAGTTGAAGTGTCAAACTAGAACAAATTTAATGATAAATTAAATAAATTTAAATAATTTATTCCTACCTTGCTTTGACAGTGATGAAGGAGCTGCGTGACCTTTTGGAATCTACGAAGATTGACCTTCCTGTTGACATCAATGATCCATACGACCTAGGTCTTCTTCTCAGACATTTACGTCACCATTCAAATCTTCTTGCTCGTATTGGAGACCCCGATGTCAAAAAGGAAGTCCTCAGCGCCATGAATGAAAAC

>SS86-B

GCACGACGTGCCGCAGAGGAAAAACCTCAAAAGGGAAAGAAGGGACGAAAAGGAAAGAAACGACGAAAGGGAAAGAAGGGACGAAAAGGAAAGAAGGGACGAAAAGGAAAGAAACGACGAAAGGGAAAGAAAAGACGAAAGGGAAAGAAAGGACGAAAGGGAAAGAAAGGACGAAAGGGAAAGAAAGGACGAAAGGGAAAGAAAGGACGAAAGGGAAAGAAAGGACGAAAGGGAAAGAAAGGACGAAAGGGAAAGAAAGGACGAAAGGGAAAGAAAGGAAAACCAATCCAACCTGAAGAAACACCAGCCATTCCGACGGAGATAAAAGCTGCAGAAATAGAAAAGGAACCAAAAACAGAAGTGGTTGTGGAACCAGTTATTCCGGAAGCAGATATTGCGGAGACCGAAATACAACAAATTAAAGCAGAAGTAGAACCAGTTGAAACAAAGCCAGAAATAGTTATGGAACCAGATGTGGAACCAGTTATTCCAGAAACGGAACTTACAGAGACCGGAAAAGAAGCGAAAGTAGAACAAGTTAAACCAGTGGAAGGGAAGCTAAACTTA------------------------------------------------------------------------------------------------------------------------------------------------------------------------------------------------------------------------------------------------------------------------------------------------------------------------------------------------------------------------------------------------------------------------------------------------------------------------------GGAAAAGGAAAGGGCAAAGGAAAGAAGGTTAGAAAGGGAAAGAAAGGACGAAAGGGAAAGAAAGGACGAAAGGGAAAGAAAGGACGGAAAGGAAAGAAGGGGCGAAAAGGAAAGAAAAGAAGAAAAGGAAAAAAAGGACGAAAGGGAAAGAAAGGACGAAAGGGAAAGAAAGGACGAAAGGGCAAGAAAGGACGAAAGGGAAAGAAAGGACGAAAGGGAAAGAAAGGACGAAAGGGAAAGAAAGGACGAANGGGAAAGAAAGGA------------------CGAAAGGGCAAGAAAGGAAATCCAATCAAAACTGAAGAAACACCAGCCATTCTGACGGAGATAAAAGCTGCAGAATTAGAAAAGGAACCAAAAACAGAAATAGTTATGAAACCAGTTATTCCCGAAACGGAACTAACTGAGACCGGAAAAGAAGCAGAAGTAGAACAAGTTAAACCAGTGGAAAGGAAGCTAAAAATAGGAAAAGGAAAGGGCAAAGGAAAGAAGGTTAGAAAGGGAAAGAAAGGACGAAAGGGAAAGAAAGGACGAAAGGGAAAGAAAGGACGGAAAGGAAAGAAGGGGCGAAAAGGAAAGAAAAGAAGAAAAGGAAAGAAAAGACGAAAAGGAAAGAAACGACGAAAGGGAAAGAAAGGACGAAAGGGAAAAAAAGGACGAAAGGGAAAGAAAGGACGAAAGGGAAAAAAAGGACGAAAGGGAAAAAAAGGACGAAAGGGAAAGAAAGGACGAAAGGGAAAGAAAGGCAAACCAATCCAAACTGAAGAAACACCAGCCATTCCGACGGAGATAAAAGCTGCAGAAATAGAAAAGGAACCAAAAACAGAAGTGGTTGTGGAACCACTTATTCAGGAAAAAGATGTTGCGGAGACCGAAATACAACCCATCGAAGCAGACGTAGAACCAGTTGAACCAAAGACAGAAATAGTTATGGAACCAGTTATTCCGGAAGCAGATATTGCGGAGACCGAAATACAACCCATCAAAGCAGAAGAAGAACCAGTT------------------------------------------------------------------------------------------------------------GAACCAAAGCCAGAAATAGTTATGGAACCAGATGAGGAACCAGTTATTCCGGAAGCAGATATTGCGGAGACCGAAATACAACCCATCAAAGCAGAAGAAGAACCAGTTGAACCAAAGCCAGAAATAGTTATGGAACCAGATGAGGAACCAGTTATTCCGGAAGCAGATATTGCGGAGACCGAAATACAACCCATCAAAGCAGAAGAAGAACCAGTTGAACCAAAGCCAGAAATAGTTATGGAACCAGATGAGGAACCAGTTATTCCGGAAGCAGATATTGCGGAGACCGAAATACAACCCATCAGAGCAGAAGAAGAACCAGTTGAACCAATGCCAGAAATAGTTATGGAACCAGATGAGGAACCAGTCATTCCAGAAACAGAACTAACAGAGACAGACAAACAACCAATCGAAGCAGAAGTAGAACCAGTTGAACCAAAAACAGAAGTGGTTGTGGAACCAGTTATTCCGGAAGCAGATATTGCGGAGACCGAAATACAACCCATCGAAGCAGAAGTAGAACCAGTTGAACCAAAGCCAGAAATAGTTATGGAACCAGATGTGGAACCAGTAATTCCAGAAACAGATCTTACAGAGACCGAAAAACAACCAATCGAAGCAGAAGTAGAACCAGTTGAACCGAAGACAGAGATAGTCGAACCAGAAACAGAGCCAGTCGAAGAAGCCGAGGAAGAAGCGGTTGAAGCACCCGTTATGGAACCAGTTATACCTGAGATAGAACCAAAGACAGAACCGGAAGTTGAAGCCGAGGAAAAAGAAGTCGAAACAAACGTTGAACCAGAACTACTGGAGACAGGAAAACCGATTGAAATTGGCCAAGCAACTGAAATCGAGGAGCCACCAGAAAAACAACCGGTGGAATCGGAGTCACAACCAGCGGAAGTTGAGATGGAACAAATTGAAACAAAGGAAGAACAAACTGAACCAAAGGAAGAACTAGCAGGAATTGATGAGAAATGTAAGTTTTAGTAACAATTATTTATTTTATGATTCCTTTTGAGCTGTTTCGGTATGCTTCGATGCAAGTCAAATTTGTGGATGTTTTAAATCCCTCATTCAATTATGTTTGCAATGTAGTCATGAAGTTTGTTCGTTTGTTCAACACAGATATGGGACATATACGCATGAGCAGACCTTTTTTGTGCACAGATCTTCGTTCAGGTTAATCGTGCATGGCAACAAAAATTGGTTCATTCCGTTTTGTGTCGTGCAGTCTTATTTTGCTTGAATTAAGCATTGCTGTAAAGTTCAAGTTAAAGGTACAGTCCATCATTTGTAATTTGTGCATTTTGTTCGTTTGAAGAAACAAACATGCTCAAAATCTAAAGAAGGCTGCTGAACAACTAACCT---------------------------------------------------------------------------------------------------------------------------------------------------------------------------------------------------------------------------------------------------------------------------------------GATGAAGTTTCAGCTCAGCTTCAGCTGTATAAATAATTTCGAGAAAAACAAAATTCTAGGATCTCCATTTCAAATCAAAAGTTGCTGAATCTTGTTTTGAATTGGGCCTGAATTCAGCGTCCGAGGCACGTTCTCATCACTGAGCCGATTGCAGCTACGCGATGTCGCGCACGAATTAAAGCAATCGAGGGCGCGTGTGCGTTGTGTCAACGCTTGCGCGCGCTTCGCCAAATAAAAAAAAAAGAACTAAACGGAAATTTCTTTAGACGCATGTCTTTAGACGCACCGTTGGAAAGGCCTACTTTCGTTGCTTTCTTCAAAATGACAGCATGCCAGAAAGAAAAATCTCTTATCTTTCAACGAAGTGTGTTTTTTAACAATAGGCCTAATTACGTCAGTTACTTTTTGGATAGTTGCAAATAATGATGGACTGTAGCTTTAAGAAATTTGTGGAACTCAAGTTGTGCTTGGCAAGTTTTACTTTAGAATTCGAAAATGCATCGACACCAAAAAAAAGTGTAATTTTGGTCCTATAGTTGAAGTGTCAAACTAGAACAAATTTAATGATAAATTAAATAAATTTAAATAATTTATTCCTACCTTGCTTTGACAGTGATGAAGGAGCTGCGTGACCTTTTGGAATCTACGAAGATTGACCTTCCTGTTGACATCAATGATCCATACGACCTAGGTCTTCTTCTCAGACATTTACGTCACCATTCAAATCTTCTTGCTCGTATTGGAGACCCCGATGTCAAAAAGGAAGTCCTCAGCGCCATGAATGAAAAC

>SS12-A

GCACGACGTGCCGCAGAGGAAAAACCTCAAAAGGGAAAGAAGGGACGAAAAGGAAAGAAACGACGAAAGGGAAAGAAGGGACGAAAAGGAAAGAAGGGACGAAAAGGAAAGAAACGACGAAAGGGAAAGAAAAGACGAAAGGGAAAGAAAGGACGAAAGGGAAAGAAAGGACGAAAGGGAAAGAAAGGACGAAAGGGAAAGAAAGGACGAAAGGGAAAGAAAGGACGAAAGGGAAAGAAAGGACGAAAGGGAAAGAAAGGACGAAAGGGAAAGAAAGGAAAACCAATCCAACCTGAAGAAACACCAGCCATTCCGACGGAGATAAAAGCTGCAGAAATAGAAAAGGAACCAAAAACAGAAGTGGTTGTGGAACCAGTTATTCCGGAAGCAGATATTGCGGAGACCGAAATACAACAAATTAAAGCAGAAGTAGAACCAGTTGAAACAAAGCCAGAAATAGTTATGGAACCAGATGTGGAACCAGTTATTCCAGAAACGGAACTTACAGAGACCGGAAAAGAAGCGAAAGTAGAACAAGTTAAACCAGTGGAAGGGAAGCTAAACTTA------------------------------------------------------------------------------------------------------------------------------------------------------------------------------------------------------------------------------------------------------------------------------------------------------------------------------------------------------------------------------------------------------------------------------------------------------------------------------GGAAAAGGAAAGGGCAAAGGAAAGAAGGTTAGAAAGGGAAAGAAAGGACGAAAGGGAAAGAAAGGACGAAAGGGAAAGAAAGGACGGAAAGGAAAGAAGGGGCGAAAAGGAAAGAAAAGAAGAAAAGGAAAAAAAGGACGAAAGGGAAAGAAAGGACGAAAGGGAAAGAAAGGACGAAAGGGCAAGAAAGGACGAAAGGGAAAGAAAGGACGAAAGGGAAAGAAAGGACGAAAGGGAAAGAAAGGACGAAGGGGAAAGAAAGGA------------------CGAAAGGGCAAGAAAGGAAATCCAATCAAAACTGAAGAAACACCAGCCATTCTGACGGAGATAAAAGCTGCAGAATTAGAAAAGGAACCAAAAACAGAAATAGTTATGAAACCNGTTATTCCCGAAACGGAACTAACAGAGACCGGAAAAGAAGCAGAAGTAGAACAAGTTAAACCAGTGGAAAGGAAGCTAAAAATAGGAAAAGGAAAGGGCAAAGGAAAGAAGGTTAGAAAGGGAAAGAAAGGACGAAAGGGAAAGAAAGGACGAAAGGGAAAGAAAGGACGGAAAGGAAAGAAGGGGCGAAAAGGAAAGAAAAGAAGAAAAGGAAAGAAAAGACGAAAAGGAAAGAAACGACGAAAGGGAAAGAAAGGACGAAAGGGAAAAAAAGGACGAAAGGGAAAGAAAGGACGAAAGGGAAAAAAAGGACGAAAGGGAAAAAAAGGACGAAAGGGAAAGAAAGGACGAAAGGGAAAGAAAGGCAAACCAATCCAAACTGAAGAAACACCAGCCATTCCGACGGAGATAAAAGCTGCAGAAATAGAAAAGGAACCAAAAACAGAAGTGGTTGTGGAACCACTTATTCAGGAAAAAGATGTTGCGGAGACCGAAATACAACCCATCGAAGCAGAAGTAGAACCAGTTGAACCAAAGACAGAAATAGTTATGGAACCAGTTATTCCGGAAGCAGATATTGCGGAGACCGAAATACAACCCATCAAAGCAGAAGAAGAACCAGTT------------------------------------------------------------------------------------------------------------GAACCAAAGCCAGAAATAGTTATGGAACCAGATGAGGAACCAGTTATTCCGGAAGCAGATATTGCGGAGACCGAAATACAACCCATCAAAGCAGAAGAAGAACCAGTTGAACCAAAGCCAGAAATAGTTATGGAACCAGATGAGGAACCAGTTATTCCGGAAGCAGATATTGCGGAGACCGAAATACAACCCATCAAAGCAGAAGAAGAACCAGTTGAACCAAAGCCAGAAATAGTTATGGAACCAGATGAGGAACCAGTTATTCCGGAAGCAGATATTGCGGAGACCGAAATACAACCCATCAAAGCAGAAGAAGAACCAGTTGAACCAAAGCCAGAAATAGTTATGGAACCAGATGAGGAACCAGTTATTCCAGAAACAGAACTAACAGAGACAGACAAACAACCAATCGAAGCAGAAGTAGAACCAGTTGAACCAAAAACAGAAGTGGTTGTGGAACCAGTTATTCCGGAAGCAGATATTGCGGAGACCGAAATACAACCCATCGAAGCAGAAGTAGAACCAGTTGAACCAAAGCCAGAAATAGTTATGGAACCAGATGTGGAACCAGTAATTCCAGAAACAGATCTTACAGAGACCGAAAAACAACCAATCGAAGCAGAAGTAGAACCAGTTGAACCGAAGACAGAGATAGTCGAACCAGAAACAGAGCCAGTCGAAGAAGCCGAGGAAGAAGCGGTTGAAGCACCCGTTATGGAACCAGTTATACCTGAGATAGAACCAAAGACAGAACCGGAAGTTGAAGCCGAGGAAAAAGAAGTCGAAACAAACGTTGAACCAGAACTACTGGAGACAGGAAAACCGATTGAAATTGGCCAAGCAACTGAAATCGAGGAGCCACCAGAAAAACAACCGGTGGAATCGGAGTCACAACCAGCGGAAGTTGAGATGGAACAAATTGAAACAAAGGAAGAACAAACTGAACCAAAGGAAGAACTAGCAGGAATTGATGAGAAATGTAAGTTTTAGTAACAATTATTTATTTTATGATTCCTTTTGAGCTGTTTCGGTATGCTTCGATGCAAGTCAAATTTGTGGATGTTTTAAATCCATCATTCAATTATGTTTGCAATGTAGTCATGAAGTTTGTTCGTTTGTTCAACACAGATATGGGACATATACGCATGAGCAGACCTTTTTTGTGCACAGATCTTCGTTCAGGTTAATCGTGCATGGCAACAAAAATTGGTTCATTCTGTTTTATGTCGTGCAGTCTTATTTTGCTTGAATTAAGCATTGCTGTAAAGTTCAAGTTAAAGGTACAGTCCATCATTTGTAATTTGTGCATTTTGTTCGTTTGAAGAAACAAACATGCTCAAAATCTAAAGAAGGCTGCTGAACAACTAACCT---------------------------------------------------------------------------------------------------------------------------------------------------------------------------------------------------------------------------------------------------------------------------------------GATGAAGTTTCAGCTCAGCTTCAGCTGTATAAATTATTTCGAGAAAAACAAAATTCTAGGATCTCCATTTCAAATCAAAAGTTGCTGAATCTTGTTTTGAATTGGGCCTGAATTCAGCGTCCGAGGCACGTTCTCATCACTGAGCCGATTGCAGCTACGCGATGTCGCGCACGAATTAAAGCAATCGAGGGCGCGTGTGCGTTGTGTCAACGCTTGCGCGCGCTTCGCCAAATAAAAAAAAAAGAACTAAACGGAAATTTCTTTAGACGCATGTCTTTAGACGCACCGTTGGAAAGGCCTACTTTCGTTGCTTTCTTCAAAATGACAGCATGCCAGAAAGAAAAATCTCTTATCTTTCAACGAAGTATGTTTTTTAACAATAGGCCTAATTACGTCAGTTACTTTTTGGATAGTTGCAAATAATGATGGACTGTAGCTTTAAGAAATTTGTGGAACTCAAGTTGTGCTTGGCAAGTTTTACTTTAGAATTCGAAAATGCATCGACACCAAAAAAAAGTGTAATTTTGGTCCTATAGTTGAAGTGTCAAACTAGAACAAATTTAATGATAAATTAAATAAATTTAAATAATTTATTCCTACCTTGCTTTGACAGTGATGAAGGAGCTGCGTGACCTTTTGGAATCTACGAAGATTGACCTTCCTGTTGACATCAATGATCCATACGACCTAGGTCTTCTTCTCAGACATTTACGTCACCATTCAAATCTTCTTGCTCGTATTGGAGACCCCGATGTCAAAAAGGAAGTCCTCAGCGCCATGAATGAAAAC

>SS74-A

GCACGACGTGCCGCAGAGGAAAAACCTCAAAAGGGAAAGAAGGGACGAAAAGGAAAGAAACGACGAAAGGGAAAGAAGGGACGAAAAGGAAAGAAGGGACGAAAAGGAAAGAAACGACGAAAGGGAAAGAAAAGACGAAAGGGAAAGAAAGGACGAAAGGGAAAGAAAGGACGAAAGGGAAAGAAAGGACGAAAGGGAAAGAAAGGACGAAAGGGAAAGAAACGACGAAAGGGAAAGAAAGGACGAAAGGGAAAGAAAGGACGAAAGGGAAAGAAAGGAAAACCAATCCAACCTGAAGAAACACCAGCCATTCCGACGGAGATAAAAGCTGCAGAAATAGAAAAGGAACCAAAAACAGAAGTGGTTGTGGAACCAGTTATTCCGGAAGCAGATATTGCGGAGACCGAAATACAACAAATTAAAGCAGAAGTAGAACCAGTTGAAACAAAGCCAGAAATAGTTATGGAACCAGATGTGGAACCAGTTATTCCAGAAACGGAACTTACAGAGACCGGAAAAGAAGCGAAAGTAGAACAAGTTAAACCAGTGGAAGGGAAGCTAAACTTA------------------------------------------------------------------------------------------------------------------------------------------------------------------------------------------------------------------------------------------------------------------------------------------------------------------------------------------------------------------------------------------------------------------------------------------------------------------------------GGAAAAGGAAAGGGCAAAGGAAAGAAGGTTAGAAAGGGAAAGAAAGGACGAAAGGGAAAGAAAGGACGAAAGGGAAAGAAAGGACGGAAAGGAAAGAAGGGGCGAAAAGGAAAGAAAAGAAGAAAAGGAAAAAAAGGACGAAAGGGAAAGAAAGGACGAAAGGGAAAGAAAGGACGAAAGGGCAAGAAAGGACGAAAGGGAAAGAAAGGACGAAAGGGAAAGAAAGGACGAAAGGGAAAGAAAGGACGAAGGGGAAAGAAAGGA------------------CGAAAGGGCAAGAAAGGAAATCCAATCAAAACTGAAGAAACACCAGCCATTCTGACGGAGATAAAAGCTGCAGAATTAGAAAAGGAACCAAAAACAGAAATAGTTATGAAACCAGTTATTCCCGAAACGGAACTAACAGAGACCGGAAAAGAAGCAGAAGTAGAACAAGTTAAACCAGTGGAAAGGAAGCTAAAAATAGGAAAAGGAAAGGGCAAAGGAAAGAAGGTTAGAAAGGGAAAGAAAGGACGAAAGGGAAAGAAAGGACGAAAGGGAAAGAAAGGACGAAAAGGAAAGAAGGGGCGAAAAGGAAAGAAAAGAAGAAAAGGAAAGAAAAGACGAAAAGGAAAGAAACGACGAAAGGGAAAGAAAGGACGAAAGGGAAAAAAAGGACGAAAGGGAAAGAAAGGACGAAAGGGAAAAAAAGGACGAAAGGGAAAAAAAGGACGAAAGGGAAAGAAAGGACGAAAGGGAAAGAAAGGCAAACCAATCCAAACTGAAGAAACACCAGCCATTCCGACGGAGATAAAAGCTGCAGAAATAGAAAAGGAACCAAAAACAGAAGTGGTTGTGGAACCACTTATTCAGGAAAAAGATGTTGCGGAGACCGAAATACAACCCATCGAAGCAGAAGTAGAACCAGTTGAACCAAAGACAGAAATAGTTATGGAACCAGTTATTCCGGAAGCAGATATTGCGGAGACCGAAATACAACCCATCAAAGCAGAAGAAGAACCAGTT------------------------------------------------------------------------------------------------------------GAACCAAAGCCAGAAATAGTTATGGAACCAGATGAGGAACCAGTTATTCCGGAAGCAGATATTGCGGAGACCGAAATACAACCCATCAAAGCAGAAGAAGAACCAGTTGAACCAAAGCCAGAAATAGTTATGGAACCAGATGAGGAACCAGTTATTCCGGAAGCAGATATTGCGGAGACCGAAATACAACCCATCAAAGCAGAAGAAGAACCAGTTGAACCAAAGCCAGAAATAGTTATGGAACCAGATGAGGAACCAGTTATTCCGGAAGCAGATATTGCGGAGACCGAAATACAACCCATCAGAGCAGAAGAAGAACCAGTTGAACCAAAGCCAGAAATAGTTATGGAACCAGATGAGGAACCAGTCATTCCAGAAACAGAACTAACAGAGACAGACAAACAACCAATCGAAGCAGAAGTAGAACCAGTTGAACCAAAAACAGAAGTGGTTGTGGAACCAGTTATTCCGGAAGCAGATATTGCGGAGACCGAAATACAACCCATCGAAGCAGAAGTAGAACCAGTTGAACCAAAGCCAGAAATAGTTATGGAACCAGATGTGGAACCAGTAATTCCAGAAACAGATCTTACAGAGACCGAAAAACAACCAATCGAAGCAGAAGTAGAACCAGTTGAACCGAAGACAGAGATAGTCGAACCAGAAACAGGGCCAGTCGAAGAAGCCGAGGAAGAAGCGGTTGAAGCACCCGTTATGGAACCAGTTATACCTGAGATAGAACCAAAGACAGAACCGGAAGTTGAAGCCGAGGAAAAAGAAGTCGAAACAAACGTTGAACCAGAACTACTGGAGACAGGAAAACCGATTGAAATTGGCCAAGCAACTGAAATCGAGGAGCCACCAGAAAAACAACCGGTGGAATCGGAGTCACAACCAGCGGAAGTTGAGATGGAACAAATTGAAACAAAGGAAGAACAAACTGAACCAAAGGAAGAACTAGCAGGAATTGATGAGAAATGTAAGTTTTAGTAACAATTATTTATTTTATGATTCCTTTTGAGCTGTTTCGGTATACTTCGATGCAAGTCAAATTTGTGGATGTTTTAAATCCATCATTCAATTATGTTTGCAATGTAGTCATGAAGTTTGTTCGTTTGTTCAACACAGATATGGGACATATACGCATGAGCAGACCTTTTTTGTGCACAGATCTTCGTTCAGGTTAATCGTGCATGGCAACAAAAATTGGTTCATTCTGTTTTATGTCGTGCAGTCTTATTTTGCTTGAATTAAGCATTGCTGTAAAGTTCAAGTTAAAGGTACAGTCCATCATTTGTAATTTGTGCATTTTGTTCGTTTGAAGAAACAAACATGCTCAAAATCTAAAGAAGGCTGCTGAACAACTAACCT---------------------------------------------------------------------------------------------------------------------------------------------------------------------------------------------------------------------------------------------------------------------------------------GATGAAGTTTCAGCTCAGCTTCAGCTGTATAAATTATTTCGAGAAAAACAAAATTCTAGGATCTCCATTTCAAATCAAAAGTTGCTGAATCTTGTTTTGAATTGGGCCTGAATTCAGCGTCCGAGGCACGTTCTCATCACTGAACCGATTGCAGCTACGCGATGTCGCGCACGAATTAAAGCAATCGAGGGCGCGTGTGCGTTGTGTCAACGCTTGCGCGCGCTTCGCCAAATAAAAAAAAAAGAACTAAACGGAAATTTCTTTAGACGCATGTCTTTAGACGCACCGTTGGAAAGGCCTACTTTCGTTGCTTTCTTCAAAATGACAGCATGCCAGAAAGAAAAATCTCTTATCTTTCAACGAAGTATGTTTTTTAACAATAGGCCTAATTACGTCAGTTACTTTTTGGATAGTTGCAAATAATGATGGACTGTAGCTTTAAGAAATTTGTGGAACTCAAGTTGTGCTTGGCAAGTTTTACTTTAGAATTCGAAAATGCATCGACACCAAAAAAAAGTGTAATTTTGGTCCTATAGTTGAAGTGTCAAACTAGAACAAATTTAATGATAAATTAAATAAATTTAAATAATTTATTCCTACCTTGCTTTGACAGTGATGAAGGAGCTGCGTGACCTTTTGGAATCTACGAAGATTGACCTTCCTGTTGACATCAATGATCCATACGACCTAGGTCTTCTTCTCAGACATTTACGTCACCATTCAAATCTTCTTGCTCGTATTGGAGACCCCGATGTCAAAAAGGAAGTCCTCAGCGCCATGAATGAAAAC

>SS76-A

GCACGACGTGCCGCAGAGGAAAAACCTCAAAAGGGAAAGAAGGGACGAAAAGGAAAGAAACGACGAAAGGGAAAGAAGGGACGAAAAGGAAAGAAGGGACGAAAAGGAAAGAAACGACGAAAGGGAAAGAAAAGACGAAAGGGAAAGAAAGGACGAAAGGGAAAGAAAGGACGAAAGGGAAAGAAAGGACGAAAGGGAAAGAAAGGACGAAAGGGAAAGAAAGGACGAAAGGGAAAGAAAGGACGAAAGGGAAAGAAAGGACGAAAGGGAAAGAAAGGAAAACCAATCCAACCTGAAGAAACACCAGCCATTCCGACGGAGATAAAAGCTGCAGAAATAGAAAAGGAACCAAAAACAGAAGTGGTTGTGGAACCAGTTATTCCGGAAGCAGATATTGCGGAGACCGAAATACAACAAATTAAAGCAGAAGTAGAACCAGTTGAAACAAAGCCAGAAATAGTTATGGAACCAGATGTGGAACCAGTTATTCCAGAAACGGAACTTACAGAGACCGGAAAAGAAGCGAAAGTAGAACAAGTTAAACCAGTGGAAGGGAAGCTAAACTTA------------------------------------------------------------------------------------------------------------------------------------------------------------------------------------------------------------------------------------------------------------------------------------------------------------------------------------------------------------------------------------------------------------------------------------------------------------------------------GGAAAAGGAAAGGGCAAAGGAAAGAAGGTTAGAAAGGGAAAGAAAGGACGAAAGGGAAAGAAAGGACGAAAGGGAAAGAAAGGACGGAAAGGAAAGAAGGGGCGAAAAGGAAAGAAAAGAAGAAAAGGAAAAAAAGGACGAAAGGGAAAGAAAGGACGAAAGGGAAAGAAAGGACGAAAGGGCAAGAAAGGACGAAAGGGAAAGAAAGGACGAAAGGGAAAGAAAGGACGAAAGGGAAAGAAAGGACGAAGGGGAAAGAAAGGA------------------CGAAAGGGCAAGAAAGGAAATCCAATCAAAACTGAAGAAACACCAGCCATTCTGACGGAGATAAAAGCTGCAGAATTAGAAAAGGAACCAAAAACAGAAATAGTTATGAAACCAGTTATTCCCGAAACGGAACTAACAGAGACCGGAAAAGAAGCAGAAGTAGAACAAGTTAAACCAGTGGAAAGGAAGCTAAAAATAGGAAAAGGAAAGGGCAAAGGAAAGAAGGTTAGAAAGGGAAAGAAAGGACGAAAGGGAAAGAAAGGACGAAAGGGAAAGAAAGGACGGAAAGGAAAGAAGGGGCGAAAAGGAAAGAAAAGAAGAAAAGGAAAGAAAAGACGAAAAGGAAAGAAACGACGAAAGGGAAAGAAAGGACGAAAGGGAAAAAAAGGACGAAAGGGAAAGAAAGGACGAAAGGGAAAAAAAGGACGAAAGGGAAAAAAAGGACGAAAGGGAAAGAAAGGACGAAAGGGAAAGAAAGGCAAACCAATCCAAACTGAAGAAACACCAGCCATTCCGACGGAGATAAAAGCTGCAGAAATAGAAAAGGAACCAAAAACAGAAGTGGTTGTGGAACCACTTATTCAGGAAAAAGATGTTGCGGAGACCGAAATACAACCCATCGAAGCAGAAGTAGAACCAGTTGAACCAAAGACAGAAATAGTTATGGAACCAGTTATTCCGGAAGCAGATATTGCGGAGACCGAAATACAACCCATCAAAGCAGAAGAAGAACCAGTT------------------------------------------------------------------------------------------------------------GAACCAAAGCCAGAAATAGTTATGGAACCAGATGAGGAACCAGTTATTCCGGAAGCAGATATTGCGGAGACCGAAATACAACCCATCAAAGCAGAAGAAGAACCAGTTGAACCAAAGCCAGAAATAGTTATGGAACCAGATGAGGAACCAGTTATTCCGGAAGCAGATATTGCGGAGACCGAAATACAACCCATCAAAGCAGAAGAAGAACCAGTTGAACCAAAGCCAGAAATAGTTATGGAACCAGATGAGGAACCAGTTATTCCGGAAGCAGATATTGCGGAGACCGAAATACAACCCATCAGAGCAGAAGAAGAACCAGTTGAACCAAAGCCAGAAATAGTTATGGAACCAGATGAGGAACCAGTCATTCCAGAAACAGAACTAACAGAGACAGACAAACAACCAATCGAAGCAGAAGTAGAACCAGTTGAACCAAAAACAGAAGTGGTTGTGGAACCAGTTATTCCGGAAGCAGATATTGCGGAGACCGAAATACAACCCATCGAAGCAGAAGTAGAACCAGTTGAACCAAAGCCAGAAATAGTTATGGAACCAGATGTGGAACCAGTAATTCCAGAAACAGATCTTACAGAGACCGAAAAACAACCAATCGAAGCAGAAGTAGAACCAGTTGAACCGAAGACAGAGATAGTCGAACCAGAAACAGAGCCAGTCGAAGAAGCCGAGGAAGAAGCGGTTGAAGCACCCGTTATGGAACCAGTTATACCTGAGATAGAACCAAAGACAGAACCGGAAGTTGAAGCCGAGGAAAAAGAAGTCGAAACAAACGTTGAACCAGAACTACTGGAGACAGGAAAACCGATTGAAATTGGCCAAGCAACTGAAATCGAGGAGCCACCAGAAAAACAACCGGTGGAATCGGAGTCACAACCAGCGGAAGTTGAGATGGAACAAATTGAAACAAAGGAAGAACAAACTGAACCAAAGGAAGAACTAGCAGGAATTGATGAGAAATGTAAGTTTTAGTAACAATTATTTATTTTATGATTCCTTTTGAGCTGTTTCGGTATGCTTCGATGCAAGTCAAATTTGTGGATGTTTTAAATCCATCATTCAATTATGTTTGCAATGTAGTCATGAAGTTTGTTCGTTTGTTCAACACAGATATGGGACATATACGCATGAGCAGACCTTTTTTGTGCACAGATCTTCGTTCAGGTTAATCGTGCATGGCAACAAAAATTGGTTCATTCTGTTTTATGTCGTGCAGTCTTATTTTGCTTGAATTAAGCATTGCTGTAAAGTTCAAGTTAAAGGTACAGTCCATCATTTGTAATTTGTGCATTTTGTTCGTTTGAAGAAACAAACATGCTCAAAATCTAAAGAAGGCTGCTGAACAACTAACCT---------------------------------------------------------------------------------------------------------------------------------------------------------------------------------------------------------------------------------------------------------------------------------------GATGAAGTTTCAGCTCAGCTTCAGCTGTATAAATTATTTCGAGAAAAACAAAATTCTAGGATCTCCATTTCAAATCAAAAGTTGCTGAATCTTGTTTTGAATTGGGCCTGAATTCAGCGTCCGAGGCACGTTCTCATCACTGAGCCGATTGCANCTACGCGATGTCGCGCACGAATTAAAGCAATCGAGGGCGCGTGTGCGTTGTGTCAACGCTTGCGCGCGCTTCGCCAAAT-AAAAAAAAAGAACTAAACGGAAATTTCTTTAGACGCATGTCTTTAGACGCACCGTTGGAAAGGCCTACTTTCGTTGCTTTCTTCAAAATGACAGCATGCCAGAAAGAAAAATCTCTTATCTTTCAACGAAGTATGTTTTTTAACAATAGGCCTAATTACGTCAGTTACTTTTTGGATAGTTGCAAATAATGATGGACTGTAGCTTTAAGAAATTTGTGGAACTCAAGTTGTGCTTGGCAAGTTTTACTTTAGAATTCGAAAATGCATCGACACCAAAAAAAAGTGTAATTTTGGTCCTATACTTGAAGTGTCAAACTAGAACAAATTTAATGATAAATTAAATAAATTTAAATAATTTATTCCTACCTTGCTTTGACAGTGATGAAGGAGCTGCGTGACCTTTTGGAATCTACGAAGATTGACCTTCCTGTTGACATCAATGATCCATACGACCTAGGTCTTCTTCTCAGACATTTACGTCACCATTCAAATCTTCTTGCTCGTATTGGAGACCCCGATGTCAAAAAGGAAGTCCTCAGCGCCATGAATGAAAAC

>SS80-B

GCACGACGTGCCGCAGAGGAAAAACCTCAAAAGGGAAAGAAGGGACGAAAAGGAAAGAAACGACGAAAGGGAAAGAAGGGACGAAAAGGAAAGAAGGGACGAAAAGGAAAGAAACGACGAAAGGGAAAGAAAAGACGAAAGGGAAAGAAAGGACGAAAGGGAAAGAAAGGACGAAAGGGAAAGAAAGGACGAAAGGGAAAGAAAGGACGAAAGGGAAAGAAAGGACGAAAGGGAAAGAAAGGACGAAAGGGAAAGAAAGGACGAAAGGGAAAGAAAGGAAAACCAATCCAACCTGAAGAAACACCAGCCATTCCGACGGAGATAAAAGCTGCAGAAATAGAAAAGGAACCAAAAACAGAAGTGGTTGTGGAACCAGTTATTCCGGAAGCAGATATTGCGGAGACCGAAATACAACAAATTAAAGCAGAAGTAGAACCAGTTGAAACAAAGCCAGAAATAGTTATGGAACCAGATGTGGAACCAGTTATTCCAGAAACGGAACTTACAGAGACCGGAAAAGAAGCGAAAGTAGAACAAGTTAAACCAGTGGAAGGGAAGCTAAACTTA------------------------------------------------------------------------------------------------------------------------------------------------------------------------------------------------------------------------------------------------------------------------------------------------------------------------------------------------------------------------------------------------------------------------------------------------------------------------------GGAAAAGGAAAGGGCAAAGGAAAGAAGGTTAGAAAGGGAAAGAAAGGACGAAAGGGAAAGAAAGGACGAAAGGGAAAGAAAGGACGGAAAGGAAAGAAGGGGCGAAAAGGAAAGAAAAGAAGAAAAGGAAAAAAAGGACGAAAGGGAAAGAAAGGACGAAAGGGAAAGAAAGGACGAAAGGGCAAGAAAGGACGAAAGGGAAAGAAAGGACGAAAGGGAAAGAAAGGACGAAAGGGAAAGAAAGGACGAAGGGGAAAGAAAGGA------------------CGAAAGGGCAAGAAAGGAAATCCAATCAAAACTGAAGAAACACCAGCCATTCTGACGGAGATAAAAGCTGCAGAATTAGAAAAGGAACCAAAAACAGAAATAGTTATGAAACCAGTTATTCCCGAAACGGAACTAACAGAGACCGGAAAAGAAGCAGAAGTAGAACAAGTTAAACCAGTGGAAAGGAAGCTAAAAATAGGAAAAGGAAAGGGCAAAGGAAAGAAGGTTAGAAAGGGAAAGAAAGGACGAAAGGGAAAGAAAGGACGAAAGGGAAAGAAAGGACGGAAAGGAAAGAAGGGGCGAAAAGGAAAGAAAAGAAGAAAAGGAAAGAAAAGACGAAAAGGAAAGAAACGACGAAAGGGAAAGAAAGGACGAAAGGGAAAAAAAGGACGAAAGGGAAAGAAAGGACGAAAGGGAAAAAAAGGACGAAAGGGAAAAAAAGGACGAAAGGGAAAGAAAGGACGAAAGGGAAAGAAAGGAAAACCAATCCAAACTGAAGAAACACCAGCCATTCCGACGGATATAAAAGCTGCAGAAATAGAAAAGGAACCAAAAACAGAAGTGGTTGTGGAACCACTTATTCAGGAAAAAGATGTTGCGGAGACCGAAATACAACCCATCGAAGCAGAAGTAGAACCAGTTGAACCAAAGACAGAAATAGTTATGGAACCAGTTATTCCGGAAGCAGATATTGCGGAGACCGAAATACAACCCATCAGAGCAGAAGAAGAACCAGTT------------------------------------------------------------------------------------------------------------------------------------------------------------------------------------------------------------------------------------------------------------------------------------------------------------------------------------------------------------------------------------------------------------------------------------------------GAACCAAAGCCAGAAATAGTTATGGAACCAGATGAGGAACCAGTCATTCCAGAAACAGAACTAACAGAGACAGACAAACAACCAATCGAAGCAGAAGTAGAACCAGTTGAACCAAAAACAGAAGTGGTTGTGGAACCAGTTATTCCGGAAGCAGATATTGCGGAGACCGAAATACAACCCATCGAAGCAGAAGTAGAACCAGTTGAACCAAAGCCAGAAATAGTTATGGAACCAGATGTGGAACCAGTAATTCCAGAAACAGATCTTACAGAGACCGAAAAACAACCAATCGAAGCAGAAGTAGAACCAGTTGAACCGAAGACAGAGATAGTCGAACCAGAAACAGAGCCAGTCGAAGAAGCCGAGGAAGAAGCGGTTGAAGCACCCGTTACGGAACCAGTTATACCTGAGATAGAACCAAAGACAGAACCGGAAGTTGAAGCCGAGGAAAAAGAAGTCGAAACAAACGTTGAACCAGAACTACTGGAGACAGGAAAACCGATTGAAATTGGCCAAGCAACTGAAATCGAGGAGCCACCAGAAAAACAACCGGTGGAATCGGAGTCACAACCAGCGGAAGTTGAGATGGAACAAATTGAAACAAAGGAAGAACCAACTGAACCAAAGGAAGAACTAGCAGGAATTGATGAGAAATGTAAGTTTTAGTAACAATTATTTATTTTATGATTCCTTTTGAGCTGTTTCGGTATGCTTCGATGCAAGTCAAATTTGTAGATGTTTTAAATCCATCATTCAATT--GTTTGCAATGTAGTCATGAAGTTTGTTCGTTTGTTCAACACAGATATGGGACATATACGCATGAGCAGACCTTTTTTGTGCACAGATCTTCGTTCAGGTTAATCGTGCATGGCAACAAAAATTGGTTCATTCCGTTTTATGTCGTGCAGTCTTATTTTGCTTGAATTAAGCATTGCTGTAAAGTTCAAGTTAAAGGTACAGTCCATCATTTGTAATTTGTGCATTTTGTTCGTTTGAAGAAACAAACATGCTCAAAATCTAAAGAAGGCTGCTGAACAACTAACCT---------------------------------------------------------------------------------------------------------------------------------------------------------------------------------------------------------------------------------------------------------------------------------------GATGAAGTTTCAGCTCAGCTTCAGCTGTATAAATTATTTCGAGAAAAACAAAATTCTAGGATCTCCATTTCAAATCAAAAGTTGCTGAATCTTGTTTTGAATTGGGCCTGAATTCAGCGTCCGAGGCACGTTCTCATCACTGAGCCGATTGCAGCTACGCGATGTCGCGCACGAATTAAAGCAATCGAGGGCGCGTGTGCGTTGTGTCAACGCTTGCGCGCGCTTCGCCAAATAAAAAAAAAAGAACTAAACGGAAATTTCTTTAGACGCATGTCTTTAGACGCACCGTTGGAAAGGCCTACTTTCGTTGCTTTCTTCAAAATGACAGCATGCCAGAAAGAAAAATCTCTTATCTTTCAACGAAGTATGTTTTTTAACAATAGGCCTAATTACGTCAGTTACTTTTTGGATAGTTGCAAATAATGATGGACTGTAGCTTTAAGAAATTTGTGGAACTCAAGTTGTGCTTGGCAAGTTTTACTTTAGAATTCGAAAATGCATCGACACCAAAAAAAAGTGTAATTTTGGTCCTATACTTGAAGTGTCAAACTAGAACAAATTTAATGATAAATTAAATAAATTTAAATAATTTATTCCTACCTTGCTTTGACAGTGATGAAGGAGCTGCGTGACCTTTTGGAATCTACGAAGATTGACCTTCCTGTTGACATCAATGATCCATACGACCTAGGTCTTCTTCTCAGACATTTACGTCACCATTCAAATCTTCTTGCTCGTATTGGAGACCCCGATGTCAAAAAGGAAGTCCTCAGCGCCATGAATGAAAAC

>SS90-A

GCACGACGTGCCGCAGAGGAAAAACCTCAAAAGGGAAAGAAGGGACGAAAAGGAAAGAAACGACGAAAGGGAAAGAAGGGACGAAAAGGAAAGAAGGGACGAAAAGGAAAGAAACGACGAAAGGGAAAGAAAAGACGAAAGGGAAAGAAAGGACGAAAGGGAAAGAAAGGACGAAAGGGAAAGAAAGGACGAAAGGGAAAGAAAGGACGAAAGGGAAAGAAAGGACGAAAGGGAAAGAAAGGACGAAAGGGAAAGAAAGGACGAAAGGGAAAGAAAGGAAAACCAATCCAACCTGAAGAAACACCAGCCATTCCGACGGAGATAAAAGCTGCAGAAATAGAAAAGGAACCAAAAACAGAAGTGGTTGTGGAACCAGTTATTCCGGAAGCAGATATTGCGGAGACCGAAATACAACAAATTAAAGCAGAAGTAGAACCAGTTGAAACAAAGCCAGAAATAGTTATGGAACCAGATGTGGAACCAGTTATTCCAGAAACGGAACTTACAGAGACCGGAAAAGAAGCGAAAGTAGAACAAGTTAAACCAGTGGAAGGGAAGCTAAACTTA------------------------------------------------------------------------------------------------------------------------------------------------------------------------------------------------------------------------------------------------------------------------------------------------------------------------------------------------------------------------------------------------------------------------------------------------------------------------------GGAAAAGGAAAGGGCAAAGGAAAGAAGGTTAGAAAGGGAAAGAAAGGACGAAAGGGAAAGAAAGGACGAAAGGGAAAGAAAGGACGGAAAGGAAAGAAGGGGCGAAAAGGAAAGAAAAGAAGAAAAGGAAAAAAAGGACGAAAGGGAAAGAAAGGACGAAAGGGAAAGAAAGGACGAAAGGGCAAGAAAGGACGAAAGGGAAAGAAAGGACGAAAGGGAAAGAAAGGACGAAAGGGAAAGAAAGGACGAAGGGGAAAGAAAGGA------------------CGAAAGGGCAAGAAAGGAAATCCAATCAAAACTGAAGAAACACCAGCCATTCTGACGGAGATAAAAGCTGCAGAATTAGAAAAGGAACCAAAAACAGAAATAGTTATGAAACCAGTTATTCCCGAAACGGAACTAACTGAGACCGGAAAAGAAGCAGAAGTAGAACAAGTTAAACCAGTGGAAAGGAAGCTAAAAATAGGAAAAGGAAAGGGCAAAGGAAAGAAGGTTAGAAAGGGAAAGAAAGGACGAAAGGGAAAGAAAGGACGAAAGGGAAAGAAAGGACGGAAAGGAAAGAAGGGGCGAAAAGGAAAGAAAAGAAGAAAAGGAAAGAAAAGACGAAAAGGAAAGAAACGACGAAAGGGAAAGAAAGGACGAAAGGGAAAAAAAGGACGAAAGGGAAAGAAAGGACGAAAGGGAAAAAAAGGACGAAAGGGAAAAAAAGGACGAAAGGGAAAGAAAGGACGAAAGGGAAAGAAAGGCAAACCAATCCAAACTGAAGAAACACCAGCCATTCCGACGGANATAAAAGCTGCAGAAATAGAAAAGGAACCAAAAACAGAAGTGGTTGTGGAACCACTTATTCAGGAAAAAGATGTTGCGGAGACCGAAATACAACCCATCGAAGCAGACGTAGAACCAGTTGAACCAAAGACAGAAATAGTTATGGAACCAGTTATTCCGGAAGCAGATATTGCGGAGACCGAAATACAACCCATCAAAGCAGAAGAAGAACCAGTT------------------------------------------------------------------------------------------------------------GAACCAAAGCCAGAAATAGTTATGGAACCAGATGAGGAACCAGTTATTCCGGAAGCAGATATTGCGGAGACCGAAATACAACCCATCAAAGCAGAAGAAGAACCAGTTGAACCAAAGCCAGAAATAGTTATGGAACCAGATGAGGAACCAGTTATTCCGGAAGCAGATATTGCGGAGACCGAAATACAACCCATCAAAGCAGAAGAAGAACCAGTTGAACCAAAGCCAGAAATAGTTATGGAACCAGATGAGGAACCAGTTATTCCGGAAGCAGATATTGCGGAGACCGAAATACAACCCATCAGAGCAGAAGAAGAACCAGTTGAACCAATGCCAGAAATAGTTATGGAACCAGATGAGGAACCAGTCATTCCAGAAACAGAACTAACAGAGACAGACAAACAACCAATCGAAGCAGAAGTAGAACCAGTTGAACCAAAAACAGAAGTGGTTGTGGAACCAGTTATTCCGGAAGCAGATATTGCGGAGACCGAAATACAACCCATCGAAGCAGAAGTAGAACCAGTTGAACCAAAGCCAGAAATAGTTATGGAACCAGATGTGGAACCAGTAATTCCAGAAACAGATCTTACAGAGACCGAAAAACAACCAATCGAAGCAGAAGTAGAACCAGTTGAACCGAAGACAGAGATAGTCGAACCAGAAACAGAGCCAGTCGAAGAAGCCGAGGAAGAAGCGGTTGAAGCACCCGTTATGGAACCAGTTATACCTGAGATAGAACCAAAGACAGAACCGGAAGTTGAAGCCGAGGAAAAAGAAGTCGAAACAAACGTTGAACCAGAACTACTGGAGACAGGAAAACCGATTGAAATTGGCCAAGCAACTGAAATCGAGGAGCCACCAGAAAAACAACCGGTGGAATCGGAGTCACAACCAGCGGAAGTTGAGATGGAACAAATTGAAACAAAGGAAGAACAAACTGAACCAAAGGAAGAACTAGCAGGAATTGATGAGAAATGTAAGTTTTAGTAACAATTATTTATTTTATGATTCCTTTTGAGCTGTTTCGGTATGCTTCGATGCAAGTCAAATTTGTGGATGTTTTAAATCCATCATTCAATTATGTTTGCAATGTAGTCATGAAGTTTGTTCGTTTGTTCAACACAGATATGGGACATATACGCATGAGCAGACCTTTTTTGTGCACAGATCTTCGTTCAGGTTAATCGTGCATGGCAACAAAAATTGGTTCATTCTGTTTTATGTCGTGCAGTCTTATTTTGCTTGAATTAAGCATTGCTGTAAAGTTCAAGTTAAAGGTACAGTCCATCATTTGTAATTTGTGCATTTTGTTCGTTTGAAGAAACAAACATGCTCAAAATCTAAAGAAGGCTGCTGAACAACTAACCT---------------------------------------------------------------------------------------------------------------------------------------------------------------------------------------------------------------------------------------------------------------------------------------GATGAAGTTTCAGCTCAGCTTCAGCTGTATAAATTATTTCGAGAAAAACAAAATTCTAGGATCTCCATTTCAAATCAAAAGTTGCTGAATCTTGTTTTGAATTGGGCCTGAATTCAGCGTCCGAGGCACGTTCTCATCACTGAGCCGATTGCAGCTACGCGATGTCGCGCACGAATTAAAGCAATCGAGGGCGCGTGTGCGTTGTGTCAACGCTTGCGCGCGCTTCGCCAAATAAAAAAAAAAGAACTAAACGGAAATTTCTTTAGACGCATGTCTTTAGACGCACCGTTGGAAAGGCCTACTTTCGTTGCTTTCTTCAAAATGACAGCATGCCAGAAAGAAAAATCTCTTATCTTTCAACGAAGTATGTTTTCTAACAATAGGCCTAATTACGTCAGTTACTTTTTGGATAGTTGCAAATAATGATGGACTGTAGCTTTAAGAAATTTGTGGAACTCAAGTTGTGCTTGGCAAGTTTTACTTTAGAATTCGAAAATGCATCGACACCAAAAAAAAGTGTAATTTTGGTCCTATAGTTGAAGTGTCAAACTAGAACAAATTTAATGATAAATTAAATAAATTTAAATAATTTATTCCTACCTTGCTTTGACAGTGATGAAGGAGCTGCGTGACCTTTTGGAATCTACGAAGATTGACCTTCCTGTTGACATCAATGATCCATACGACCTAGGTCTTCTTCTCAGACATTTACGTCACCATTCAAATCTTCTTGCTCGTATTGGAGACCCCGATGTCAAAAAGGAAGTCCTCAGCGCCATGAATGAAAAC

>SS90-B

GCACGACGTGCCGCAGAGGAAAAACCTCAAAAGGGAAAGAAGGGACGAAAAGGAAAGAAACGACGAAAGGGAAAGAAGGGACGAAAAGGAAAGAAGGGACGAAAAGGAAAGAAACGACGAAAGGGAAAGAAAAGACGAAAGGGAAAGAAAGGACGAAAGGGAAAGAAAGGACGAAAGGGAAAGAAAGGACGAAAGGGAAAGAAAGGACGAAAGGGAAAGAAAGGACGAAAGGGAAAGAAAGGACGAAAGGGAAAGAAAGGACGAAAGGGAAAGAAAGGAAAACCAATCCAACCTGAAGAAACACCAGCCATTCCGACGGAGATAAAAGCTGCAGAAATAGAAAAGGAACCAAAAACAGAAGTGGTTGTGGAACCAGTTATTCCGGAAGCAGATATTGCGGAGACCGAAATACAACAAATTAAAGCAGAAGTAGAACCAGTTGAAACAAAGCCAGAAATAGTTATGGAACCAGATGTGGAACCAGTTATTCCAGAAACGGAACTTACAGAGACCGGAAAAGAAGCGAAAGTAGAACAAGTTAAACCAGTGGAAGGGAAGCTAAACTTA------------------------------------------------------------------------------------------------------------------------------------------------------------------------------------------------------------------------------------------------------------------------------------------------------------------------------------------------------------------------------------------------------------------------------------------------------------------------------GGAAAAGGAAAGGGCAAAGGAAAGAAGGTTAGAAAGGGAAAGAAAGGACGAAAGGGAAAGAAAGGACGAAAGGGAAAGAAAGGACGGAAAGGAAAGAAGGGGCGAAAAGGAAAGAAAAGAAGAAAAGGAAAAAAAGGACGAAAGGGAAAGAAAGGACGAAAGGGAAAGAAAGGACGAAAGGGCAAGAAAGGACGAAAGGGAAAGAAAGGACGAAAGGGAAAGAAAGGACGAAAGGGAAAGAAAGGACGAAGGGGAAAGAAAGGA------------------CGAAAGGGCAAGAAAGGAAATCCAATCAAAACTGAAGAAACACCAGCCATTCTGACGGAGATAAAAGCTGCAGAATTAGAAAAGGAACCAAAAACAGAAATAGTTATGAAACCAGTTATTCCCGAAACGGAACTAACAGAGACCGGAAAAGAAGCAGAAGTAGAACAAGTTAAACCAGTGGAAAGGAAGCTAAAAATAGGAAAAGGAAAGGGCAAAGGAAAGAAGGTTAGAAAGGGAAAGAAAGGACGAAAGGGAAAGAAAGGACGAAAGGGAAAGAAAGGACGGAAAGGAAAGAAGGGGCGAAAAGGAAAGAAAAGAAGAAAAGGAAAGAAAAGACGAAAAGGAAAGAAACGACGAAAGGGAAAGAAAGGACGAAAGGGAAAAAAAGGACGAAAGGGAAAGAAAGGACGAAAGGGAAAAAAAGGACGAAAGGGAAAAAAAGGACGAAAGGGAAAGAAAGGACGAAAGGGAAAGAAAGGCAAACCAATCCAAACTGAAGAAACACCAGCCATTCCGACGGAGATAAAAGCTGCAGAAATAGAAAAGGAACCAAAAACAGAAGTGGTTGTGGAACCACTTATTCAGGAAAAAGATGTTGCGGAGACCGAAATACAACCCATCGAAGCAGAAGTAGAACCAGTTGAACCAAAGACAGAAATAGTTATGGAACCAGTTATTCCGGAAGCAGATATTGCGGAGACCGAAATACAACCCATCAAAGCAGAAGAAGAACCAGTT------------------------------------------------------------------------------------------------------------GAACCAAAGCCAGAAATAGTTATGGAACCAGATGAGGAACCAGTTATTCCGGAAGCAGATATTGCGGAGACCGAAATACAACCCATCAAAGCAGAAGAAGAACCAGTTGAACCAAAGCCAGAAATAGTTATGGAACCAGATGAGGAACCAGTTATTCCGGAAGCAGATATTGCGGAGACCGAAATACAACCCATCAAAGCAGAAGAAGAACCAGTTGAACCAAAGCCAGAAATAGTTATGGAACCAGATGAGGAACCAGTTATTCCGGAAGCAGATATTGCGGAGACCGAAATACAACCCATCAGAGCAGAAGAAGAACCAGTTGAACCAAAGCCAGAAATAGTTATGGAACCAGATGAGGAACCAGTCATTCCAGAAACAGAACTAACAGAGACAGACAAACAACCAATCGAAGCAGAAGTAGAACCAGTTGAACCAAAAACAGAAGTGGTTGTGGAACCAGTTATTCCGGAAGCAGATATTGCGGAGACCGAAATACAACCCATCGAAGCAGAAGTAGAACCAGTTGAACCAAAGCCAGAAATAGTTATGGAACCAGATGTGGAACCAGTAATTCCAGAAACAGATCTTACAGAGACCGAAAAACAACCAATCGAAGCAGAAGTAGAACCAGTTGAACCGAAGACAGAGATAGTCGAACCAGAAACAGAGCCAGTCGAAGAAGCCGAGGAAGAAGCGGTTGAAGCACCCGTTATGGAACCAGTTATACCTGAGATAGAACCAAAGACAGAACCGGAAGTTGAAGCCGAGGAAAAAGAAGTCGAAACAAACGTTGAACCAGAACTACTGGAGACAGGAAAACCGATTGAAATTGGCCAAGCAACTGAAATCGAGGAGCCACCAGAAAAACAACCGGTGGAATCGGAGTCACAACCAGCGGAAGTTGAGATGGAACAAATTGAAACAAAGGAAGAACAAACTGAACCAAAGGAAGAACTAGCAGGAATTGATGAGAAATGTAAGTTTTAGTAACAATTATTTATTTTATGATTCCTTTTGAGCTGTTTCGGTATGCTTCGATGCAAGTCAAATTTGTGGATGTTTTAAATCCATCATTCAATTATGTTTGCAATGTAGTCATGAAGTTTGTTCGTTTGTTCAACACAGATATGGGACATATACGCATGAGCAGACCTTTTTTGTGCACAGATCTTCGTTCAGGTTAATCGTGCATGGCAACAAAAATTGGTTCATTCCGTTTTATGTCGTGCAGTCTTATTTTGCTTGAATTAAGCATTGCTGTAAAGTTCAAGTTAAAGGTACAGTCCATCATTTGTAATTTGTGCATTTTGTTCGTTTGAAGAAACAAACATGCTCAAAATCTAAAGAAGGCTGCTGAACAACTAACCT---------------------------------------------------------------------------------------------------------------------------------------------------------------------------------------------------------------------------------------------------------------------------------------GATGAAGTTTCAGCTCAGCTTCAGCTGTATAAATTATTTCGAGAAAAACAAAATTCTAGGATCTCCATTTCAAATCAAAAGTTGCTGAATCTTGTTTTGAATTGGGCCTGAATTCAGCGTCCGAGGCACGTTCTCATCACTGAGCCGATTGCAGCTACGCGATGTCGCGCACGAATTAAAGCAATCGAGGGCGCGTGTGCGTTGTGTCAACGCTTGCGCGCGCTTCGCCAAATAAAAAAAAAAGAACTAAACGGAAATTTCTTTAGACGCATGTCTTTAGACGCACCGTTGGAAAGGCCTACTTTCGTCGCTTTCTTCAAAATGACAGCATGCCAGAAAGAAAAATCTCTTATCTTTCAACGAAGTATGTTTTTTAACAATAGGCCTAATTACGTCAGTTACTTTTTGGATAGTTGCAAATAATGATGGACTGTAGCTTTAAGAAATTTGTGGAACTCAAGTTGTGCTTGGCAAGTTTTACTTTAGAATTCGAAAATGCATCGACACCAAAAAAAAGTGTAATTTTGGTCCTATAGTTGAAGTGTCAAACTAGAACAAATTTAATGATAAATTAAATAAATTTAAATAATTTATTCCTACCTTGCTTTGACAGTGATGAAGGAGCTGCGTGACCTTTTGGAATCTACGAAGATTGACCTTCCTGTTGACATCAATGATCCATACGACCTAGGTCTTCTTCTCAGACATTTACGTCACCATTCAAATCTTCTTGCTCGTATTGGAGACCCCGATGTCAAAAAGGAAGTCCTCAGCGCCATGAATGAAAAC

>SS98-B

GCACGACGTGCCGCAGAGGAAAAACCTCAAAAGGGAAAGAAGGGACGAAAAGGAAAGAAACGACGAAAGGGAAAGAAGGGACGAAAAGGAAAGAAGGGACGAAAAGGAAAGAAACGACGAAAGGGAAAGAAAAGACGAAAGGGAAAGAAAGGACGAAAGGGAAAGAAAGGACGAAAGGGAAAGAAAGGACGAAAGGGAAAGAAAGGACGAAAGGGAAAGAAAGGACGAAAGGGAAAGAAAGGACGAAAGGGAAAGAAAGGACGAAAGGGAAAGAAAGGAAAACCAATCCAACCTGAAGAAACACCAGCCATTCCGACGGAGATAAAAGCTGCAGAAATAGAAAAGGAACCAAAAACAGAAGTGGTTGTGGAACCAGTTATTCCGGAAGCAGATATTGCGGAGACCGAAATACAACAAATTAAAGCAGAAGTAGAACCAGTTGAAACAAAGCCAGAAATAGTTATGGAACCAGATGTGGAACCAGTTATTCCAGAAACGGAACTTACAGAGACCGGAAAAGAAGCGAAAGTAGAACAAGTTAAACCAGTGGAAGGGAAGCTAAACTTA------------------------------------------------------------------------------------------------------------------------------------------------------------------------------------------------------------------------------------------------------------------------------------------------------------------------------------------------------------------------------------------------------------------------------------------------------------------------------GGAAAAGGAAAGGGCAAAGGAAAGAAGGTTAGAAAGGGAAAGAAAGGACGAAAGGGAAAGAAAGGACGAAAGGGAAAGAAAGGACGGAAAGGAAAGAAGGGGCGAAAAGGAAAGAAAAGAAGAAAAGGAAAAAAAGGACGAAAGGGAAAGAAAGGACGAAAGGGAAAGAAAGGACGAAAGGGCAAGAAAGGACGAAAGGGAAAGAAAGGACGAAAGGGAAAGAAAGGACGAAAGGGAAAGAAAGGACGAAGGGGAAAGAAAGGA------------------CGAAAGGGCAAGAAAGGAAATCCAATCAAAACTGAAGAAACACCAGCCATTCTGACGGAGATAAAAGCTGCAGAATTAGAAAAGGAACCAAAAACAGAAATAGTTATGAAACCAGTTATTCCCGAAACGGAACTAACAGAGACCGGAAAAGAAGCAGAAGTAGAACAAGTTAAACCAGTGGAAAGGAAGCTAAAAATAGGAAAAGGAAAGGGCAAAGGAAAGAAGGTTAGAAAGGGAAAGAAAGGACGAAAGGGAAAGAAAGGACGAAAGGGAAAGAAAGGACGGAAAGGAAAGAAGGGGCGAAAAGGAAAGAAAAGAAGAAAAGGAAAGAAAAGACGAAAAGGAAAGAAACGACGAAAGGGAAAGAAAGGACGAAAGGGAAAAAAAGGACGAAAGGGAAAGAAAGGACGAAAGGGAAAAAAAGGACGAAAGGGAAAAAAAGGACGAAAGGGAAAGAAAGGACGAAAGGGAAAGAAAGGCAAACCAATCCAAACTGAAGAAACACCAGCCATTCCGACGGAGATAAAAGCTGCAGAAATAGAAAAGGAACCAAAAACAGAAGTGGTTGTGGAACCACTTATTCAGGAAAAAGATGTTGCGGAGACCGAAATACAACCCATCGAAGCAGAAGTAGAACCAGTTGAACCAAAGACAGAAATAGTTATGGAACCAGTTATTCCGGAAGCAGATATTGCGGAGACCGAAATACAACCCATCAAAGCAGAAGAAGAACCAGTT------------------------------------------------------------------------------------------------------------GAACCAAAGCCAGAAATAGTTATGGAACCAGATGAGGAACCAGTTATTCCGGAAGCAGATATTGCGGAGACCGAAATACAACCCATCAAAGCAGAAGAAGAACCAGTTGAACCAAAGCCAGAAATAGTTATGGAACCAGATGAGGAACCAGTTATTCCGGAAGCAGATATTGCGGAGACCGAAATACAACCCATCAAAGCAGAAGAAGAACCAGTTGAACCAAAGCCAGAAATAGTTATGGAACCAGATGAGGAACCAGTTATTCCGGAAGCAGATATTGCGGAGACCGAAATACAACCCATCAGAGCAGAAGAAGAACCAGTTGAACCAAAGCCAGAAATAGTTATGGAACCAGATGAGGAACCAGTCATTCCAGAAACAGAACTAACAGAGACAGACAAACAACCAATCGAAGCAGAAGTAGAACCAGTTGAACCAAAAACAGAAGTGGTTGTGGAACCAGTTATTCCGGAAGCAGATATTGCGGAGACCGAAATACAACCCATCGAAGCAGAAGTAGAACCAGTTGAACCAAAGCCAGAAATAGTTATGGAACCAGATGTGGAACCAGTAATTCCAGAAACAGATCTTACAGAGACCGAAAAACAACCAATCGAAGCAGAAGTAGAACCAGTTGAACCGAAGACAGAGATAGTCGAACCAGAAACAGAGCCAGTCGAAGAAGCCGAGGAAGAAGCGGTTGAAGCACCCGTTATGGAACCAGTTATACCTGAGATAGAACCAAAGACAGAACCGGAAGTTGAAGCCGAGGAAAAAGAAGTCGAAACAAACGTTGAACCAGAACTACTGGAGACAGGAAAACCGATTGAAATTGGCCAAGCAACTGAAATCGAGGAGCCACCAGAAAAACAACCGGTGGAATCGGAGTCACAACCAGCGGAAGTTGAGATGGAACAAATTGAAACAAAGGAAGAACAAACTGAACCAAAGGAAGAACTAGCAGGAATTGATGAGAAATGTAAGTTTTAGTAACAATTATTTATTTTATGATTCCTTTTGAGCTGTTTCGGTATGCTTCGATGCAAGTCAAATTTGTGGATGTTTTAAATCCATCATTCAATTATGTTTGCAATGTAGTCATGAAGTTTGTTCGTTTGTTCAACACAGATATGGGACATATACGCATGAGCAGACCTTTTTTGTGCACAGATCTTCGTTCAGGTTAATCGTGCATGGCAACAAAAATTGGTTCATTCTGTTTTATGTCGTGCAGTCTTATTTTGCTTGAATTAAGCATTGCTGTAAAGTTCAAGTTAAAGGTACAGTCCATCATTTGTAATTTGTGCATTTTGTTCGTTTGAAGAAACAAACATGCTCAAAATCTAAAGAAGGCTGCTGAACAACTAACCT---------------------------------------------------------------------------------------------------------------------------------------------------------------------------------------------------------------------------------------------------------------------------------------GATGAAGTTTCAGCTCAGCTTCAGCTGTATAAATTATTTCGAGAAAAACAAAATTCTAGGATCTCCATTTCAAATCAAAAGTTGCTGAATCTTGTTTTGAATTGGGCCTGAATTCAGCGTCCGAGGCACGTTCTCATCACTGAGCCGATTGCAGCTACGCGATGTCGCGCACGAATTAAAGCAATCNAGGGCGCGTGTGCGTTGTGTCAACGCTTGCGCGCGCTTCGCCAAATAAAAAAAAAAGAACTAAACGGAAATTTCTTTAGACGCATGTCTTTAGACGCACCGTTGGAAAGGCCTACTTTCGTTGCTTTCTTCAAAATGACAGCATGCCAGAAAGAAAAATCTCTTATCTTTCAACGAAGTATGTTTTTTAACAATAGGCCTAATTACGTCAGTTACTTTTTGGATAGTTGCAAATAATGATGGACTGTAGCTTTAAGAAATTTGTGGAACTCAAGTTGTGCTTGGCAAGTTTTACTTTAGAATTCGAAAATGCATCGACACCAAAAAAAAGTGTAATTTTGGTCCTATAGTTGAAGTGTCAAACTAGAACAAATTTAATGATAAATTAAATAAATTTAAATAATTTATTCCTACCTTGCTTTGACAGTGATGAAGGAGCTGCGTGACCTTTTGGAATCTACGAAGATTGACCTTCCTGTTGACATCAATGATCCATACGACCTAGGTCTTCTTCTCAGACATTTACGTCACCATTCAAATCTTCTTGCTCGTATTGGAGACCCCGATGTCAAAAAGGAAGTCCTCAGCGCCATGAATGAAAAC

>SS28-B

GCACGACGTGCCGCAGAGGAAAAACCTCAAAAGGGAAAGAAGGGACGAAAAGGAAAGAAACGACGAAAGGGAAAGAAGGGACGAAAAGGAAAGAAGGGACGAAAAGGAAAGAAACGACGAAAGGGAAAGAAAAGACGAAAGGGAAAGAAAGGACGAAAGGGAAAGAAAGGACGAAAGGGAAAGAAAGGACGAAAGGGAAAGAAAGGACGAAAGGGAAAGAAAGGACGAAAGGGAAAGAAAGGACGAAAGGGAAAGAAAGGA------------------AAACCAATCCAACCTGAAGAAACACCAGCCATTCCGACGGAGATAAAAGCTGCAGAAATAGAAAAGGAACCAAAAACAGAAGTGGTTGTGGAACCAGTTATTCCGGAAGCAGATATTGCGGAGACCGAAATACAACAAATTAAAGCAGAAGTAGAACCAGTTGAAACAAAGCCAGAAATAGTTATGGAACCAGATGTGGAACCAGTTATTCCAGAAACGGAACTTACAGAGACCGGAAAAGAAGCGAAAGTAGAACAAGTTAAACCAGTGGAAGGGAAGCTAAACTTA------------------------------------------------------------------------------------------------------------------------------------------------------------------------------------------------------------------------------------------------------------------------------------------------------------------------------------------------------------------------------------------------------------------------------------------------------------------------------GGAAAAGGAAAGGGCAAAGGAAAGAAGGTTAGAAAGGGAAAGAAAGGACGAAAGGGAAAGAAAGGACGAAAGGGAAAGAAAGGACGGAAAGGAAAGAAGGGGCGAAAAGGAAAGAAAAGAAGAAAAGGAAAAAAAGGACGAAAGGGAAAGAAAGGACGAAAGGGAAAGAAAGGACGAAAGGGCAAGAAAGGACGAAAGGGAAAGAAAGGACGAAAGGGAAAGAAAGGACGAAAGGGAAAGAAAGGACGAAGGGGAAAGAAAGGA------------------CGAAAGGGCAAGAAAGGAAATCCAATCAAAACTGAAGAAACACCAGCCATTCTGACGGAGATAAAAGCTGCAGAATTAGAAAAGGAACCAAAAACAGAAATAGTTATGAAACCAGTTATTCCCGAAACGGAACTAACAGAGACCGGAAAAGAAGCAGAAGTAGAACAAGTTAAACCAGTGGAAAGGAAGCTAAAAATAGGAAAAGGAAAGGGCAAAGGAAAGAAGGTTAGAAAGGGAAAGAAAGGACGAAAGGGAAAGAAAGGACGAAAGGGAAAGAAAGGACGGAAAGGAAAGAAGGGGCGAAAAGGAAAGAAAAGAAGAAAAGGAAAGAAAAGACGAAAAGGAAAGAAACGACGAAAGGGAAAGAAAGGACGAAAGGGAAAAAAAGGACGAAAGGGAAAGAAAGGACGAAAGGGAAAAAAAGGACGAAAGGGAAAAAAAGGACGAAAGGGAAAGAAAGGACGAAAGGGAAAGAAAGGCAAACCAATCCAAACTGAAGAAACACCAGCCATTCCGACGGAGATAAAAGCTGCAGAAATAGAAAAGGAACCAAAAACAGAAGTGGTTGTGGAACCACTTATTCAGGAAAAAGATGTTGCGGAGACCGAAATACAACCCATCGAAGCAGAAGTAGAACCAGTTGAACCAAAGACAGAAATAGTTATGGAACCAGTTATTCCGGAAGCAGATATTGCGGAGACCGAAATACAACCCATCAAAGCAGAAGAAGAACCAGTT------------------------------------------------------------------------------------------------------------GAACCAAAGCCAGAAATAGTTATGGAACCAGATGAGGAACCAGTTATTCCGGAAGCAGATATTGCGGAGACCGAAATACAACCCATCAAAGCAGAAGAAGAACCAGTTGAACCAAAGCCAGAAATAGTTATGGAACCAGATGAGGAACCAGTTATTCCGGAAGCAGATATTGCGGAGACCGAAATACAACCCATCAAAGCAGAAGAAGAACCAGTTGAACCAAAGCCAGAAATAGTTATGGAACCAGATGAGGAACCAGTTATTCCGGAAGCAGATATTGCGGAGACCGAAATACAACCCATCAGAGCAGAAGAAGAACCAGTTGAACCAAAGCCAGAAATAGTTATGGAACCAGATGAGGAACCAGTCATTCCAGAAACAGAACTAACAGAGACAGACAAACAACCAATCGAAGCAGAAGTAGAACCAGTTGAACCAAAAACAGAAGTGGTTGTGGAACCAGTTATTCCGGAAGCAGATATTGCGGAGACCGAAATACAACCCATCGAAGCAGAAGTAGAACCAGTTGAACCAAAGCCAGAAATAGTTATGGAACCAGATGTGGAACCAGTAATTCCAGAAACAGATCTTACAGAGACCGAAAAACAACCAATCGAAGCAGAAGTAGAACCAGTTGAACCGAAGACAGAGATAGTCGAACCAGAAACAGAGCCAGTCGAAGAAGCCGAGGAAGAAGCGGTTGAAGCACCCGTTATGGAACCAGTTATACCTGAGATAGAACCAAAGACAGAACCGGAAGTTGAAGCCGAGGAAAAAGAAGTCGAAACAAACGTTGAACCAGAACTACTGGAGACAGGAAAACCGATTGAAATTGGCCAAGCAACTGAAATCGAGGAGCCACCAGAAAAACAACCGGTGGAATCGGAGTCACAACCAGCGGAAGTTGAGATGGAACAAATTGAAACAAAGGAAGAACAAACTGAACCAAAGGAAGAACTAGTAGGAATTGATGAGAAATGTAAGTTTTAGTAACAATTATTTATTTTATGATTCCTTTTGAGCTGTTTCGGTATGCTTCGATGCAAGTCAAATTTGTGGATGTTTTAAATCCATCATTCAATTATGTTTGCAATGTAGTCATGAAGTTTGTTCGTTTGTTCAACACAGATATGGGACATATACGCATGAGCAGACCTTTTTTGTGCACAGATCTTCGTTCAGGTTAATCGTGCATGGCAACAAAAATTGGTTCATTCCGTTTTATGTCGTGCAGTCTTATTTTGCTTGAATTAAGCATTGCTGTAAAGTTCAAGTTAAAGGTACAGTCCATCATTTGTAATTTGTGCATTTTGTTCGTTTGAAGAAACAAACATGCTCAAAATCTAAAGAAGGCTGCTGAACAACTAACCT---------------------------------------------------------------------------------------------------------------------------------------------------------------------------------------------------------------------------------------------------------------------------------------GATGAAGTTTCAGCTCAGCTTCAGCTGTATAAATAATTTCGAGAAAAACAAAATTCTAGGATCTCCATTTCAAATCAAAAGTTGCTGAATCTTGTTTTGAATTGGGCCTGAATTCAGCGTCCGAGGCACGTTCTCATCACTGAGCCGATTGCAGCTACGCGATGTCGCGCACGAATTAAAGCAATCGAGGGCGCGTGTGCGTTGTGTCAACGCTTGCGCGCGCTTCGCCAAATAAAAAAAAAAGAACTAAACGGAAATTTCTTTAGACGCATGTCTTTAGACGCACCGTTGGAAAGGCCTACTTTCGTTGCTTTCTTCAAAATGACAGCATGCCAGAAAGAAAAATCTCTTATCTTTCAACGAAGTATGTTTTTTAACAATAGGCCTAATTACGTCAGTTACTTTTTGGATAGTTGCAAATAATGATGGACTGTAGCTTTAAGAAATTTGTGGAACTCAAGTTGTGCTTGGCAAGTTTTACTTTAGAATTCGAAAATGCATCGACACCAAAAAAAAGTGTAATTTTGGTCCTATAGTTGAAGTGTCAAACTAGAACAAATTTAATGATAAATTAAATAAATTTAAATAATTTATTCCTACCTTGCTTTGACAGTGATGAAGGAGCTGCGTGACCTTTTGGAATCTACGAAGATTGACCTTCCTGTTGACATCAATGATCCATACGACCTAGGTCTTCTTCTCAGACATTTACGTCACCATTCAAATCTTCTTGCTCGTATTGGAGACCCCGATGTCAAAAAGGAAGTCCTCAGCGCCATGAATGAAAAC

>SS28-A

GCACGACGTGCCGCAGAGGAAAAACCTCAAAAGGGAAAGAAGGGACGAAAAGGAAAGAAACGACGAAAGGGAAAGAAGGGACGAAAAGGAAAGAAGGGACGAAAAGGAAAGAAACGACGAAAGGGAAAGAAAAGACGAAAGGGAAAGAAAGGACGAAAGGGAAAGAAAGGACGAAAGGGAAAGAAAGGACGAAAGGGAAAGAAAGGACGAAAGGGAAAGAAAGGACGAAAGGGAAAGAAAGGACGAAAGGGAAAGAAAGGACGAAAGGGAAAGAAAGGAAAACCAATCCAACCTGAAGAAACACCAGCCATTCCGACGGAGATAAAAGCTGCAGAAATAGAAAAGGAACCAAAAACAGAAGTGGTTGTGGAACCAGTTATTCCGGAAGCAGATATTGCGGAGACCGAAATACAACAAATTAAAGCAGAAGTAGAACCAGTTGAAACAAAGCCAGAAATAGTTATGGAACCAGATGTGGAACCAGTTATTCCAGAAACGGAACTTACAGAGACCGGAAAAGAAGCGAAAGTAGAACAAGTTAAACCAGTGGAAGGGAAGCTAAACTTA------------------------------------------------------------------------------------------------------------------------------------------------------------------------------------------------------------------------------------------------------------------------------------------------------------------------------------------------------------------------------------------------------------------------------------------------------------------------------GGAAAAGGAAAGGGCAAAGGAAAGAAGGTTAGAAAGGGAAAGAAAGGACGAAAGGGAAAGAAAGGACGAAAGGGAAAGAAAGGACGGAAAGGAAAGAAGGGGCGAAAAGGAAAGAAAAGAAGAAAAGGAAAAAAAGGACGAAAGGGAAAGAAAGGACGAAAGGGAAAGAAAGGACGAAAGGGCAAGAAAGGACGAAAGGGAAAGAAAGGACGAAAGGGAAAGAAAGGACGAAAGGGAAAGAAAGGACGAAGGGGAAAGAAAGGA------------------CGAAAGGGCAAGAAAGGAAATCCAATCAAAACTGAAGAAACACCAGCCATTCTGACGGAGATAAAAGCTGCAGAATTAGAAAAGGAACCAAAAACAGAAATAGTTATGAAACCAGTTATTCCCGAAACGGAACTAACTGAGACCGGAAAAGAAGCAGAAGTAGAACAAGTTAAACCAGTGGAAAGGAAGCTAAAAATAGGAAAAGGAAAGGGCAAAGGAAAGAAGGTTAGAAAGGGAAAGAAAGGACGAAAGGGAAAGAAAGGACGAAAGGGAAAGAAAGGACGGAAAGGAAAGAAGGGGCGAAAAGGAAAGAAAAGAAGAAAAGGAAAGAAAAGACGAAAAGGAAAGAAACGACGAANGGGAAAGAAAGGACGAAAGGGAAAAAAAGGACGAAAGGGAAAGAAAGGACGAAAGGGAAAAAAAGGACGAAAGGGAAAAAAAGGACGAAAGGGAAAGAAAGGACGAAAGGGAAAGAAAGGCAAACCAATCCAAACTGAAGAAACACCAGCCATTCCGACGGAGATAAAAGCTGCAGAAATAGAAAAGGAACCAAAAACAGAAGTGGTTGTGGAACCACTTATTCAGGAAAAAGATGTTGCGGAGACCGAAATACAACCCATCGAAGCAGACGTAGAACCAGTTGAACCAAAGACAGAAATAGTTATGGAACCAGTTATTCCGGAAGCAGATATTGCGGAGACCGAAATACAACCCATCAAAGCAGAAGAAGAACCAGTT------------------------------------------------------------------------------------------------------------GAACCAAAGCCAGAAATAGTTATGGAACCAGATGAGGAACCAGTTATTCCGGAAGCAGATATTGCGGAGACCGAAATACAACCCATCAAAGCAGAAGAAGAACCAGTTGAACCAAAGCCAGAAATAGTTATGGAACCAGATGAGGAACCAGTTATTCCGGAAGCAGATATTGCGGAGACCGAAATACAACCCATCAAAGCAGAAGAAGAACCAGTTGAACCAAAGCCAGAAATAGTTATGGAACCAGATGAGGAACCAGTTATTCCGGAAGCAGATATTGCGGAGACCGAAATACAACCCATCAGAGCAGAAGAAGAACCAGTTGAACCAATGCCAGAAATAGTTATGGAACCAGATGAGGAACCAGTCATTCCAGAAACAGAACTAACAGAGACAGACAAACAACCAATCGAAGCAGAAGTAGAACCAGTTGAACCAAAAACAGAAGTGGTTGTGGAACCAGTTATTCCGGAAGCAGATATTGCGGAGACCGAAATACAACCCATCGAAGCAGAAGTAGAACCAGTTGAACCAAAGCCAGAAATAGTTATGGAACCAGATGTGGAACCAGTAATTCCAGAAACAGATCTTACAGAGACCGAAAAACAACCAATCGAAGCAGAAGTAGAACCAGTTGAACCGAAGACAGAGATAGTCGAACCAGAAACAGAGCCAGTCGAAGAAGCCGAGGAAGAAGCGGTTGAAGCACCCGTTATGGAACCAGTTATACCTGAGATAGAACCAAAGACAGAACCGGAAGTTGAAGCCGAGGAAAAAGAAGTCGAAACAAACGTTGAACCAGAACTACTGGAGACAGGAAAACCGATTGAAATTGGCCAAGCAACTGAAATCGAGGAGCCACCAGAAAAACAACCGGTGGAATCGGAGTCACAACCAGCGGAAGTTGAGATGGAACAAATTGAAACAAAGGAAGAACAAACTGAACCAAAGGAAGAACTAGCAGGAATTGATGAGAAATGTAAGTTTTAGTAACAATTATTTATTTTATGATTCCTTTTGAGCTGTTTCGGTATGCTTCGATGCAAGTCAAATTTGTGGATGTTTTAAATCCATCATTCAATTATGTTTGCAATGTAGTCATGAAGTTTGTTCGTTTGTTCAACACAGATATGGGACATATACGCATGAGCAGACCTTTTTTGTGCACAGATCTTCGTTCAGGTTAATCGTGCATGGCAACAAAAATTGGTTCATTCTGTTTTATGTCGTGCAGTCTTATTTTGCTTGAATTAAGCATTGCTGTAAAGTTCAAGTTAAAGGTACAGTCCATCATTTGTAATTTGTGCATTTTGTTCGTTTGAAGAAACAAACATGCTCAAAATCTAAAGAAGGCTGCTGAACAACTAACCT---------------------------------------------------------------------------------------------------------------------------------------------------------------------------------------------------------------------------------------------------------------------------------------GATGAAGTTTCAGCTCAGCTTCAGCTGTATAAATTATTTCGAGAAAAACAAAATTCTAGGATCTCCATTTCAAATCAAAAGTTGCTGAATCTTGTTTTGAATTGGGCCTGAATTCAGCGTCCGAGGCACGTTCTCATCACTGAGCCGATTGCAGCTACGCGATGTCGCGCACGAATTAAAGCAATCGAGGGCGCGTGTGCGTTGTGTCAACGCTTGCGCGCGCTTCGCCAAATAAAAAAAAAAGAACTAAACGGAAATTTCTTTAGACGCATGTCTTTAGACGCACCGTTGGAAAGGCCTACTTTCGTTGCTTTCTTCAAAATGACAGCATGCCAGAAAGAAAAATCTCTTATCTTTCAACGAAGTATGTTTTCTAACAATAGGCCTAATTACGTCAGTTACTTTTTGGATAGTTGCAAATAATGATGGACTGTAGCTTTAAGAAATTTGTGGAACTCAAGTTGTGCTTGGCAAGTTTTACTTTAGAATTCGAAAATGCATCGACACCAAAAAAAAGTGTAATTTTGGTCCTATAGTTGAAGTGTCAAACTAGAACAAATTTAATGATAAATTAAATAAATTTAAATAATTTATTCCTACCTTGCTTTGACAGTGATGAAGGAGCTGCGTGACCTTTTGGAATCTACGAAGATTGACCTTCCTGTTGACATCAATGATCCATACGACCTAGGTCTTCTTCTCAGACATTTACGTCACCATTCAAATCTTCTTGCTCGTATTGGAGACCCCGATGTCAAAAAGGAAGTCCTCAGCGCCATGAATGAAAAC

>SS75-A

GCACGACGTGCCGCAGAGGAAAAACCTCAAAAGGGAAAGAAGGGACGAAAAGGAAAGAAACGACGAAAGGGAAAGAAGGGACGAAAAGGAAAGAAGGGACGAAAAGGAAAGAAACGACGAAAGGGAAAGAAAAGACGAAAGGGAAAGAAAGGACGAAAGGGAAAGAAAGGACGAAAGGGAAAGAAAGGACGAAAGGGAAAGAAAGGACGAAAGGGAAAGAAAGGACGAAAGGGAAAGAAAGGACGAAAGGGAAAGAAAGGACGAAAGGGAAAGAAAGGAAAACCAATCCAACCTGAAGAAACACCAGCCATTCCGACGGAGATAAAAGCTGCAGAAATAGAAAAGGAACCAAAAACAGAAGTGGTTGTGGAACCAGTTATTCCGGAAGCAGATATTGCGGAGACCGAAATACAACAAATTAAAGCAGAAGTAGAACCAGTTGAAACAAAGCCAGAAATAGTTATGGAACCAGATGTGGAACCAGTTATTCCAGAAACGGAACTTACAGAGACCGGAAAAGAAGCGAAAGTAGAACAAGTTAAACCAGTGGAAGGGAAGCTAAACTTA------------------------------------------------------------------------------------------------------------------------------------------------------------------------------------------------------------------------------------------------------------------------------------------------------------------------------------------------------------------------------------------------------------------------------------------------------------------------------GGAAAAGGAAAGGGCAAAGGAAAGAAGGTTAGAAAGGGAAAGAAAGGACGAAAGGGAAAGAAAGGACGAAAGGGAAAGAAAGGACGGAAAGGAAAGAAGGGGCGAAAAGGAAAGAAAAGAAGAAAAGGAAAAAAAGGACGAAAGGGAAAGAAAGGACGAAAGGGAAAGAAAGGACGAAAGGGCAAGAAAGGACGAAAGGGAAAGAAAGGACGAAAGGGAAAGAAAGGACGAAAGGGAAAGAAAGGACGAAGGGGAAAGAAAGGA------------------CGAAAGGGCAAGAAAGGAAATCCAATCAAAACTGAAGAAACACCAGCCATTCTGACGGAGATAAAAGCTGCAGAATTAGAAAAGGAACCAAAAACAGAAATAGTTATGAAACCAGTTATTCCCGAAACGGAACTAACAGAGACCGGAAAAGAAGCAGAAGTAGAACAAGTTAAACCAGTGGAAAGGAAGCTAAAAATAGGAAAAGGAAAGGGCAAAGGAAAGAAGGTTAGAAAGGGAAAGAAAGGACGAAAGGGAAAGAAAGGACGAAAGGGAAAGAAAGGACGGAAAGGAAAGAAGGGGCGAAAAGGAAAGAAAAGAAGAAAAGGAAAGAAAAGACGAAAAGGAAAGAAACGACGAAAGGGAAAGAAAGGACGAAAGGGAAAAAAAGGACGAAAGGGAAAGAAAGGACGAAAGGGAAAAAAAGGACGAAAGGGAAAAAAAGGACGAAAGGGAAAGAAAGGACGAAAGGGAAAGAAAGGCAAACCAATCCAAACTGAAGAAACACCAGCCATTCCGACGGAGATAAAAGCTGCAGAAATAGAAAAGGAACCAAAAACAGAAGTGGTTGTGGAACCACTTATTCAGGAAAAAGATGTTGCGGAGACCGAAATACAACCCATCGAAGCAGAAGTAGAACCAGTTGAACCAAAGACAGAAATAGTTATGGAACCAGTTATTCCGGAAGCAGATATTGCGGAGACCGAAATACAACCCATCAAAGCAGAAGAAGAACCAGTT------------------------------------------------------------------------------------------------------------GAACCAAAGCCAGAAATAGTTATGGAACCAGATGAGGAACCAGTTATTCCGGAAGCAGATATTGCGGAGACCGAAATACAACCCATCAAAGCAGAAGAAGAACCAGTTGAACCAAAGCCAGAAATAGTTATGGAACCAGATGAGGAACCAGTTATTCCGGAAGCAGATATTGCGGAGACCGAAATACAACCCATCAAAGCAGAAGAAGAACCAGTTGAACCAAAGCCAGAAATAGTTATGGAACCAGATGAGGAACCAGTTATTCCGGAAGCAGATATTGCGGAGACCGAAATACAACCCATCAGAGCAGAAGAAGAACCAGTTGAACCAAAGCCAGAAATAGTTATGGAACCAGATGAGGAACCAGTCATTCCAGAAACAGAACTAACAGAGACAGACAAACAACCAATCGAAGCAGAAGTAGAACCAGTTGAACCAAAAACAGAAGTGGTTGTGGAACCAGTTATTCCGGAAGCAGATATTGCGGAGACCGAAATACAACCCATCGAAGCAGAAGTAGAACCAGTTGAACCAAAGCCAGAAATAGTTATGGAACCAGATGTGGAACCAGTAATTCCAGAAACAGATCTTACAGAGACCGAAAAACAACCAATCGAAGCAGAAGTAGAACCAGTTGAACCGAAGACAGAGATAGTCGAACCAGAAACAGAGCCAGTCGAAGAAGCCGAGGAAGAAGCGGTTGAAGCACCCGTTATGGAACCAGTTATACCTGAGATAGAACCAAAGACAGAACCGGAAGTTGAAGCCGAGGAAAAAGAAGTCGAAACAAACGTTGAACCAGAACTACTGGAGACAGGAAAACCGATTGAAATTGGCCAAGCAACTGAAATCGAGGAGCCACCAGAAAAACAACCGGTGGAATCGGAGTCACAACCAGCGGAAGTTGAGATGGAACAAATTGAAACAAAGGAAGAACAAACTGAACCAAAGGAAGAACTAGCAGGAATTGATGAGAAATGTAAGTTTTAGTAACAATTATTTATTTTATGATTCCTTTTGAGCTGTTTCGGTATGCTTCGATGCAAGTCAAATTTGTGGATGTTTTAAATCCATCATTCAATTATGTTTGCAATGTAGTCATGAAGTTTGTTCGTTTGTTCAACACAGATATGGGACATATACGCATGAGCAGACCTTTTTTGTGCACAGATCTTCGTTCAGGTTAATCGTGCATGGCAACAAAAATTGGTTCATTCTGTTTTATGTCGTGCAGTCTTATTTTGCTTGAATTAAGCATTGCTGTAAAGTTCAAGTTAAAGGTACAGTCCATCATTTGTAATTTGTGCATTTTGTTCGTTTGAAGAAACAAACATGCTCAAAATCTAAAGAAGGCTGCTGAACAACTAACCT---------------------------------------------------------------------------------------------------------------------------------------------------------------------------------------------------------------------------------------------------------------------------------------GATGAAGTTTCAGCTCAGCTTCAGCTGTATAAATTATTTCGAGAAAAACAAAATTCTAGGATCTCCATTTCAAATCAAAAGTTGCTGAATCTTGTTTTGAATTGGGCCTGAATTCAGCGTCCGAGGCACGTTCTCATCACTGAGCCGATTGCAGCTACGCGATGTCGCGCACGAATTAAAGCAATCGAGGGCGCGTGTGCGTTGTGTCAACGCTTGCGCGCGCTTCGCCAAATAAAAAAAAAAGAACTAAACGGAAATTTCTTTAGACGCATGTCTTTAGACGCACCGTTGGAAAGGCCTACTTTCGTTGCTTTCTTCAAAATGACAGCATGCCAGAAAGAAAAATCTCTTATCTTTCAACGAAGTATGTTTTTTAACAATAGGCCTAATTACGTCAGTTACTTTTTGGATAGTTGCAAATAATGATGGACTGTAGCTTTAAGAAATTTGTGGAACTCAAGTTGTGCTTGGCAAGTTTTACTTTAGAATTCGAAAATGCATCGACACCAAAAAAAAGTGTAATTTTGGTCCTATAGTTGAAGTGTCAAACTAGAACAAATTTAATGATAAATTAAATAAATTTAAATAATTTATTCCTACCTTGCTTTGACAGTGATGAAGGAGCTGCGTGACCTTTTGGAATCTACGAAGATTGACCTTCCTGTTGACATCAATGATCCATACGACCTAGGTCTTCTTCTCAGACATTTACGTCACCATTCAAATCTTCTTGCTCGTATTGGAGACCCCGATGTCAAAAAGGAAGTCCTCAGCGCCATGAATGAAAAC

>SS75-B

GCACGACGTGCCGCAGAGGAAAAACCTCAAAAGGGAAAGAAGGGACGAAAAGGAAAGAAACGACGAAAGGGAAAGAAGGGACGAAAAGGAAAGAAGGGACGAAAAGGAAAGAAACGACGAAAGGGAAAGAAAAGACGAAAGGGAAAGAAAGGACGAAAGGGAAAGAAAGGACGAAAGGGAAAGAAAGGACGAAAGGGAAAGAAAGGACGAAAGGGAAAGAAAGGACGAAAGGGAAAGAAAGGACGAAAGGGAAAGAAAGGACGAAAGGGAAAGAAAGGAAAACCAATCCAACCTGAAGAAACACCAGCCATTCCGACGGAGATAAAAGCTGCAGAAATAGAAAAGGAACCAAAAACAGAAGTGGTTGTGGAACCAGTTATTCCGGAAGCAGATATTGCGGAGACCGAAATACAACAAATTAAAGCAGAAGTAGAACCAGTTGAAACAAAGCCAGAAATAGTTATGGAACCAGATGTGGAACCAGTTATTCCAGAAACGGAACTTACAGAGACCGGAAAAGAAGCGAAAGTAGAACAAGTTAAACCAGTGGAAGGGAAGCTAAACTTAGGAAAAGGAAAGGGCAAAGGAAAGAAGGTTAGAAAGGGAAAGAAAGGACGAAAGGGAAAGAAAGGACGAAAGGGAAAGAAAGGACGGAAAGGAAAGAAGGGGCGAAAAGGAAAGAAAAGAAGAAAAGGAAAAAAAGGACGAAAGGGAAAGAAAGGACGAAAGGGAAAGAAAGGACGAAAGGGCAAGAAAGGACGAAAGGGAAAGAAAGGACGAAAGGGAAAGAAAGGACGAAAGGGAAAGAAAGGACGAAGGGGAAAGAAAGGACGAAAGGGCAAGAAAGGAAATCCAATCAAAACTGAAGAAACACCAGCCATTCTGACGGAGATAAAAGCTGCAGAATTAGAAAAGGAACCAAAAACAGAAATAGTTATGAAACCAGTTATTCCCGAAACGGAACTAACAGAGACCGGAAAAGAAGCAGAAGTAGAACAAGTTAAACCAGTGGAAAGGAAGCTAAAAATAGGAAAAGGAAAGGGCAAAGGAAAGAAGGTTAGAAAGGGAAAGAAAGGACGAAAGGGAAAGAAAGGACGAAAGGGAAAGAAAGGACGGAAAGGAAAGAAGGGGCGAAAAGGAAAGAAAAGAAGAAAAGGAAAAAAAGGACGAAAGGGAAAGAAAGGACGAAAGGGAAAGAAAGGACGAAAGGGCAAGAAAGGACGAAAGGGAAAGAAAGGACGAAAGGGAAAGAAAGGACGAAAGGGAAAGAAAGGACGAAGGGGAAAGAAAGGA------------------CGAAAGGGCAAGAAAGGAAATCCAATCAAAACTGAAGAAACACCAGCCATTCTGACGGAGATAAAAGCTGCAGAATTAGAAAAGGAACCAAAAACAGAAATAGTTATGAAACCAGTTATTCCCGAAACGGAACTAACAGAGACCGGAAAAGAAGCAGAAGTAGAACAAGTTAAACCAGTGGAAAGGAAGCTAAAAATAGGAAAAGGAAAGGGCAAAGGAAAGAAGGTTAGAAAGGGAAAGAAAGGACGAAAGGGAAAGAAAGGACGAAAGGGAAAGAAAGGACGGAAAGGAAAGAAGGGGCGAAAAGGAAAGAAAAGAAGAAAAGGAAAGAAAAGACGAAAAGGAAAGAAACGACGAAAGGGAAAGAAAGGACGAAAGGGAAAAAAAGGACGAAAGGGAAAGAAAGGACGAAAGGGAAAAAAAGGACGAAAGGGAAAAAAAGGACGAAAGGGAAAGAAAGGACGAAAGGGAAAGAAAGGCAAACCAATCCAAACTGAAGAAACACCAGCCATTCCGACGGAGATAAAAGCTGCAGAAATAGAAAAGGAACCAAAAACAGAAGTGGTTGTGGAACCACTTATTCAGGAAAAAGATGTTGCGGAGACCGAAATACAACCCATCGAAGCAGAAGTAGAACCAGTTGAACCAAAGACAGAAATAGTTATGGAACCAGTTATTCCGGAAGCAGATATTGCGGAGACCGAAATACAACCCATCAAAGCAGAAGAAGAACCAGTT------------------------------------------------------------------------------------------------------------GAACCAAAGCCAGAAATAGTTATGGAACCAGATGAGGAACCAGTTATTCCGGAAGCAGATATTGCGGAGACCGAAATACAACCCATCAAAGCAGAAGAAGAACCAGTTGAACCAAAGCCAGAAATAGTTATGGAACCAGATGAGGAACCAGTTATTCCGGAAGCAGATATTGCGGAGACCGAAATACAACCCATCAAAGCAGAAGAAGAACCAGTTGAACCAAAGCCAGAAATAGTTATGGAACCAGATGAGGAACCAGTTATTCCGGAAGCAGATATTGCGGAGACCGAAATACAACCCATCAGAGCAGAAGAAGAACCAGTTGAACCAAAGCCAGAAATAGTTATGGAACCAGATGAGGAACCAGTCATTCCAGAAACAGAACTAACAGAGACAGACAAACAACCAATCGAAGCAGAAGTAGAACCAGTTGAACCAAAAACAGAAGTGGTTGTGGAACCAGTTATTCCGGAAGCAGATATTGCGGAGACCGAAATACAACCCATCGAAGCAGAAGTAGAACCAGTTGAACCAAAGCCAGAAATAGTTATGGAACCAGATGTGGAACCAGTAATTCCAGAAACAGATCTTACAGAGACCGAAAAACAACCAATCGAAGCAGAAGTAGAACCAGTTGAACCGAAGACAGAGATAGTCGAACCAGAAACAGAGCCAGTCGAAGAAGCCGAGGAAGAAGCGGTTGAAGCACCCGTTATGGAACCAGTTATACCTGAGATAGAACCAAAGACAGAACCGGAAGTTGAAGCCGAGGAAAAAGAAGTCGAAACAAACGTTGAACCAGAACTACTGGAGACAGGAAAACCGATTGAAATTGGCCAAGCAACTGAAATCGAGGAGCCACCAGAAAAACAACCGGTGGAATCGGAGTCACAACCAGCGGAAGTTGAGATGGAACAAATTGAAACAAAGGAAGAACAAACTGAACCAAAGGAAGAACTAGCAGGAATTGATGAGAAATGTAAGTTTTAGTAACAATTATTTATTTTATGATTCCTTTTGAGCTGTTTCGGTATGCTTCGATGCAAGTCAAATTTGTGGATGTTTTAAATCCATCATTCAATTATGTTTGCAATGTAGTCATGAAGTTTGTTCGTTTGTTCAACACAGATATGGGACATATACGCATGAGCAGACCTTTTTTGTGCACAGATCTTCGTTCAGGTTAATCGTGCATGGCAACAAAAATTGGTTCATTCTGTTTTATGTCGTGCAGTCTTATTTTGCTTGAATTAAGCATTGCTGTAAAGTTCAAGTTAAAGGTACAGTCCATCATTTGTAATTTGTGCATTTTGTTCGTTTGAAGAAACAAACATGCTCAAAATCTAAAGAAGGCTGCTGAACAACTAACCT---------------------------------------------------------------------------------------------------------------------------------------------------------------------------------------------------------------------------------------------------------------------------------------GATGAAGTTTCAGCTCAGCTTCAGCTGTATAAATTATTTCGAGAAAAACAAAATTCTAGGATCTCCATTTCAAATCAAAAGTTGCTGAATCTTGTTTTGAATTGGGCCTGAATTCAGCGTCCGAGGCACGTTCTCATCACTGAGCCGATTGCAGCTACGCGATGTCGCGCACGAATTAAAGCAATCGAGGGCGCGTGTGCGTTGTGTCAACGCTCGCGCGCGCTTCGCCAAATAAAAAAAAAAGAACTAAACGGAAATTTCTTTAGACGCATGTCTTTAGACGCACCGTTGGAAAGGCCTACTTTCGTTGCTTTCTTCAAAATGACAGCATGCCAGAAAGAAAAATCTCTTATCTTTCGACGAAGTATGTTTTTTAACAATAGGCCTAATTACGTCAGTTACTTTTTGGATAGTTGCAAATAATGATGGACTGTAGCTTTAAGAAATTTGTGGAACTCAAGTTGTGCTTGGCAAGTTTTACTTTAGAATTCGAAAATGCATCGACACCAAAAAAAAGTGTAATTTTGGTCCTATAGTTGAAGTGTCAAACTAGAACAAATTTAATGATAAATTAAATAAATTTAAATAATTTATTCCTACCTTGCTTTGACAGTGATGAAGGAGCTGCGTGACCTTTTGGAATCTACGAAGATTGACCTTCCTGTTGACATCAATGATCCATACGACCTAGGTCTTCTTCTCAGACATTTACGTCACCATTCAAATCTTCTTGCTCGTATTGGAGACCCCGATGTCAAAAAGGAAGTCCTCAGCGCCATGAATGAAAAC

>AK51-A

GCACGACGTGCCGCAGAGGAAAAACCTCAAAAGGGAAAGAAGGGACGAAAAGGAAAGAAACGACGAAAGGGAAAGAAGGGACGAAAAGGAAAGAAGGGACGAAAAGGAAAGGAACGACGAAAGGGAAAGAAAAGACGAAAGGGAAAGAAAGGACGAAAGGGAAAGAAAGGACGAAAGGGAAAGAAAGGACGAAAGGGAAAGAAAGGACGAAAGGGAAAGAAACGACGAAAGGGAAAGAAAGGACGAAAGGGAAAGAAAGGACGAAAGGGAAAGAAAGGAAAACCAATCCAACCTGAAGAAACACCAGCCATTCCGACGGAGATAAAAGCTGCAGAAATAGAAAAGGAACCAAAAACAGAAGTGGTTGTGGAACCAGTTATTCCGGAAGCAGATATTGCGGAGACCGAAATACAACAAATTAAAGCAGAAGTAGAACCAGTTGAAACAAAGCCAGAAATAGTTATGGAACCAGATGTGGAACCAGTTATTCCAGAAACGGAACTTACAGAGACCGGAAAAGAAGCGAAAGTAGAACAAGTTAAACCAGTGGAAGGGAAGCTAAACTTA------------------------------------------------------------------------------------------------------------------------------------------------------------------------------------------------------------------------------------------------------------------------------------------------------------------------------------------------------------------------------------------------------------------------------------------------------------------------------GGAAAAGGAAAGGGCAAAGGAAAGAAGGTTAGAAAGGGAAAGAAAGGACGAAAGGGAAAGAAAGGACGAAAGGGAAAGAAAGGACGGAAAGGAAAGAAGGGGCGAAAAGGAAAGAAAAGAAGAAAAGGAAAAAAAGGACGAAAGGGAAAGAAAGGACGAAAGGGAAAGAAAGGACGAAAGGGCAAGAAAGGACGAAAGGGAAAGAAAGGACGAAAGGGAAAGAAAGGACGAAAGGGAAAGAAAGGACGAAGGGGAAAGAAAGGA------------------CGAAAGGGCAAGAAAGGAAATCCAATCAAAACTGAAGAAACACCAGCCATTCTGACGGAGATAAAAGCTGCAGAATTAGAAAAGGAACCAAAAACAGAAATAGTTATGAAACCAGTTATTCCCGAAACGGAACTAACAGAGACCGGAAAAGAAGCAGAAGTAGAACAAGTTAAACCAGTGGAAAGGAAGCTAAAAATAGGAAAAGGAAAGGGCAAAGGAAAGAAGGTTAGAAAGGGAAAGAAAGGACGAAAGGGAAAGAAAGGACGAAAGGGAAAGAAAGGACGAAAAGGAAAGAAGGGGCGAAAAGGAAAGAAAAGAAGAAAAGGAAAGAAAAGACGAAAAGGAAAGAAACGACGAAAGGGAAAGAAAGGACGAAAGGGAAAAAAAGGACGAAAGGGAAAGAAAGGACGAAAGGGAAAAAAAGGACGAAAGGGAAAAAAAGGACGAAAGGGAAAGAAAGGACGAAAGGGAAAGAAAGGCAAACCAATCCAAACTGAAGAAACACCAGCCATTCCGACGGAGATAAAAGCTGCAGAAATAGAAAAGGAACCAAAAACAGAAGTGGTTGTGGAACCACTTATTCAGGAAAAAGATGTTGCGGAGACCGAAATACAACCCATCGAAGCAGAAGTAGAACCAGTTGAACCAAAGACAGAAATAGTTATGGAACCAGTTATTCCGGAAGCAGATATTGCGGAGACCGAAATACAACCCATCAAAGCAGAAGAAGAACCAGTT------------------------------------------------------------------------------------------------------------GAACCAAAGCCAGAAATAGTTATGGAACCAGATGAGGAACCAGTTATTCCGGAAGCAGATATTGCGGAGACCGAAATACAACCCATCAAAGCAGAAGAAGAACCAGTTGAACCAAAGCCAGAAATAGTTATGGAACCAGATGAGGAACCAGTTATTCCGGAAGCAGATATTGCGGAGACCGAAATACAACCCATCAAAGCAGAAGAAGAACCAGTTGAACCAAAGCCAGAAATAGTTATGGAACCAGATGAGGAACCAGTTATTCCGGAAGCAGATATTGCGGAGACCGAAATACAACCCATCAGAGCAGAAGAAGAACCAGTTGAACCAAAGCCAGAAATAGTTATGGAACCAGATGAGGAACCAGTCATTCCAGAAACAGAACTAACAGAGACAGACAAACAACCAATCGAAGCAGAAGTAGAACCAGTTGAACCAAAAACAGAAGTGGTTGTGGAACCAGTTATTCCGGAAGCAGATATTGCGGAGACCGAAATACAACCCATCGAAGCAGAAGTAGAACCAGTTGAACCAAAGCCAGAAATAGTTATGGAACCAGATGTGGAACCAGTAATTCCAGAAACAGATCTTACAGAGACCGAAAAACAACCAATCGAAGCAGAAGTAGAACCAGTTGAACCGAAGACAGAGATAGTCGAACCAGAAACAGAGCCAGTCGAAGAAGCCGAGGAAGAAGCGGTTGAAGCACCCGTTATGGAACCAGTTATACCTGAGATAGAACCAAAGACAGAACCGGAAGTTGAAGCCGAGGAAAAAGAAGTCGAAACAAACGTTGAACCAGAACTACTGGAGACAGGAAAACCGATTGAAATTGGCCAAGCAACTGAAATCGAGGAGCCACCAGAAAAACAACCGGTGGAATCGGAGTCACAACCAGCGGAAGTTGAGATGGAACAAATTGAAACAAAGGAAGAACAAACTGAACCAAAGGAAGAACTAGCAGGAATTGATGAGAAATGTAAGTTTTAGTAACAATTATTTATTTTATGATTCCTTTTGAGCTGTTTCGGTATGCTTCGATGCAAGTCAAATTTGTGGATGTTTTAAATCCATCATTCAATTATGTTTGCAATGTAGTCATGAAGTTTGTTCGTTTGTTCAACACAGATATGGGACATATACACATGAGCAGACCTTTTTTGTGCACAGATCTTCGTTCAGGTTAATCGTGCATGGCAACAAAAATTGGTTCATTCTGTTTTATGTCGTGCAGTCTTATTTTGCTTGAATTAAGCATTGCTGTAAAGTTCAAGTTAAAGGTACAGTCCATCATTTGTAATTTGTGCATTTTGTTCGTTTGAAGAAACAAACATGCTCAAAATCTAAAGAAGGCTGCTGAACAACTAACCT---------------------------------------------------------------------------------------------------------------------------------------------------------------------------------------------------------------------------------------------------------------------------------------GATGAAGCTTCAGCTCAGCTTCAGCTGTATAAATTATTTCGAGAAAAACAAAATTCTAGGATCTCCATTTCAAATCAAAAGTTGCTGAATCTTGTTTTGAATTGGGCCTGAATTCAGCGTCCGAGGCACGTTCTCATCACTGAGCCGATTGCAGCTACGCGATGTCGCGCACGAATTAAAGCAATCGAGGGCGCGTGTGCGTTGTGTCAACGCTTGCGCGCGCTTCGCCAAATAAAAAAAAAAGAACTAAACGGAAATTTCTTTAGACGCATGTCTTTAGACGCACCGTTGGAAAGGCCTACTTTCGTTGCTTTCTTCAAAATGACAGCATGCCAGAAAGAAAAATCTCTTATCTTTCAACGAAGTATGTTTTTTAACAATAGGCCTAATTACGTCAGTTACTTTTTGGATAGTTGCAAATAATGATGGACTGTAGCTTTAAGAAATTTGTGGAACTCAAGTTGTGCTTGGCAAGTTTTACTTTAGAATTCGAAAATGCATCGACACCAAAAAAAAGTGTAATTTTGGTCCTATAGTTGAAGTGTCAAACTAGAACAAATTTAATGATAAATTAAATAAATTTAAATAATTTATTCCTACCTTGCTTTGACAGTGATGAAGGAGCTGCGTGACCTTTTGGAATCTACGAAGATTGACCTTCCTGTTGACATCAATGATCCATACGACCTAGGTCTTCTTCTCAGACATTTACGTCACCATTCAAATCTTCTTGCTCGTATTGGAGACCCCGATGTCAAAAAGGAAGTCCTCAGCGCCATGAATGAAAAC

>AK51-B

GCACGACGTGCCGCAGAGGAAAAACCTCAAAAGGGAAAGAAGGGACGAAAAGGAAAGAAACGACGAAAGGGAAAGAAGGGACGAAAAGGAAAGAAGGGACGAAAAGGAAAGAAACGACGAAAGGGAAAGAAAAGACGAAAGGGAAAGAAAGGACGAAAGGGAAAGAAAGGACGAAAGGGAAAGAAAGGACGAAAGGGAAAGAAAGGACGAAAGGGAAAGAAAGGACGAAAGGGAAAGAAAGGACGAAAGGGAAAGAAAGGACGAAAGGGAAAGAAAGGAAAACCAATCCAACCTGAAGAAACACCAGCCATTCCGACGGAGATAAAAGCTGCAGAAATAGAAAAGGAACCAAAAACAGAAGTGGTTGTGGAACCAGTTATTCCGGAAGCAGATATTGCGGAGACCGAAATACAACAAATTAAAGCAGAAGTAGAACCAGTTGAAACAAAGCCAGAAATAGTTATGGAACCAGATGTGGAACCAGTTAATCCAGAAACGGAACTTACAGAGACCGGAAAAGAAGCGAAAGTAGAACAAGTTAAACCAGTGGAAGGGAAGCTAAACTTA------------------------------------------------------------------------------------------------------------------------------------------------------------------------------------------------------------------------------------------------------------------------------------------------------------------------------------------------------------------------------------------------------------------------------------------------------------------------------GGAAAAGGAAAGGGCAAAGGAAAGAAGGTTAGAAAGGGAAAGAAAGGACGAAAGGGAAAGAAAGGACGAAAGGGAAAGAAAGGACGGAAAGGAAAGAAGGGGCGAAAAGGAAAGAAAAGAAGAAAAGGAAAAAAAGGACGAAAGGGAAAGAAAGGACGAAAGGGGAAGAAAGGACGAAAGGGCAAGAAAGGACGAAAGGGAAAGAAAGGACGAAAGGGAAAGAAAGGACGAAAGGGAAAGAAAGGACGAAGGGGAAAGAAAGGA------------------CGAAAGGGCAAGAAAGGAAATCCAATCAAAACTGAAGAAACACCAGCCATTCTGACGGAGATAAAAGCTGCAGAATTAGAAAAGGAACCAAAAACAGAAATAGTTATGAAACCAGTTATTCCCGAAACGGAAATAACAGAGACCGGAAAAGAAGCAGAAGTAGAACAAGTTAAACCAGTGGAAAGGAAGCTAAAAATAGGAAAAGGAAAGGGCAAAGGAAAGAAG------------------AGGAGAAAGGGAAAGAGAGGACGAAAGGGAAAGAAAGGACGGAAAGGAAAGAAGGGGCGT------------------AAAGGAAAGAAAAGACGAAAAGGAAAGAAACGACGAAAGGGAAAGAAAGGACGAAAGGGAAAAAAAGGACGAAAGGGAAAGAAAGGACGAAAGGGAAAAAAAGGACGAAAGGGAAAAAAAGGACGAAAGGGAAAGAAAGGACGAAAGGGAAAGAAAGGAAAACCAATCCAAACTGAAGAAACACCAGCCATTCCGACGGAGATAAAAGCTGCAGAAATAGAAAAGGAACCAAAAACAGAAGTGGTTGTGGAACCACTTATTCAGGAAAAAGATGTTGCGGAGACCGAAATACAACCCATCGAAGCAGAAGTAGAACCAGTTGAACCAAAGACAGAAATAGTTATGGAACCAGTTATTCCGGAAGCAGATATNGCGGAGACCGAAGTACAACCCATCAAAGCAGAAGAAGAACCAGTT------------------------------------------------------------------------------------------------------------GAACCAAAGCCAGAAATAGTTATGGAACCAGATGAGGAACCAGTTATTCCGGAAGCAGATATTGCGGAGACCGAAATACAACCCATCAAAGCAGAAGAAGAACCAGTTGAACCAAAGCCAGAAATAGTTATGGAACCAGATGAGGAACCAGTTATTCCGGAAGCAGATATTGCGGAGACCGAAATACAACCCATCAGAGCAGAAGAAGAACCAGTTGAACCAAAGCCAGAAATAGTTATGGAACCAGATGAGGAACCAGTCATTCCAGAAACAGATATTGCGGAGACCGAAATACAACCCATCAGAGCAGAAGAAGAACCAGTTGAACCAAAGCCAGAAATAGTTATGGAACCAGATGAGGAACCAGTCATTCCAGAAACAGAACTAACAGAGACAGACAAACAACCAATCGAAGCAGAAGTAGAACCAGTTGAACCAAAAACAGAAGTGGTTGTGGAACCAGTTATTCCGGAAGCAGATATTGCGGAGACCGAAATACAACCCATCGAAGCAGAAGTAGAACCAGTTGAACCAAAGCCAGAAATAGTTATGGAACCAGATGTGGAACCAGTAATTCCAGAAACAGATCTTACAGAGACCGAAAAACAACCAATCGAAGCAGAAGTAGAACCAGTTGAACCGAAGACAGAGATAGTCGAACCAGAAACAGGGCCAGTCGAAGAAGCCGAGGAAGAAGCGGTTGAAGCACCCGTTATGGAACCAGTTATACCTGAG------------ACAGAACCGGAAGTTGAAGCCGAGGAAAAAGAAGTCGAAACAAACGTTGAACCAGAACTACTGGAGACAGGAAAACCGATTGAAATTGGCCAAGCAACTGAAATCGAGGAGCCACCAGAAAAACAACCGGTGGAATCGGAGTCACAACCAGCGGAAGTTGAGATGGAACAAATTGAAACAAAGGAAGAACCAACTGAACCAAAGGAAGAACTAGCAGGAATTGATGAGAAATGTAAGTTTTAGTAACAATTATTTATTTTATGATTCCTTTTGAGCTGTTTCGGTATGCTTCGATGCAAGTCAAATTTGTGGATGTTTTAAATCCATCATTCAATTATGTTTGCAATGTAGTCATGAAGTTTGTTCGTTTGTTCAACACAGATATGGGACATATACGCATGAGCAGACCTTTTTTGTGCACAGATCTTCGTTCAGGTTAATCGTGCATGGCAACAAAAATTGGTTCATTCCGTTTTATGTCGTGCAGTCTTATTTTGCTTGAATTAAGCATTGCCGTAAAGTTCAAGTTAAAGGTACAGTCCATCATTTGTAATTTGTGCATTTTGTTCGTTTGAAGAAACAAACATGCTCAAAATCTAAAGAAGGCTGCTGAACAACTAACCT---------------------------------------------------------------------------------------------------------------------------------------------------------------------------------------------------------------------------------------------------------------------------------------GATGAAGTTTCAGCTCAGCTTCAGCTGTATAAATTATTTCGAGAAAAACAAAATTCTAGGATCTCCATTTCAAATAAAAAGTTGCTGAATCTTGTTTTGAATTGGGCCTGAATTCAGCGTCCGAGGCACGTTCTCATCACTGAGCCGATTGCAGCTACGCGATGTCGCGCACGAATTAAAGCAATCGAGGGCGCGTGTGCGTTGTGTCAACGCTTGCGCGCGCTTCGCCAAATAAAAAAAAAAGAACTAAACGGAAATTTCTTTAGACGCATGTCTTTAGACGCACCGTTGGAAAGGCCTACTTTCGTTGCTTTCTTCAAAATGACAGCATGCCAGAAAGAAAAATCTCTTATCTTTCAACGAAGTATGTTTTTTAACAATAGGCCTAATTACGTCAGTTACTTTTTGGATAGTTGCAAATAATGATGGACTGTAGCTTTAAGAAATTTGTGGAACTAAAGTTGTGCTTGGCAAGTTTTACTTTAGAATTCGAAAATGCATCGACACCAAAAAAAAGTGTAATTTTGGTCCTATAGTTGAAGTGTCAAACTAGAGCAAATTTAATGATAAATTAAATAAATTTAAATAATTTATTCCTACCTTGCTTTGACAGTGATGAAGGAGCTGCGTGACCTTTTGGAATCTACGAAGATTGACCTTCCTGTTGACATCAATGATCCATACGACCTAGGTCTTCTTCTCAGACATTTACGTCACCATTCAAATCTTCTTGCTCGTATTGGAGACCCCGATGTCAAAAAGGAAGTCCTCAGCGCCATGAATGAAAAC

>AK52-A
[truncated: 9,204 more chars]
